# Expedient synthesis of *E*-hydrazone esters and 1*H*-indazole scaffolds through heterogeneous single-atom platinum catalysis

**DOI:** 10.1126/sciadv.aay1537

**Published:** 2019-12-06

**Authors:** Cuibo Liu, Zhongxin Chen, Huan Yan, Shibo Xi, Kah Meng Yam, Jiajian Gao, Yonghua Du, Jing Li, Xiaoxu Zhao, Keyu Xie, Haisen Xu, Xing Li, Kai Leng, Stephen J. Pennycook, Bin Liu, Chun Zhang, Ming Joo Koh, Kian Ping Loh

**Affiliations:** 1 Department of Chemistry, National University of Singapore, 3 Science Drive 3, Singapore 117543, Singapore.; 2 Institute of Chemical and Engineering Sciences, Agency for Science, Technology and Research (A*STAR), 1 Pesek Road, Jurong Island, Singapore 627833, Singapore.; 3 Department of Physics, National University of Singapore, 3 Science Drive 3, Singapore 117543, Singapore.; 4 School of Chemical and Biomedical Engineering, Nanyang Technological University, 62 Nanyang Drive, Singapore 637459, Singapore.; 5 Department of Materials Science and Engineering, National University of Singapore, Singapore 117575, Singapore.; 6 State Key Laboratory of Solidification Processing, Center for Nano Energy Materials, Northwestern Polytechnical University, Xi’an 710072, China.

## Abstract

Unprotected *E*-hydrazone esters are prized building blocks for the preparation of 1*H*-indazoles and countless other N-containing biologically active molecules. Despite previous advances, efficient and stereoselective synthesis of these compounds remains nontrivial. Here, we show that Pt single atoms anchored on defect-rich CeO_2_ nanorods (Pt_1_/CeO_2_), in conjunction with the alcoholysis of ammonia borane, promotes exceptionally *E*-selective hydrogenation of α-diazoesters to afford a wide assortment of *N*-H hydrazone esters with an overall turnover frequency of up to 566 hours^−1^ upon reaction completion. The α-diazoester substrates could be generated in situ from readily available carboxylic esters in one-pot hydrogenation reaction. Utility is demonstrated through concise, scalable synthesis of 1*H*-indazole–derived pharmaceuticals and their ^15^N-labeled analogs. The present protocol highlights a key mechanistic nuance wherein simultaneous coordination of a Pt site with the diazo N═N and ester carbonyl motifs plays a central role in controlling stereoselectivity, which is supported by density functional theory calculations.

## Teaser

A heterogeneous Pt single-atom catalyst promotes E-selective synthesis of hydrazones en route to bioactive 1*H*-indazoles.

## INTRODUCTION

The advent of single-atom catalysis as a new frontier that integrates the merits of both homogeneous and heterogeneous catalysis (*1*, *2*) has garnered widespread attention since its seminal coinage in 2011 (*3*). Finely dispersed metal atoms with robust, well-characterized active centers, stabilized by judiciously designed support, are highly desirable in heterogeneous catalysis for a number of reasons: (i) their maximized atom utilization efficiency, high surface coverage, and uniform structure often translate to excellent selectivity, turnover numbers, and turnover frequencies (TOFs); (ii) they have reactive sites wherein the isolated atoms and their local coordination environment can be reliably elucidated through modern microscopy and spectroscopy techniques (*4*), facilitating useful active site structure-activity relationships to be established for rational catalyst design; and (iii) they are easily separable from the products and can be recycled without appreciable deterioration in catalytic performance. Discovery of new single-atom catalysts (SACs) that promote a variety of chemical transformations such as hydrogenation (*5*), oxidation (*6*), and C─C bond formation (*7*) (see note S1 for extended bibliography) has burgeoned in recent years, with the demonstration of superior activity/selectivity profiles (compared to metal clusters, nanoparticles, or homogeneous variants) (*8*) in many instances.

Notwithstanding these advances, critical challenges remain to be solved. Although SACs display distinct advantages in promoting gas-phase reactions (*9*), their utility under liquid-phase conditions is underdeveloped. This may be due to the instability of certain SACs in solution and insufficient activation of reactants by single metal sites under ambient conditions, where leaching and aggregation of metallic atoms diminish catalytic activity (*10*). Consequently, applications of single-atom catalysis to promote complex liquid-phase organic transformations that facilitate synthesis of biologically active molecules are scant (*11*). We sought to address this shortcoming by identifying highly stable and functional group tolerant heterogeneous SACs to promote liquid-phase reactions for the preparation of key building blocks en route to fine chemicals and specialty chemicals (e.g., pharmaceuticals and agrochemicals) (*12*).

### The key challenges and proposed solution

**Fig. 1:**
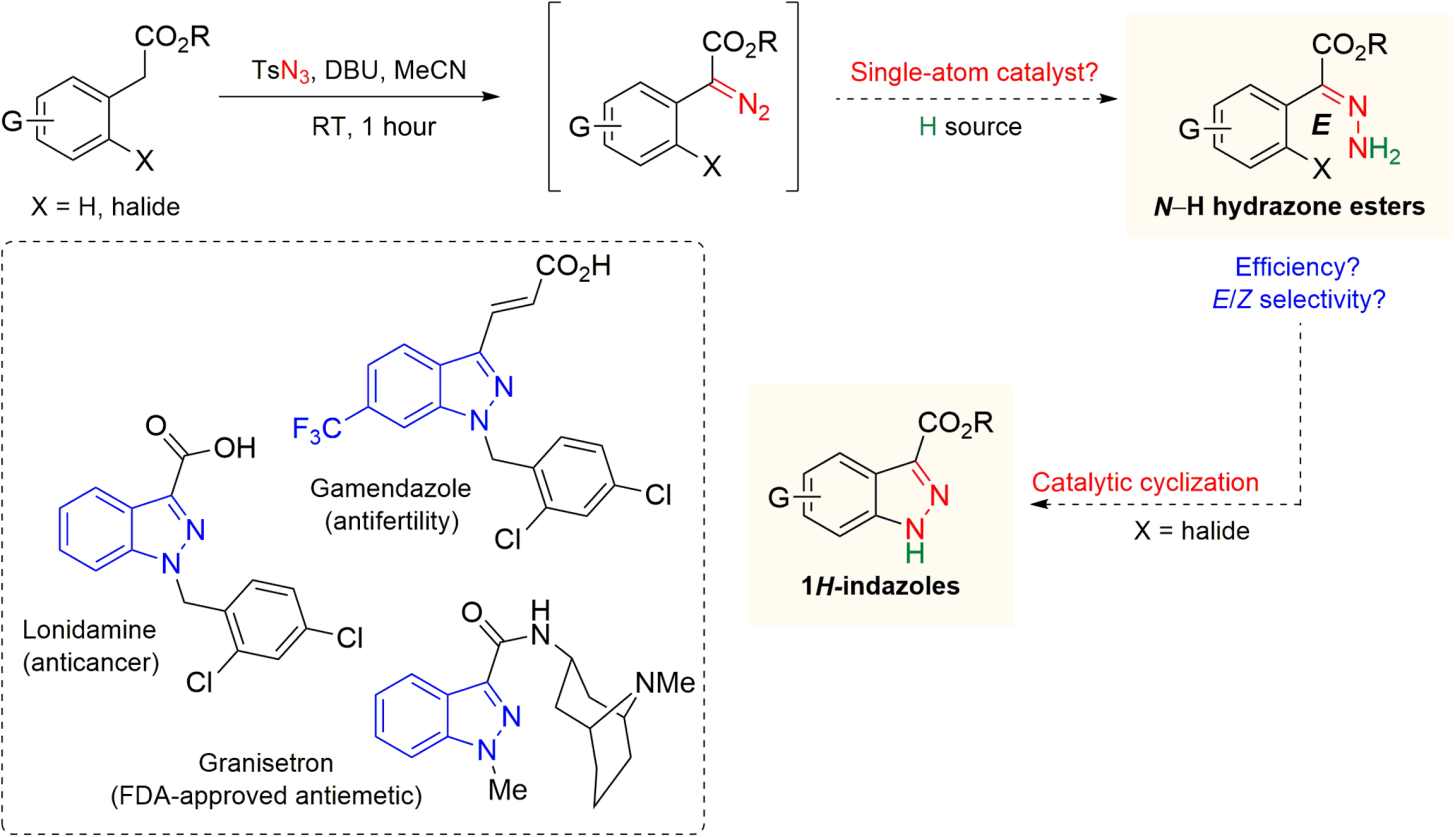
The significance and challenges in developing heterogeneous single-atom metal catalysts that furnish *E*-hydrazones and 1*H*-indazoles. 1*H*-Indazoles are common entities in medicinally relevant compounds (e.g., granisetron, lonidamine, and gamendazole in dashed box) and are conventionally derived from unprotected *E*-hydrazone precursors, of which synthesis is nontrivial. An attractive approach to *E*-hydrazones involves in situ diazo formation followed by catalytic hydrogenation in one sequence.

A long-standing limitation in chemical synthesis relates to the efficient construction of 1*H*-indazoles and their derivatives. These privileged heterocyclic compounds commonly reside in pharmacologically active entities (Fig. 1) that exhibit a broad range of anti-inflammatory, antidepressant, anticancer, and/or antifertility properties (*13*); examples include lonidamine (*14*), adjudin (*14*), gamendazole (*15*), and the U.S. Food and Drug Administration (FDA)–approved granisetron (*16*). A direct and convenient approach to assemble the 1*H*-indazole nucleus involves the synthesis of aryl-substituted *E*-α-hydrazone esters, followed by intramolecular cyclization with the neighboring aryl motif (*17*). Current methods that afford α-hydrazone esters typically involve the condensation of hydrazine with α-ketoesters under harsh acidic conditions (*18*) and sometimes require the use of sensitive reagents at cryogenic temperatures (*19*). Varying mixtures of *E*/*Z* isomers were generated from these reactions depending on substrate structure (*18*–*20*); thus, the overall approach is not sufficiently general. It merits mention that the *E*-hydrazone isomers are indispensable to the success of cyclization (*21*) and that *Z*-to-*E* isomerization typically requires heat or prolonged ultraviolet (UV) irradiation (*22*), which may give rise to undesired side reactions and/or substrate degradation.

Here, we proposed a reaction sequence that commences with in situ formation of α-diazoesters from simple carboxylic esters, followed by *E*-selective reduction of the diazo motif (catalyzed by an appropriate heterogeneous SAC) to give the desired *N*-H hydrazone esters for further transformation to 1*H*-indazoles (Fig. 1). Such a strategy offers the following unique advantages: (i) compared to conventionally used α-ketoesters, carboxylic esters are less costly and more widely available; (ii) severely acidic/basic and/or sensitive reagents which may be detrimental to certain functionalities can be precluded; (iii) unprotected α-hydrazone esters, which can be diversified to other important N-containing linear and heterocyclic compounds like *N*-acylhydrazones (*19*–*23*) and pyrroles (*24*), can be directly obtained without isolation of any diazo intermediates. An inherent challenge is that the *Z* isomers of *N*-H hydrazone esters, particularly the aryl-derived variants, are typically lower in energy and therefore favored by thermodynamics. This is supported by density functional theory (DFT) studies on the configurational stability of the hydrazone ester–free molecule. Thus, a kinetically controlled transformation that generates *E* isomers preferentially would have to overcome adventitious *E*-to-*Z* isomerization during the course of reaction. The central issue is the identification of an effective SAC system that is sufficiently stable, robust, and active to accomplish the key hydrogenation step with high selectivity under mild conditions.

## RESULTS

### Synthesis of an effective SAC

Although single-atom metal-catalyzed hydrogenation of organic compounds such as nitroarenes (*25*), carbonyl compounds (*5*), alkenes (*5*, *26*), alkynes (*8*, *26*), and phenols (*27*) (see note S2 for extended bibliography) has been described, none demonstrated utility for the preparation of complex bioactive molecules. A useful strategy in these transformations involves catalyst activation by the substrate/reagent to form a highly reactive metal species (such as a metal hydride), which are used to initiate subsequent reactions (*5*, *28*, *29*). For instance, Zheng and co-workers (*5*) discovered that reduction of alkenes and aldehydes is triggered by a Pd-H species generated in situ from H_2_ dissociation over Pd atoms, aided by neighboring oxygen on the support. Therefore, efficient and controlled formation of the putative metal hydride in the presence of a suitable SAC may offer a route toward stereoselective synthesis of *E*-hydrazones while minimizing side reactions like over-reduction or undesired hydrogenation of other functionalities. Our preliminary experiments showed that the activation of supported Pt catalysts (*30*) through alcoholysis of a hydrogen source [e.g., ammonia borane (*31*)] reliably generates the required metal-H species for diazo reduction.

**Fig. 2:**
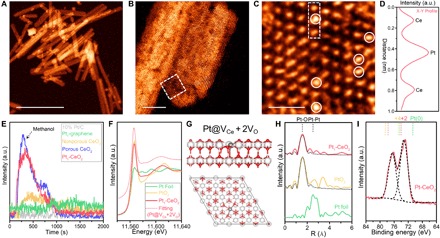
Evidence of Pt single atoms on porous CeO_2_ nanorods. (**A** and **B**) STEM-HAADF images; (**C**) atomic-resolution STEM-HAADF image of Pt_1_/CeO_2_ nanorods. (**D**) Intensity profile along the line in (C). (**E**) Temperature-programmed desorption (TPD) curves of the chemo-adsorption of methanol on various catalysts. (**F**) Pt L_3_-edge x-ray absorption near-edge spectroscopy (XANES) spectra and the calculated spectrum using the model (**G**) with the replacement of a Ce atom by a Pt single atom and two O vacancies next to a Pt single atom (Pt@V_Ce_ + 2V_O_). The color scheme used: white-gray for Ce; red for O; gray for Pt. Encut, 400 eV; convergence criterion, energy = 10^−4^; force = 0.02 eV Å. (**H**) Fourier-transformed extended x-ray absorption fine structure (FT-EXAFS) spectra of Pt foil, PtO_2_, and Pt_1_/CeO_2_ nanorods. Dash lines represent the fitting of EXAFS spectra. (**I**) High-resolution x-ray photoelectron spectroscopy (XPS) Pt_4f_ spectra of Pt_1_/CeO_2_ nanorods. Scale bars, 100 nm (A), 5 nm (B), and 1 nm (C). a.u., arbitrary units.

We fabricate a hybrid catalytic system based on Pt single atoms on porous, defect-rich CeO_2_ nanorods (Pt_1_/CeO_2_). CeO_2_ nanorods are endowed with the Ce^3+^/Ce^4+^ redox couple and structural defects due to edges and oxygen vacancies; the latter act as strong promoter sites and anchoring sites for SAC due to strong metal-substrate interaction (SMSI) (*9*). Defect-rich CeO_2_ nanorods were synthesized by a two-step hydrothermal reaction on gram scale, where the second step at 160°C was critical for the generation of oxygen vacancies as anchoring sites for Pt SACs (fig. S1 for nondefective CeO_2_ nanorods). The defects in CeO_2_ nanorods were revealed by scanning transmission electron microscopy in the annular dark-field mode (STEM-ADF) in Fig. 2A and fig. S2. Typical atomic force microscopy (AFM) images of the CeO_2_ nanorods reveal diameters of 4 to 8 nm (fig. S3). The absorption/desorption isothermal curves of defective CeO_2_ in fig. S4 reveals a type IV behavior with higher Brunauer-Emmett-Teller (BET) surface area than nondefective CeO_2_ (132 m^2^ g^−1^ versus 96 m^2^ g^−1^). Because of the abundance of O vacancies, Pt single atoms can be readily doped into the CeO_2_ nanorods by atomic layer deposition (ALD) (*32*). Atomic-resolution STEM-HAADF (high-angle annular dark-field) images in Fig. 2 (B and C) and fig. S5 confirm the uniform distribution of individual Pt atoms on CeO_2_, which are observed as bright spots overlapping with the Ce column in the lattice structure of CeO_2_ and marked with white circles. The Pt mass loading was determined as 1.38% by inductively coupled plasma optical emission spectroscopy (ICP-OES). The intensity profile along the line in Fig. 2C corroborates the presence of isolated Pt atoms. Energy-dispersive x-ray spectroscopy (EDS) elemental mapping in fig. S6 further supports the existence of Pt single atoms on CeO_2_ nanorods.

X-ray absorption near-edge spectroscopy (XANES) and extended x-ray absorption fine structure (EXAFS) profiles were recorded to determine the electronic structure and coordination of the Pt atoms in Pt_1_/CeO_2_. The white line of Pt L_3_-edge XANES of Pt_1_/CeO_2_ is similar to that of PtO_2_ in Fig. 2F, revealing that the Pt species in Pt_1_/CeO_2_ exists in the oxidized state. The combination of calculated and experimental XANES curves in Fig. 2F confirms the replacement of a Ce atom by a Pt single atom and the existence of two O vacancies nearby (Pt@V_Ce_ + 2V_O_). Other atomic configuration models from DFT-based structural optimization fail to reproduce the main features of experimental XANES curve, as shown in fig. S7, and have been ruled out. Fourier-transformed EXAFS (FT-EXAFS) spectrum also exhibits a prominent Pt-O peak at 1.56 Å, which was fitted and obtained a coordination number of 4 in table S1. No metallic Pt-Pt peak at 2.51 Å was observed for Pt_1_/CeO_2_, revealing that Pt_1_ exists as isolated single atoms, consistent with the HAADF-STEM data. The oxidation state of a Pt single atom is determined to be ~+4 from x-ray photoelectron spectroscopy (XPS), where the Pt_4f_ core-level spectrum consists of the spin-orbit doublets Pt 4f_7/2_ and 4f_5/2_ at 73.5 and 76.7 eV, respectively. These binding energies are much higher than that of Pt(0) species, which can be attributed to the formation of Pt─O bonding (Pt^2+^) and the SMSI between Pt single atoms and defective CeO_2_ nanorods. Moreover, the Ce species in defective CeO_2_ was determined to be a mixture of the Ce^3+^ and Ce^4+^ states in fig. S8, suggesting that O vacancies are abundant. This is confirmed by the stronger methanol chemical absorption for defective CeO_2_ than the nonporous counterpart in the temperature-programmed desorption (TPD) curves in Fig. 2E, whereas the benchmark 10% Pt/C and Pt_1_ SAC on graphene have very weak methanol absorption. Detailed characterization of Pt_1_/CeO_2_ and control samples could be found in figs. S9 to S11.

### *E*-selective synthesis of unprotected hydrazone esters

**Fig. 3:**
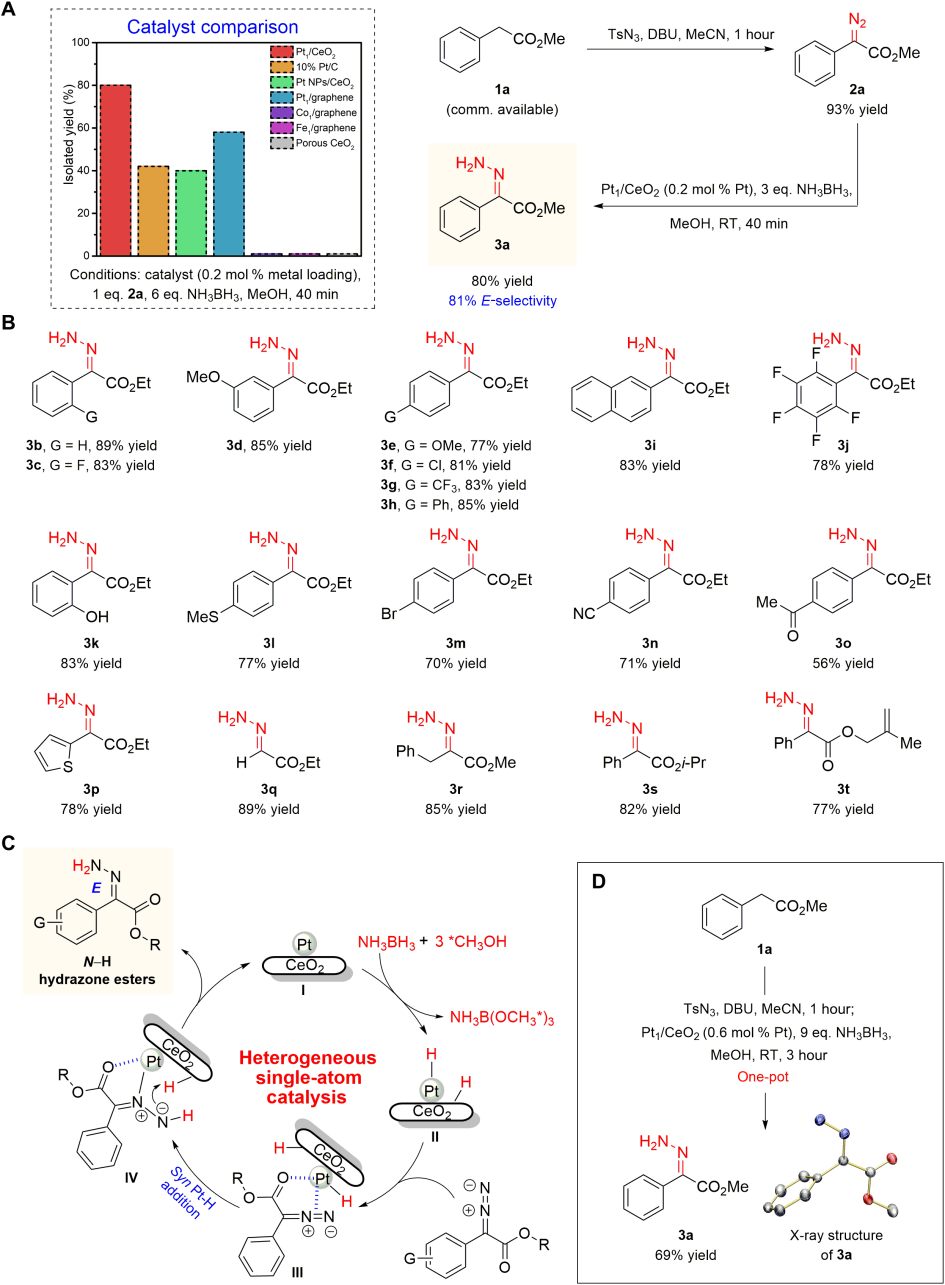
*E*-selective synthesis of *N*-H hydrazone esters. (**A**) Compared to other classes of Pt catalysts and non-noble metal-based SACs, Pt_1_/CeO_2_ exhibits superior activity in catalyzing hydrogenation of α-diazoesters to *E*-hydrazones. (**B**) A wide assortment of H-, alkyl-, and aryl(heteroaryl)-substituted *E*-hydrazone esters containing useful functional units can be accessed with Pt_1_/CeO_2_ catalyst. (**C**) Proposed catalytic cycle highlighting the importance of the ester moiety in directing regio- and stereoselective Pt-H addition across the diazo N═N bond (*MeOH depicts MeOH adsorbed on the surface of CeO_2_). (**D**) Direct conversion of readily available carboxylic esters to *N*-H *E*-hydrazones in a single vessel enhances the practicality of our catalytic method. The observed *E*-selectivity is up to 98% [determined by ^1^H NMR (nuclear magnetic resonance) analysis of the crude reaction mixture]. All isolated yields are of the pure *E* isomer. The reaction to obtain **3o** was performed with 0.6-equivalent NH_3_BH_3_ at 10°C for 3 hours. See methods S1 to S13 for details.

Control experiments revealed that Pt_1_/CeO_2_ SAC promoted release of H_2_ from the hydrolysis of ammonia borane in water at ambient temperature more rapidly than other Pt-based materials (fig. S12), which can be exploited for the hydrogenation of diazoesters. We began by investigating various heterogeneous catalytic systems in the hydrogenation of α-diazoester **2a** (prepared from methyl 2-phenylacetate **1a**) using excess ammonia borane in CH_3_OH (to ensure efficient generation of Pt-H species; Fig. 3A). With Pt_1_/CeO_2_ [0.2 mole percent (mol %) Pt], the reaction was completed within 40 min (81% *E*-selectivity), furnishing **3a** in 80% yield as the pure *E* isomer. The calculated TOF value for Pt_1_/CeO_2_ upon reaction completion is ~566 hours^−1^, which is much higher than other Pt-based catalysts such as commercial 10 weight % (wt %) Pt/C (~297 hours^−1^, 77% *E*-selectivity), Pt nanoparticles on CeO_2_ (~367 hours^−1^, 76% *E*-selectivity), and Pt_1_/graphene (~410 hours^−1^, 60% *E*-selectivity) (table S2, entries 4 to 6, and fig. S13). The less efficient utilization of Pt nanoparticles and the weak adsorption of methanol on carbon support account for the reduced catalytic efficiency, substantiating the importance of well-dispersed Pt atoms on defective CeO_2_ in promoting hydrogenation. Pt single atoms on nonporous CeO_2_ nanorods (denoted as Pt_1_/CeO_2_-non) gives a much lower Pt loading (0.85%; fig. S10) and a relatively poor performance (56% yield; table S2, entry 7), indicating the importance of defect engineering on catalytic performance. Although non-noble metal catalysts (e.g., Co and Fe based) have been reported to catalyze ammonia borane hydrolysis or alcoholysis (*33*), both Co_1_/graphene and Fe_1_/graphene were found to be ineffective under the reduction conditions, due perhaps to the insufficient activation of the diazo substrate (table S2, entries 8 and 9).

Additional investigations showed that other solvents were unsatisfactory, and both Pt and ammonia borane were essential for the hydrogenation reaction (table S3, entries 10 to 14). The loading of ammonia borane can be reduced to three equivalents without appreciable diminution in efficiency (table S4, entries 16 and 17). The use of 8 atm H_2_ to replace ammonia borane afforded **3a** in only 37% yield (table S4, entry 18), suggesting that generation of the Pt-H species through alcoholysis of ammonia borane is more efficient than direct Pt-promoted H_2_ dissociation. Other borane sources, including borane tetrahydrofuran complex, borane dimethyl sulfide complex, (CH_3_)_3_N·BH_3_, (CH_3_)_2_NH·BH_3_, and tBuNH_2_·BH_3_ were inferior or exhibited no activity despite longer reaction times (table S4, entries 19 to 23). These results corroborate the critical role of Pt_1_/CeO_2_ and ammonia borane in the stereoselective hydrogenation of diazo compounds to *E*-hydrazones. The Pt_1_/CeO_2_ catalyst can be recycled five times with a minor decrease in reaction efficiency from 89 to 81% (isolated yield of **3r**; fig. S14). The small drop in efficiency probably stems from the deposition of the B(OMe)_3_ by-product on the surface active sites of the catalyst (0.36% B residual from ICP-OES) (*31*, *34*). The leaching of Pt metal can be ruled out from the ICP-OES measurement with negligible difference between the fresh and spent catalysts (1.38% versus 1.37% Pt loading). This is also proven by the absence of Pt in the clear solution after the recovery of catalyst from reaction mixture. STEM and EXAFS results further revealed the intact single-atom nature of the spent catalyst in figs. S15 and S16, which can be attributed to the strong bonding of O with Pt in defect-rich CeO_2_.

To assess the generality of our established conditions, various α-diazoesters with different electronic and/or steric attributes were examined (Fig. 3B). Diazoesters that bear either electron-rich or electron-deficient aryl units are effective substrates, affording *N*-H hydrazone esters in 77 to 89% yield (**3b** to **3j**). Notably, **3j** may serve as a useful precursor for the preparation of polyfluorinated indazoles with neuroprotective activities (*35*). Commonly occurring and versatile functionalities such as a thioether (**3l**), a phenol (**3k**), a bromide (**3m**), a cyano group (**3n**), a ketone (**3o**), and an alkene (**3t**) were tolerated. The latter five examples that contain potentially reducible functional groups (*5*, *26*, *27*) highlight the remarkable chemoselectivity of the present hydrogenation protocol. Transformations with heterocyclic diazoesters and their H- and alkyl-substituted variants were similarly efficient, delivering the desired products in 85 to 89% yield (**3p** to **3r**). Likewise, isopropyl and allyl ester substrates underwent hydrogenation to deliver the corresponding *E*-hydrazones (**3s** and **3t**). In all instances, high stereoselectivity was observed (up to 98:2 *E*:*Z* ratios), and the *E* isomeric form could be isolated cleanly after simple chromatography purification.

As mentioned earlier, *Z* isomers of *N*-H hydrazone esters (especially those that are aryl substituted) are thermodynamically favored (fig. S17). The question is asked therefore on why high kinetic *E*-selectivity can be obtained for diazo reduction here. As illustrated in the catalytic cycle in Fig. 3C, we proposed that ammonia borane alcoholysis (*34*, *36*) in the presence of catalytic Pt_1_/CeO_2_ generates the putative Pt-H species **II** with concomitant protonation of a nearby oxygen on CeO_2_ (*5*). **II** can simultaneously interact with the weakly Lewis basic diazo and ester carbonyl motifs to give **III**, enabling regio- and stereoselective Pt-H addition across the N═N bond in a *syn* fashion to afford **IV**. Subsequent protonation of **IV** by a neighboring O-H unit then releases the stereo-defined *E* product and regenerates the catalyst. DFT calculations revealed that there is stronger adsorption of the diazo substrate on a Pt single atom than O vacancy of CeO_2_ (fig. S18 and table S5). Steric effects of the aryl ring appear to be insignificant since reactions with diazo substrates bearing relatively smaller groups (**3q** and **3r**) were similarly *E*-selective. Control experiments indicated that adventitious *Z*-to-*E* isomerization was minimal under the reaction conditions (fig. S19). DFT studies showed that the calculated magnitude of the adsorption energies of the *E* product adsorbed on the Pt catalyst is much larger than that of the corresponding *Z* isomer, providing further justification for the predominant formation of *E*-hydrazones in our system (figs. S20 to S22 and table S5).

In line with our initial proposition, we proceeded to test the feasibility of a one-pot process by combining diazo formation and reduction in a single vessel (Fig. 3D). Using Pt_1_/CeO_2_ (0.6 mol % Pt) in the presence of excess ammonia borane, the desired *E*-hydrazone **3a** (stereochemistry ascertained by x-ray crystal structure analysis) could be obtained in 69% yield within 3 hours. This result illustrates the reliability of the Pt catalytic system for efficient generation of unprotected hydrazone esters from simple ester molecules, an important strategy that we adopted for the concise synthesis of 1*H*-indazole scaffolds (see below for further discussion). For the sake of versatility and practical use, we also supplied gram-scale synthesis in fig. S23, wherein a reasonably high yield of 80% (1.7 g) could be obtained by our approach using model substrate **2a**.

### Synthesis of biologically active compounds

To demonstrate the utility of our catalytic method, we focused on devising new synthetic approaches to pharmaceutically important 1*H*-indazole-3-carboxylates. These molecules contain a versatile ester handle that may be further converted to other useful functionalities [e.g., hydrogen (**14**), bromine (**17**), 1,2,4-oxadiazole (**19**), alcohol (**20**); see note S3 and methods S1 to S13 for details].

**Fig. 4:**
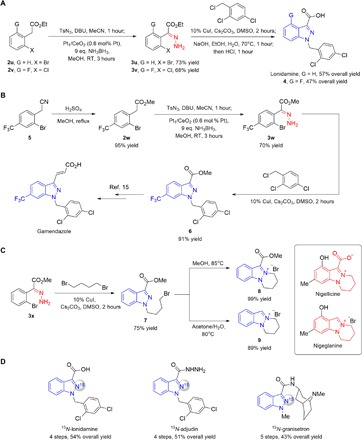
Synthesis of 1*H*-indazole–derived biologically active compounds. (**A**) Anticancer lonidamine was assembled in 42% overall yield by a concise two-pot sequence, which may be used to prepare derivatives such as **4**. (**B**) Formal synthesis of gamendazole, a drug candidate for male contraception, was accomplished in 61% overall yield within three steps through a 1*H*-indazole intermediate **6**. (**C**) The method is amenable to the construction of the tricyclic pyridazino[1,2-*a*]indazolium scaffold commonly found in alkaloids such as nigellicine and nigeglanine. (**D**) The versatility of our protocol is further highlighted through facile preparation of ^15^N-labeled analogs of key therapeutic agents. See methods S1 to S13 for details. DMSO, dimethyl sulfoxide.

The first application involves the synthesis of anticancer lonidamine (*14*) and its fluoro-analog **4** (Fig. 4A). Stereoselective access to *E*-hydrazones **3u** and **3v** from the corresponding aryl acetate esters through a single-pot diazo formation/hydrogenation followed by catalytic cyclization, alkylation, and hydrolysis furnished the desired products in 32 to 42% overall yields (see table S6 for comparison). The two-pot process compares favorably with previously reported multistep procedures to construct the 1*H*-indazole core (*14*, *17*, *37*, *38*) and is amenable to the preparation of different derivatives (e.g., **4**), which are otherwise difficult to access by alternative routes. It merits mention that synthesis of lonidamine can also be achieved on gram scale in four separate steps with an improved overall yield of 53% (see figs. S24 and S25).

In another instance, *E*-hydrazone ester **3w** obtained through a similar pathway as before, was subjected to catalytic cyclization/alkylation to afford 1*H*-indazole-3-carboxylate **6**, a known precursor en route to the male contraceptive drug candidate gamendazole (Fig. 4B) (*15*). Overall, **6** can be prepared in three steps with an overall yield of 61%, in contrast to a previous six-step sequence that used toxic thionyl chloride, generating the product in 40% overall yield (*15*).

Yet, another utility is the efficient construction of the fused tricyclic pyridazino[1,2-*a*]indazolium ring system present in bioactive alkaloids such as nigellicine and nigeglanine (*39*), which can be readily accomplished from *E*-hydrazone **3x** (Fig. 4C), further showcasing the advantage of our unprotected *E*-hydrazone strategy to facilitate preparation of complex N-containing molecules. Numerous other 1*H*-indazole–substituted pharmaceuticals can be obtained in similar fashion through the present protocol (see retrosynthetic analysis in fig. S26).

Last, the SAC-catalyzed strategy is highly versatile and can be extended to prepare the ^15^N-labeled analog of key therapeutic agents, with the objective of streamlining the process of target cloning, protein expression, screening, and preliminary protein folding/aggregation assessment by nuclear magnetic resonance (NMR) spectroscopy and other methods (*40*). In addition, ^15^N labeling studies may offer insights pertaining to the metabolism and degradation of N-containing drug molecules (*41*). ^15^N-labeled pharmaceuticals such as lonidamine, adjudin, and granisetron were synthesized in high yields, and the excellent ^15^N-labeling efficiency (using sodium azide-1-^15^N in α-diazoester formation) offers opportunities to study their pharmacokinetic and pharmacodynamic properties through metabolism investigations (Fig. 4D).

## CONCLUSIONS

We have demonstrated that Pt-based SAC anchored on defect-rich CeO_2_ nanorods (Pt_1_/CeO_2_) is capable of delivering selective access to unprotected *E*-hydrazone esters, under mild reaction conditions, and with ammonia borane as the hydrogen source. A wide range of functionalized α-diazoesters may be used, highlighting the exceptional robustness and functionality tolerance of the Pt-based catalyst. For example, the present method can be extended to practical one-pot procedures, where α-diazoesters generated in situ from commercial and readily available carboxylic esters are directly reduced with ammonia borane and catalytic amounts of Pt_1_/CeO_2_ to furnish the desired *E*-hydrazones in a single sequence. Implementation of the current protocol as the key step in the concise synthesis of several 1*H*-indazole–derived biologically active molecules and their ^15^N-labeled analogs demonstrates the viability of SACs as a powerful platform through which synthetically valuable building blocks can be assembled.

## MATERIALS AND METHODS

### Gram-scale synthesis of defect-rich CeO_2_ nanorods

CeO_2_ nanorods were prepared by hydrothermal reaction according to the literature (*42*). Ce(NO_3_)_3_·6H_2_O (1.736 g) and NaOH (19.2 g) were dissolved in 10 and 70 ml of deionized (DI) water and mixed under stirring for 30 min. The milky slurry was then transferred in a Teflon liner sealed tightly in a stainless-steel Parr autoclave. Hydrothermal reaction was carried out at 100°C for 24 hours. Crude products were separated by centrifugation, washed with DI water and ethanol to remove excess NaOH, and then re-dispersed in DI water at 2 mg ml^−1^ for the second-step hydrothermal reaction at 160°C for 12 hours to create surface oxygen vacancies on CeO_2_ nanorods. The products were dried at 80°C for 12 hours and lastly ground in a mortar and pestle to obtain pale yellow powders.

### Synthesis of Pt_1_/CeO_2_ SACs by ALD

The synthesis of Pt_1_ SACs was performed in a viscous ALD flow reactor (Plasma-assisted ALD system, Wuxi MNT Micro and Nanotech Co. Ltd., China) by alternatively exposing CeO_2_ nanorods to MeCpPtMe_3_ precursors and O_3_ at 150°C (*8*, *31*). Ultrahigh purity N_2_ (99.99%) was used as carrier gas with a flow rate of 50 ml min^−1^. The Pt precursors were heated at 100°C to generate a high enough vapor pressure. The reactor and reactor inlets were held at 150° and 120°C, respectively, to avoid any precursor condensation. An in situ thermal reduction of as-received CeO_2_ nanorods was conducted at 300°C for 5 min before performing Pt ALD. The timing sequence was 100, 120, 150, and 120 s for the MeCpPtMe_3_ exposure, N_2_ purge, O_3_ exposure, and N_2_ purge, respectively. Conducting Pt ALD with 1 cycle allows for the synthesis of Pt_1_/CeO_2_ with a Pt loading of 1.38 ± 0.02 wt %.

### One-pot synthesis of *E*-hydrazone esters from carboxylic esters

Typically, carboxylic ester (0.1 mmol), TsN_3_ (0.12 mmol), and CH_3_CN (0.3 ml) were added to a glass vial (4 ml). With stirring, 1,8-diazabicyclo[5,4,0]undec-7-ene (DBU) (0.12 mmol) was added dropwise. The reaction mixture stirred at room temperature (RT) for 1 hour. Then, 9 mg of Pt_1_/CeO_2_ catalysts and 2.0 ml of MeOH were added directly and sonicated for 15 min. After that, 0.9 mmol of ammonia borane was added. The vial was quickly sealed, and the reaction mixture was stirred at RT for another 3 hours. After reaction, the mixture was centrifuged to remove catalyst and washed three times with CH_2_Cl_2_. The supernatant was vapored under reduced pressure, and the residuals were subjected to be separated using thin-layer chromatography plate. The yield was calculated by dividing the amount of the obtained desired product by the theoretical yield. Details on reaction setup and synthesis of substrates can be found in methods S1 to S13.

### Synthesis of *E*-hydrazone esters from diazo compounds

Typically, 3 mg of Pt_1_/CeO_2_ catalysts was dispersed in 2.0 ml of MeOH and sonicated for 15 min. Then, 0.1 mmol of diazo compound and 0.3 mmol of ammonia borane were added sequentially. The vial (4 ml) was sealed quickly, and the reaction mixture was stirred at RT for 40 min. Other conditions remain identical to the one-pot synthesis from aryl acetate esters.

### Equipment

STEM/EDS (JEOL ARM200F equipped with ASCOR probe corrector, Oxford X-Max 100TLE, at 200 kV), XPS (Axis Ultra DLD monochromatic Al Kα), x-ray diffraction (XRD; Bruker D8), ICP-OES (Perkin Elmer 5300DV), NMR (Bruker AV300), gas chromatography–mass spectrometry (MS) (Agilent 5975 C inert MSD with triple-axis detector), MS (Bruker MicroTOF-QII), electron paramagnetic resonance (EPR) (Jeol FA200), Raman (Horiba Jobin Yvon), TPD (Quantachrome chemBET pulsar), AFM (Dimension Fast Scan), BET (Quantachrome Autosorb-iQ), and Fourier transform infrared (Varian 3100). XANES and EXAFS: 150 mg of sample was first ground into fine powder using a mortar and pestle before being pressed into a 10-mm pellet. Measurements were carried out at Singapore Synchrotron Light Source, x-ray absorption fine structure for catalysis beamline in fluorescence mode (*43*). Data analysis and simulation were carried out on Athena and Artemis (version 0.9.26) (*44*). Details on NMR spectra and single-crystal XRD data can be found in the Supplementary Materials.

## Supplementary Material

http://advances.sciencemag.org/cgi/content/full/5/12/eaay1537/DC1

Download PDF

Expedient synthesis of E-hydrazone esters and 1H-indazole scaffolds through heterogeneous single-atom platinum catalysis

## SUPPLEMENTARY MATERIALS

Supplementary material for this article is available at http://advances.sciencemag.org/cgi/content/full/5/12/eaay1537/DC1 Note S1. Expanded discussion on SACs catalyzed organic reactions Note S2. Expanded discussion on SACs catalyzed hydrogenation Note S3. Transformation of the ester moiety in the product to generate more complex molecules Method S1. Computational details Method S2. Synthesis of Co_1_/graphene and Fe_1_/graphene Method S3. Synthesis of Pt_1_/graphene Method S4. Synthesis of *E*-hydrazone esters **3o** Method S5. Total synthesis of lonidamine and F-containing lonidamine **4** Method S6. The synthesis of key intermediate **6** for gamendazole Method S7. The construction of tricyclic pyridazino[1,2-*a*]indazolium ring frameworks **8** and **9** Method S8. The synthesis of ^15^N-labeled lonidamine and adjudin Method S9. The synthesis of ^15^N-labeled granisetron Method S10. Product transformation by decarboxylation of ester to hydrogenation Method S11. Product transformation by decarboxylation of ester to bromine Method S12. Product transformation to 1,2,4-oxadiazoles by cyclization of indazole carboxylic acid esters and amidoximes Methods S13. Product transformation by reduction of carboxylic acid esters to alcohol Fig. S1. STEM-ADF images of nonporous CeO_2_ nanorods. Fig. S2. STEM-ADF images of porous CeO_2_ nanorods. Fig. S3. Representative AFM image of porous CeO_2_ nanorods. Fig. S4. BET and pore-size distribution of various catalysts. Fig. S5. Atomic-resolution STEM-ADF images of Pt_1_-CeO_2_ catalyst. Fig. S6. EDS mapping of Pt_1_-CeO_2_ catalyst. Fig. S7. Detailed XANES simulations and the experimental curve of Pt_1_/CeO_2_. Fig. S8. XRD and XPS data of various catalysts. Fig. S9. UV-Raman, EPR, and TPD data of various catalysts. Fig. S10. STEM-HAADF images of Pt_1_ on nonporous CeO_2_ by ALD. Fig. S11. STEM-HAADF images of Co_1_/graphene, Fe_1_/graphene, and Pt_1_/graphene. Fig. S12. The hydrolysis of ammonia borane by various catalysts. Fig. S13. *E*/*Z* selectivity of the Pt-catalyzed reduction of diazo substrate **2a** from NMR. Fig. S14. Recycling efficiency of Pt_1_-CeO_2_ and Pt_1_-CeO_2_-non for selective *E*-hydrazone synthesis. Fig. S15. STEM-HAADF images of the used Pt_1_/CeO_2_ catalyst. Fig. S16. Pt L_3_-edge XANES and EXAFS spectra of the fresh and spent Pt_1_/CeO_2_ catalysts. Fig. S17. Thermodynamic stability of hydrazone-free molecule by DFT. Fig. S18. Comparison of **2a** adsorption on Pt and O vacancy of CeO_2_. Fig. S19. The *E*/*Z* transformation under standard conditions. Fig. S20. H adsorption on a Pt single atom on CeO_2_ nanorods. Fig. S21. The optimized adsorption configuration of the *E*- and *Z*-isomers on Pt_1_/CeO_2_ catalyst. Fig. S22. Adsorption energies for the *E* and *Z* isomers on Pt_1_/CeO_2_ catalyst. Fig. S23. Large-scale synthesis for selective *E*-hydrazone synthesis. Fig. S24. Total synthesis of lonidamine in four steps. Fig. S25. Gram-scale synthesis of lonidamine. Fig. S26. Retrosynthesis of pharmaceuticals using the *E*-hydrazone strategy. Table S1. Results of DFT calculations. Table S2. Catalyst screening for selective *E*-hydrazone synthesis. Table S3. Solvent screening for selective *E*-hydrazone synthesis. Table S4. Borane screening for selective *E*-hydrazone synthesis. Table S5. Results of the EXAFS fitting on PtO_2_ and Pt_1_/CeO_2_. Table S6. Representative methods for the total-synthesis of lonidamine. References (*45*–*53*)
