# Supplementary material for: Expedient synthesis of E-hydrazone esters and 1H-indazole scaffolds through heterogeneous single-atom platinum catalysis
Source: Sci Adv. 2019 Dec 6;5(12):eaay1537. doi: 10.1126/sciadv.aay1537 (PMC6897547; doi:10.1126/sciadv.aay1537)
Supplement: http://advances.sciencemag.org/cgi/content/full/5/12/eaay1537/DC1 [file supp_5_12_eaay1537__index.html]

Science Advances | Science AdvancesAAASSearchScience AdvancesMenu

## Supplementary Materials

**This PDF file includes:**

- Note S1. Expanded discussion on SACs catalyzed organic reactions
- Note S2. Expanded discussion on SACs catalyzed hydrogenation
- Note S3. Transformation of the ester moiety in the product to generate more complex molecules
- Method S1. Computational details
- Method S2. Synthesis of Co1/graphene and Fe1/graphene
- Method S3. Synthesis of Pt1/graphene
- Method S4. Synthesis of *E*-hydrazone esters **3o**
- Method S5. Total synthesis of lonidamine and F-containing lonidamine **4**
- Method S6. The synthesis of key intermediate **6** for gamendazole
- Method S7. The construction of tricyclic pyridazino1,2-*a*indazolium ring frameworks **8** and **9**
- Method S8. The synthesis of 15N-labeled lonidamine and adjudin
- Method S9. The synthesis of 15N-labeled granisetron
- Method S10. Product transformation by decarboxylation of ester to hydrogenation
- Method S11. Product transformation by decarboxylation of ester to bromine
- Method S12. Product transformation to 1,2,4-oxadiazoles by cyclization of indazole carboxylic acid esters and amidoximes
- Methods S13. Product transformation by reduction of carboxylic acid esters to alcohol
- Fig. S1. STEM-ADF images of nonporous CeO2 nanorods.
- Fig. S2. STEM-ADF images of porous CeO2 nanorods.
- Fig. S3. Representative AFM image of porous CeO2 nanorods.
- Fig. S4. BET and pore-size distribution of various catalysts.
- Fig. S5. Atomic-resolution STEM-ADF images of Pt1-CeO2 catalyst.
- Fig. S6. EDS mapping of Pt1-CeO2 catalyst.
- Fig. S7. Detailed XANES simulations and the experimental curve of Pt1/CeO2.
- Fig. S8. XRD and XPS data of various catalysts.
- Fig. S9. UV-Raman, EPR, and TPD data of various catalysts.
- Fig. S10. STEM-HAADF images of Pt1 on nonporous CeO2 by ALD.
- Fig. S11. STEM-HAADF images of Co1/graphene, Fe1/graphene, and Pt1/graphene.
- Fig. S12. The hydrolysis of ammonia borane by various catalysts.
- Fig. S13. *E*/*Z* selectivity of the Pt-catalyzed reduction of diazo substrate **2a** from NMR.
- Fig. S14. Recycling efficiency of Pt1-CeO2 and Pt1-CeO2-non for selective *E*-hydrazone synthesis.
- Fig. S15. STEM-HAADF images of the used Pt1/CeO2 catalyst.
- Fig. S16. Pt L3-edge XANES and EXAFS spectra of the fresh and spent Pt1/CeO2 catalysts.
- Fig. S17. Thermodynamic stability of hydrazone-free molecule by DFT.
- Fig. S18. Comparison of **2a** adsorption on Pt and O vacancy of CeO2.
- Fig. S19. The *E*/*Z* transformation under standard conditions.
- Fig. S20. H adsorption on a Pt single atom on CeO2 nanorods.
- Fig. S21. The optimized adsorption configuration of the *E*- and *Z*-isomers on Pt1/CeO2 catalyst.
- Fig. S22. Adsorption energies for the *E* and *Z* isomers on Pt1/CeO2 catalyst.
- Fig. S23. Large-scale synthesis for selective *E*-hydrazone synthesis.
- Fig. S24. Total synthesis of lonidamine in four steps.
- Fig. S25. Gram-scale synthesis of lonidamine.
- Fig. S26. Retrosynthesis of pharmaceuticals using the *E*-hydrazone strategy.
- Table S1. Results of DFT calculations.
- Table S2. Catalyst screening for selective *E*-hydrazone synthesis.
- Table S3. Solvent screening for selective *E*-hydrazone synthesis.
- Table S4. Borane screening for selective *E*-hydrazone synthesis.
- Table S5. Results of the EXAFS fitting on PtO2 and Pt1/CeO2.
- Table S6. Representative methods for the total-synthesis of lonidamine.
- References (*45*–*53*)

Download PDF

**Files in this Data Supplement:**

- Adobe PDF - aay1537\_SM.pdf
