# Supplementary material for: Expedient synthesis of E-hydrazone esters and 1H-indazole scaffolds through heterogeneous single-atom platinum catalysis
Source: Sci Adv. 2019 Dec 6;5(12):eaay1537. doi: 10.1126/sciadv.aay1537 (PMC6897547; doi:10.1126/sciadv.aay1537)
Supplement: Download PDF [file aay1537_SM.pdf]

Science Advances
AAAS

advances.sciencemag.org/cgi/content/full/5/12/eaay1537/DC1

Supplementary Materials for

Expedient synthesis of *E*-hydrazone esters and 1*H*-indazole scaffolds through heterogeneous single-atom platinum catalysis

Cuibo Liu, Zhongxin Chen, Huan Yan, Shibo Xi, Kah Meng Yam, Jiajian Gao, Yonghua Du, Jing Li, Xiaoxu Zhao, Keyu Xie, Haisen Xu, Xing Li, Kai Leng, Stephen J. Pennycook, Bin Liu, Chun Zhang, Ming Joo Koh*, Kian Ping Loh*

*Corresponding author. Email: chmkmj@nus.edu.sg (M.J.K.); chmlohkp@nus.edu.sg (K.P.L.)

Published 6 December 2019, *Sci. Adv.* **5**, eaay1537 (2019)
DOI: 10.1126/sciadv.aay1537

This PDF file includes:

Note S1. Expanded discussion on SACs catalyzed organic reactions
Note S2. Expanded discussion on SACs catalyzed hydrogenation
Note S3. Transformation of the ester moiety in the product to generate more complex molecules
Method S1. Computational details
Method S2. Synthesis of Co_1/graphene and Fe_1/graphene
Method S3. Synthesis of Pt_1/graphene
Method S4. Synthesis of *E*-hydrazone esters **3o**
Method S5. Total synthesis of lonidamine and F-containing lonidamine **4**
Method S6. The synthesis of key intermediate **6** for gamendazole
Method S7. The construction of tricyclic pyridazino[1,2-*a*]indazolium ring frameworks **8** and **9**
Method S8. The synthesis of ^{15}N-labeled lonidamine and adjudin
Method S9. The synthesis of ^{15}N-labeled granisetron
Method S10. Product transformation by decarboxylation of ester to hydrogenation
Method S11. Product transformation by decarboxylation of ester to bromine
Method S12. Product transformation to 1,2,4-oxadiazoles by cyclization of indazole carboxylic acid esters and amidoximes
Methods S13. Product transformation by reduction of carboxylic acid esters to alcohol
Fig. S1. STEM-ADF images of nonporous CeO_2 nanorods.
Fig. S2. STEM-ADF images of porous CeO_2 nanorods.
Fig. S3. Representative AFM image of porous CeO_2 nanorods.
Fig. S4. BET and pore-size distribution of various catalysts.
Fig. S5. Atomic-resolution STEM-ADF images of Pt_1-CeO_2 catalyst.
Fig. S6. EDS mapping of Pt_1-CeO_2 catalyst.
Fig. S7. Detailed XANES simulations and the experimental curve of Pt_1/CeO_2.
Fig. S8. XRD and XPS data of various catalysts.
Fig. S9. UV-Raman, EPR, and TPD data of various catalysts.
Fig. S10. STEM-HAADF images of Pt_1 on nonporous CeO_2 by ALD.

Fig. S11. STEM-HAADF images of Co_1/graphene, Fe_1/graphene, and Pt_1/graphene.
Fig. S12. The hydrolysis of ammonia borane by various catalysts.
Fig. S13. *E*/*Z* selectivity of the Pt-catalyzed reduction of diazo substrate **2a** from NMR.
Fig. S14. Recycling efficiency of Pt_1-CeO_2 and Pt_1-CeO_2-non for selective *E*-hydrazone synthesis.
Fig. S15. STEM-HAADF images of the used Pt_1/CeO_2 catalyst.
Fig. S16. Pt L_3-edge XANES and EXAFS spectra of the fresh and spent Pt_1/CeO_2 catalysts.
Fig. S17. Thermodynamic stability of hydrazone-free molecule by DFT.
Fig. S18. Comparison of **2a** adsorption on Pt and O vacancy of CeO_2.
Fig. S19. The *E*/*Z* transformation under standard conditions.
Fig. S20. H adsorption on a Pt single atom on CeO_2 nanorods.
Fig. S21. The optimized adsorption configuration of the *E*- and *Z*-isomers on Pt_1/CeO_2 catalyst.
Fig. S22. Adsorption energies for the *E* and *Z* isomers on Pt_1/CeO_2 catalyst.
Fig. S23. Large-scale synthesis for selective *E*-hydrazone synthesis.
Fig. S24. Total synthesis of lonidamine in four steps.
Fig. S25. Gram-scale synthesis of lonidamine.
Fig. S26. Retrosynthesis of pharmaceuticals using the *E*-hydrazone strategy.
Table S1. Results of DFT calculations.
Table S2. Catalyst screening for selective *E*-hydrazone synthesis.
Table S3. Solvent screening for selective *E*-hydrazone synthesis.
Table S4. Borane screening for selective *E*-hydrazone synthesis.
Table S5. Results of the EXAFS fitting on PtO_2 and Pt_1/CeO_2.
Table S6. Representative methods for the total-synthesis of lonidamine.
References (*45–53*)

Note S1. Expanded discussion on SACs catalyzed organic reactions

Single atom catalyst (SAC) with enhanced activity, improved selectivity and maximized atom utilization is now becoming a powerful tool in synthetic chemistry, which forms a new frontier in heterogeneous catalysis. During the past decade, a substantial effort has been devoted to the preparation of SACs and many valuable reactions have been explored, such as **hydrogenation** of nitroarenes, quinolines, alkynes, alkenes (*Nat. Commun.* **5,** 5634 (2014); *J. Am. Chem. Soc.* **139,** 9419–9422 (2017); *Angew. Chem. Int. Ed.* **54,** 11265–11269 (2015); *Nat. Commun.* **8,** 16100 (2017); *J. Am. Chem. Soc.* **135**, 12634–12645 (2013)); **oxidation** of carbon monoxide, benzene and the C-H bonds (*Nat. Chem.* **3,** 634–641(2011); *Sci. Adv.* **1,** e1500462 (2015); *J. Am. Chem. Soc.* **139,** 10790–10798 (2017)) and **C-C bond formation** reactions, such as aerobic oxidative cross-coupling of primary and secondary alcohols (*ACS Catal.* **5,** 6563–6572 (2015); *Angew. Chem., Int. Ed.* **128,** 16288–16292 (2016)).

Note S2. Expanded discussion on SACs catalyzed hydrogenation

The hydrogenation of nitro-compounds and unsaturated organic molecules is an important process in the manufacture of agrochemicals, pharmaceuticals and dyes. SACs display remarkable advantages over the homogeneous catalysts and nanoparticles or clusters on the chemo-selectivity and reaction efficiency. Some important achievements on hydrogenation reactions are as follow: *Nat. Common.* **9,** 3197 (2018); *Chem. Sci.* **7,** 5758–5764 (2016) for hydrogenation of nitroarenes; *J. Phys. Chem. C* **119,** 18140–18148 (2015) for hydrogenation of carbonyl compounds; *Nat. Commun.* **6,** 8550 (2015) for hydrogenation of alkenes; *ACS Catal.* **5,** 3717–3725 (2015); *J. Am. Chem. Soc.* **139,** 7294–7301 (2017) for hydrogenation of alkynes.

Note S3. Transformation of the ester moiety in the product to generate more complex molecules

The ester moiety in the product is versatile intermediate, which can be facilely transformed to other functional groups for further generation of more complex molecules (Supplementary Method). For instance, the facile conversion of ester to bromine (**17**) provides good opportunity to create new C-C or C-N bond via coupling reactions. 1,2,4-Oxadiazoles skeletons (**19**) frequently seen in many drugs with biological importance can be easily prepared by the reaction of carboxylic acid ester with amidoximes in the presence of base.

Method S1. Computational details

The first-principles calculations were performed with spin-polarized density functional theory (DFT) as implemented in Vienna *ab-initio* Simulation Package (VASP) (*45-46*). The generalized gradient approximation (GGA) in the Perdew-Burke-Ernzerhof (PBE) format (*47*), the projector-augmented wave (PAW) method (*48*) and a plane-wave basis with the kinetic cut-off energy of 400 eV is used are employed in all calculations. To describe the highly localized Ce 4*f* orbitals better, the Dudarev approach was adopted (*49*), which the value of the parameter (U - J) was set to be 4.5 eV only for the Ce 4*f* states, following previous work (*50*).

Modelling of CeO_2 Slab. The CeO_2 (111) surface was modelled with nine atomic layers containing a 2 × 2 unit cell. During geometry optimization, only the atoms in the top six CeO_2 atomic layers and the adsorbates were allowed to relax until the force acting each atom was less than 0.02 eV $Å^{-1}$. To avoid the artificial interactions along the Z-direction between its periodic images, a vacuum space of at least 20 Å was employed.

Method S2. Synthesis of Co_1/graphene and Fe_1/graphene

200 mg of GO was firstly dispersed in 400 mL of deionized (DI) water under stirring and sonication to achieve a homogeneous dispersion. An aqueous solution of 0.0675 M $CoCl_2$ or $FeCl_3$ was added into the GO solution followed by sonication. The mixture was lyophilized to form a 3D GO foam with uniformly distributed Co or Fe ions and then annealed at 750 °C under Ar (100 sccm) and NH_3 (30 sccm) at room pressure for simultaneous N doping and GO reduction to obtain Co_1 or Fe_1/graphene powders.

Method S3. Synthesis of Pt_1/graphene

The synthesis of Pt_1/graphene was similar to that of Pt_1/CeO_2 by using N-doped graphene as support. The Pt precursor were heated at 100 °C to generate a high enough vapor pressure. The reactor and reactor inlets were held at 150 and 120 °C respectively to avoid any precursor condensation. The timing sequence was 100, 120, 150, and 120 seconds for the $MeCpPtMe_3$ exposure, N_2 purge, O_3 exposure and N_2 purge, respectively.

Method S4. Synthesis of *E*-hydrazone esters 3o

To a 4 mL vial was added 3 mg of Pt_1/CeO_2 catalysts and 2.0 mL of MeOH and sonicated for 15 min at RT. Then, 0.1 mmol of diazo compound **2o** was added and the mixture was stirred at 10 °C for 30 min. After that, 0.06 mmol of ammonia borane were added quickly. The vial was sealed and the reaction mixture was stirred at RT for another 3 h. After reaction, the mixture was centrifuged to remove catalyst and washed with CH_2Cl_2 for three times. The supernatant was vapored under reduced pressure and the residuals was subjected to be separated using thin layer chromatography (TLC) plate. The yield was calculated by dividing the amount of the obtained desired product by the theoretical yield. ***Note***: the all the operations are carried out in a dark room. The TLC plate was neutralized used NEt_3 in advance. And the temperature of water base for rotary evaporator is no more than 25 °C.

Method S5. Total synthesis of lonidamine and F-containing lonidamine 4

The synthesis of **3u** and **3t**: Typically, carboxylic ester (0.2 mmol), TsN_3 (0.24 mmol) and CH_3CN (0.3 mL) were added to a glass vial (4 mL). With stirring, DBU (0.24 mmol) was added dropwise. The reaction mixture stirred at RT for 1 h. Then, 18 mg of Pt_1/CeO_2 catalysts and 2.0 mL of MeOH were added directedly and sonicated for 15 min. After that, 1.8 mmol of AB was

added. The vial was quickly sealed and the reaction mixture was stirred at RT for another 3 h. After reaction, the mixture was centrifuged to remove catalyst and washed with CH_2Cl_2 for three times. The supernatant was vapored under reduced pressure and the residuals was subjected to be separated using TLC plate to obtain the pure products **3u** and **3v**. **3u**: 39.4 mg, 73% yield; **3v**: 33.2 mg, 68% yield.

The synthesis of *Lonidamine* and F-containing *Lonidamine* **4**: To an over-dried 10 mL Schlenk tube was added *E*-*α*-hydrazone esters (0.2 mmol), CuI (0.02 mmol), Cs_2CO_3 (0.5 mmol). After vacuum for 15 min, 2,4-dichloro-1-(chloromethyl)benzene (0.26 mmol) and anhydrous DMSO (0.2 mL) were added via syringe. The reaction mixture was immersed in 40 ^oC oil bath and stirred for 2 h. After the reaction finished by TLC, the reaction mixture was centrifuged and washed with CH_2Cl_2 (3 × 5 mL). The combined organic phase was vapored. To the residues was added 1.2 mL of EtOH and 1.2 mL of 1 N NaOH. The reaction mixture was sonicated for 15 min and moved to a 70 ^oC oil bath stirring for 2 h. After cooling to room temperature, the reaction mixture was acidified to PH = 1 using 1 N HCl and stirred at 80 ^oC for 5 h. Then cooled down the reaction mixture, and filtered. The crude products was separated using TLC plate to obtain the pure products *Lonidamine* and **4**. *Lonidamine*: 36.5 mg, 57% yield. **4**: 30.1 mg, 47% yield.

Method S6. The synthesis of key intermediate 6 for gamendazole

The synthesis of methyl 2-(2-bromo-4-(trifluoromethyl)phenyl)acetate **2w** was according to a reported literature (*51*): Conc. sulfuric acid (100 mmol, 10 equiv.) was added dropwise to a stirred solution of **5** (10 mmol) in MeOH (15 mL) at 0 °C. The mixture was then refluxed until completion of the reaction. After cooling, water (80 mL) was added and the mixture was extracted with CH_2Cl_2 (3 × 20 mL). The combined extracts were washed with brine (2 × 50 mL), dried ($MgSO_4$) and the solvent removed under reduced pressure to afford the methyl ester that was used without further purification. **2w**: 2.81 g, 95% yield.

One-pot synthesis of *E*-*α*-hydrazone ester **3w**: the procedure was similar with the synthesis of **3u** and **3v** and 0.2 mmol of **2w** was used as the starting material. **3w**: 45.4 mg, 70% yield.

The synthesis of key intermediate **6**: To an over-dried 10 mL Schlenk tube was added *E*-*α*-hydrazone esters **3w** (0.2 mmol), CuI (0.02 mmol), Cs_2CO_3 (0.5 mmol). After vacuum for 15 min, 2,4-dichloro-1-(chloromethyl)benzene (0.26 mmol) and anhydrous DMSO (0.2 mL) were added via syringe. The reaction mixture was immersed in 40 ^oC oil bath and stirred for 2 h. After the reaction finished by TLC, the reaction mixture was centrifuged and washed with CH_2Cl_2 (3 × 5 mL). The combined organic phase was vapored. The crude products was separated using TLC plate to obtain the pure **6**. 73.2 mg, 91% yield.

Method S7. The construction of tricyclic pyridazino[1,2-*a*]indazolium ring frameworks 8 and 9

The synthesis of **3x**: 6 mg of Pt_1/CeO_2 catalysts were dispersed in 2.0 mL of MeOH and sonicated for 15 min. Then, 0.2 mmol of diazo compound **2x** and 0.6 mmol of ammonia borane were added sequentially. The vial (4 mL) was sealed quickly and the reaction mixture was stirred at RT for 40 min. After reaction, the mixture was centrifuged to remove catalyst and washed with CH_2Cl_2 for three times. The supernatant was vapored under reduced pressure and the residuals was subjected to be separated using TLC plate. **3x**: 41.4 mg, 81% yield.

The synthesis of **7**: To an over-dried 10 mL Schlenk tube was added **3x** (0.2 mmol), CuI (0.02 mmol), Cs_2CO_3 (0.5 mmol). After vacuum for 15 min, 1,4-Dibromobutane (2.0 mmol) and anhydrous DMSO (0.5 mL) were added via syringe. The reaction mixture was immersed in 40 ^oC oil bath and stirred for 2 h. After the reaction finished by TLC, the reaction mixture was

centrifuged and washed with CH_2Cl_2 (3 × 5 mL). The combined organic phase was vapored. The crude products was separated using TLC plate to obtain the **7**, 46.5 mg, 75% yield.
The synthesis of **8**: **7** (0.1 mmol) was dissolved in methanol (4.0 mL) under a rapid stream of Ar and the solution was sealed in a thick-walled pressure flask. The yellow solution was heated at 85 °C for 24 h, at which time the flask was cooled to 0 °C and the solvent was evaporated on a rotary evaporator to yield **8** as a yellow-white solid, 30.7 mg, 99% yield.
The synthesis of **9**: **7** (0.1 mmol) (0.1 mmol) was dissolved in a mixture of 10% H_2O in acetone (v/v, 5.0 mL) and the solution was sealed in a thick-walled pressure flask. The yellow solution was heated at 80 °C for 24 h, at which time the flask was cooled to ambient temperature and the solvent was evaporated to yield **9** as a yellow-white solid, 22.4 mg, 89% yield.

Method S8. The synthesis of ^{15}N-labeled lonidamine and adjudin
The synthesis of **Ts^{15}N$_3$** was according to the reported literature (*52*): To a flask of 100 mL, a solution of sodium azide (1.00 g, 15.2 mmol) in water (4 mL) and acetone (4 mL) was rapidly added a solution of *p*-toluenesulfonyl chloride (2.63 g, 13.8 mmol) in acetone (4 mL). The mixture warmed slightly and two phases were formed. After stirring at room temperature for 4 h, acetone was evaporated under reduced pressure (bath temperature 35 ºC), the residue was extracted with CH_2Cl_2 (3 × 6 mL), washed with water (2 × 6 mL), dried over $MgSO_4$, and concentrated under reduced pressure (bath temperature 35 ºC) to give the compound as a colorless oil. **Ts^{15}N$_3$**: 2.68 g, 98% yield.
The synthesis of ^{15}N-labelled diazo compound **2u'** was according to the reported literature (*52*): To a mixture of **2u** (10 mmol) and **Ts^{15}N$_3$** (2.37 g, 12 mmol) in anhydrous MeCN (15 mL), 1,8-diazabicyclo[5.4.0]undec-7-ene (DBU) (1.83 g, 12 mmol) was added. The reaction mixture was stirred at room temperature for overnight. Upon complete consumption of the starting materials, the reaction mixture was quenched with saturated aqueous solution of NH_4Cl (5 mL), extracted with CH_2Cl_2 (3 × 30 mL), washed with brine (3 × 10 mL), dried over $MgSO_4$, and concentrated under reduced pressure to give the product. The residue was purified by flash chromatography to afford the *α*-diazoester **2u'**, 2.50 g, 93% yield.
The synthesis of ^{15}N-labelled *E*-*α*-hydrazone ester **3u'**: 30 mg of Pt_1/CeO_2 catalysts were dispersed in 20 mL of MeOH and sonicated for 15 min. Then, 1.0 mmol of diazo compound and 3.0 mmol of AB were added sequentially. The vial (4 mL) was sealed quickly and the reaction mixture was stirred at RT for 6 h. After reaction, the mixture was centrifuged to remove catalyst and washed with CH_2Cl_2 (3 × 10 mL). The supernatant was vapored under reduced pressure and the residuals was subjected to be separated using TLC plate to obtain the pure products **3u'**, 224.9 mg, 83% yield.
The synthesis of **10**: To an over-dried 10 mL Schlenk tube was added **3u'** (0.2 mmol), CuI (0.02 mmol), Cs_2CO_3 (0.5 mmol). After vacuum for 15 min, 2,4-dichloro-1-(chloromethyl)benzene (2.0 mmol) and anhydrous DMSO (0.5 mL) were added via syringe. The reaction mixture was immersed in 40 ºC oil bath and stirred for 2 h. After the reaction finished by TLC, the reaction mixture was centrifuged and washed with CH_2Cl_2 (3 × 5 mL). The combined organic phase was vapored. The crude products was separated using TLC plate to obtain the **10**, 60.7 mg, 87% yield.
The synthesis of ^{15}N-labelled *Lonidamine* was according to the reported literature (*14*): To a 25 mL round bottom flask was added **10** (0.2 mmol), EtOH (1.2 mL) and 1 N NaOH (1.2 mL). The reaction mixture was sonicated for 15 min and then moved to a 70 ºC oil bath stirring for 2 h. After cooling to room temperature, the reaction mixture was acidified to PH = 1 using 1 N HCl and stirred at 80 ºC for another 5 h. Then cooled down the reaction mixture, and filtered. The

crude products was separated using TLC plate to obtain the pure product ^{15}N-labelled *Lonnidamine*: 52.0 mg, 81% yield.

The synthesis of ^{15}N-labelled *Adjudin* was according to the reported literature (*14*): To a solution of Ethanol (2.0 mL) and water (0.5 mL), **10** (0.2 mmol) and hydrazine hydrate (0.64 mL) were added at RT. Reaction mass heated to 80°C and stirred for 3 h. After completion of reaction cooled to RT, stirred for 30 minutes. The solvent was then vapored and the crude product was separated using TLC plate to obtain the pure product ^{15}N-labelled *Adjudin*: 50.9 mg, 76% yield.

Method S9. The synthesis of ^{15}N-labeled granisetron

The synthesis of **11**: To an over-dried 10 mL Schlenk tube was added **3u'** (0.2 mmol), CuI (0.02 mmol), Cs_2CO_3 (0.5 mmol). After vacuum for 15 min, CH_3I (0.26 mmol (2.0 M in *tert*-butyl methyl ether)) and anhydrous DMSO (0.2 mL) were added via syringe. The reaction mixture was immersed in 40 °C oil bath and stirred for 2 h. After the reaction finished by TLC, the reaction mixture was centrifuged and washed with CH_2Cl_2 (3 × 5 mL). The combined organic phase was vapored. The crude products was separated using TLC plate to obtain the pure **11**. 31.1 mg, 76% yield.

The synthesis of **12**: The procedure was similar with the synthesis of ^{15}N-labelled *Lonnidamine* and 0.2 mmol of **11** was used as the starting material. **12**: 31.9 mg, 90% yield.

The synthesis of ^{15}N-labelled *Granisetron* was according to the reported literature (*53*): Indazole acid **12** (0.2 mmol), 1-Ethyl-3-(3-dimethylaminopropyl)-carbodiimide (EDC) (0.25 mmol) and 1-Hydroxybenzotriazole hydrate (HOBt) (0.2 mmol) were mixed together in a flask and dried under high vacuum for 1 h before dissolving in dry DMF:CH_2Cl_2 (1:4, 5 mL), and then stirring the mixture for 2 h at room temperature. A solution of endo-3-amine-9-methyl-9-azabicyclo 3,3,1 nonane dihydrochloride (0.26 mmol) in dry CH_2Cl_2 (1 mL) was added drop wise to the mixture under N_2 atmosphere and stirred for 12 h at room temperature. The progress of the reaction was monitored by TLC. The solvents were removed in vacuo and the crude product was extracted with CH_2Cl_2 (3 × 10 mL) and washed with sat. $NaHCO_3$ (5 mL). The combined organic phases were dried over Na_2SO_4, filtered and concentrated to give the crude product. The crude product was further purified by flash column chromatography to afford ^{15}N-labelled *Granisetron*, 50.7 mg, 81% yield.

Method S10. Product transformation by decarboxylation of ester to hydrogenation

The synthesis of **13**: The procedure was similar with the synthesis of ^{15}N-labelled **10** and 1.0 mmol of *E*-*α*-hydrazone ester was used as the starting material. **13**: 288.8 mg, 83% yield.

The synthesis of **14**: **13** (0.2 mmol) was dissolved in MeOH (2 mL) and aq. HCl (6N, 12 mmol) was added. The reaction mixture was refluxed for 12 h. After removing the solvent, MeOH (0.6 mL), H_2O (0.6 mL) and conc. HCl (0.6 mL) were added and the mixture was refluxed for further 14 h. The solvent was removed and the crude product was purified by flash column chromatography to give **14**, 37.0 mg, 67% yield.

Method S11. Product transformation by decarboxylation of ester to bromine

The synthesis of **15**: To an over-dried 10 mL Schlenk tube was added **3u** (0.2 mmol), CuI (0.02 mmol), Cs_2CO_3 (0.5 mmol). After vacuum for 15 min, anhydrous DMSO (0.5 mL) were added via syringe. The reaction mixture was immersed in 40 °C oil bath and stirred for 2 h. After the reaction finished by TLC, the reaction mixture was centrifuged and washed with CH_2Cl_2 (3 × 5 mL). The combined organic phase was vapored. The crude products was separated using TLC plate to obtain the **15**, 30.8 mg, 81% yield.

The synthesis of **16**: The procedure was similar with the synthesis of ^{15}N-labelled *Lonidamine* and 0.2 mmol of **15** was used as the starting material. **16**: 27.9 mg, 86% yield.
The synthesis of **17**: To an over-dried 10 mL Schlenk tube was added **16** (0.2 mmol) and NBS (0.2 mmol). After vacuum for 15 min, anhydrous DMF (1.5 mL) were added via syringe. The reaction mixture was stirred at RT for 16 h. After the reaction finished by TLC, the reaction mixture was centrifuged and washed with CH_2Cl_2 (3 × 5 mL). The combined organic phase was vapored. The crude products was separated using TLC plate to obtain **17**, 30.6 mg, 78% yield.

Method S12. Product transformation to 1,2,4-oxadiazoles by cyclization of indazole carboxylic acid esters and amidoximes
The synthesis of **18**: To a 100 mL round bottom flask was acetonitrile (10.0 mmol), hydroxylamine hydrochloride (50.0 mmol), potassium carbonate (3.0 mmol) and methanol (50 mL). The reaction mixture was stirred at 60 °C for 12 h. After that, the mixture was filtered and the solvent was vapored. The crude product was recrystallized using methanol to obtain the pure **18**, 636.4 mg, 86% yield.
The synthesis of **19**: To a mixture of **15** (0.2 mmol) and **18** (0.26 mmol) in toluene (2.5 mL) was added potassium carbonate (0.4 mmol). Reaction mixture was heated to reflux for 12 h and reaction progress was monitored by TLC. After completion, reaction mixture was cooled to RT and filtered. Filtrate was concentrated to get residue and dissolved in ethyl acetate. Resulting mixture was washed with water and brine. The organic phase dried over anhydrous sodium sulfate. The solvent was removed under reduced pressure and the resulting residue was chromatographed on silica gel, to give corresponding **19**, 34.8 mg, 87% yield.

Methods S13. Product transformation by reduction of carboxylic acid esters to alcohol
The synthesis of **20**: To a solution of THF (0.5 mL), **13** (0.2 mol) and sodiumborohydride (12.0 mol), methanol (0.4 mL) was added drop wise at 55-60 °C over a period of 2 minutes and stirred for 30 minutes at 55-60 °C. After completion of reaction, distilled off the solvent to get the crude. Water (0.5 mL) was added and stirred for 30 minutes at RT. pH of the reaction mass was adjusted to 2.0-2.5 with 2N HCl at 20-25 °C, extracted the compound with dichloromethane CH_2Cl_2 (3 × 5.0 mL). Combined organic layers were washed with water (10.0 mL) and dried over anhydrous Na_2SO_4. The organic layer was vapored and the residue was chromatographed on silica gel, to give corresponding **34**, 55.1 mg, 90% yield.

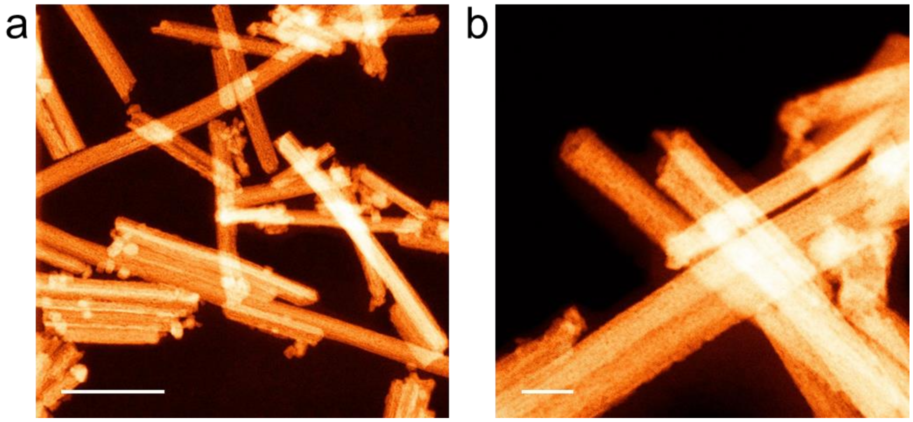

Fig. S1. STEM-ADF images of nonporous CeO_2 nanorods. Scale bar: a. 50; b. 10 nm.

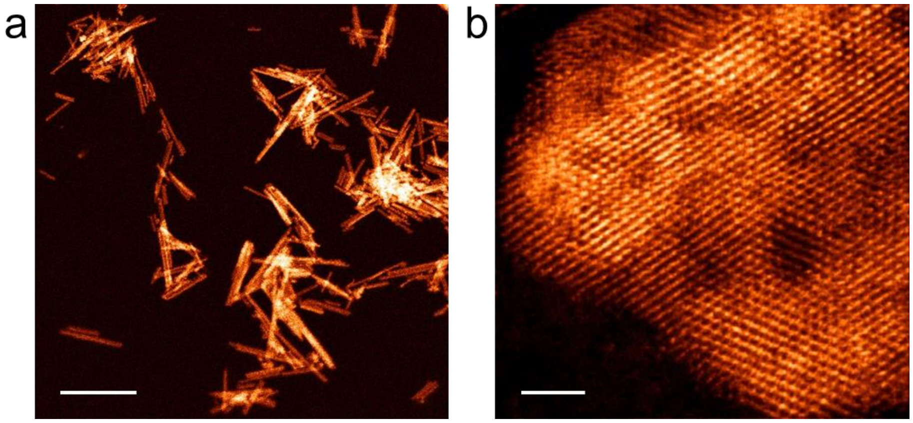

Fig. S2. STEM-ADF images of porous CeO_2 nanorods, revealing the crystalline nanodomains and nanopores on CeO_2. Scale bar: a. 200 nm; b. 2 nm.

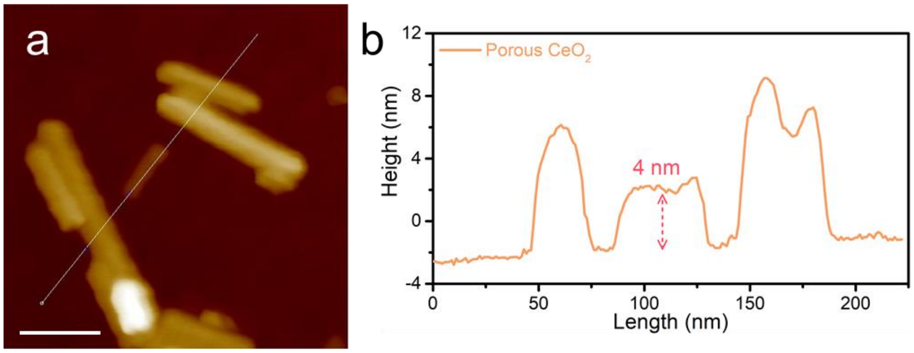

Fig. S3. Representative AFM image of porous CeO_2 nanorods, showing its ultrathin layer structure with a thickness of 4 nm. Scale bar: 50 nm.

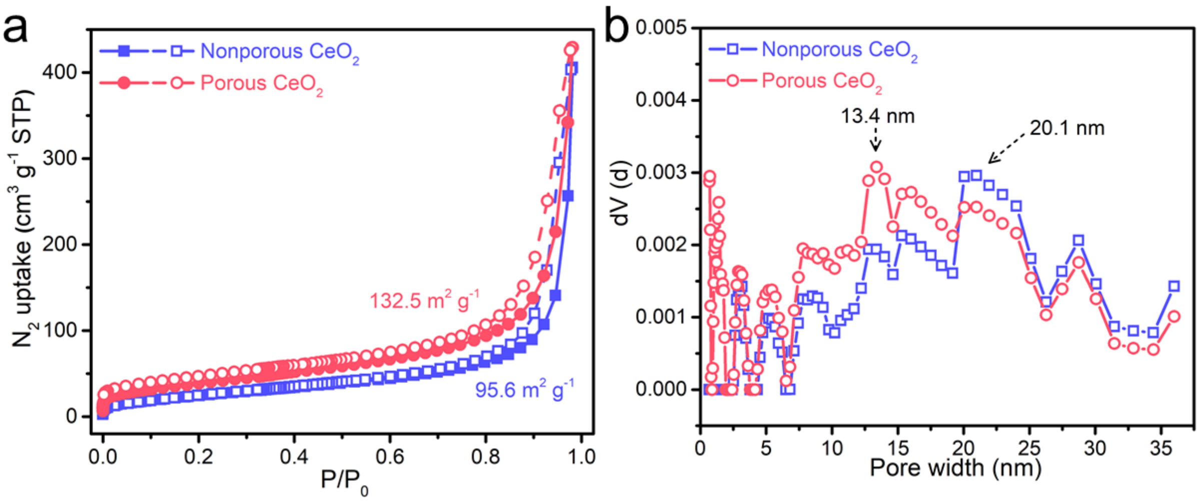

Fig. S4. BET and pore-size distribution of various catalysts. (**a**) N_2 absorption/desorption isotherms and (**b**) the corresponding pore-size distribution of non-porous and porous CeO_2 nanorods.

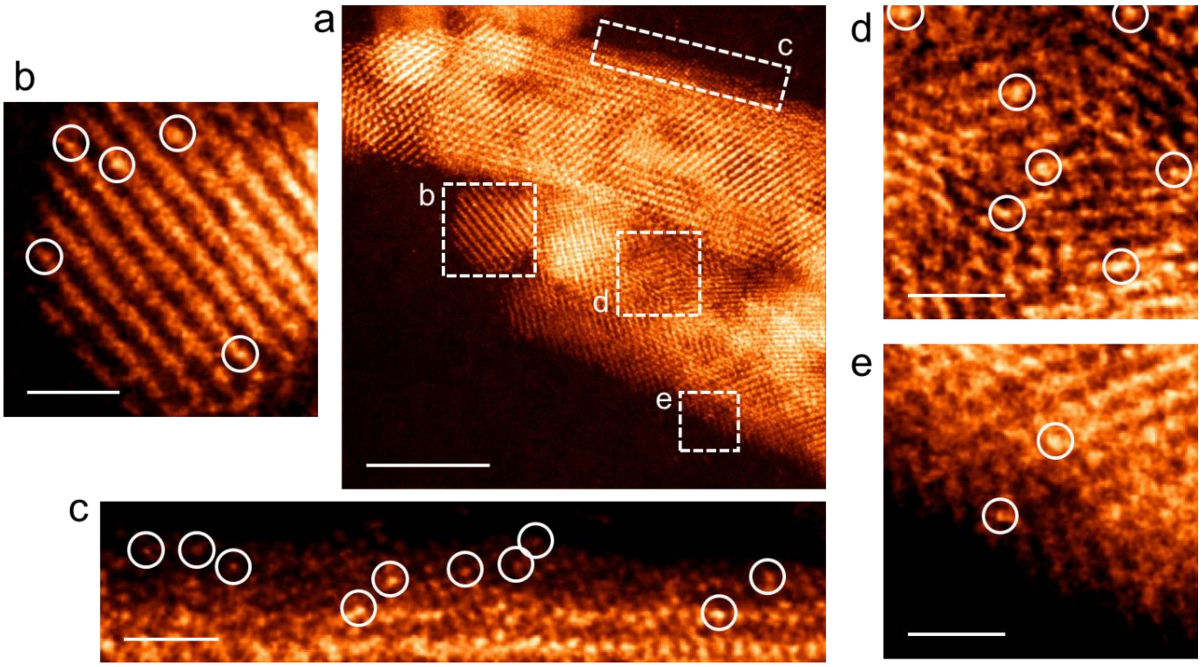

Fig. S5. Atomic-resolution STEM-ADF images of Pt_1-CeO_2 catalyst, revealing the existence of Pt single atoms (highlighted in white cycles) on porous CeO_2. Scale bar: a. 5 nm; b-e. 1 nm.

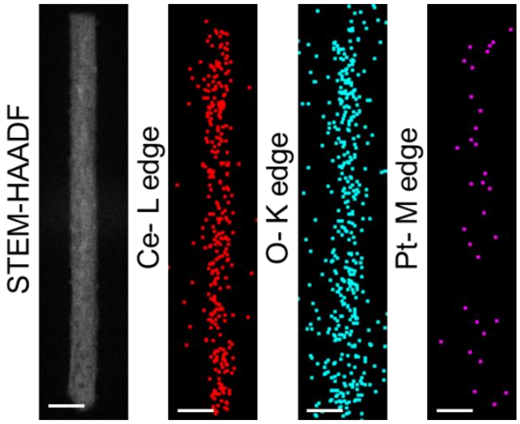

Fig. S6. EDS mapping of Pt_1-CeO_2 catalyst. Scale bar: 10 nm.

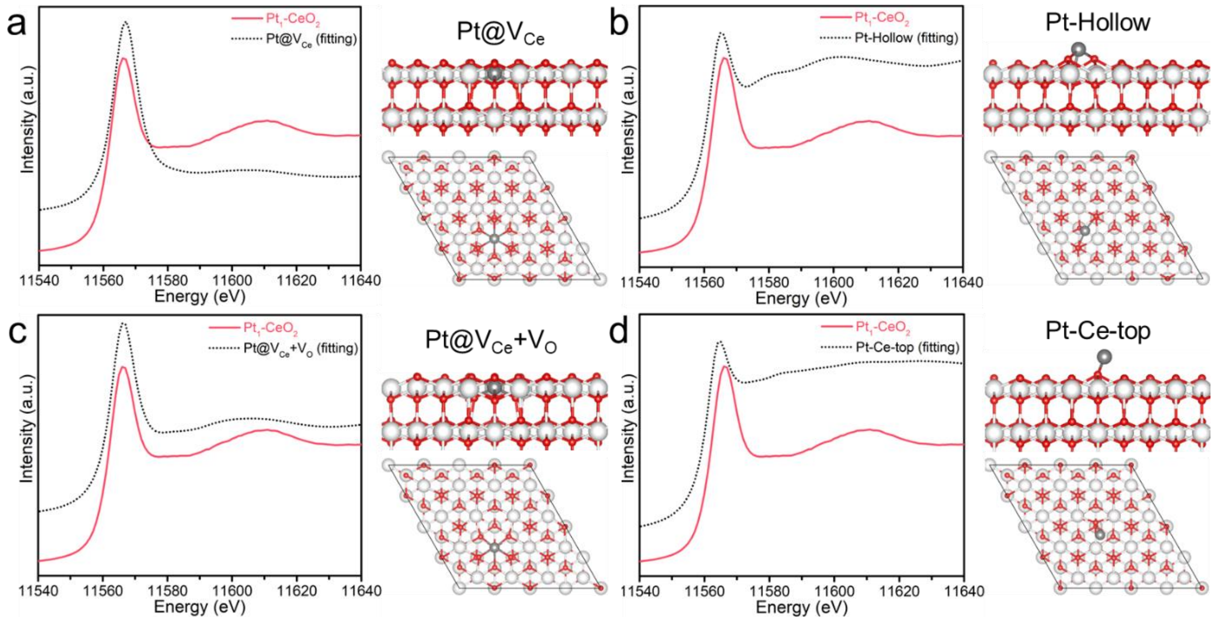

Fig. S7. Detailed XANES simulations and the experimental curve of Pt_1/CeO_2. (**a**) a Ce atom is replaced by Pt single atom (Pt@V_{Ce}); (**b**) Pt single atom sits on top of the hollow sites of CeO_2 (Pt-Hollow); (**c**) a Ce atom is replaced by Pt single atom and one O vacancy next to Pt single atom (Pt@V_{Ce}+V_O); (**d**) Pt single atom sits on top of the Ce atom (Pt-Ce-top). The color scheme used: white-grey for Ce; red for O; grey for Pt. E_{ncut} = 400 eV, Conv. criteria: energy = 10^{-4}, force = 0.02 eV Å^{-1}.

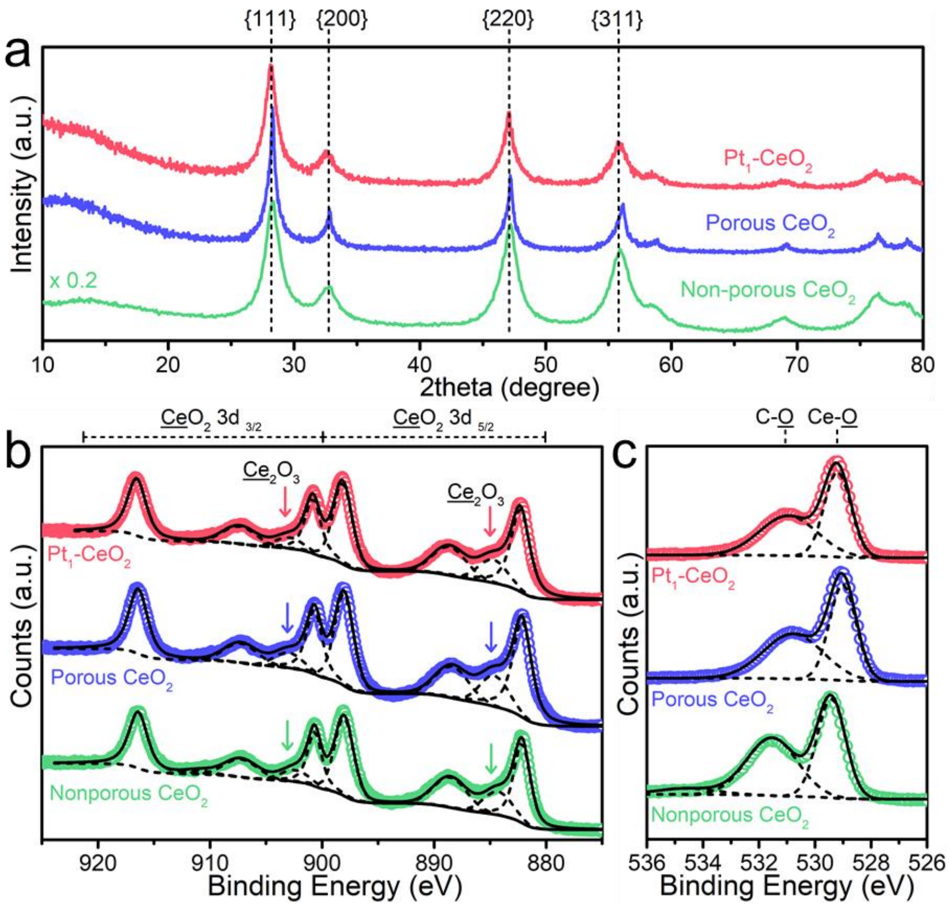

Fig. S8. XRD and XPS data of various catalysts. (**a**) Powder XRD patterns, (**b**) XPS Ce_{3d} and (**c**) O_{1s} spectra of nonporous CeO_2, porous CeO_2 and Pt_1-CeO_2 catalyst.

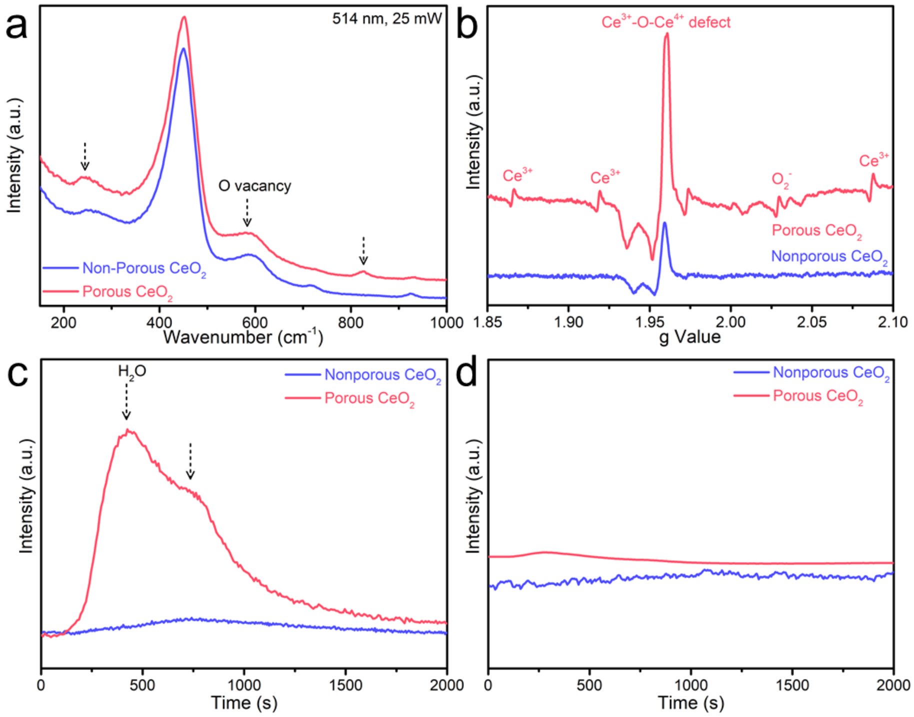

Fig. S9. UV-Raman, EPR, and TPD data of various catalysts. (**a**) UV-Raman and (**b**) EPR spectra of non-porous and porous CeO_2; (**c**) TPD curves of the chemo-adsorption of H_2O and (**d**) CO on porous and non-porous CeO_2.

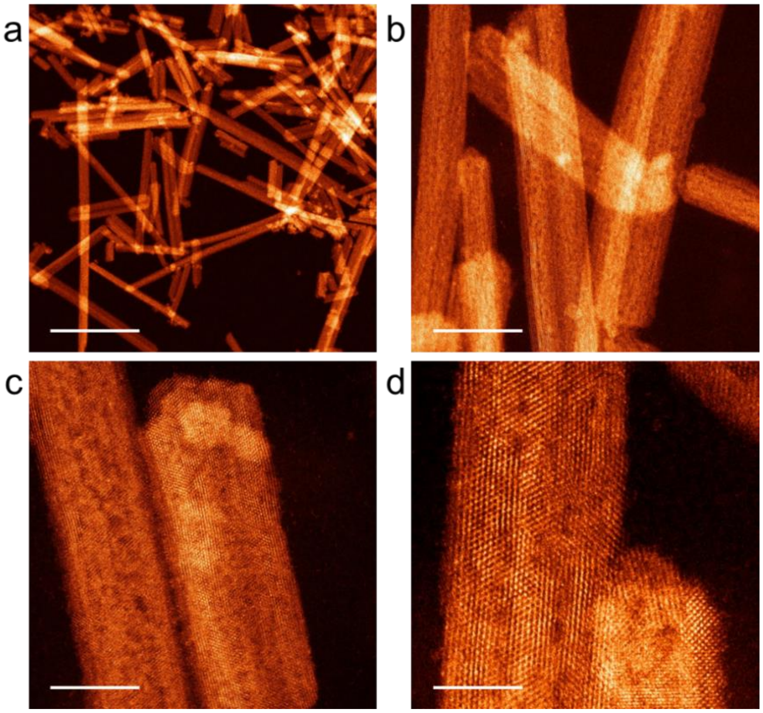

Fig. S10. STEM-HAADF images of Pt_1 on nonporous CeO_2 by ALD. Scale bar: a. 100 nm; b. 20 nm; c. 10 nm; d. 5 nm.

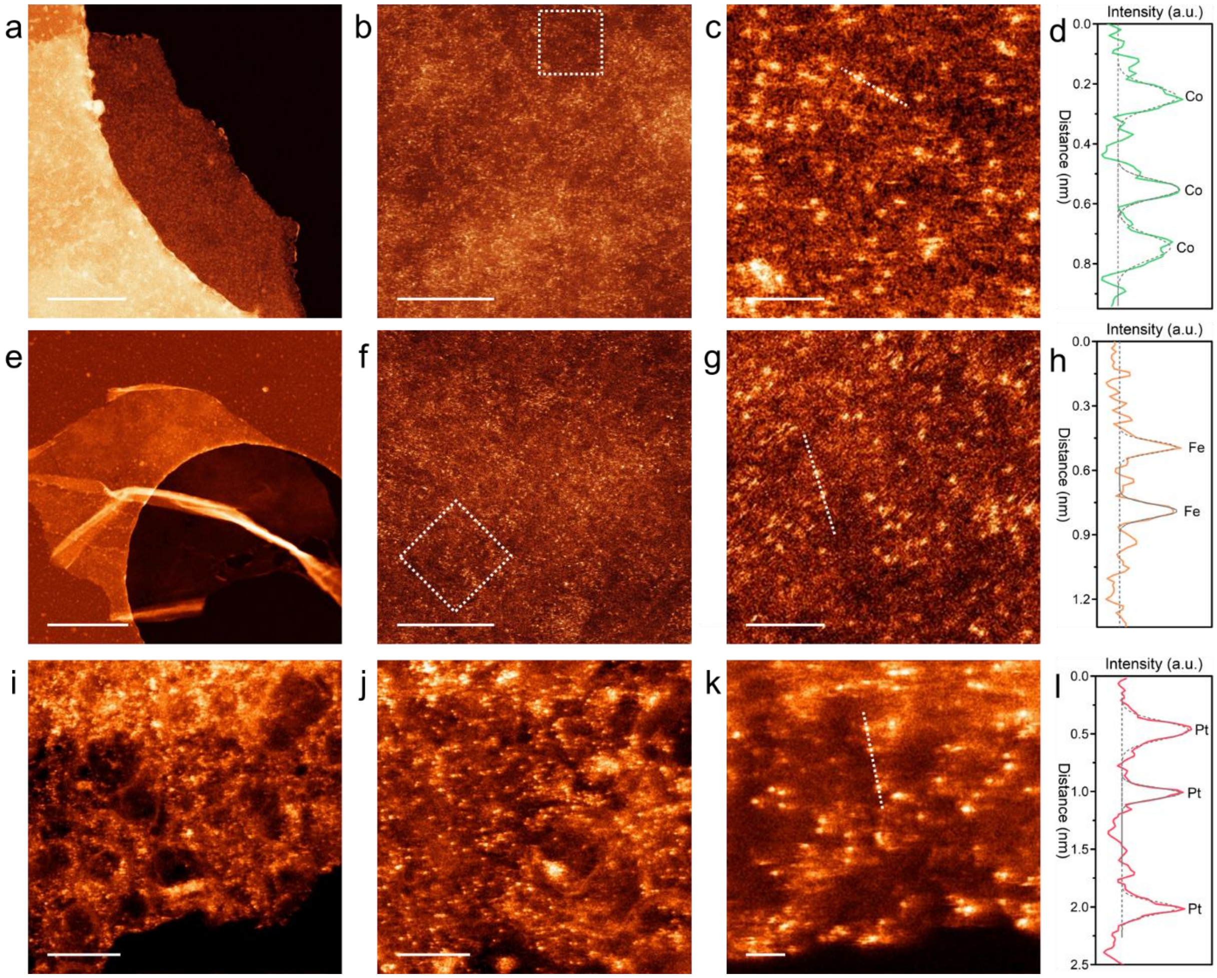

Fig. S11. STEM-HAADF images of Co_1/graphene, Fe_1/graphene, and Pt_1/graphene. (a-d), Fe_1/graphene (**e-h**) and Pt_1/graphene (**i-l**) catalysts. Scale bar: a. 200 nm; b. 5 nm; c. 1 nm; e. 500 nm; f. 5 nm; g. 1 nm; i. 10 nm; j. 5 nm; k. 1 nm.

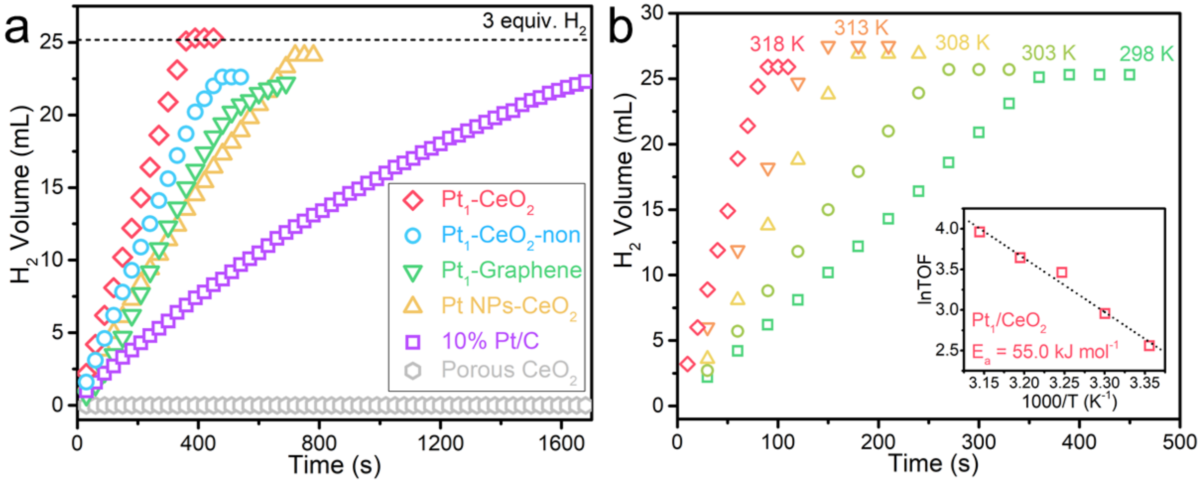

Fig. S12. The hydrolysis of ammonia borane by various catalysts. (**a**) The hydrolysis of ammonia borane (10.2 mg, 0.33 mmol) using benchmark 10% Pt/C, porous CeO_2, Pt_1-graphene, Pt nanoparticles on porous CeO_2, Pt_1-CeO_2-non and Pt_1-CeO_2 catalysts at 298 K; (**b**) Temperature dependence on the hydrolysis of ammonia borane using Pt_1-CeO_2 from 298 K to 318 K. Inset of (b): the Arrhenius plot shows an apparent activation energy of 55.0 kJ mol^{-1}.

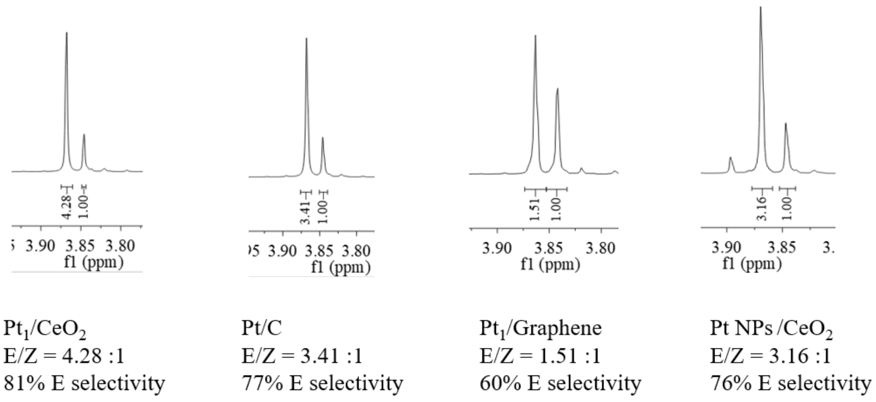

Fig. S13. *E*/*Z* selectivity of the Pt-catalyzed reduction of diazo substrate 2a from NMR.

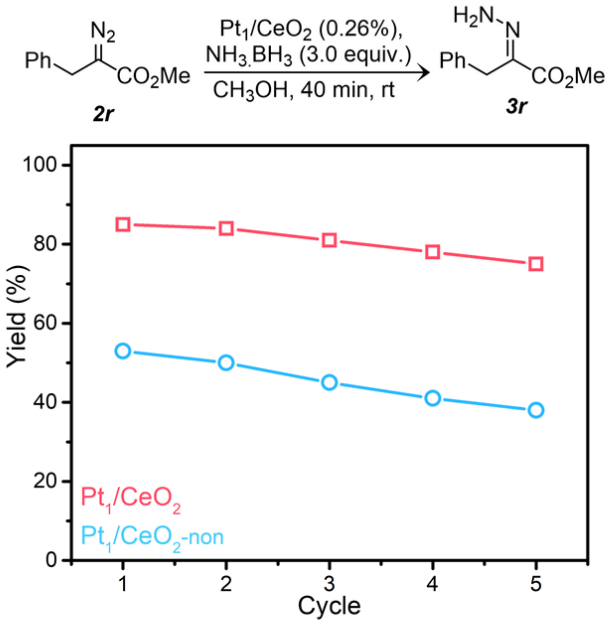

Fig. S14. Recycling efficiency of Pt_1-CeO_2 and Pt_1-CeO_2-non for selective *E*-hydrazone synthesis. Each run was conducted with 0.1 mmol of diazo compound (***2r***) under the optimized condition.

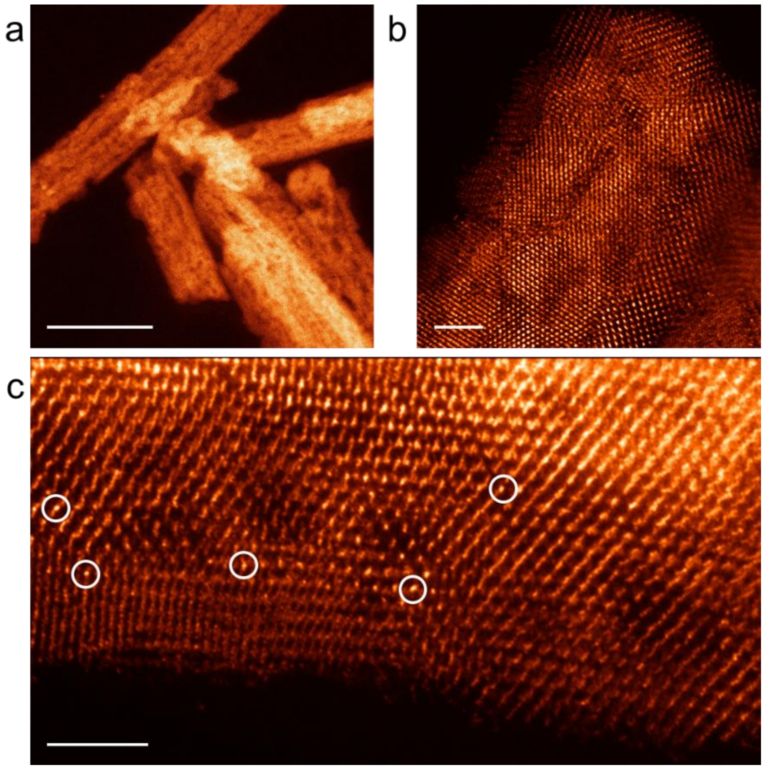

Fig. S15. STEM-HAADF images of the used Pt_1/CeO_2 catalyst. Scale bar: a. 20 nm; b, c. 2 nm.

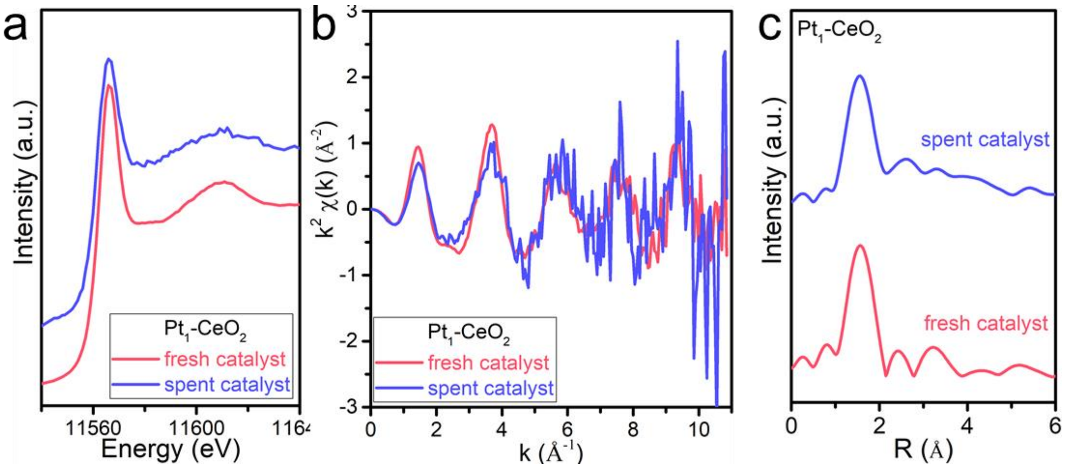

Fig. S16. Pt L_3-edge XANES and EXAFS spectra of the fresh and spent Pt_1/CeO_2 catalysts. Good agreement in the *k*-space between the fresh and spent catalyst suggests its nearly identical structure, despite of a lower resolution in the spent catalyst due to the limited beamtime.

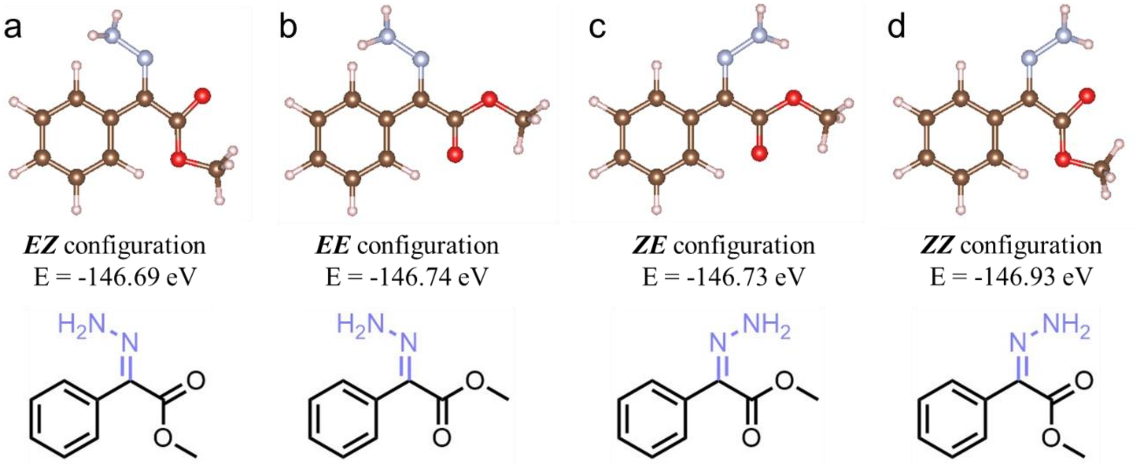

Fig. S17. Thermodynamic stability of hydrazone-free molecule by DFT. E_{ncut} = 400 eV, Conv. criteria: energy = 10^{-4}, force = 0.02 eV $Å^{-1}$.

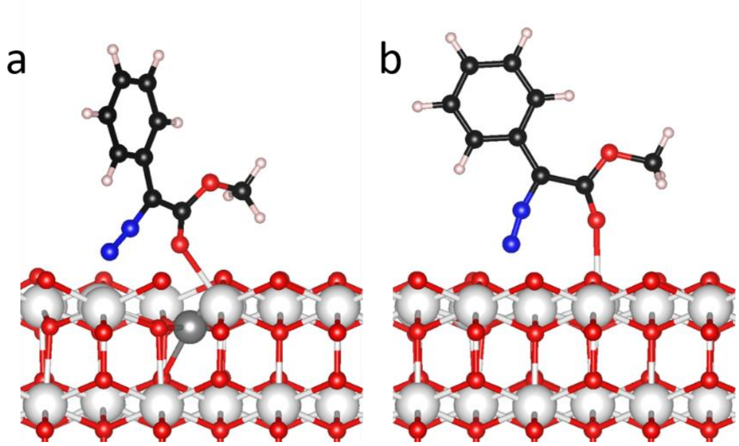

Fig. S18. Comparison of 2a adsorption on Pt and O vacancy of CeO_2. (**a**) and O vacancy of CeO_2 (**b**).

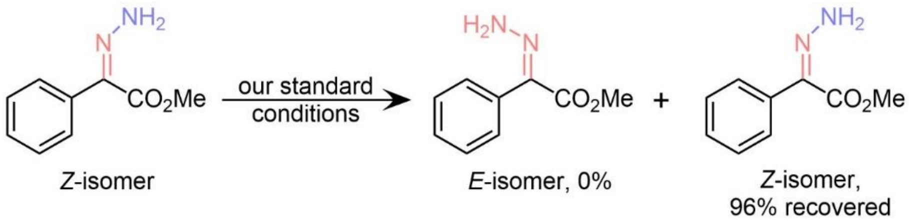

Fig. S19. The *E*/*Z* transformation under standard conditions. The *Z*-isomer cannot transform into the *E*-isomer even after prolonging reaction period.

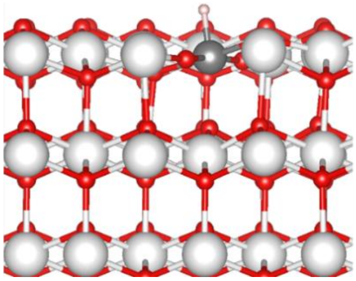

Fig. S20. H adsorption on a Pt single atom on CeO_2 nanorods.

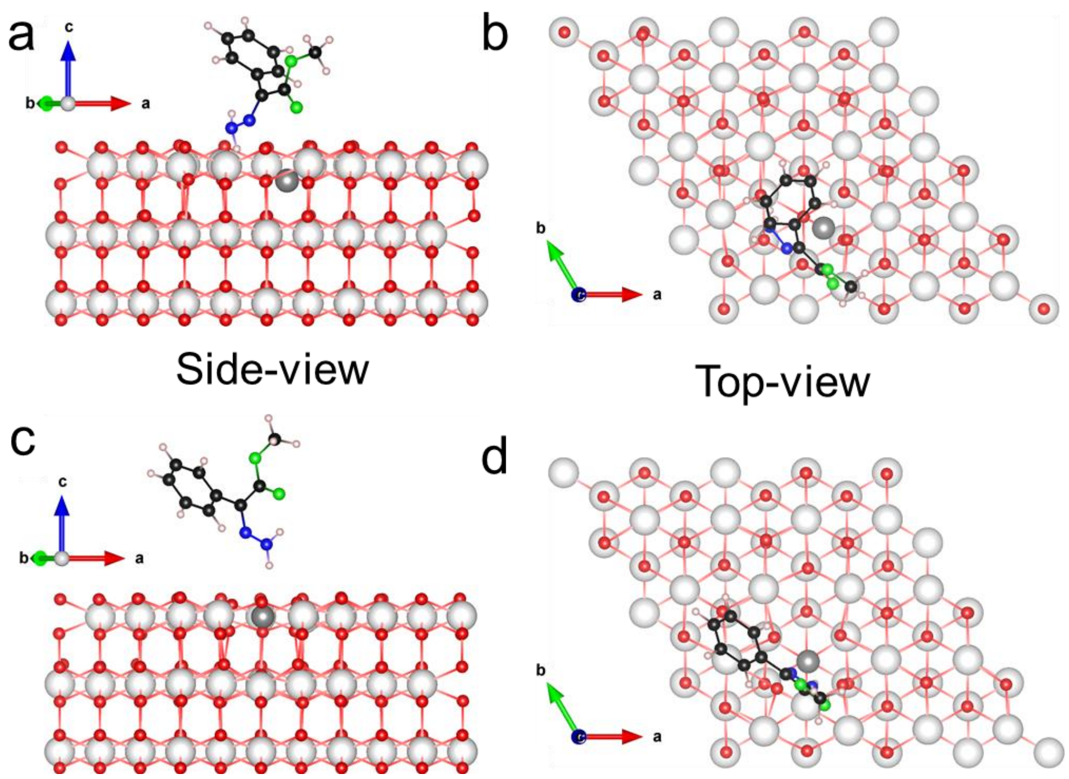

Fig. S21. The optimized adsorption configuration of the *E*- and *Z*-isomers on Pt_1/CeO_2 catalyst. (**a**, **b**) *E*-isomer and (**c**, **d**) *Z*-isomer on the catalyst. The color scheme used: black for C; pink for H; blue for N; green for O belonging the molecule; white-grey for Ce; red for O belonging to doped CeO_2; grey for Pt. E_{ncut} = 400 eV, Conv. criteria: energy = 10^{-4}, force = 0.02 eV $Å^{-1}$.

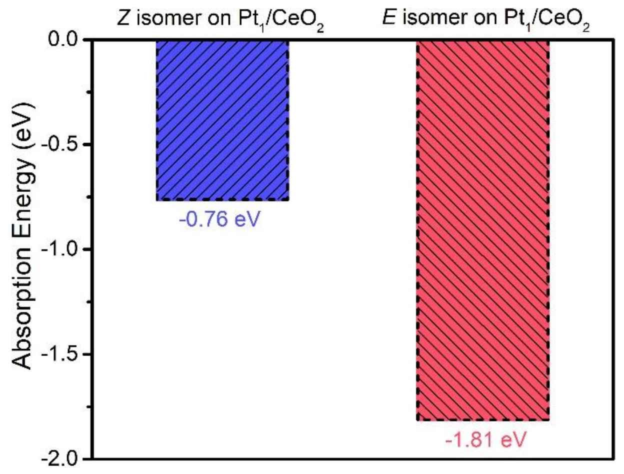

Fig. S22. Adsorption energies for the *E* and *Z* isomers on Pt_1/CeO_2 catalyst. *Z* and *E* isomers represent the *ZZ* and *EZ* configurations in Supplementary Fig. 15 (a) and (d), respectively.

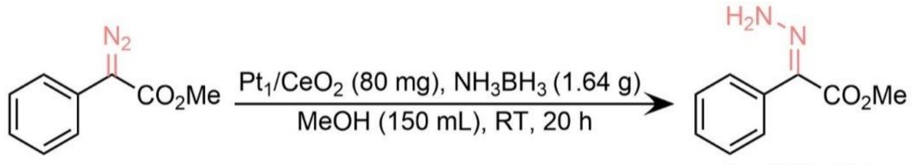

2a

3a, 80%, 1.7 g

Fig. S23. Large-scale synthesis for selective *E*-hydrazone synthesis.

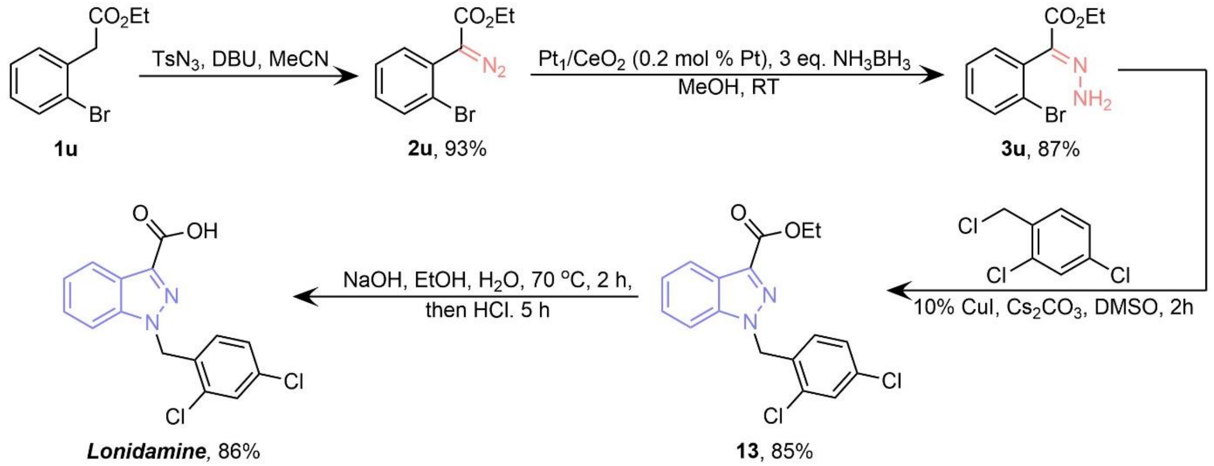

Fig. S24. Total synthesis of lonidamine in four steps using Pt_1 SACs-catalysed, selective *E*-hydrazone conversion with an overall yield of 58%.

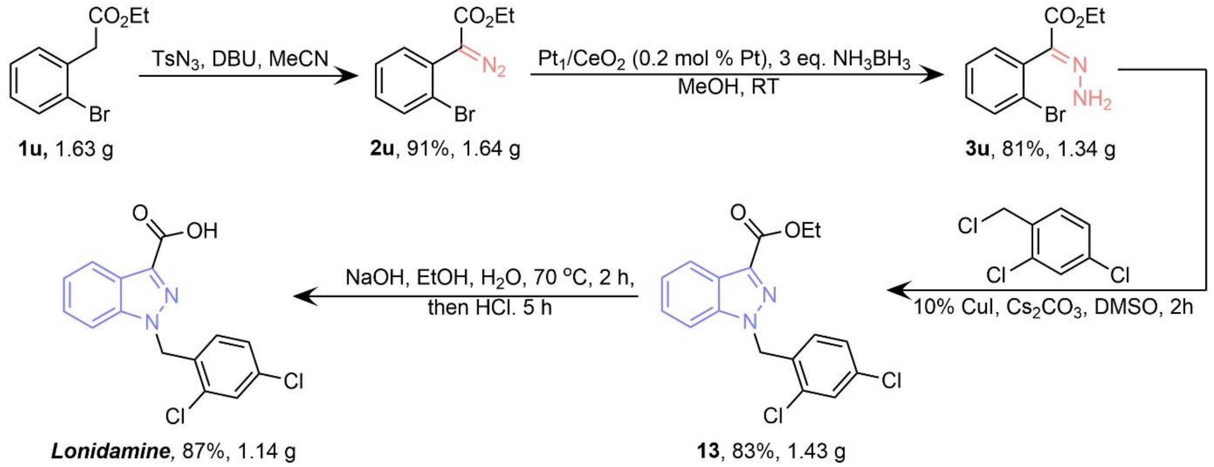

Fig. S25. Gram-scale synthesis of lonidamine using Pt_1 SACs-catalysed, selective *E*-hydrazone conversion with an overall yield of 53%.

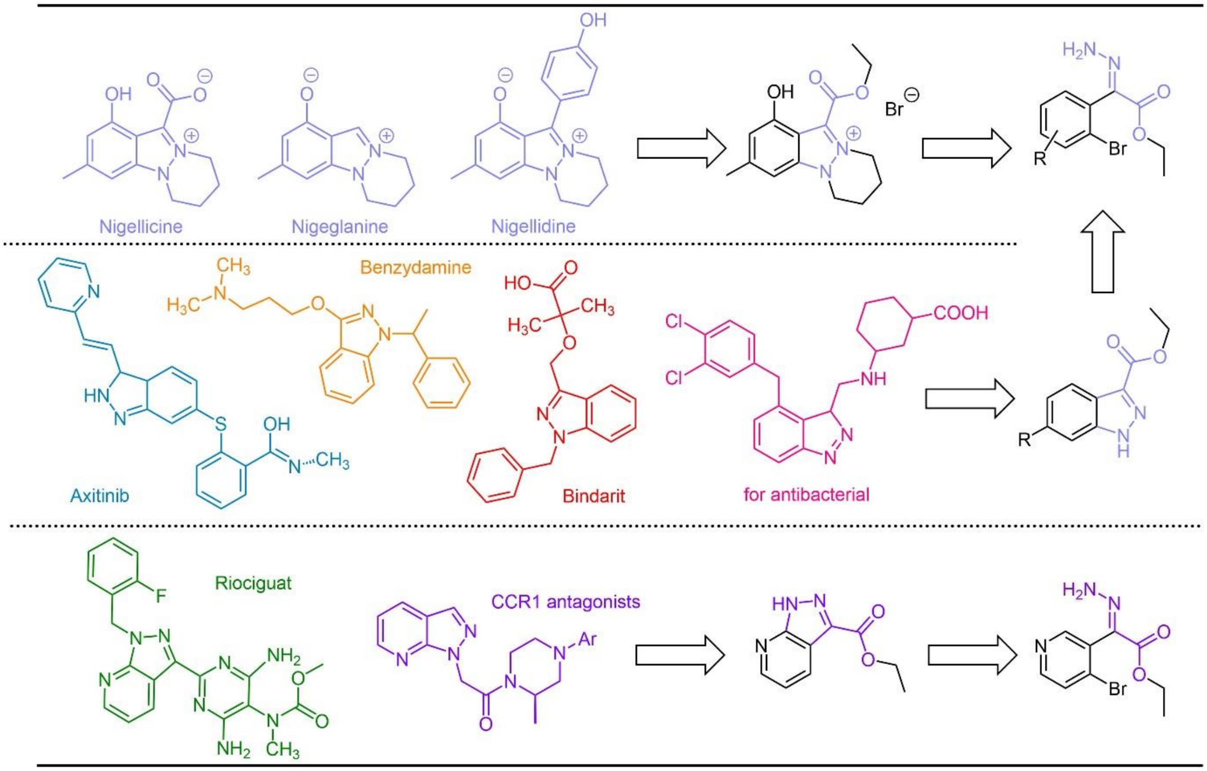

Fig. S26. Retrosynthesis of pharmaceuticals using the *E*-hydrazone strategy.

Table S1. Results of DFT calculations.

	N	R	Sigma^2	R-factor
PtO_2	6.0	2.01 ± 0.02	0.0012 ± 0.0002	0.003
Pt_1/CeO_2	4.1 ± 0.4	2.01 ± 0.02	0.0022 ± 0.0002	0.009

Table S2. Catalyst screening for selective *E*-hydrazone synthesis. [a)]

Entry	Catalyst	Loading	Selectivity	Isolated Yield
1	--	--	-	-
2	Porous CeO_2	3 mg	-	-
3	Pt_1/CeO_2	3 mg	81%	80%
4	10% Pt/C	0.4 mg	77%	42%
5	Pt NPs/CeO_2	3 mg	76%	49%
6	Pt_1/graphene	3 mg	60%	58%
7	Pt_1/CeO_2-non	4.9 mg	80%	56%
8	Co_1/graphene	3 mg	-	Trace
9	Fe_1/graphene	3 mg	-	Trace

[a)] Test condition: ***2a*** (0.1 mmol), catalyst (0.2 μmol_{metal}, 0.2 mol% to substrate), NH_3BH_3 (0.6 mmol, 6 equiv.), 2 mL of MeOH, react for 40 min. The Pt loading for Pt_1/CeO_2, Pt NPs/CeO_2, Pt_1/graphene and Pt_1/CeO_2-non are 1.38, ~ 1.40, ~ 1.0 and 0.85 wt%, respectively. The Fe and Co loading on graphene are both ~ 1.0 wt% from ICP-OES.

Table S3. Solvent screening for selective *E*-hydrazone synthesis. [a)]

Entry	Catalyst	Loading	Solvent	Selectivity	Isolated Yield
3	Pt_1/CeO_2	3 mg	MeOH	81%	80%
10	Pt_1/CeO_2	3 mg	DMF/H_2O (1:1)	68%	45%
11	Pt_1/CeO_2	3 mg	THF/H_2O (1:1)	78%	39%
12	Pt_1/CeO_2	3 mg	AN/H_2O (1:1)	79%	35%
13	Pt_1/CeO_2	3 mg	Toluene/H_2O (1:1)	78%	51%
14	Pt_1/CeO_2	3 mg	CH_3Cl/H_2O (1:1)	80%	9%

[a)] Test condition: ***2a*** (0.1 mmol), catalyst (0.2 μmol_{Pt}, 0.2 mol% to substrate), NH_3BH_3 (0.6 mmol, 6 equiv.), 2 mL of solvent, react for 40 min.

Table S4. Borane screening for selective *E*-hydrazone synthesis. [a)]

Entry	Catalyst	Borane	Time	Isolated Yield
3	Pt_1/CeO_2	NH_3BH_3	40 min	80%
15	Pt_1/CeO_2	--	40 min	-
16	Pt_1/CeO_2	NH_3BH_3 [b)]	40 min	80%
17	Pt_1/CeO_2	NH_3BH_3 [c)]	40 min	65% (77%)[d]
18	Pt_1/CeO_2	8 atm H_2	40 min	37%
19	Pt_1/CeO_2	$(CH_2)_4O{\cdot}BH_3$	36 h	Trace
20	Pt_1/CeO_2	$(CH_3)_2S{\cdot}BH_3$	36 h	Trace
21	Pt_1/CeO_2	$(CH_3)_3N{\cdot}BH_3$	36 h	4%
22	Pt_1/CeO_2	$(CH_3)_2NH{\cdot}BH_3$	36 h	33%
23	Pt_1/CeO_2	*t*BuNH$_2{\cdot}BH_3$	36 h	63%

[a)] Test condition: ***2a*** (0.1 mmol), catalyst (0.2 μmol_{Pt}, 0.2 mol% to substrate), borane (0.6 mmol, 6 equiv.), 2 mL of solvent, react for 40 min; [b)] NH_3BH_3 (0.3 mmol, 3 equiv.); [c)] NH_3BH_3 (0.15 mmol, 1.5 equiv.); [d)] react for 2 h.

Table S5. Results of the EXAFS fitting on PtO_2 and Pt_1/CeO_2.

DFT Model	Energy (eV)
hydrazone free molecule - *EE*	-146.74
hydrazone free molecule - *EZ*	-146.70
hydrazone free molecule - *ZE*	-146.73
hydrazone free molecule - *ZZ*	-146.93
Diazo substrate free molecule	-138.79
	Adsorption Energy (eV)
EZ isomer on Pt_1/CeO_2	-1.81
ZZ isomer on Pt_1/CeO_2	-0.77
Diazo substrate on Pt_1/CeO_2	-1.97
Diazo substrate on O vacancy	-0.79
H on Pt_1/CeO_2 (Pt site)	-3.162

Table S6. Representative methods for the total-synthesis of lonidamine.

Method	Total yield (%)[a]	Steps	Total reaction time (h)	Toxic reagents
**Ours**	**58**[b]	**4**	**~ 15**	**No**
Tang' method[c]	28	7	~ 119	CrO_3
Wang' method[d]	36	6	~ 92	CrO_3
Zhu' method[e]	38	5	-	Chloral Hydrate
Veerareddy' method[f]	-	7	-	CuCN

[a] Total yield is calculated from the starting materials including all reaction steps; [b] our reaction can be finished in two-pot with the total yield of 42%; [c] *Adv. Synth. Catal.* **2016**, *358*, 926; [d] *Adv. Synth. Catal.* **2017**, *359*, 2747; [e] *J. Heterocyclic Chem.* **2014**, *51*, 1311; [f] 10.13550/j.jxhg.

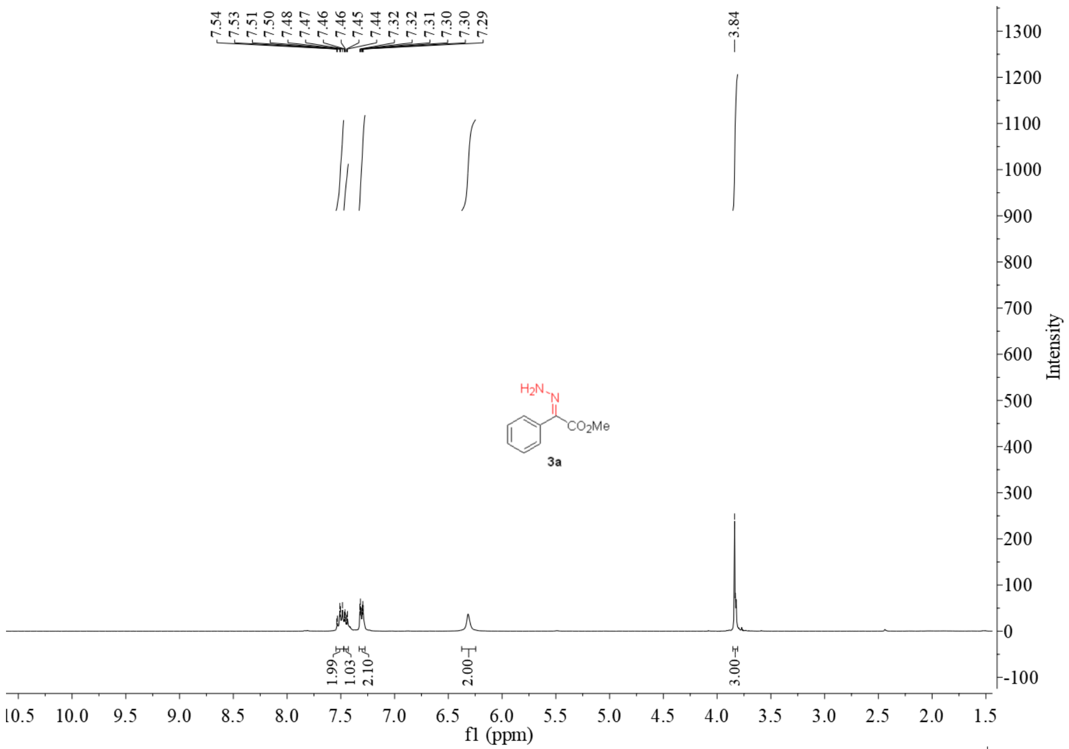

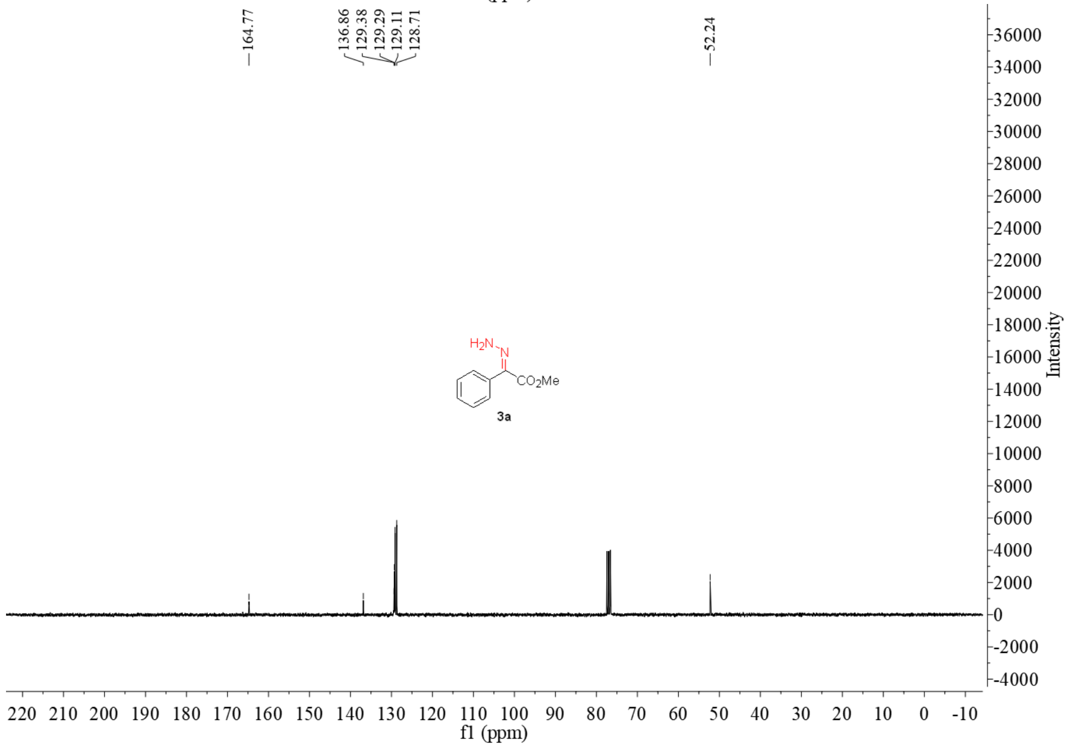

^{1}H, ^{13}C-NMR spectra of product 3a. ^{1}H NMR (300 MHz, Chloroform-*d*) δ [ppm] 7.55 – 7.47 (m, 2H), 7.47 – 7.43 (m, 1H), 7.31 (dt, J = 5.7, 1.5 Hz, 2H), 6.31 (s, 2H), 3.84 (s, 3H); **^{13}C NMR** (75 MHz, Chloroform-*d*) δ [ppm] 164.8, 136.9, 129.4, 129.3, 129.1, 128.7, 52.2; **HRMS** (ESI) 201.0630, theoretical value for $C_9H_{10}N_2O_2$ $[M+Na]^+$ 201.0622.

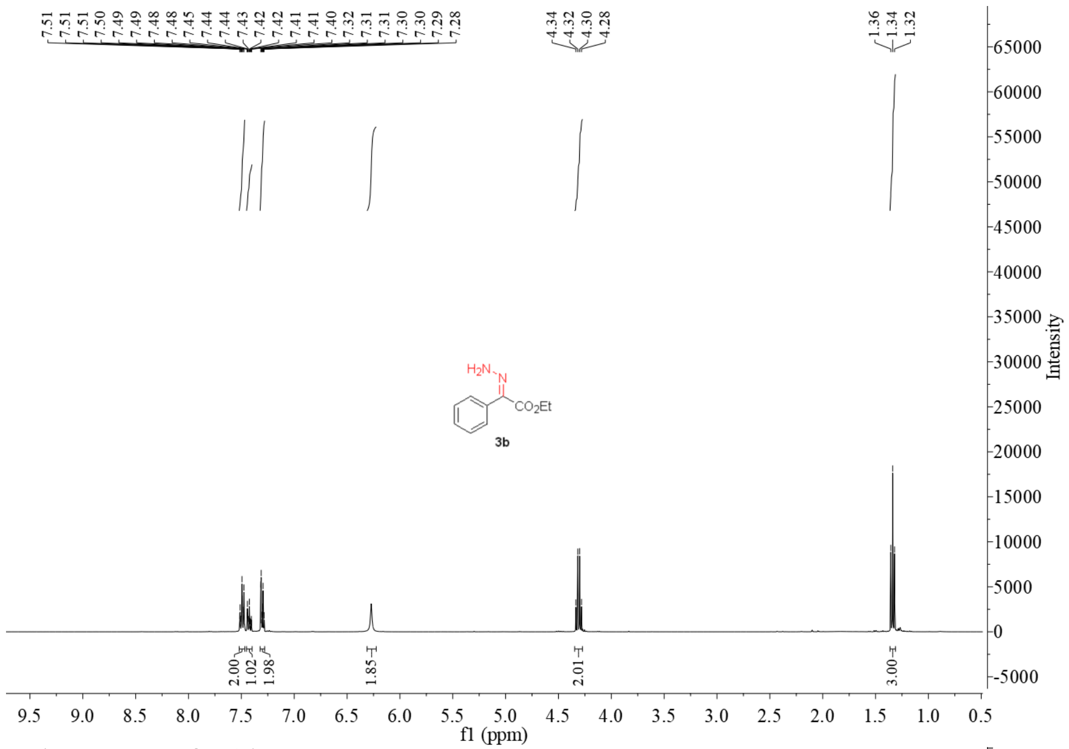

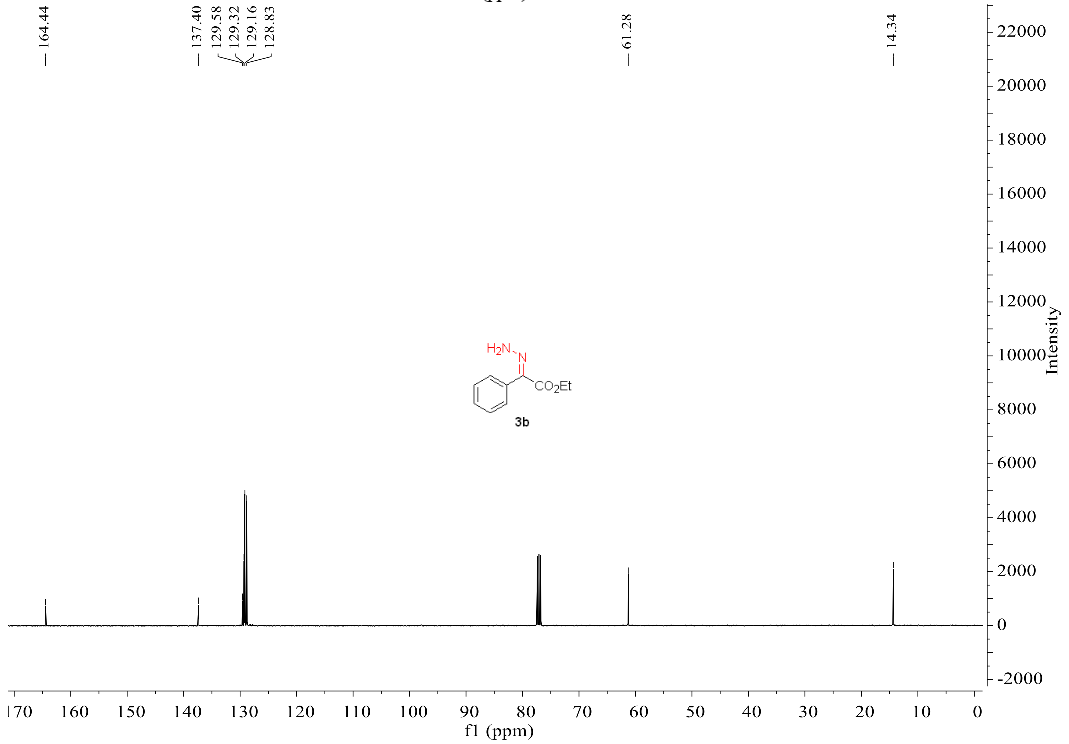

^{1}H, ^{13}C-NMR spectra of product 3b. **^{1}H NMR** (400 MHz, Chloroform-*d*) δ [ppm] 7.49 (ddd, J = 7.5, 6.3, 1.3 Hz, 2H), 7.45 – 7.40 (m, 1H), 7.32 – 7.28 (m, 2H), 6.27 (s, 2H), 4.31 (q, J = 7.1 Hz, 2H), 1.34 (t, J = 7.1 Hz, 3H); **^{13}C NMR** (100 MHz, Chloroform-*d*) δ [ppm] 164.4, 137.4, 129.6, 129.3, 129.2, 128.8, 61.3, 14.3; **HRMS** (ESI) 215.0790, theoretical value for $C_{10}H_{12}N_2O_2$ $[M+Na]^+$ 215.0797.

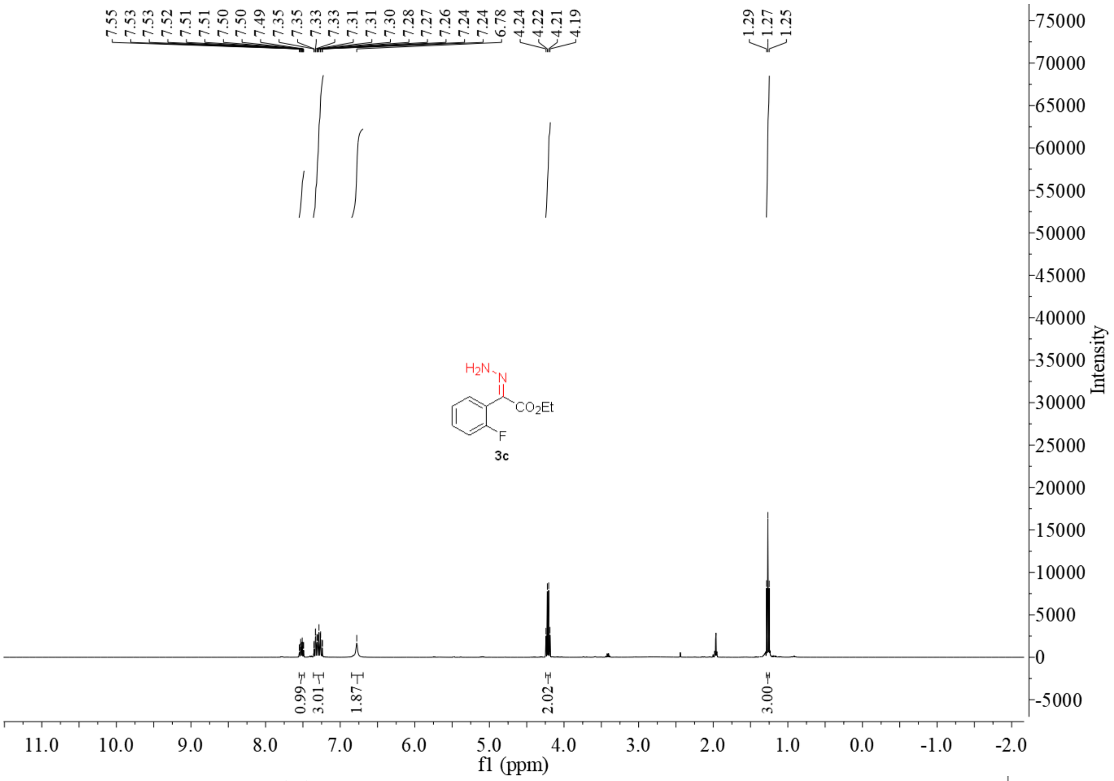

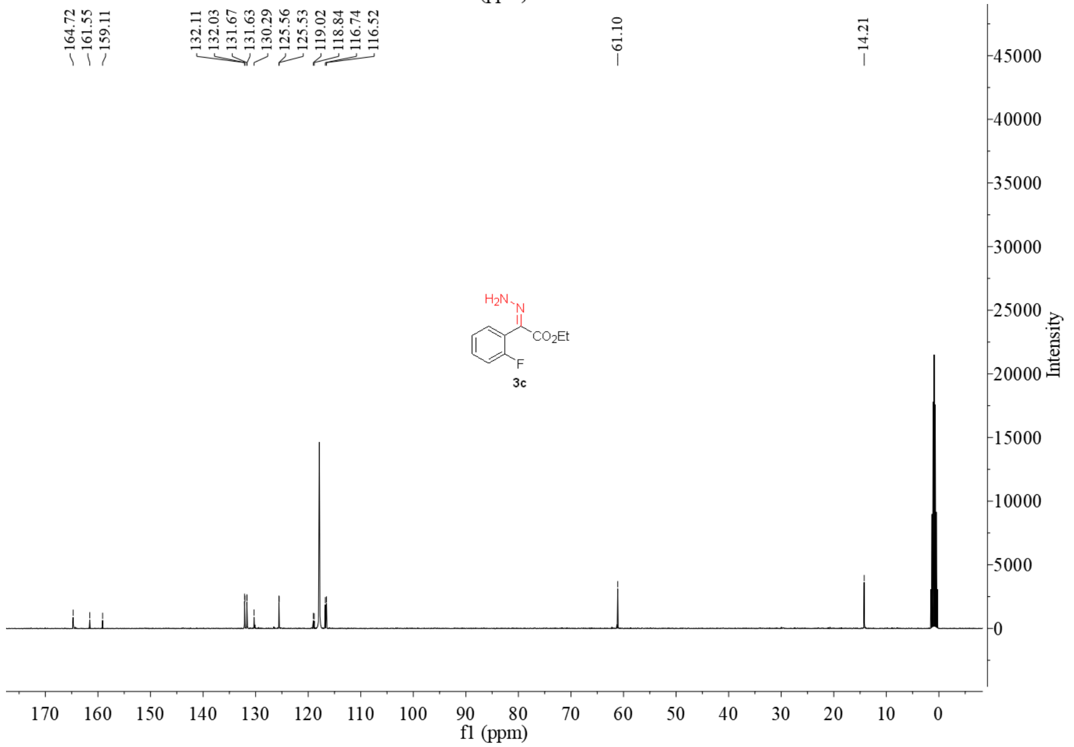

^{1}H, ^{13}C-NMR spectra of product 3c. **^{1}H NMR** (400 MHz, Acetonitrile-d_3) δ [ppm] 7.55 – 7.48 (m, 1H), 7.36 – 7.22 (m, 3H), 6.78 (s, 2H), 4.21 (q, J = 7.1 Hz, 2H), 1.27 (t, J = 7.1 Hz, 3H); **^{13}C NMR** (100 MHz, Acetonitrile-d_3) δ [ppm] 164.7, 160.3 (d, J^{1}_{F} = 244 Hz), 132.1 (d, J^{3}_{F} = 8 Hz), 131.6 (d, J^{3}_{F} = 4 Hz), 130.3, 125.5 (d, J^{4}_{F} = 3 Hz), 118.9 (d, J^{2}_{F} = 18 Hz), 116.6 (d, J^{2}_{F} = 22 Hz), 61.1, 14.2; **HRMS** (ESI) 233.0694, theoretical value for $C_{10}H_{11}FN_2O_2$ $[M+Na]^+$ 233.0703.

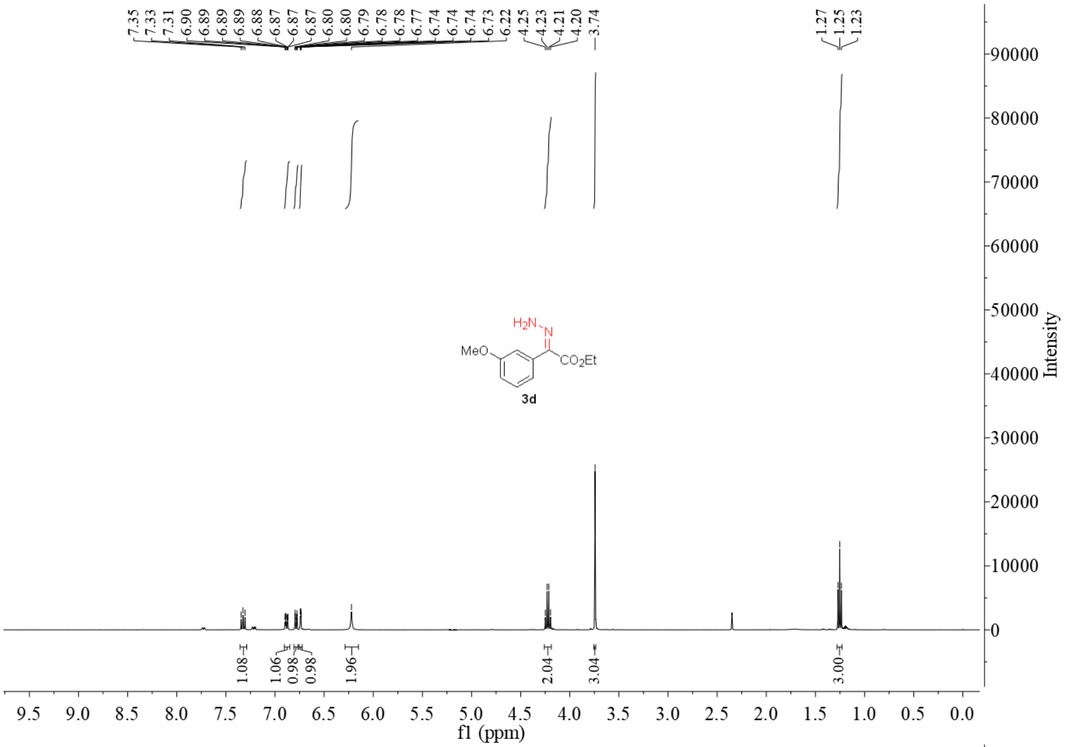

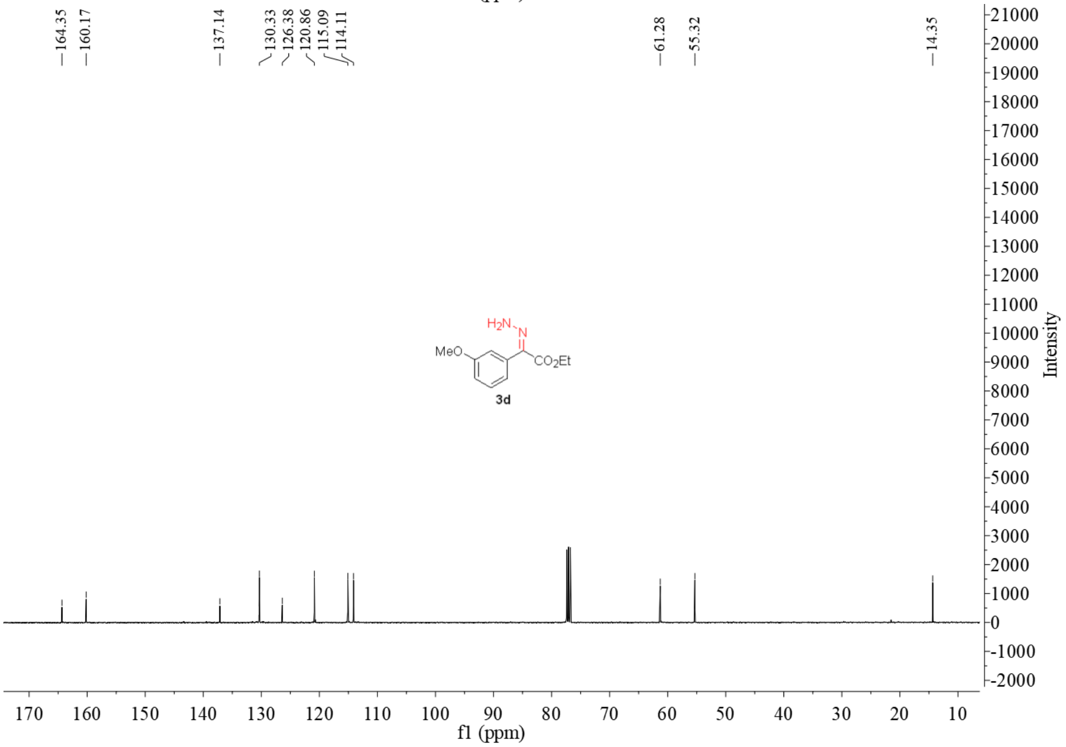

^{1}H, ^{13}C-NMR spectra of product 3d. **^{1}H NMR** (400 MHz, Chloroform-*d*) δ [ppm] 7.36 – 7.29 (m, 1H), 6.88 (ddd, J = 8.4, 2.6, 0.9 Hz, 1H), 6.79 (dt, J = 7.5, 1.1 Hz, 1H), 6.74 (dd, J = 2.5, 1.4 Hz, 1H), 6.22 (s, 2H), 4.22 (q, J = 7.1 Hz, 2H), 3.74 (s, 3H), 1.25 (t, J = 7.1 Hz, 3H); **^{13}C NMR** (100 MHz, Chloroform-*d*) δ [ppm] 164.4, 160.2, 137.1, 130.3, 126.4, 120.9, 115.1, 114.1, 61.3, 55.3, 14.4; **HRMS** (ESI) 245.0892, theoretical value for $C_{11}H_{14}N_2O_3$ $[M+Na]^+$ 245.0902.

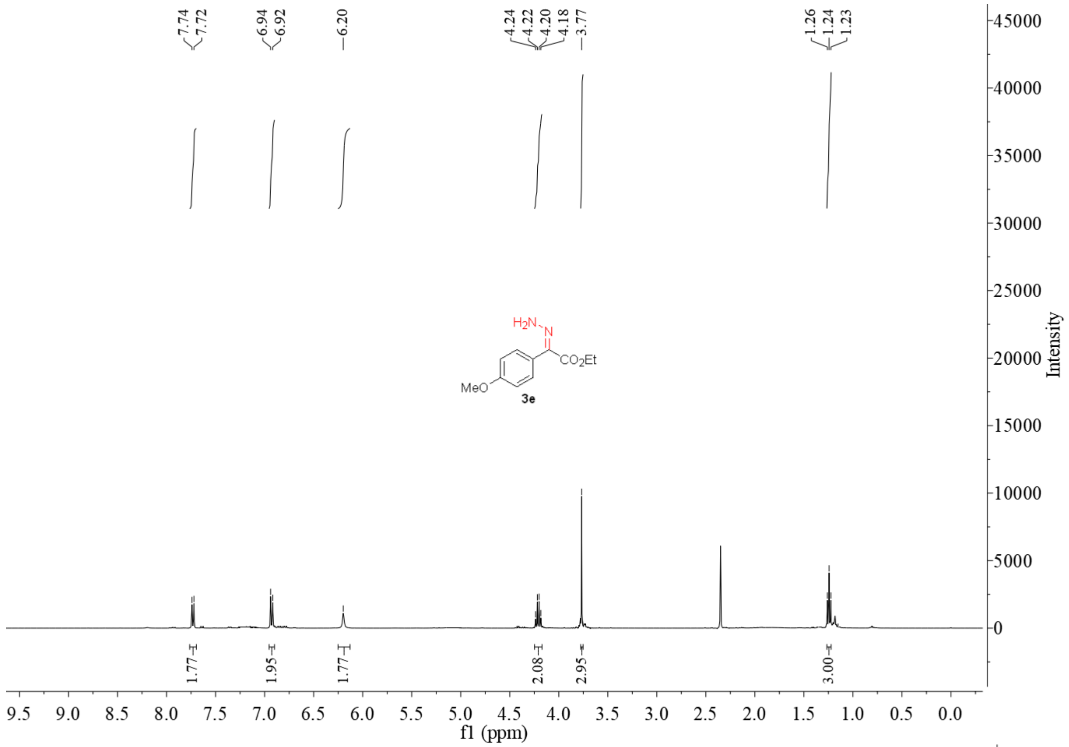

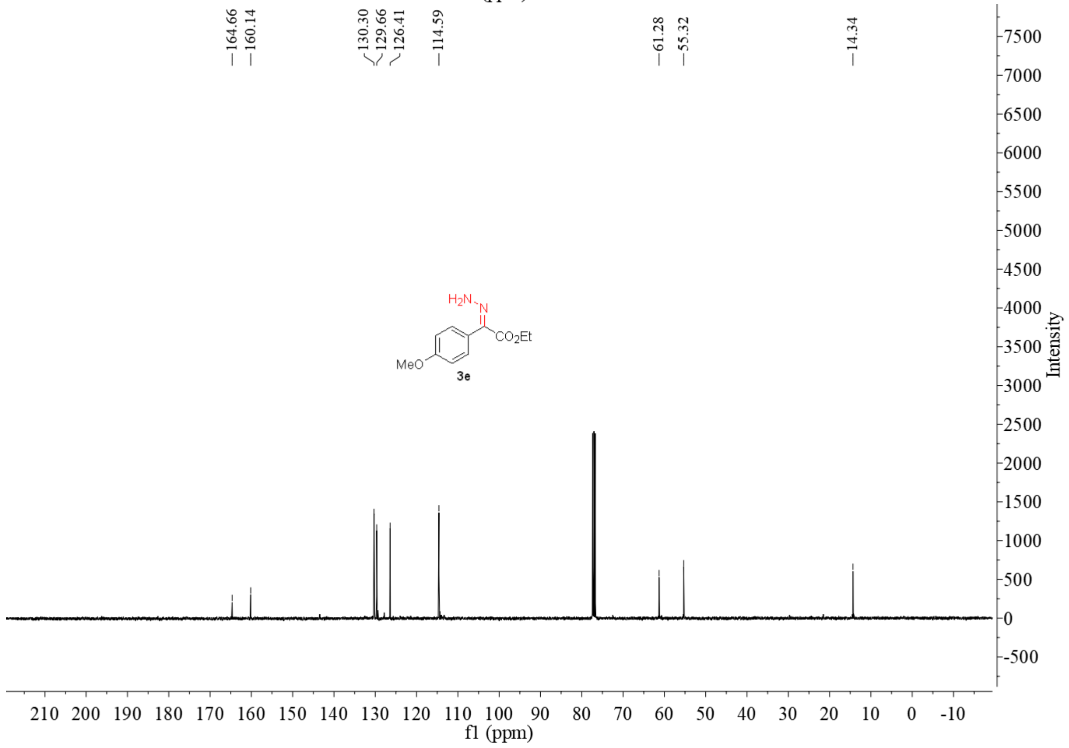

^{1}H, ^{13}C-NMR spectra of product 3e. **^{1}H NMR** (400 MHz, Chloroform-*d*) δ [ppm] 7.73 (d, J = 8.3 Hz, 2H), 6.93 (d, J = 8.7 Hz, 2H), 6.20 (s, 2H), 4.21 (q, J = 7.2 Hz, 2H), 3.77 (s, 3H), 1.24 (t, J = 7.1 Hz, 3H); **^{13}C NMR** (100 MHz, Chloroform-*d*) δ [ppm] 164.7, 160.1, 130.3, 129.7, 126.4, 114.6, 61.3, 55.3, 14.3; **HRMS** (ESI) 245.0892, theoretical value for $C_{11}H_{14}N_2O_3$ $[M+Na]^+$ 245.0902.

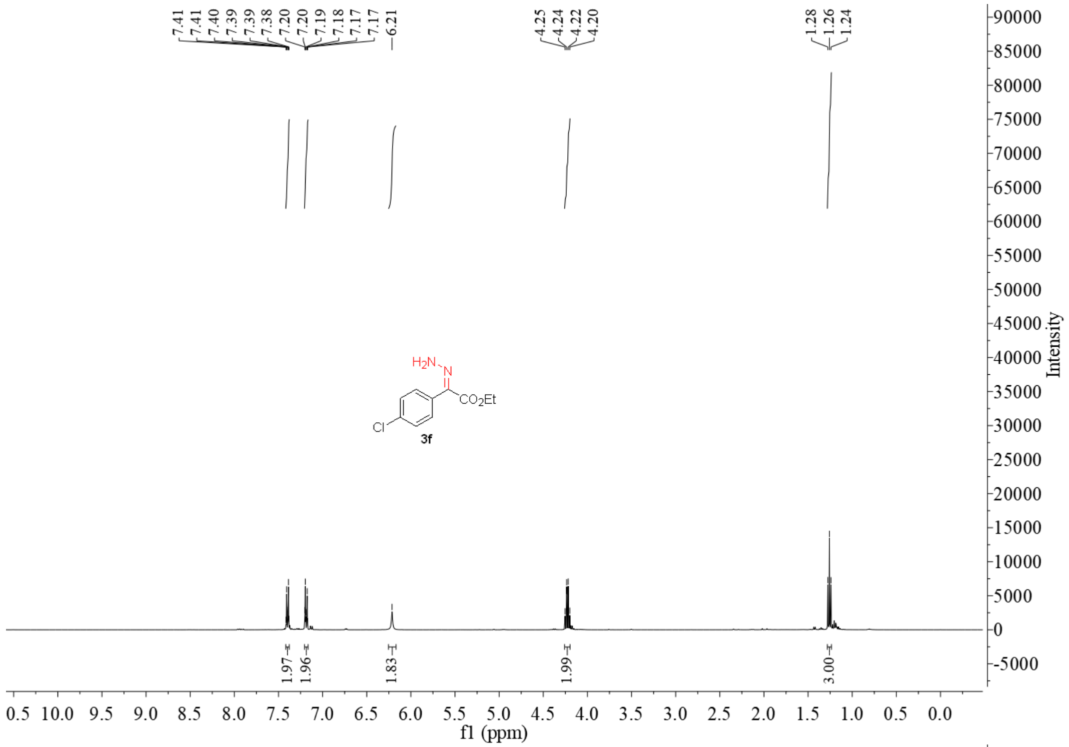

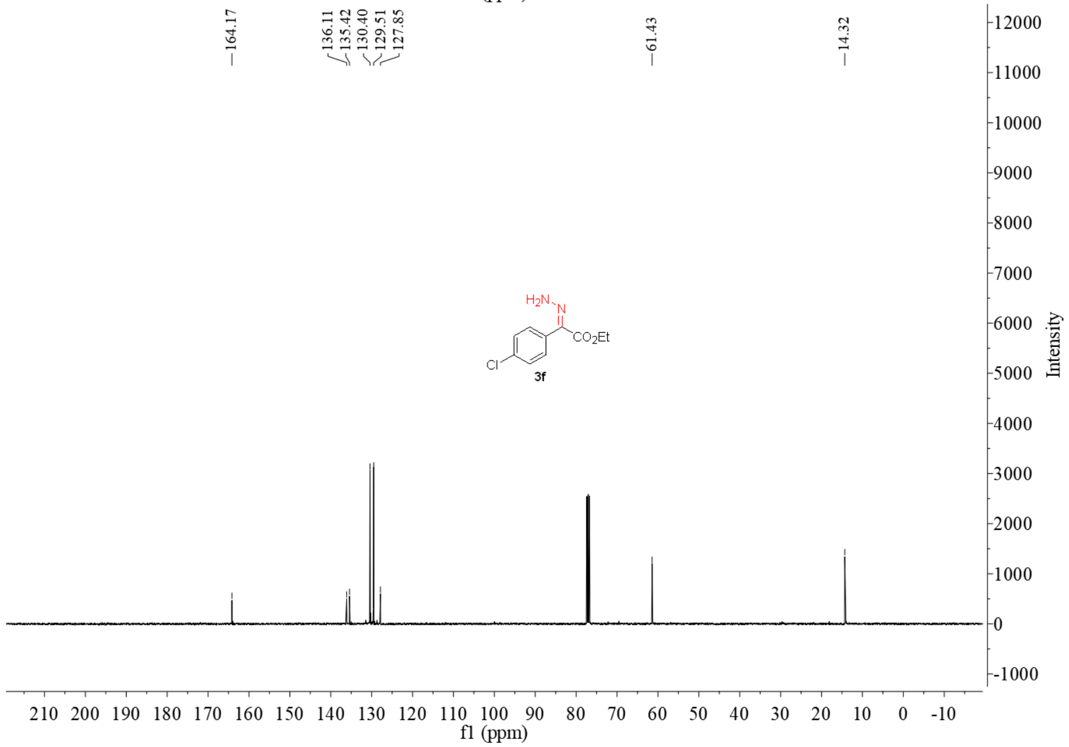

^{1}H, ^{13}C-NMR spectra of product 3f. **^{1}H NMR** (400 MHz, Chloroform-*d*) δ [ppm] 7.42 – 7.38 (m, 2H), 7.19 (dt, J = 8.9, 1.9 Hz, 2H), 6.21 (s, 2H), 4.23 (q, J = 7.1 Hz, 2H), 1.26 (t, J = 7.1 Hz, 3H); **^{13}C NMR** (100 MHz, Chloroform-*d*) δ [ppm] 164.2, 136.1, 135.4, 130.4, 129.5, 127.8, 61.4, 14.3; **HRMS** (ESI) 249.0401, theoretical value for $C_{10}H_{11}ClN_2O_2$ $[M+Na]^+$ 249.0407.

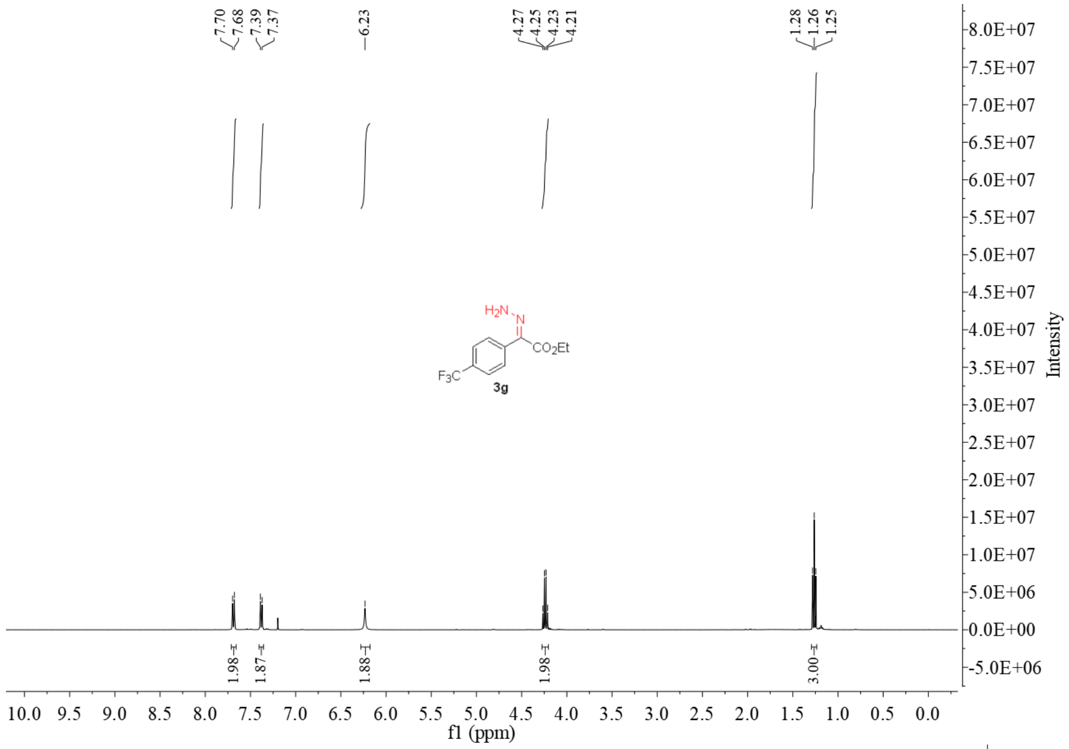

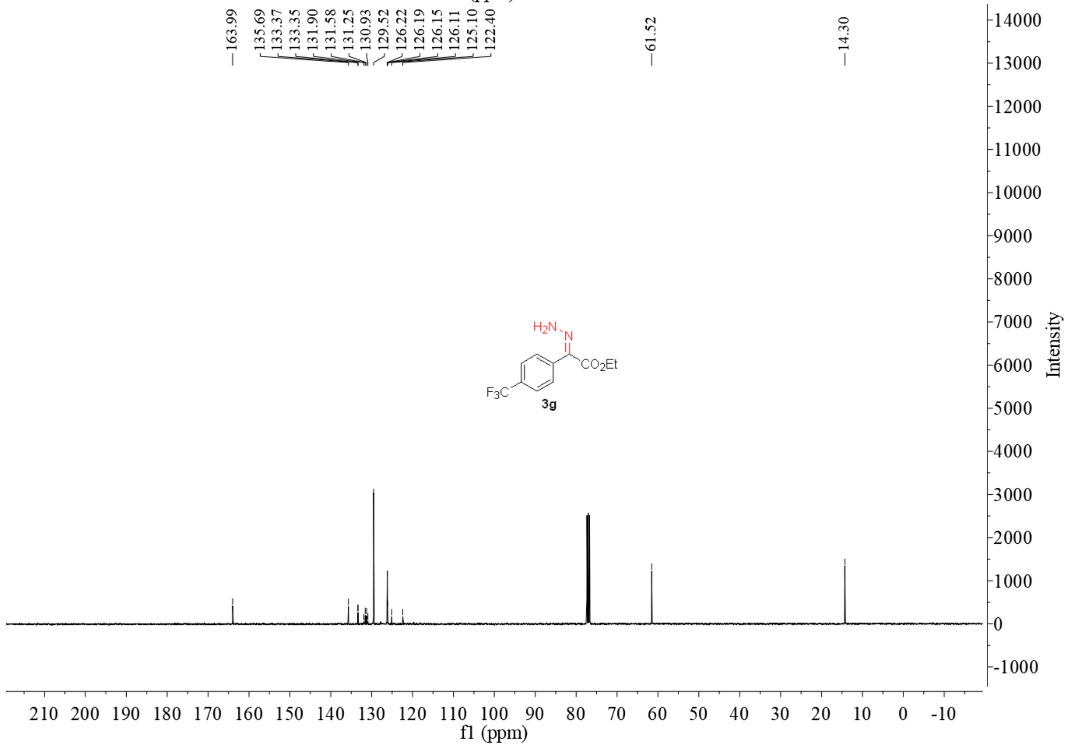

^{1}H, ^{13}C-NMR spectra of product 3g. **^{1}H NMR** (400 MHz, Chloroform-*d*) δ [ppm] 7.69 (d, J = 8.1 Hz, 2H), 7.38 (d, J = 7.9 Hz, 2H), 6.23 (s, 2H), 4.24 (q, J = 7.1 Hz, 2H), 1.26 (t, J = 7.1 Hz, 3H); **^{13}C NMR** (100 MHz, Chloroform-*d*) δ [ppm] 164.0, 135.7, 133.6 (d, $J^4{}_F$ = 2.0 Hz), 131.4 (q, $J^1{}_F$ = 32.5 Hz), 129.5, 126.1 (q, $J^3{}_F$ = 4.0 Hz), 123.8 (q, $J^1{}_F$ = 270.0 Hz), 61.5, 14.3; **HRMS** (ESI) 283.0658, theoretical value for $C_{11}H_{11}F_3N_2O_2$ $[M+Na]^+$ 283.0671.

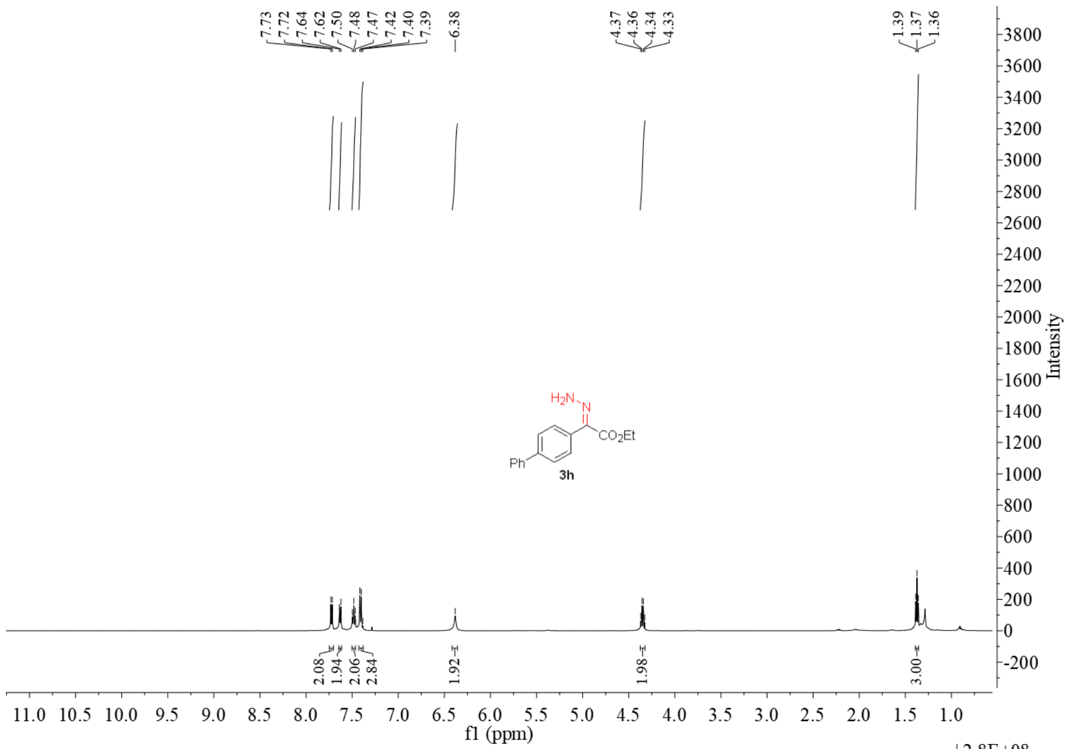

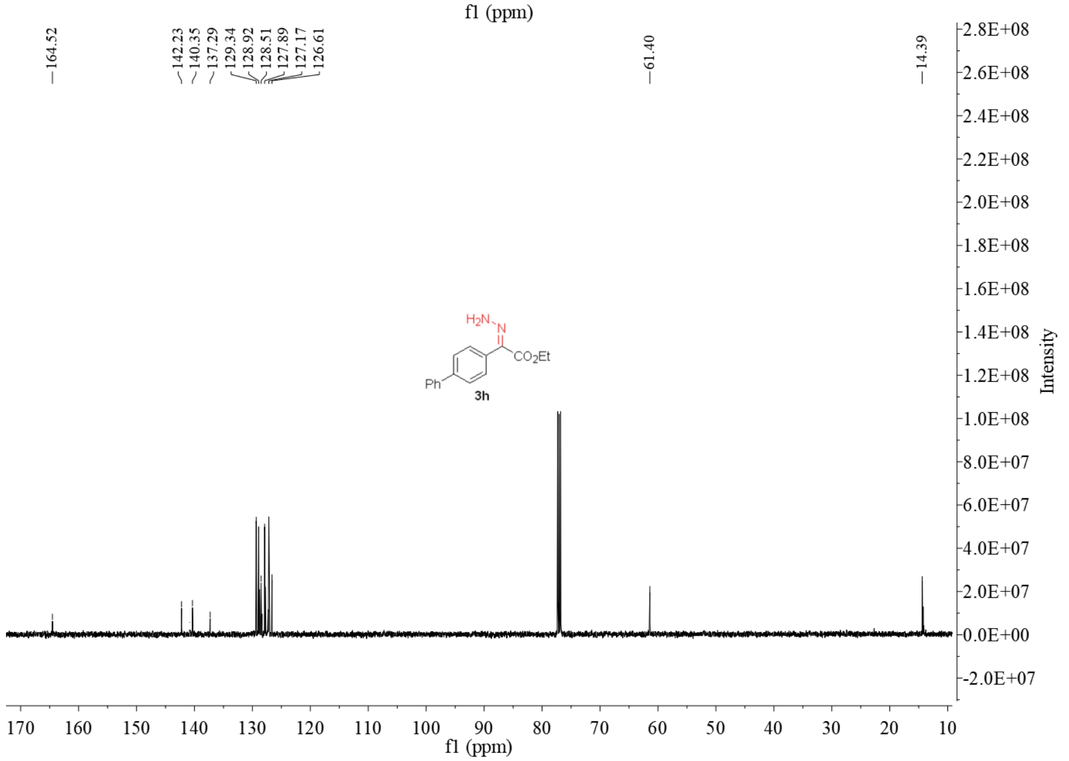

^{1}H, ^{13}C-NMR spectra of product 3h. **^{1}H NMR** (500 MHz, Chloroform-*d*) δ [ppm] 7.72 (d, J = 8.1 Hz, 2H), 7.63 (d, J = 8.4 Hz, 2H), 7.48 (t, J = 7.6 Hz, 2H), 7.41 (d, J = 8.0 Hz, 3H), 6.38 (s, 2H), 4.35 (q, J = 7.1 Hz, 2H), 1.37 (t, J = 7.1 Hz, 3H); **^{13}C NMR** (125 MHz, Chloroform-*d*) δ [ppm] 164.5, 142.2, 140.4, 137.3, 129.3, 128.9, 128.5, 127.9, 127.2, 126.6. 61.4, 14.4; **HRMS** (ESI) 291.1113, theoretical value for $C_{16}H_{16}N_2O_2$ 291.1111.

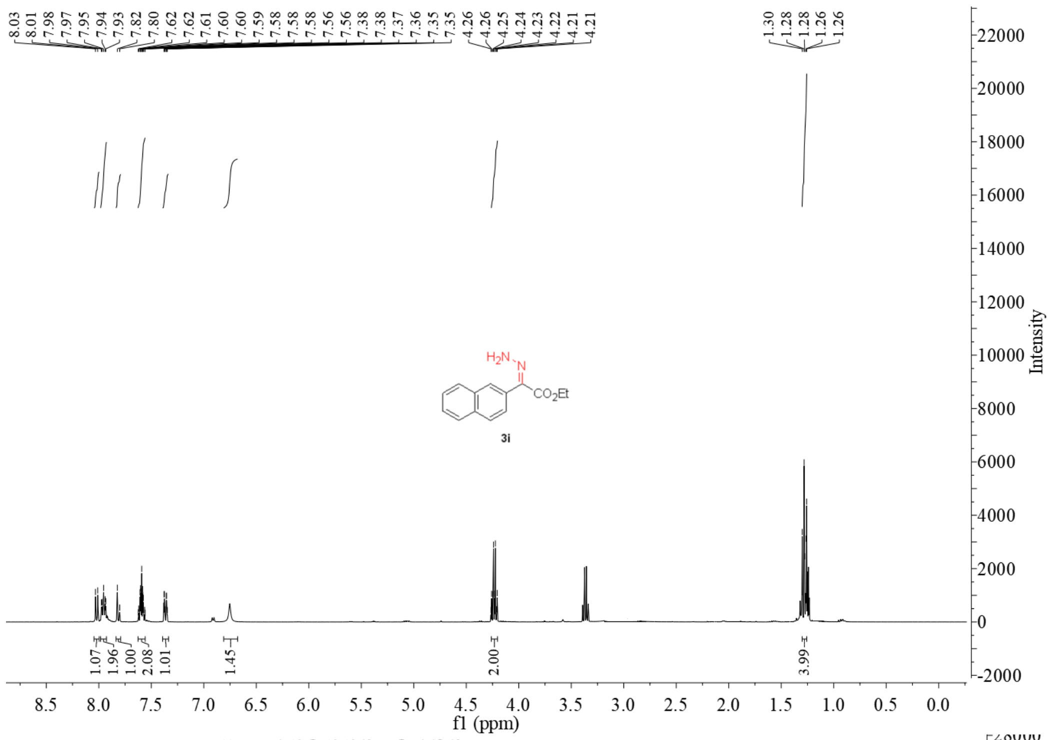

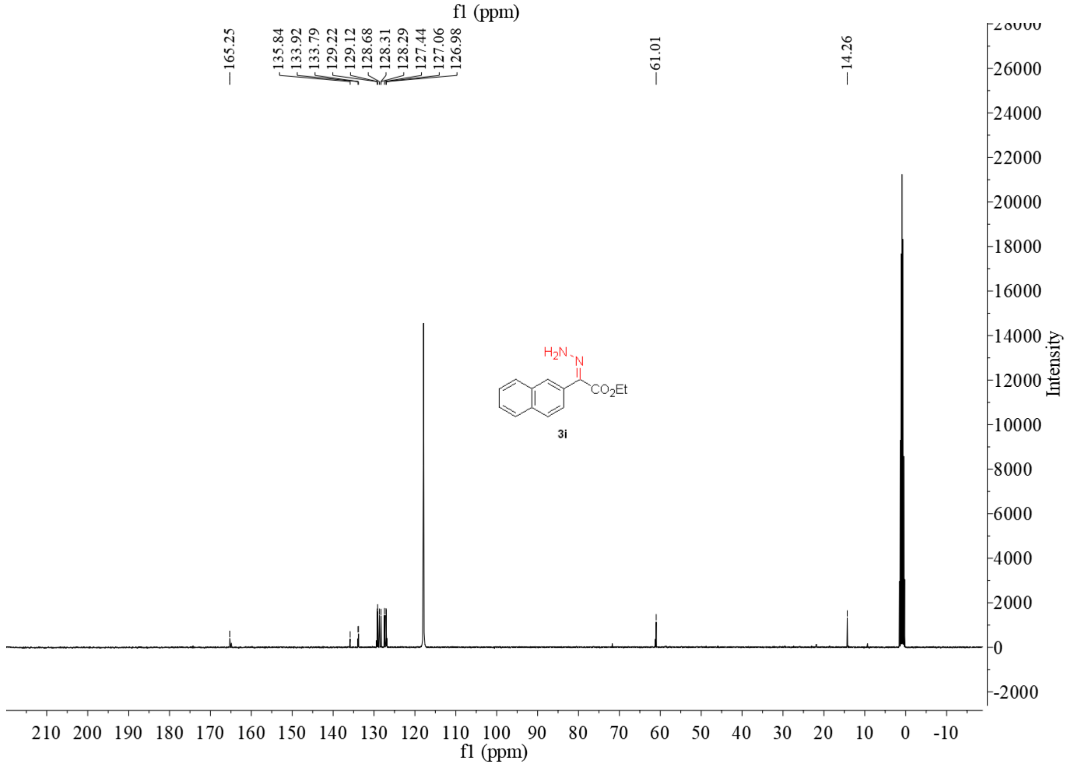

^{1}H, ^{13}C-NMR spectra of product 3i. ^{1}H NMR (400 MHz, Acetonitrile-d_3) δ [ppm] 8.02 (d, J = 8.5 Hz, 1H), 7.98 – 7.93 (m, 2H), 7.82 (s, 1H), 7.63 – 7.56 (m, 2H), 7.36 (dt, J = 8.4, 2.2 Hz, 1H), 6.75 (s, 2H), 4.26 – 4.20 (m, 2H), 1.30 – 1.26 (m, 3H); **^{13}C NMR** (100 MHz, Acetonitrile-d_3) δ [ppm] 165.3, 135.8, 133.9, 133.8, 129.2, 129.1, 128.7, 128.3, 128.3, 127.4, 127.1, 127.0, 61.0, 14.3; **HRMS** (ESI) 265.0944, theoretical value for $C_{14}H_{14}N_2O_2$ [M+Na]$^+$ 265.0953.

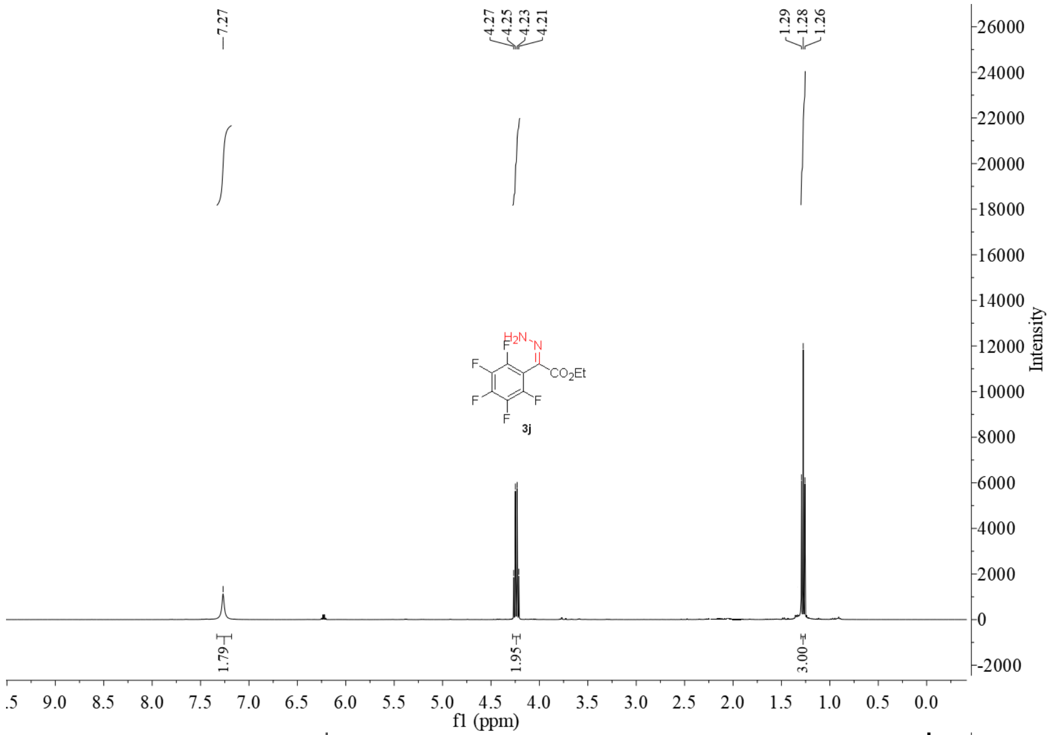

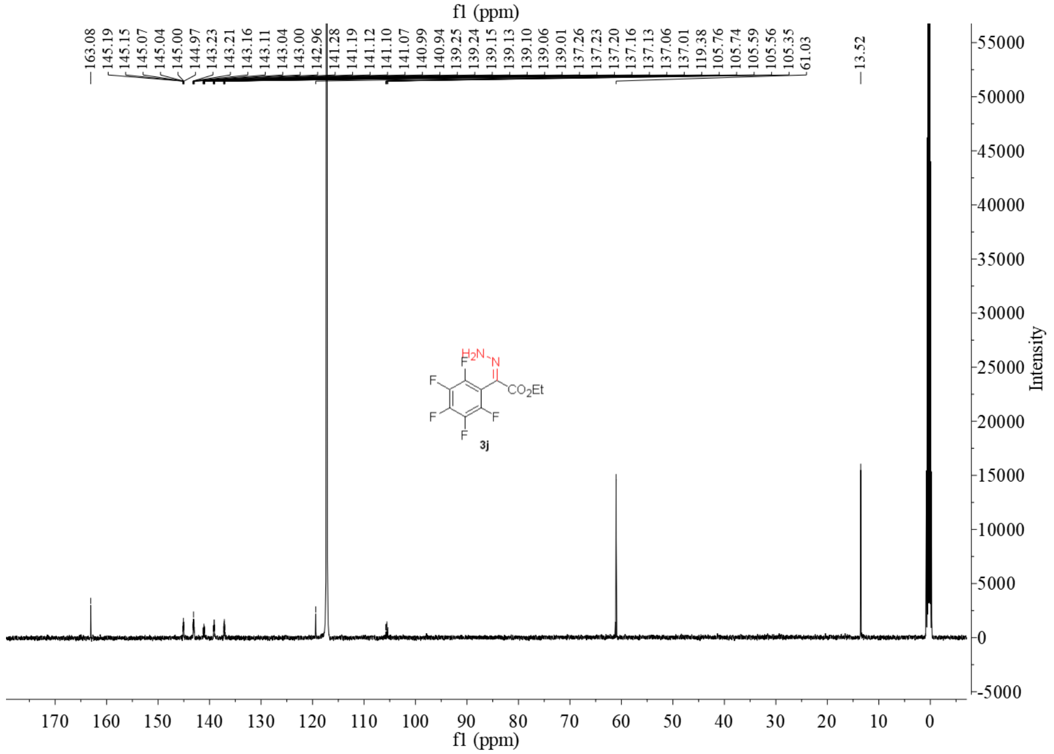

^{1}H, ^{13}C-NMR spectra of product 3j. **^{1}H NMR** (400 MHz, Acetonitrile-d_3) δ [ppm] 7.27 (s, 2H), 4.24 (q, J = 7.1 Hz, 2H), 1.28 (t, J = 7.1 Hz, 3H); **^{13}C NMR** (100 MHz, Acetonitrile-d_3) δ [ppm] 163.1, 145.0 (m), 143.1 (m), 141.1 (m), 139.1 (m), 137.1 (m), 119.4, 105.6 (m), 61.0, 13.5; **HRMS** (ESI) 305.0326, theoretical value for $C_{10}H_7F_5N_2O_2$ 305.0324.

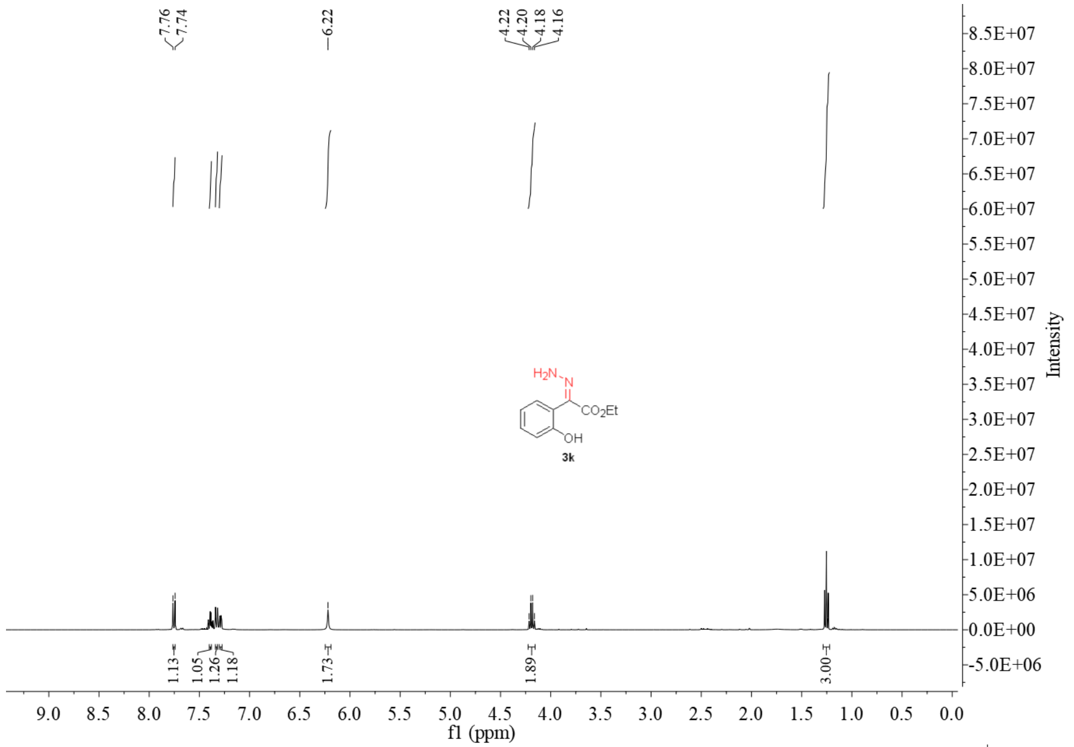

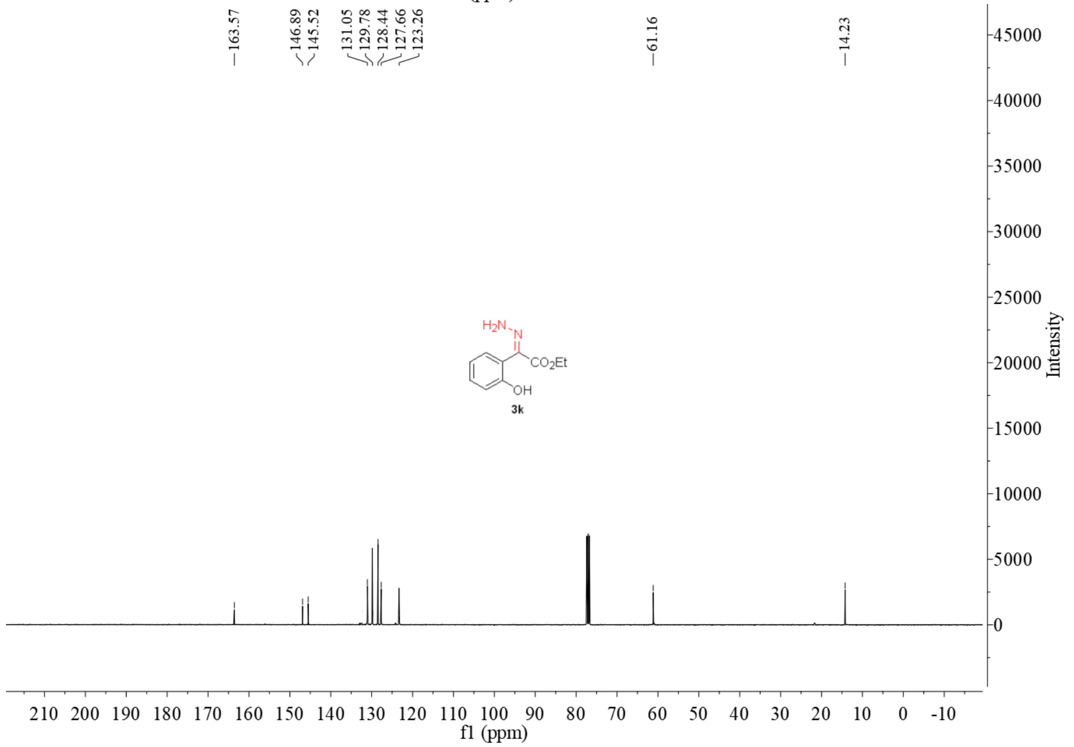

^{1}H, ^{13}C-NMR spectra of product 3k. ^{1}H NMR (400 MHz, Chloroform-*d*) δ [ppm] 7.75 (d, J = 8.4 Hz, 1H), 7.40 – 7.38 (m, 1H), 7.34 (s, 1H), 7.30 – 7.27 (m, 1H), 6.22 (s, 2H), 4.19 (q, J = 7.1 Hz, 2H), 1.25 (t, J = 7.1 Hz, 3H); **^{13}C NMR** (100 MHz, Chloroform-*d*) δ [ppm] 163.6, 146.9, 145.5, 131.0, 129.8, 128.4, 127.7, 123.3, 61.2, 14.2.

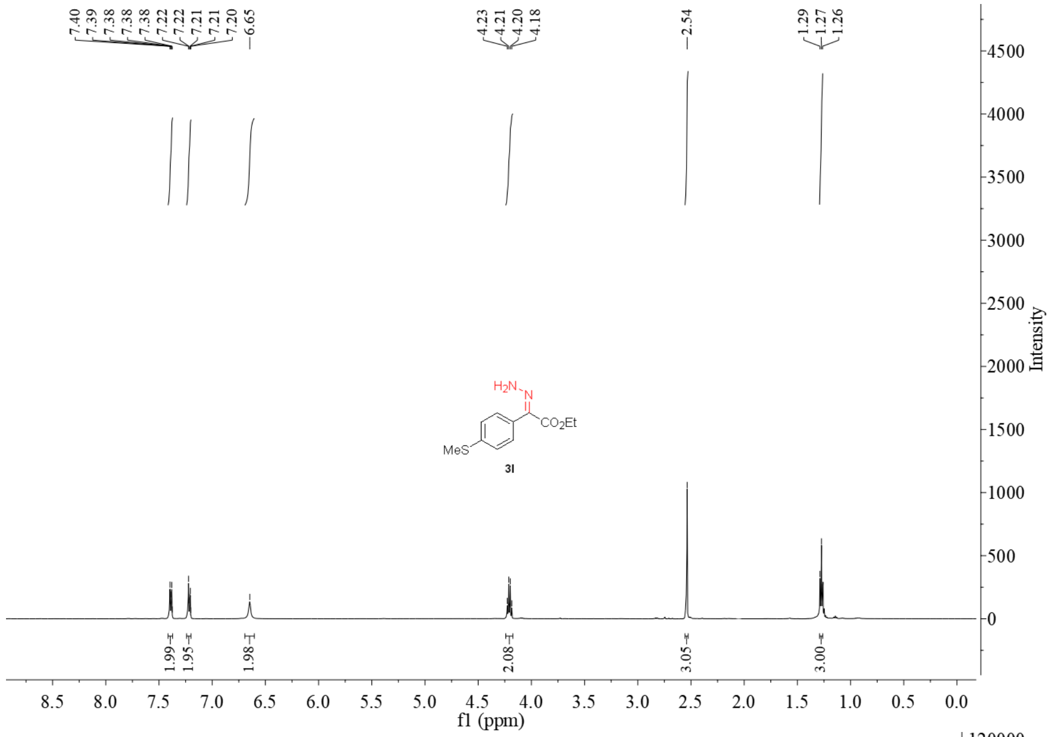

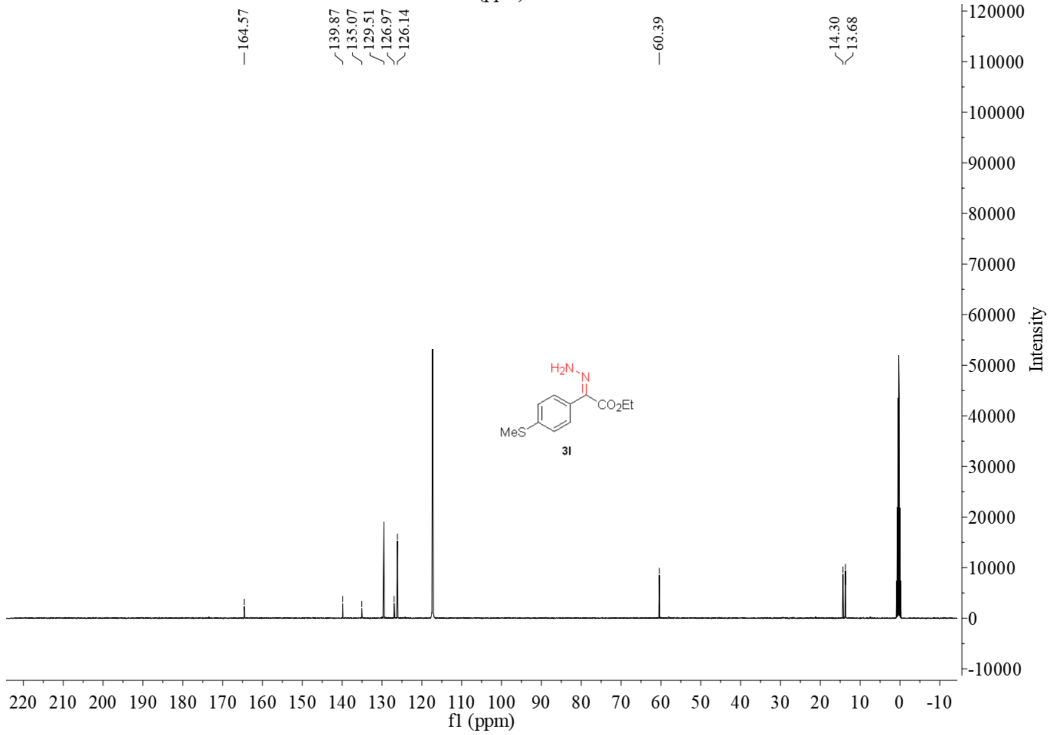

^{1}H, ^{13}C-NMR spectra of product 3l. **^{1}H NMR** (500 MHz, Acetonitrile-*d*$_3$) δ [ppm] 7.41 – 7.37 (m, 2H), 7.24 – 7.20 (m, 2H), 6.65 (s, 2H), 4.21 (q, J = 7.1 Hz, 2H), 2.54 (s, 3H), 1.27 (t, J = 6.0 Hz, 3H); **^{13}C NMR** (125 MHz, Acetonitrile-*d*$_3$) δ [ppm] 164.6, 139.9, 135.1, 129.5, 127.0, 126.1, 60.4, 14.3, 13.7; **HRMS** (ESI) 261.0668, theoretical value for $C_{11}H_{14}N_2O_2S$ $[M+Na]^+$ 261.0674.

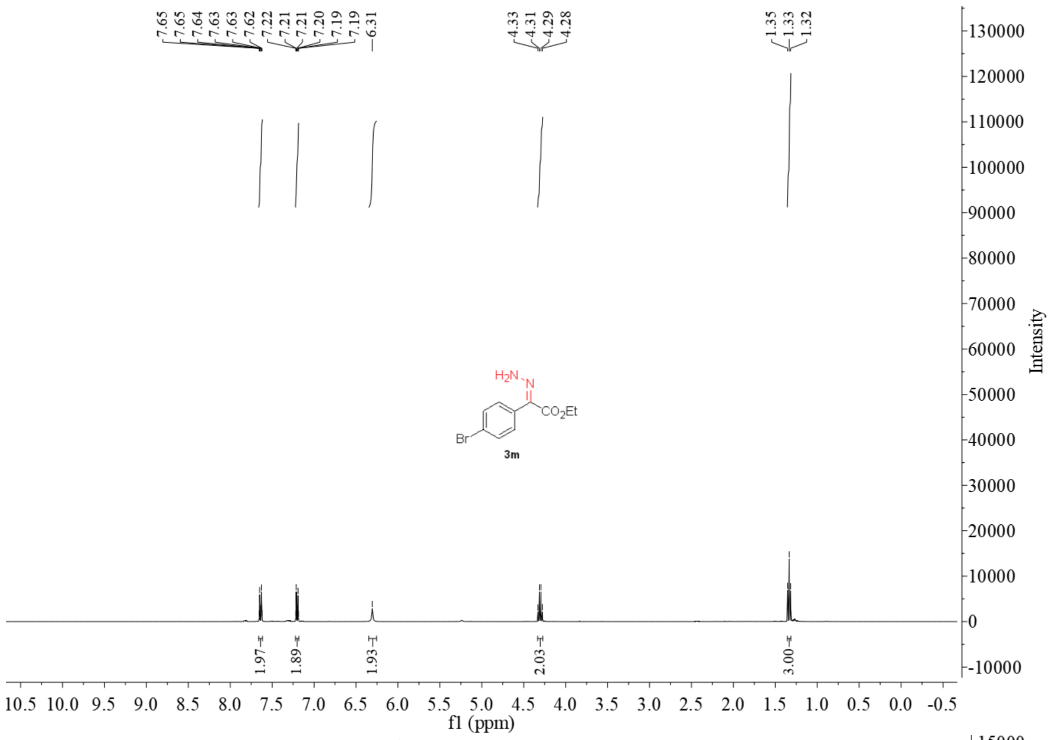

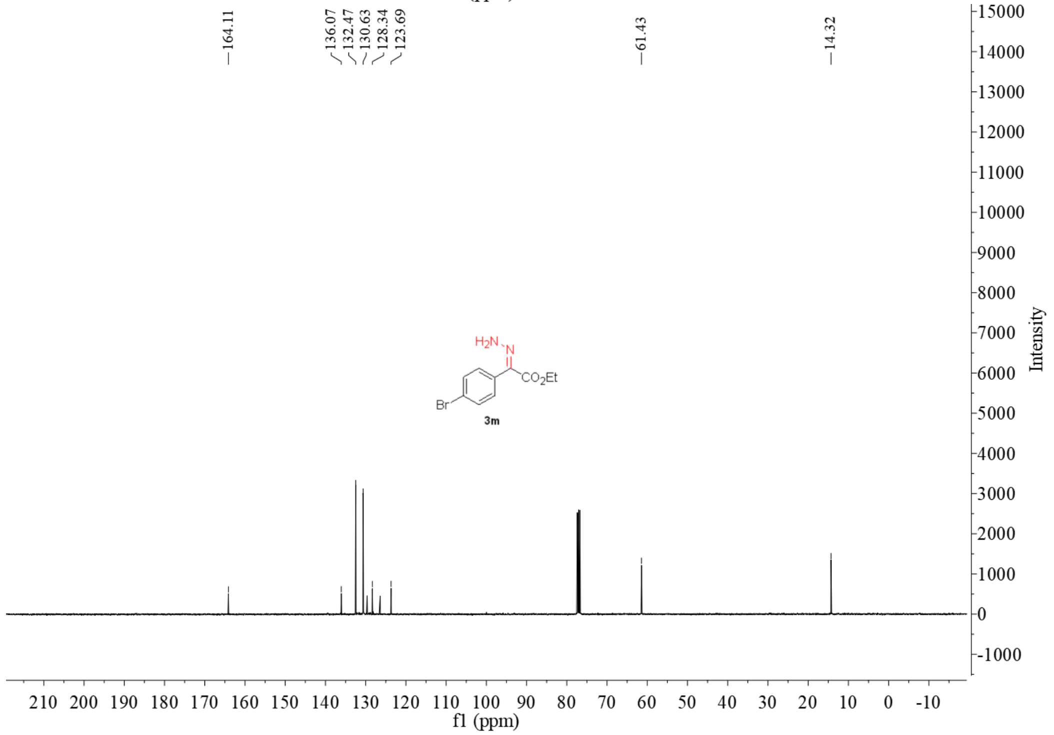

^{1}H, ^{13}C-NMR spectra of product 3m. ^{1}H NMR (400 MHz, Chloroform-*d*) δ [ppm] 7.66 – 7.62 (m, 2H), 7.22 – 7.18 (m, 2H), 6.31 (s, 2H), 4.30 (q, J = 7.1 Hz, 2H), 1.33 (t, J = 7.1 Hz, 3H); **^{13}C NMR** (100 MHz, Chloroform-*d*) δ [ppm] 164.1, 136.1, 132.5, 130.6, 128.3, 123.7, 61.4, 14.3; **HRMS** (ESI) 292.9891, theoretical value for $C_{10}H_{11}BrN_2O_2$ $[M+Na]^+$ 292.9902.

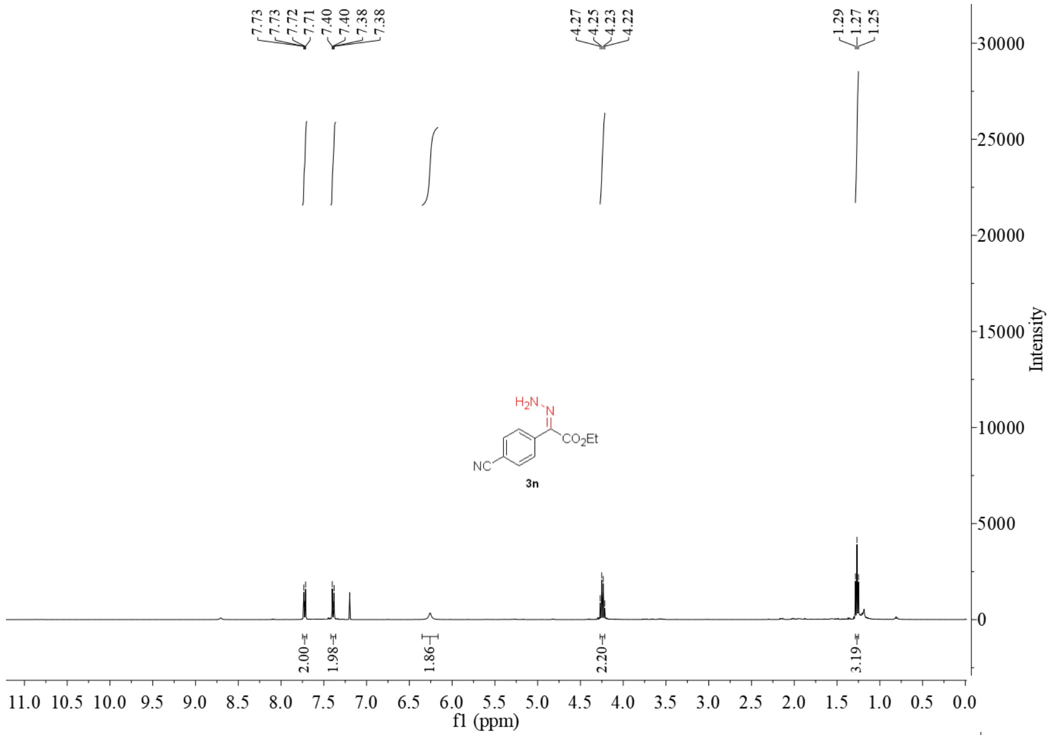

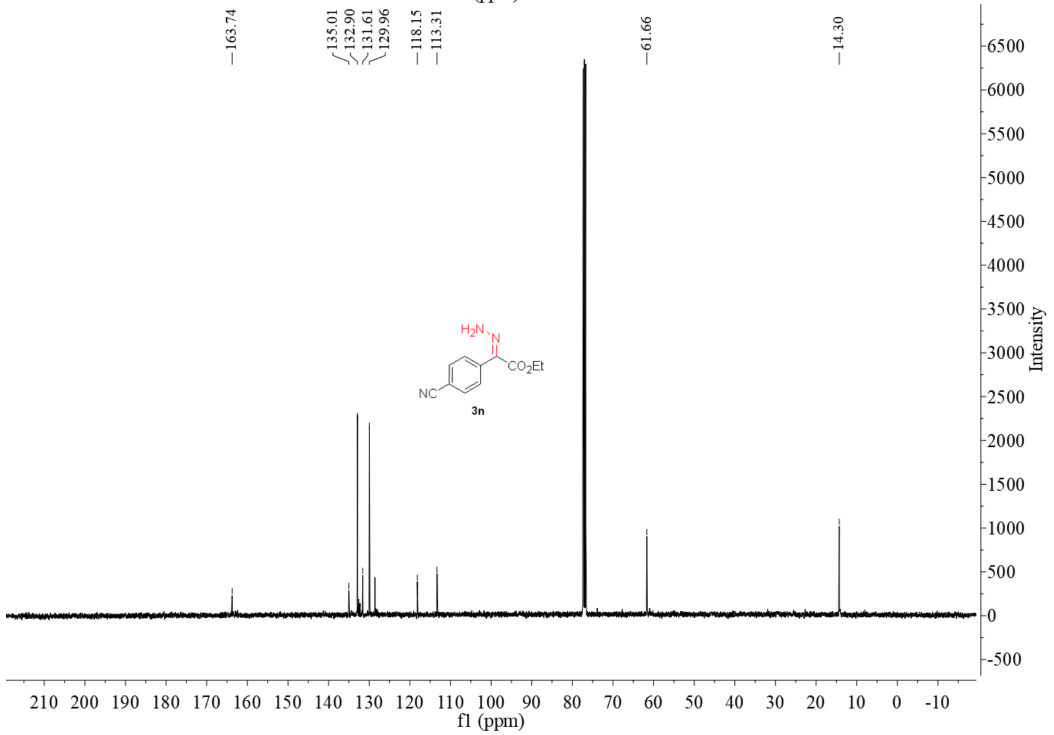

^{1}H, ^{13}C-NMR spectra of product 3n. **^{1}H NMR** (400 MHz, Chloroform-*d*) δ [ppm] 7.75 – 7.70 (m, 2H), 7.42 – 7.36 (m, 2H), 6.26 (s, 2H), 4.24 (q, $J = 7.1$ Hz, 2H), 1.28 (d, $J = 7.1$ Hz, 3H); **^{13}C NMR** (100 MHz, Chloroform-*d*) δ [ppm] 163.7, 135.0, 132.9, 131.6, 130.0, 118.2, 113.3, 61.7, 14.3; **HRMS** (ESI) 216.0777, theoretical value for $C_{11}H_{11}N_3O_2$ $[M-H]^-$ 216.0773.

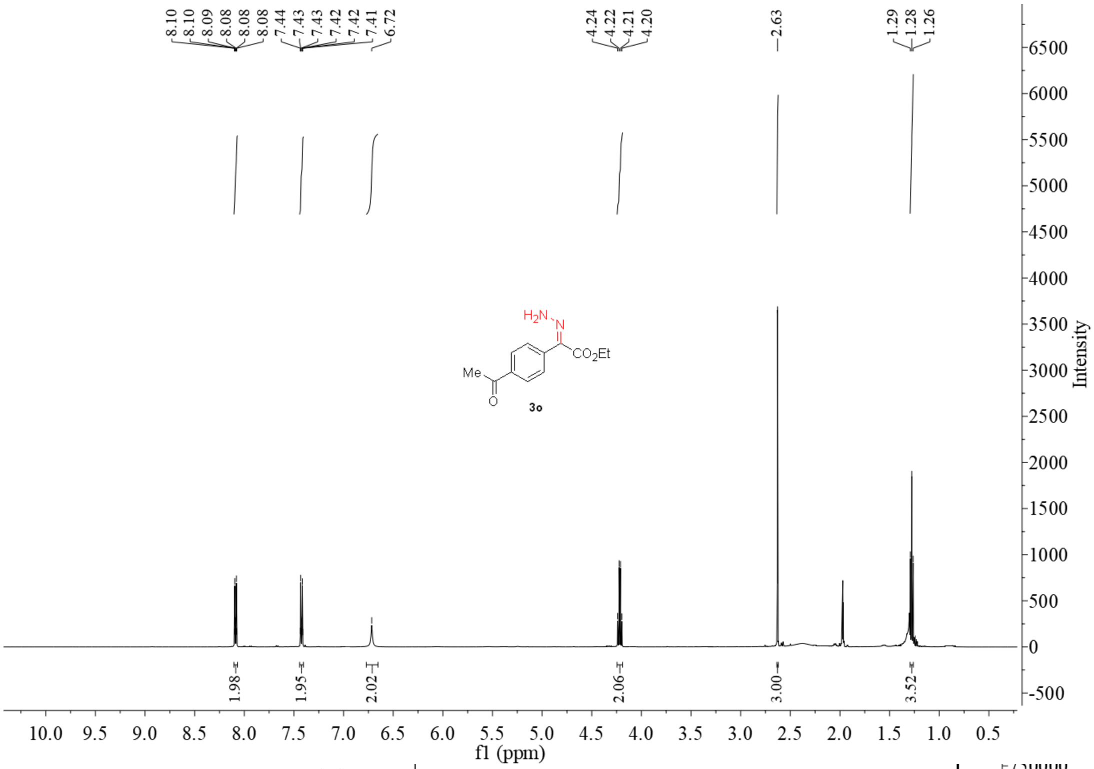

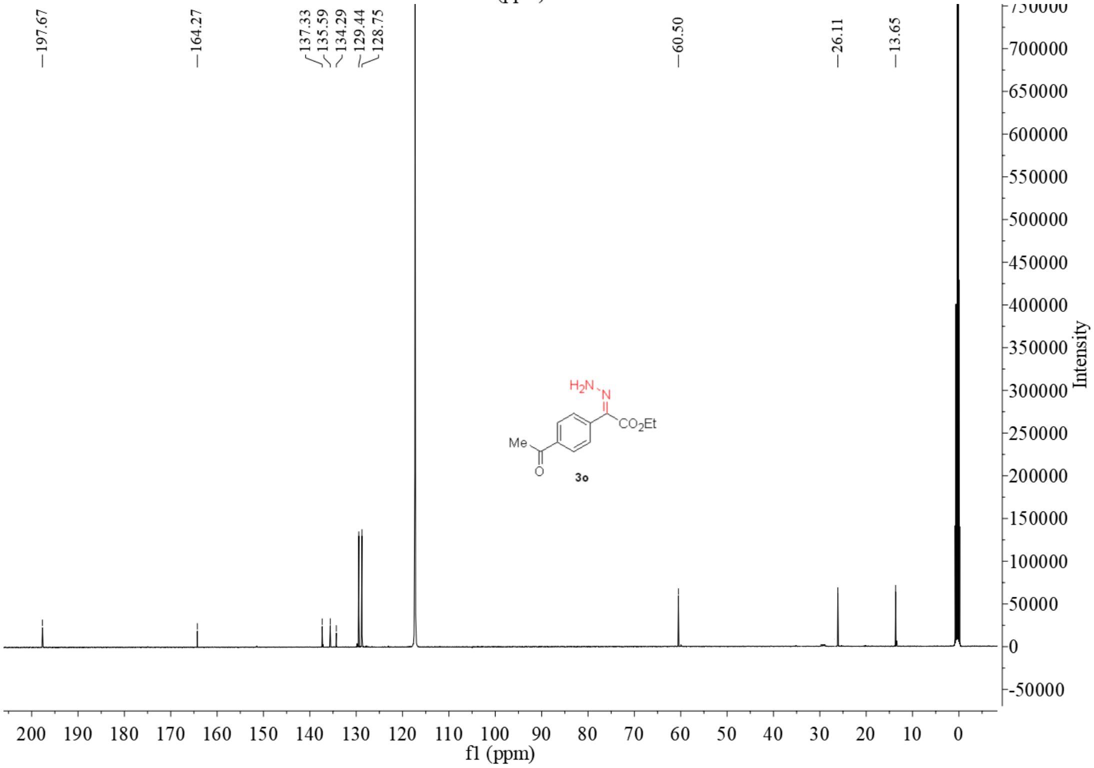

¹H, ¹³C-NMR spectra of product 3o. **¹H NMR** (500 MHz, Acetonitrile-*d*3) *δ* [ppm] 8.10 – 8.07 (m, 2H), 7.44 – 7.40 (m, 2H), 6.72 (s, 2H), 4.22 (q, *J* = 7.1 Hz, 2H), 2.63 (s, 3H), 1.28 (t, *J* = 7.1 Hz, 3H); **¹³C NMR** (125 MHz, Acetonitrile-*d*3) *δ* [ppm] 197.7, 164.3, 137.3, 135.6, 134.3, 129.4, 128.8, 60.5, 26.1, 13.6; **HRMS** (ESI) 257.0898, theoretical value for $C_{12}H_{14}N_2O_3$ $[M+Na]^+$ 257.0902.

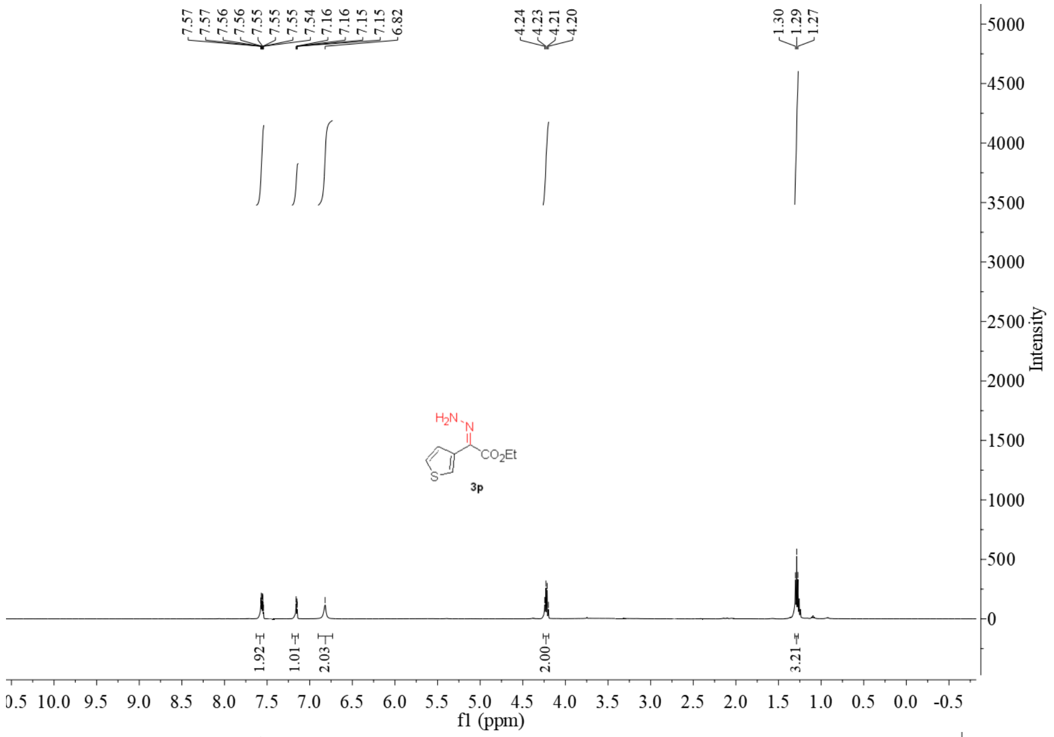

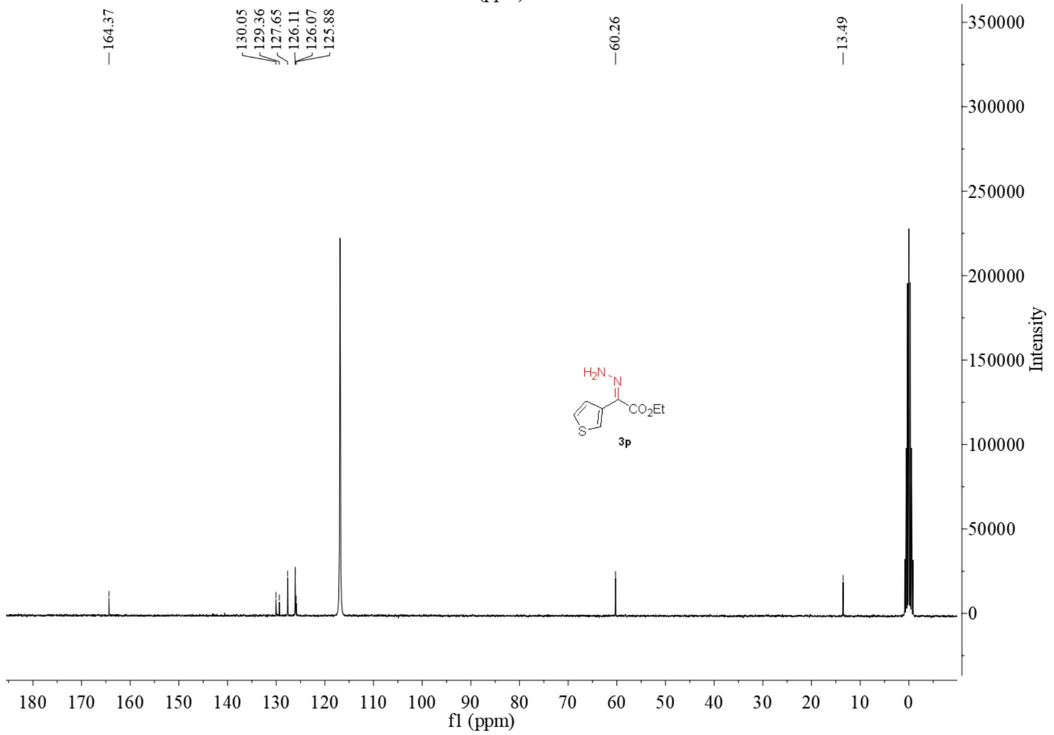

^{1}H, ^{13}C-NMR spectra of product 3p. **^{1}H NMR** (500 MHz, Acetonitrile-d_3) δ [ppm] 7.63 – 7.54 (m, 2H), 7.15 (dd, J = 4.9, 1.3 Hz, 1H), 6.82 (s, 2H), 4.22 (q, J = 7.1 Hz, 2H), 1.29 (t, J = 7.1 Hz, 3H); **^{13}C NMR** (125 MHz, Acetonitrile-d_3) δ [ppm] 164.4, 130.0, 129.4, 127.6, 126.1, 126.1, 125.9, 60.3, 13.5; **HRMS** (ESI) 221.0358, theoretical value for $C_8H_{10}N_2O_2S$ $[M+Na]^+$ 221.0361.

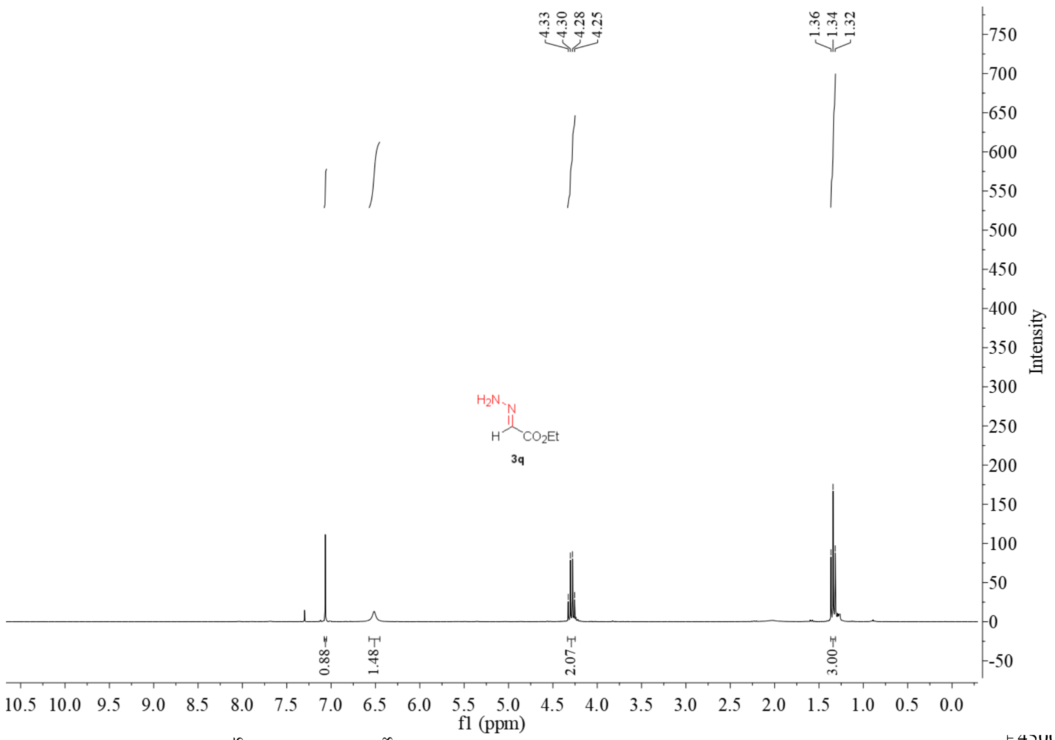

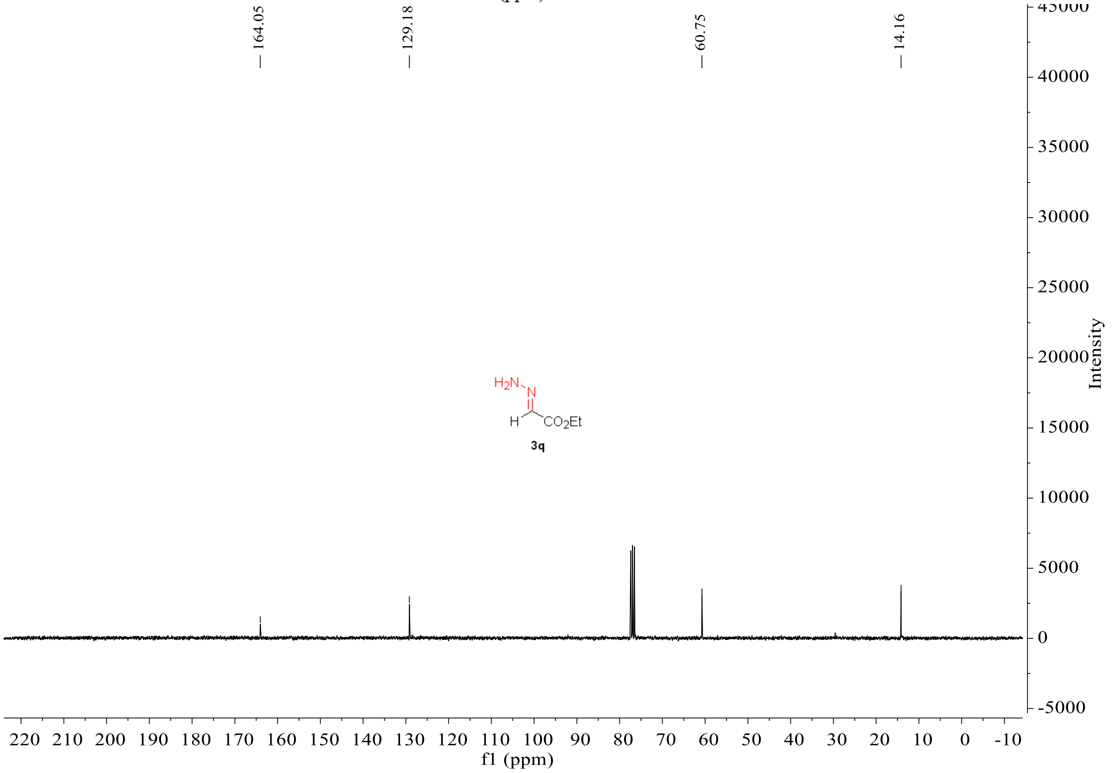

^{1}H, ^{13}C-NMR spectra of product 3q. ^{1}H NMR (300 MHz, Chloroform-*d*) δ [ppm] 7.07 (s, 1H), 6.52 (s, 2H), 4.29 (q, J = 7.1 Hz, 2H), 1.34 (t, J = 7.1 Hz, 3H); **^{13}C NMR** (75 MHz, Acetonitrile-*d*$_3$) δ [ppm] 164.1, 129.2, 60.8, 14.2; **HRMS** (ESI) 115.0514, theoretical value for $C_4H_8N_2O_2$ [M-H]$^-$ 115.0508.

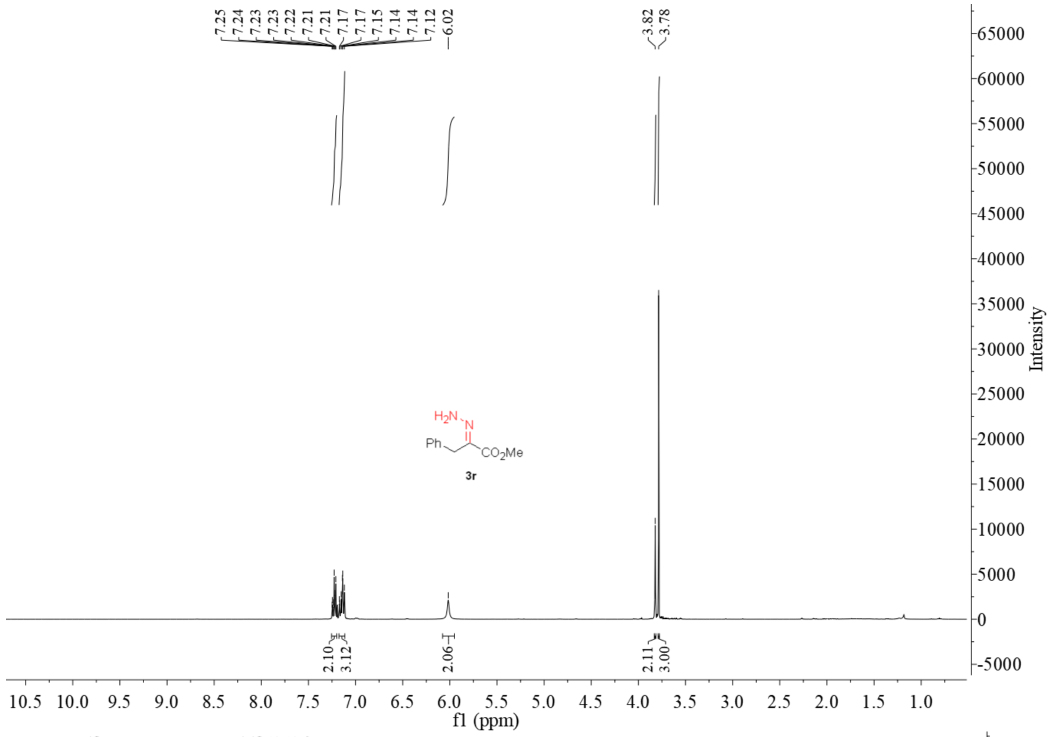

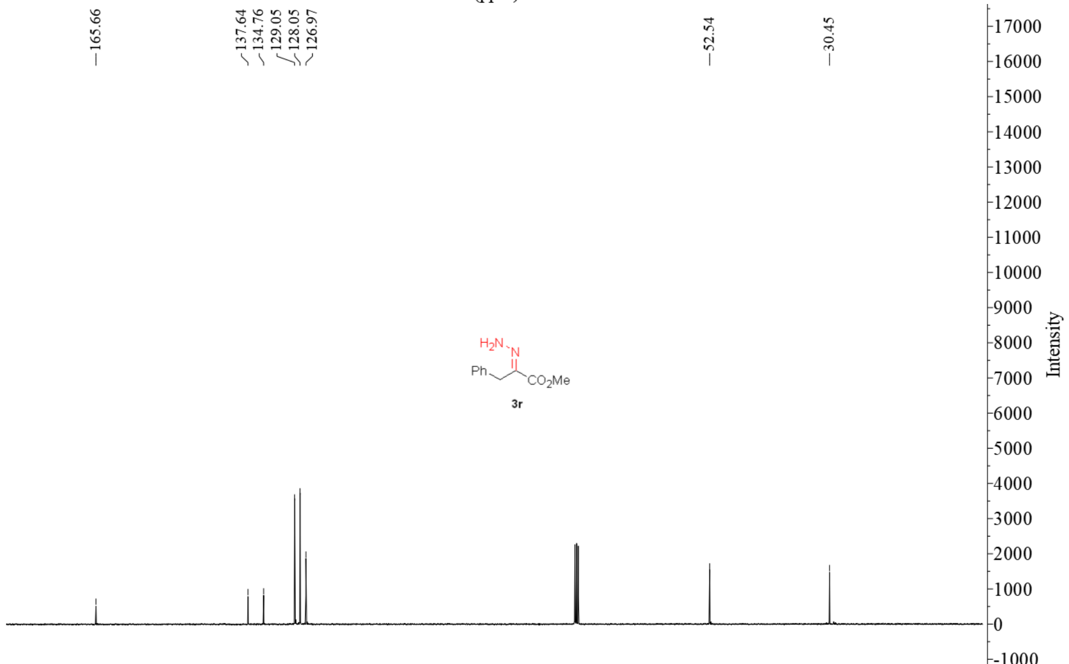

^{1}H, ^{13}C-NMR spectra of product 3r. ^{1}H NMR (400 MHz, Chloroform-*d*) δ 7.23 (ddd, *J* = 7.5, 6.2, 1.3 Hz, 2H), 7.17 – 7.11 (m, 3H), 6.02 (s, 2H), 3.82 (s, 2H), 3.78 (s, 3H); **^{13}C NMR** (75 MHz, Chloroform-*d*) *δ* [ppm] 165.7, 137.6, 134.8, 129.0, 128.0, 127.0, 52.5, 30.4; **HRMS** (ESI) 215.0790, theoretical value for $C_4H_8N_2O_2$ $[M+Na]^+$ 215.0797.

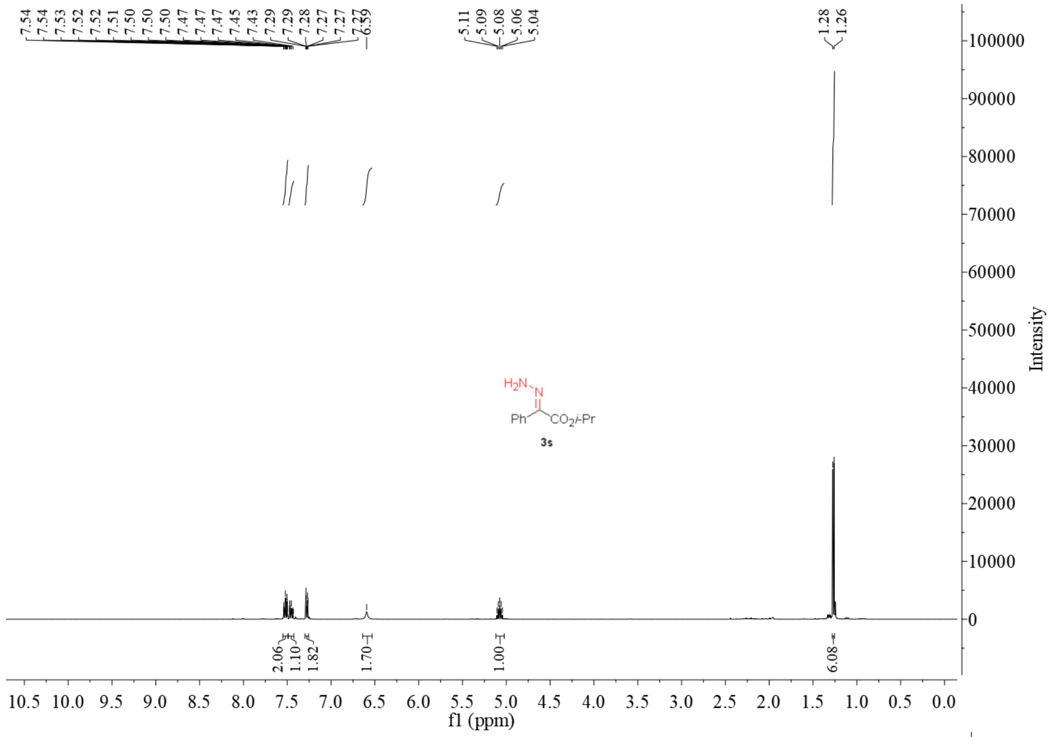

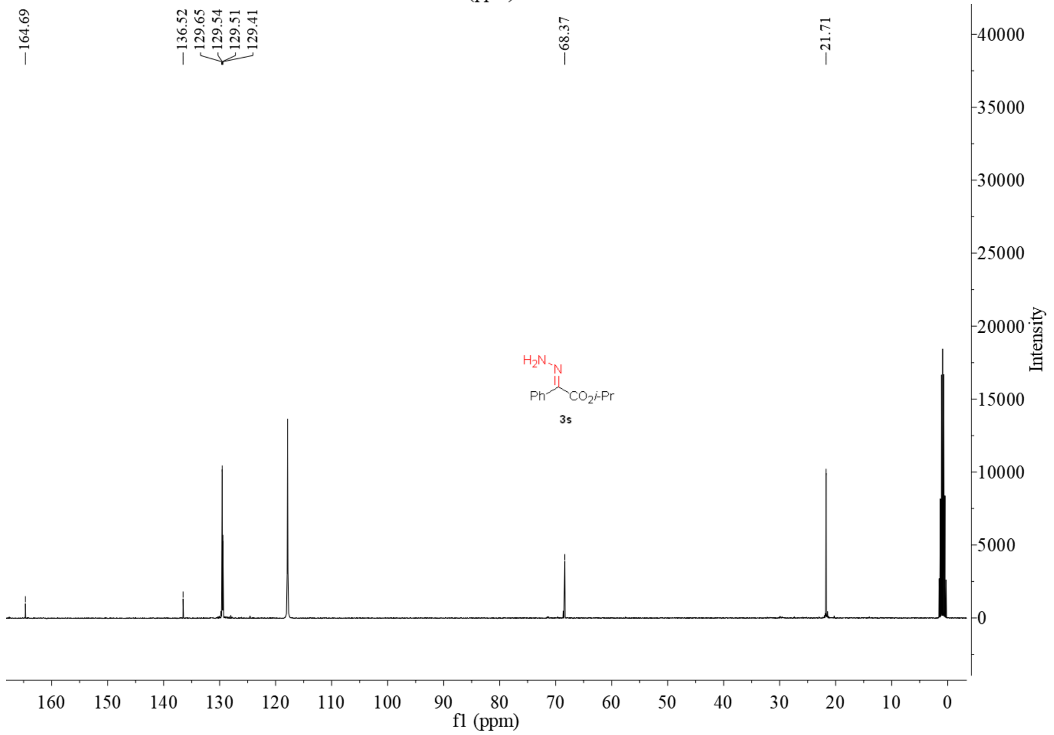

^{1}H, ^{13}C-NMR spectra of product 3s. **^{1}H NMR** (400 MHz, Acetonitrile-*d*3) δ [ppm] 7.55 – 7.49 (m, 2H), 7.49 – 7.42 (m, 1H), 7.30 – 7.26 (m, 2H), 6.59 (s, 2H), 5.08 (p, J = 6.3 Hz, 1H), 1.27 (d, J = 6.3 Hz, 6H); **^{13}C NMR** (100 MHz, Acetonitrile-*d*3) δ [ppm] 164.7, 136.5, 129.6, 129.5, 129.5, 129.4, 68.4, 21.7; **HRMS** (ESI) 229.0954, theoretical value for $C_{11}H_{14}N_2O_2$ $[M+Na]^+$ 229.0953.

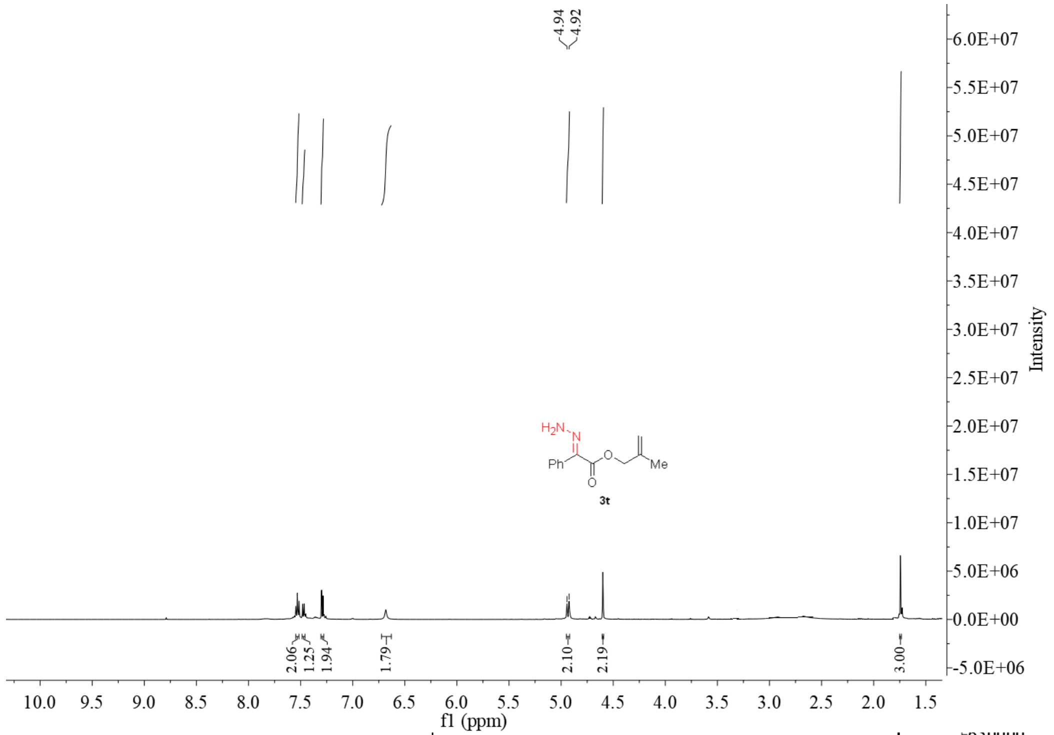

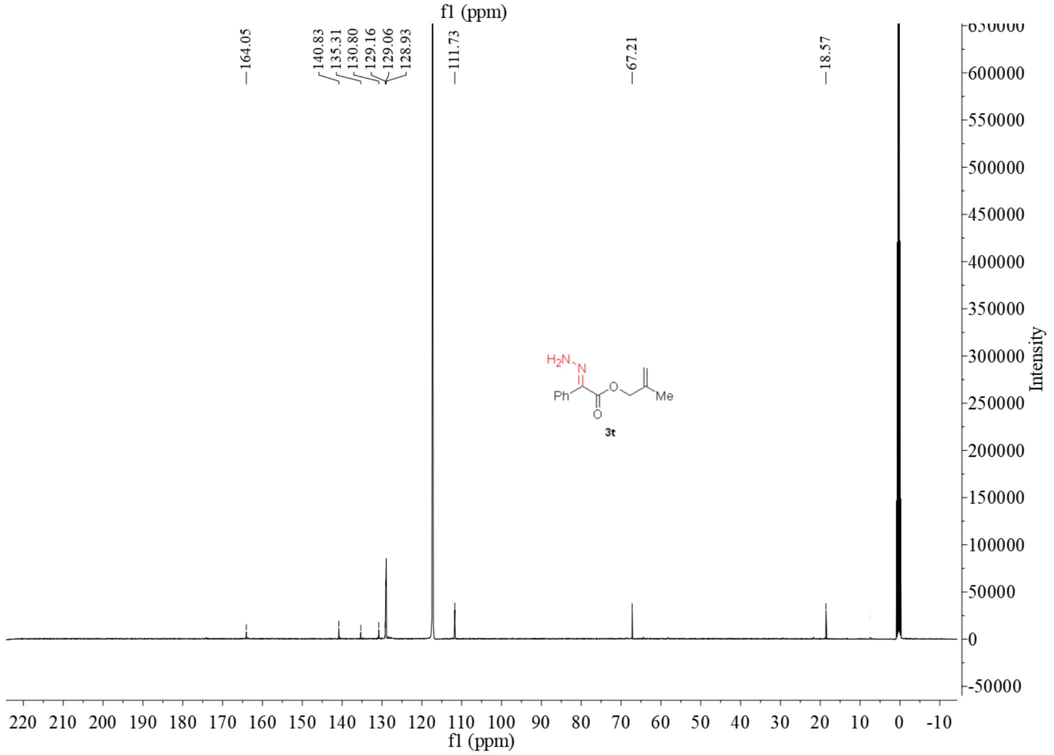

^{1}H, ^{13}C-NMR spectra of product 3t. **^{1}H NMR** (500 MHz, Acetonitrile-d_3) δ [ppm] 7.55 – 7.52 (m, 2H), 7.47 (d, J = 7.9 Hz, 1H), 7.29 (d, J = 7.3 Hz, 2H), 6.68 (s, 2H), 4.93 (d, J = 10.1 Hz, 2H), 4.60 (s, 2H), 1.74 (s, 3H); **^{13}C NMR** (125 MHz, Acetonitrile-d_3) δ [ppm] 164.0, 140.8, 135.3, 130.8, 129.1, 129.1, 128.9, 111.7, 67.2, 18.6; **HRMS** (ESI) 241.0949, theoretical value for $C_{11}H_{14}N_2O_2$ $[M+Na]^+$ 241.0953.

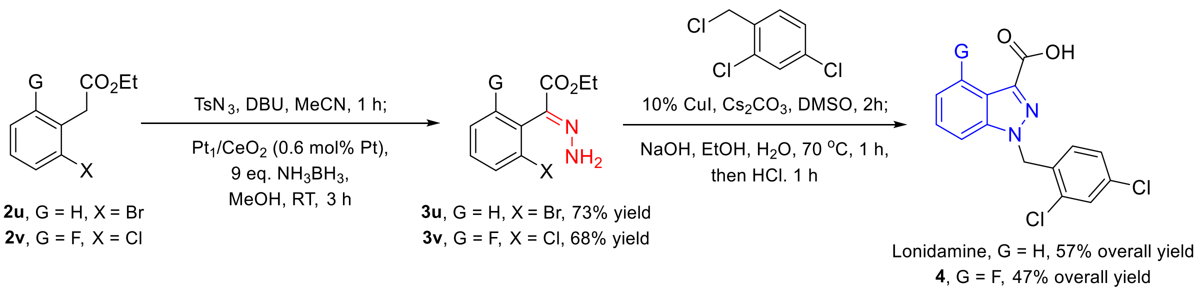

2u, G = H, X = Br
2v, G = F, X = Cl

3u, G = H, X = Br, 73% yield
3v, G = F, X = Cl, 68% yield

Lonidamine, G = H, 57% overall yield
4, G = F, 47% overall yield

Total synthesis of *Lonidamine* and 4.

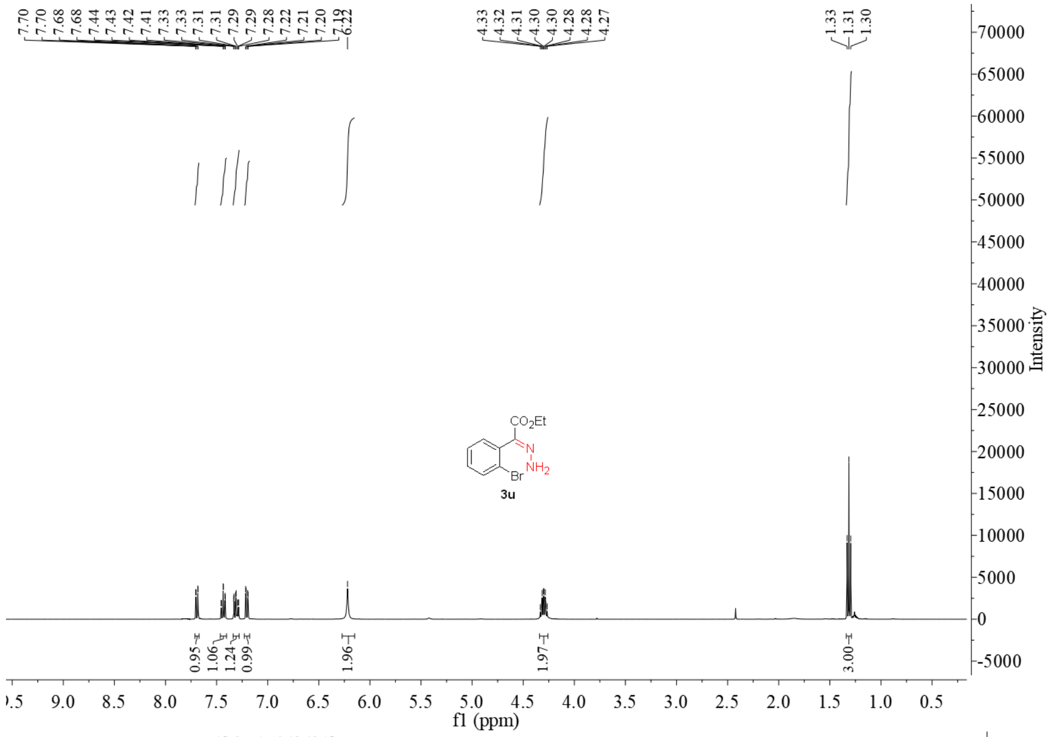

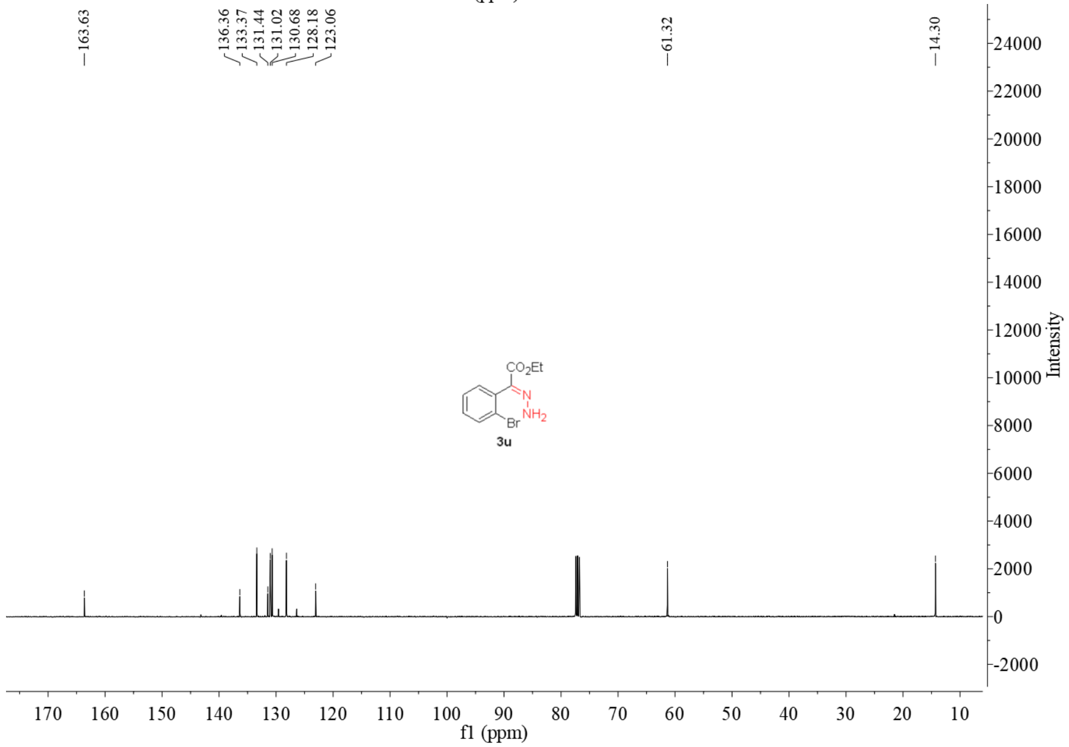

^{1}H, ^{13}C-NMR spectra of product 3u. **^{1}H NMR** (400 MHz, Chloroform-*d*) δ [ppm] 7.69 (dd, J = 8.1, 1.1 Hz, 1H), 7.44 (td, J = 7.5, 1.2 Hz, 1H), 7.34 – 7.28 (m, 1H), 7.20 (dd, J = 7.6, 1.7 Hz, 1H), 6.22 (s, 2H), 4.30 (qd, J = 7.1, 5.1 Hz, 2H), 1.31 (t, J = 7.1 Hz, 3H); **^{13}C NMR** (100 MHz, Chloroform-*d*) δ [ppm] 163.6, 136.4, 133.4, 131.4, 131.0, 130.7, 128.2, 123.1, 61.3, 14.3; **HRMS** (ESI) 292.9892, theoretical value for $C_{10}H_{11}BrN_2O_2$ $[M+Na]^+$ 292.9902.

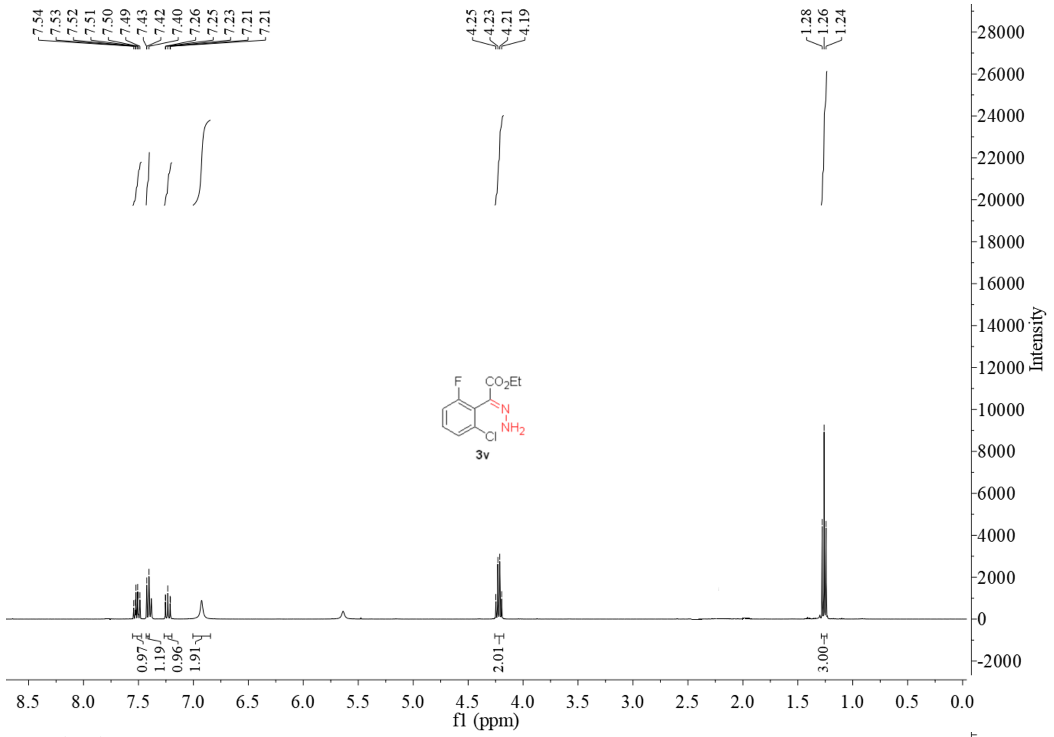

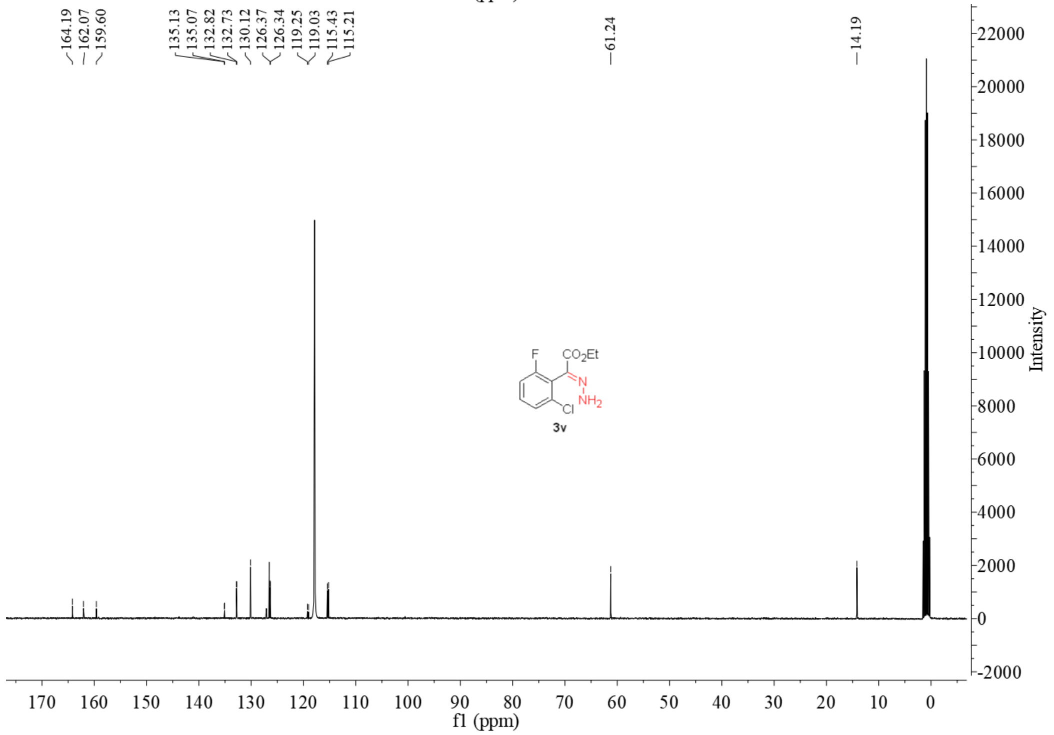

^{1}H, ^{13}C-NMR spectra of product 3v. **^{1}H NMR** (400 MHz, Acetonitrile-*d*$_3$) δ [ppm] 7.52 (td, J = 8.3, 6.1 Hz, 1H), 7.43 – 7.40 (m, 1H), 7.27 – 7.19 (m, 1H), 6.93 (s, 2H), 4.22 (q, J = 7.4 Hz, 2H), 1.26 (t, J = 7.1 Hz, 3H); **^{13}C NMR** (100 MHz, Acetonitrile-*d*$_3$) δ [ppm] 164.2, 160.3 (d, $J^1{}_F$ = 247 Hz), 135.1 (d, $J^3{}_F$ = 6 Hz), 132.8 (d, $J^3{}_F$ = 9 Hz), 130.1, 126.3 (d, $J^4{}_F$ = 2 Hz), 119.2 (d, $J^2{}_F$ = 22 Hz), 115.3 (d, $J^2{}_F$ = 22 Hz), 61.2, 14.2; **HRMS** (ESI) 267.0304, theoretical value for $C_{10}H_{10}ClFN_2O_2$ [M+Na]$^+$ 267.0313.

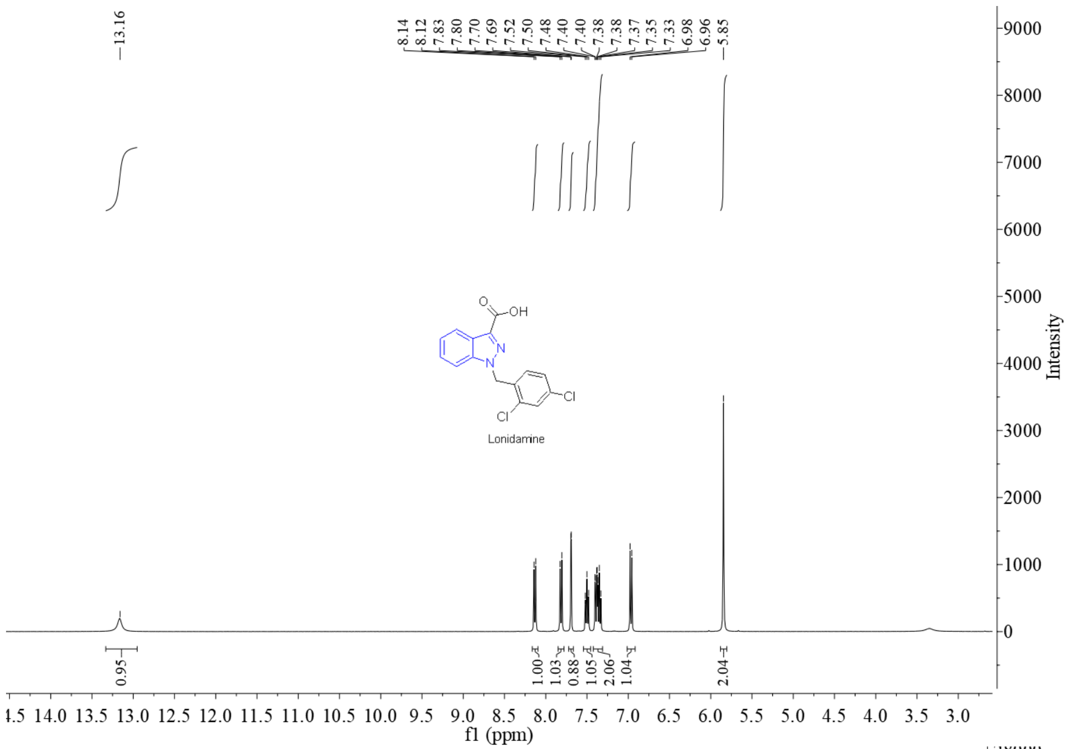

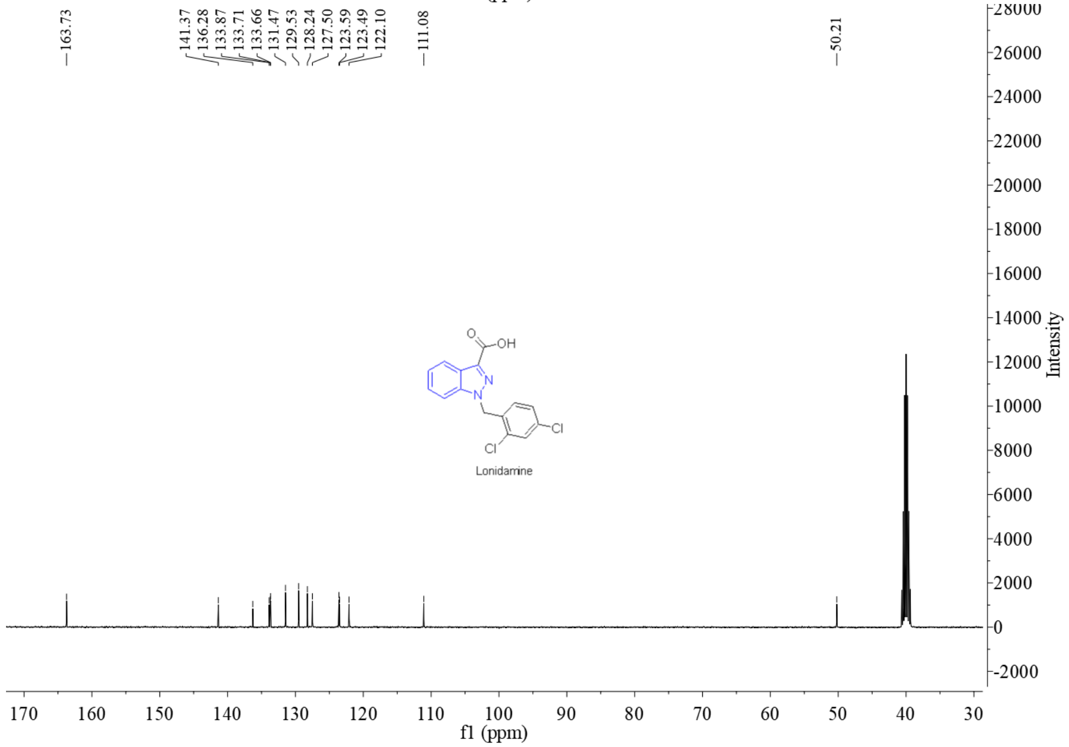

^{1}H, ^{13}C-NMR spectra of product *Lonidamine*. **^{1}H NMR** (400 MHz, DMSO-d_6) δ [ppm] 13.16 (s, 1H), 8.13 (d, J = 8.2 Hz, 1H), 7.81 (d, J = 8.5 Hz, 1H), 7.69 (d, J = 2.1 Hz, 1H), 7.50 (t, J = 8.0 Hz, 1H), 7.42 – 7.31 (m, 2H), 6.97 (d, J = 8.4 Hz, 1H), 5.85 (s, 2H); **^{13}C NMR** (100 MHz, DMSO-d_6) δ [ppm] 163.7, 141.4, 136.3, 133.9, 133.7, 133.7, 131.5, 129.5, 128.2, 127.5, 123.6, 123.5, 122.1, 111.1, 50.2; **HRMS** (ESI) 343.0010, theoretical value for $C_{15}H_{10}Cl_2N_2O_2$ $[M+Na]^+$ 343.0017.

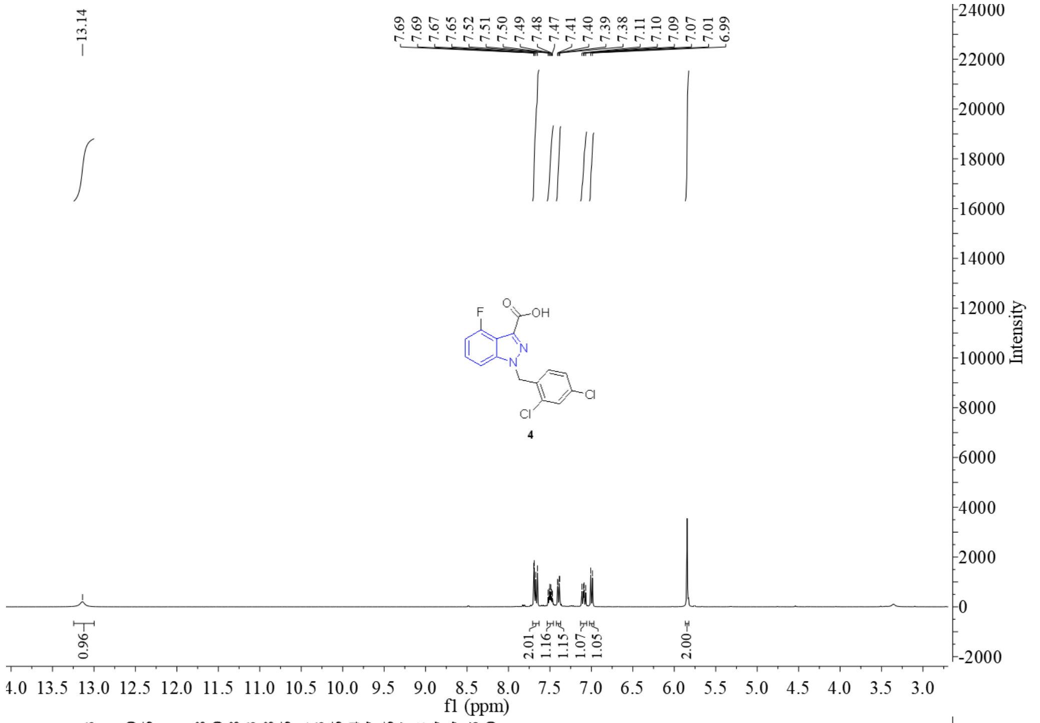

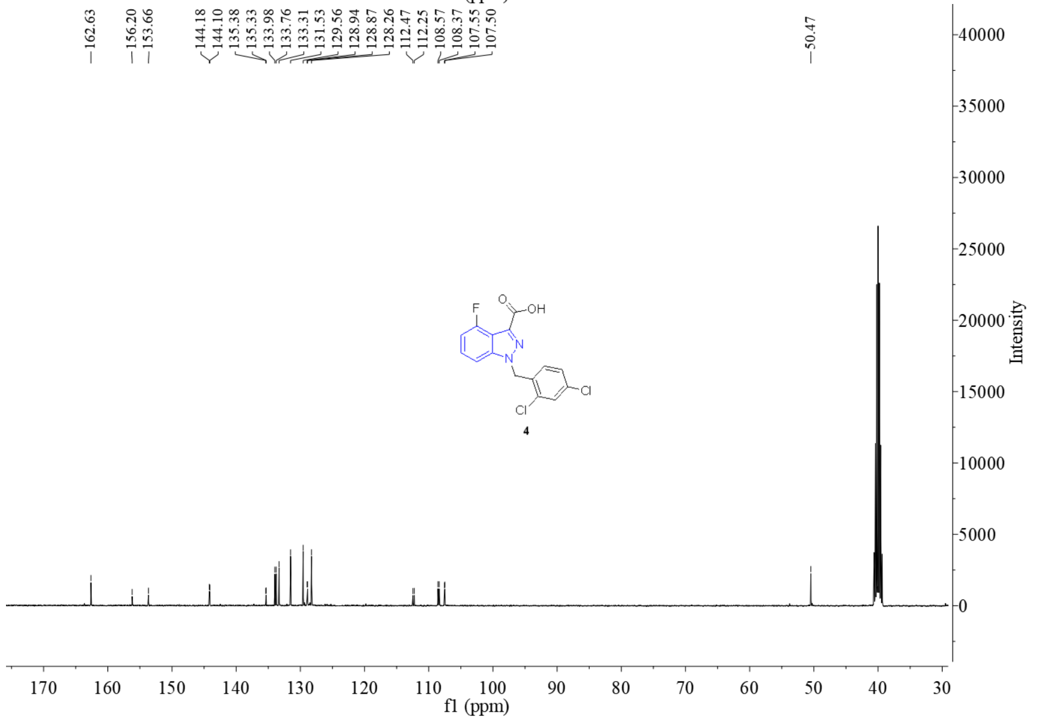

^{1}H, ^{13}C-NMR spectra of F-containing *Lonidamine* 4. **^{1}H NMR** (400 MHz, DMSO-d_6) δ [ppm] 13.14 (s, 1H), 7.71 – 7.63 (m, 2H), 7.50 (td, J = 8.1, 4.5 Hz, 1H), 7.40 (dd, J = 8.3, 2.0 Hz, 1H), 7.09 (dd, J = 10.9, 7.7 Hz, 1H), 7.00 (d, J = 8.4 Hz, 1H), 5.85 (s, 2H); **^{3}C NMR** (100 MHz, DMSO-d_6) δ [ppm] 162.6, 155.0 (d, $J^{1}{}_{F}$ = 254 Hz), 144.1 (d, $J^{3}{}_{F}$ = 8 Hz), 135.4 (d, $J^{4}{}_{F}$ = 5 Hz), 134.0, 134.8, 133.3, 131.5, 129.6, 128.9 (d, $J^{3}{}_{F}$ = 7 Hz), 128.3, 112.3 (d, $J^{2}{}_{F}$ = 22 Hz), 108.4 (d, $J^{2}{}_{F}$ = 20 Hz), 107.5 (d, $J^{4}{}_{F}$ = 5 Hz), 50.5; **HRMS** (ESI) 339.0098, theoretical value for $C_{15}H_9Cl_2FN_2O_2$ $[M+H]^+$ 339.0103.

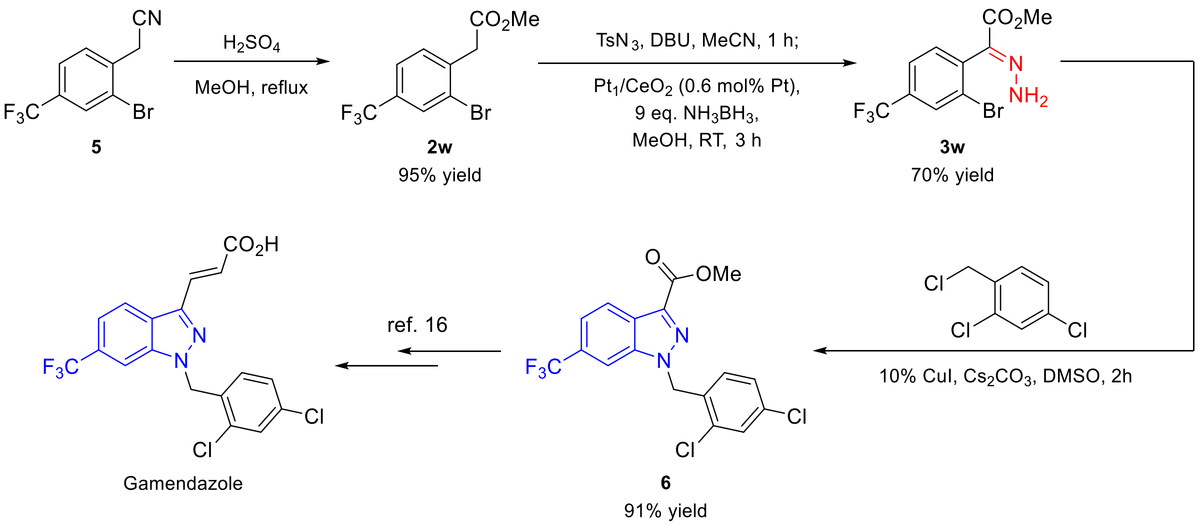

The synthesis of key intermediate 6 for *Gamendazole*

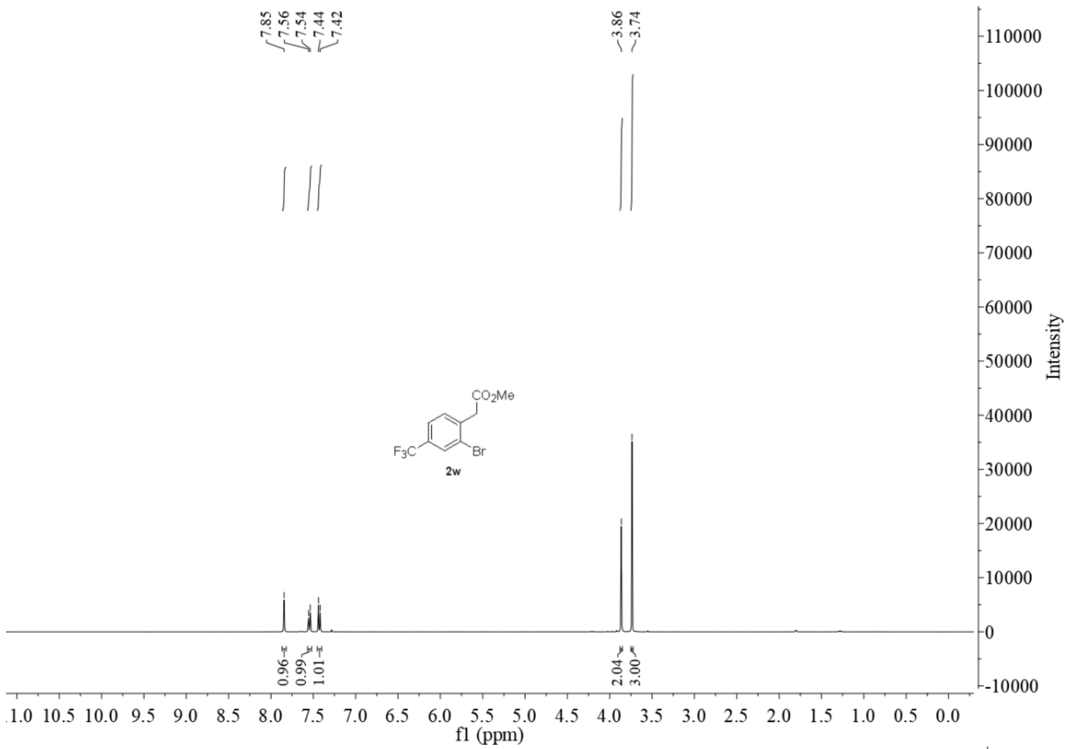

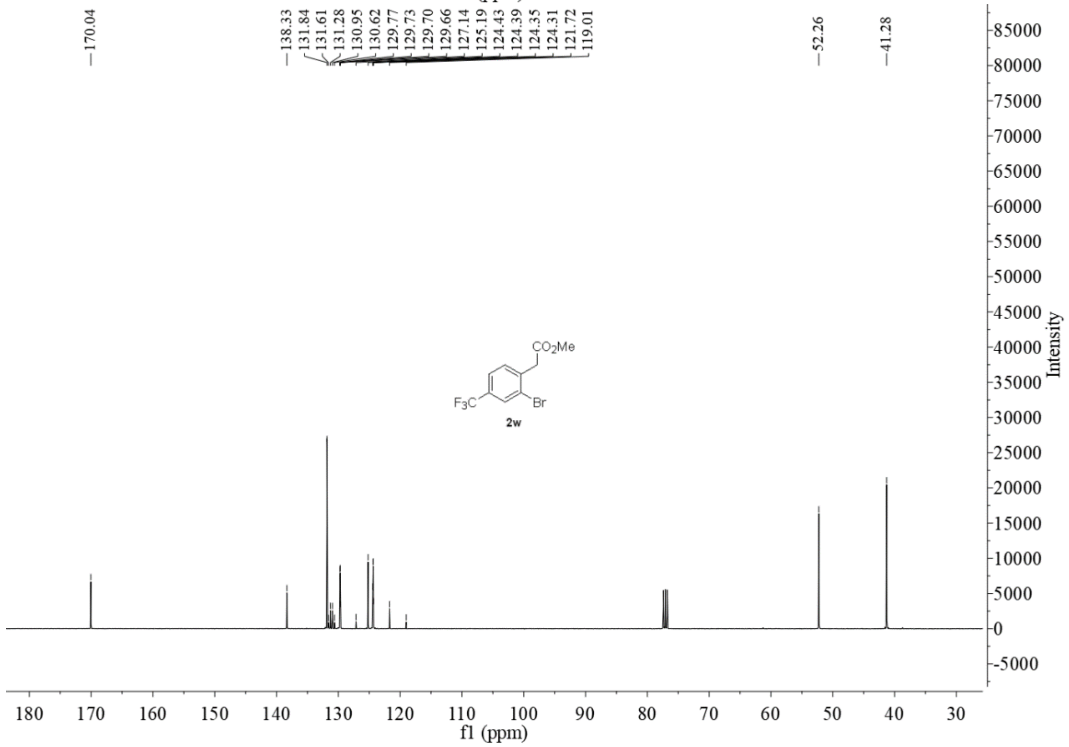

^{1}H, ^{13}C-NMR spectra of product 2w. ^{1}H NMR (400 MHz, Chloroform-*d*) δ [ppm] 7.85 (s, 1H), 7.55 (d, J = 8.0 Hz, 1H), 7.43 (d, J = 8.0 Hz, 1H), 3.86 (s, 2H), 3.74 (s, 3H); **^{13}C NMR** (100 MHz, Chloroform-*d*) δ [ppm] 170.0, 138.3, 131.8, 131.1 (q, $J^1{}_F$ = 330 Hz), 129.7 (q, $J^2{}_F$ = 7 Hz), 125.2, 124.4 (q, $J^2{}_F$ = 4 Hz), 121.7, 52.3, 41.3; **HRMS** (APCI) 296.9727, theoretical value for $C_{10}H_8BrF_3O_2$ $[M+H]^+$ 296.9738.

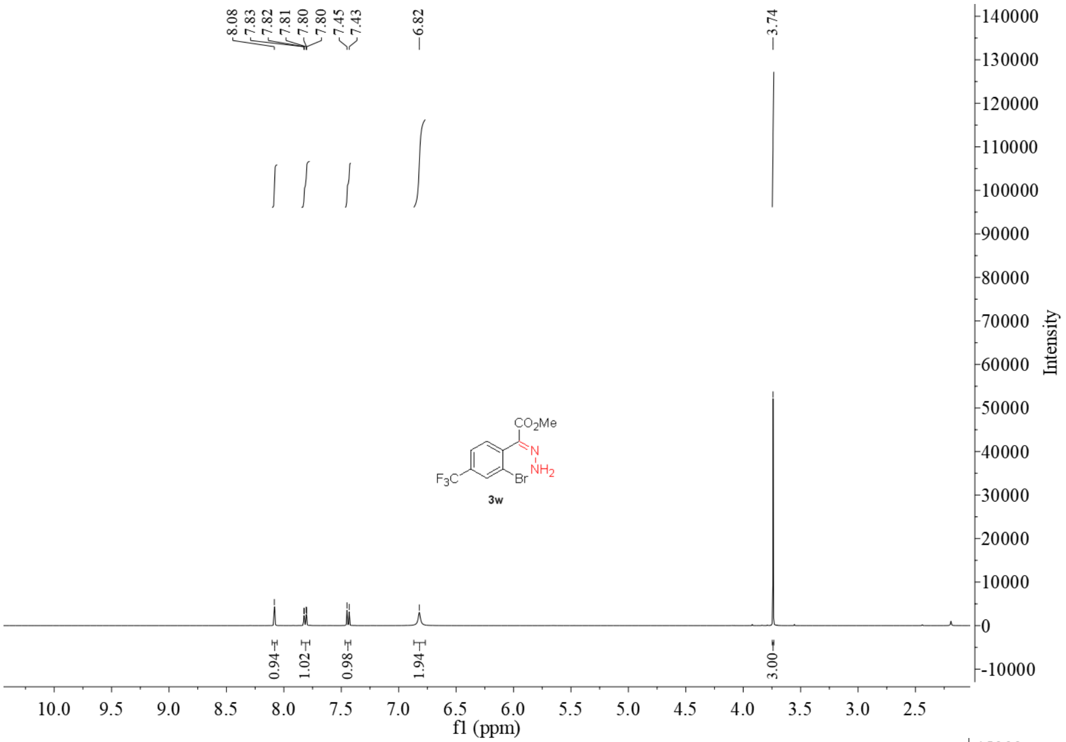

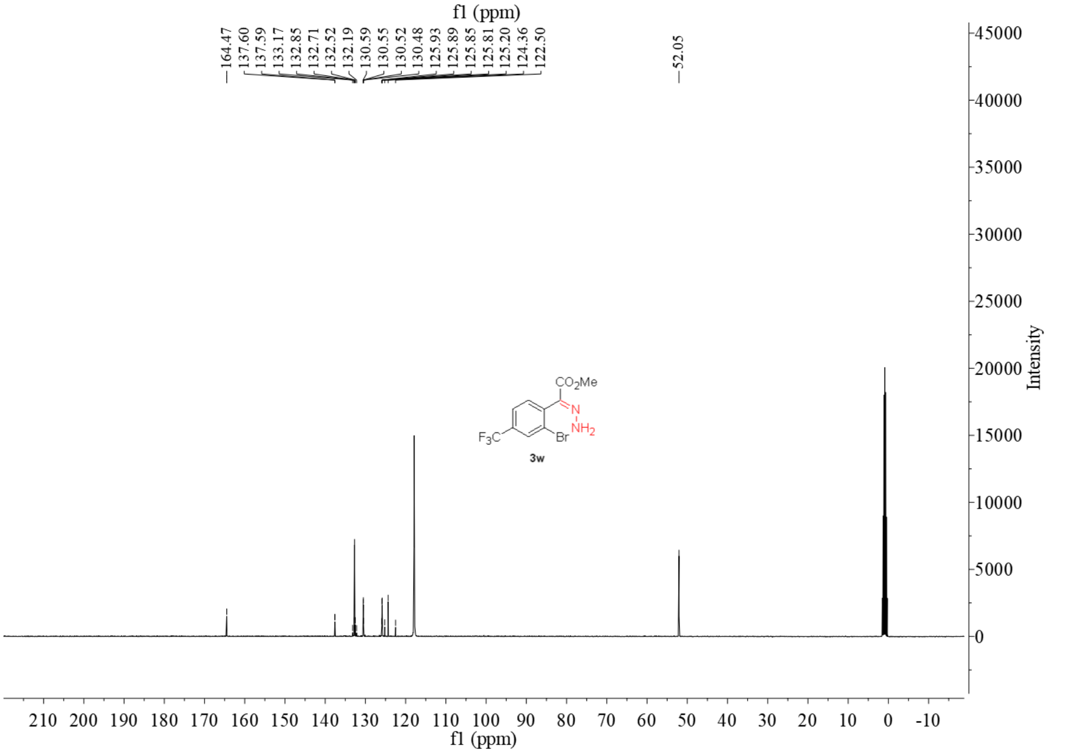

^{1}H, ^{13}C-NMR spectra of product 3w. **^{1}H NMR** (400 MHz, Acetonitrile-d_3) δ [ppm] 8.08 (s, 1H), 7.85 – 7.77 (m, 1H), 7.44 (d, J = 8.0 Hz, 1H), 6.82 (s, 2H), 3.74 (s, 3H); **^{13}C NMR** (100 MHz, Acetonitrile-d_3) δ [ppm] 164.5, 137.6 (q, $J^4{}_F$ = 1 Hz), 132.7 (q, $J^1{}_F$ = 330 Hz), 132.7, 130.5 (q, $J^2{}_F$ = 3 Hz), 125.9 (q, $J^2{}_F$ = 4 Hz), 125.2, 124.4, 122.5, 52.1; **HRMS** (ESI) 346.9614, theoretical value for $C_{10}H_8BrF_3N_2O_2$ $[M+Na]^+$ 346.9619.

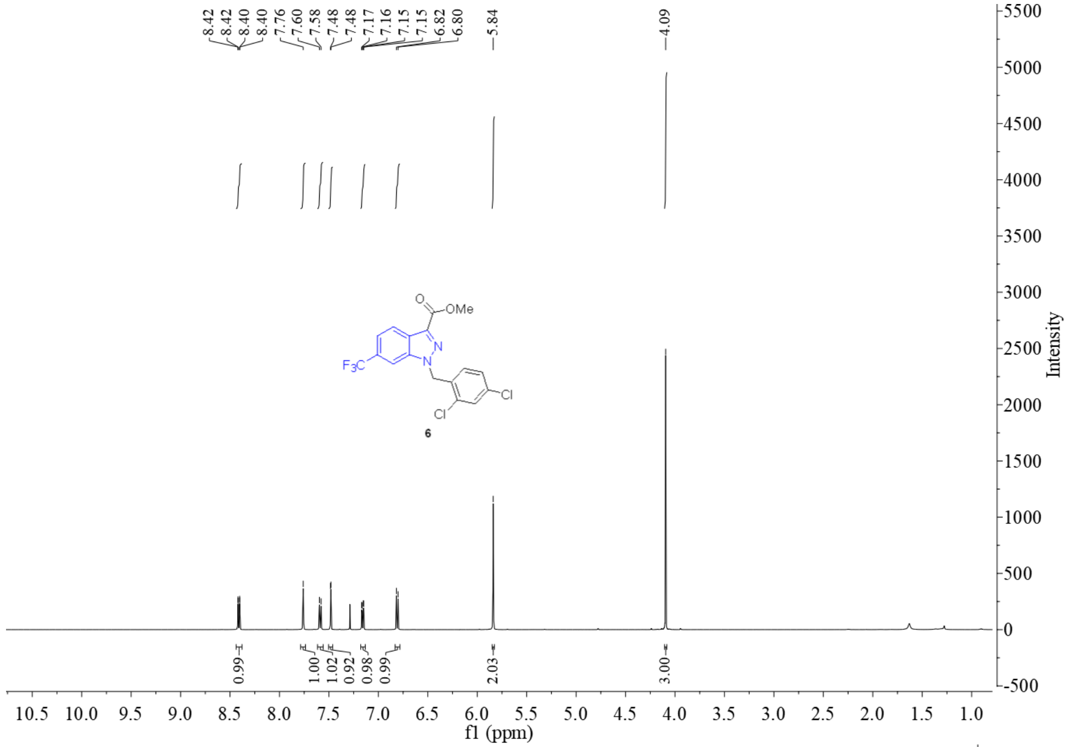

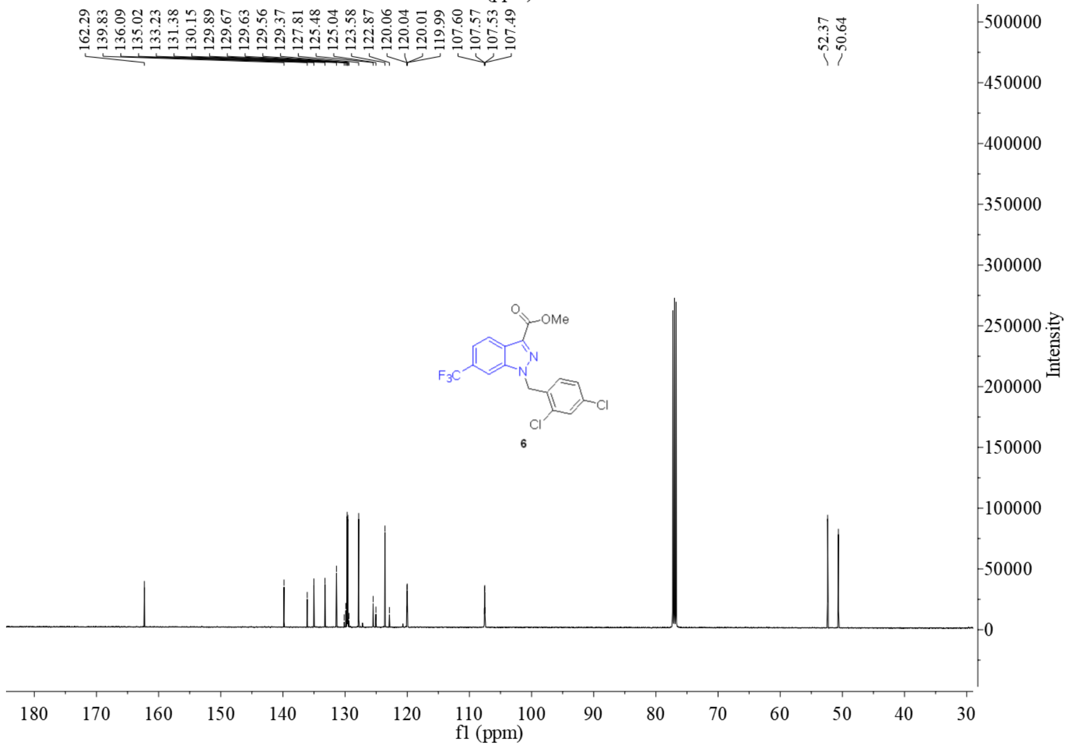

^{1}H, ^{13}C-NMR spectra of product 6. **^{1}H NMR** (500 MHz, Chloroform-*d*) δ [ppm] 8.44 – 8.38 (m, 1H), 7.76 (s, 1H), 7.59 (d, J = 8.6 Hz, 1H), 7.48 (d, J = 2.1 Hz, 1H), 7.16 (dd, J = 8.4, 2.1 Hz, 1H), 6.81 (d, J = 8.4 Hz, 1H), 5.84 (s, 2H), 4.09 (s, 3H); **^{13}C NMR** (125 MHz, Chloroform-*d*) δ [ppm] 162.3, 139.8, 136.1, 135.0, 133.2, 131.4, 129.8 (q, $J^2{}_F$ = 32.5 Hz), 129.7, 129.6, 127.8, 125.5, 124.0 (q, $J^1{}_F$ = 271.3 Hz), 123.6, 120.0 (q, $J^4{}_F$ = 3.75 Hz), 107.5 (q, $J^3{}_F$ = 5.0 Hz), 52.4, 50.6; **HRMS** (ESI) 403.0224, theoretical value for $C_{17}H_{11}Cl_2F_3N_2O_2$ $[M+H]^+$ 403.0228.

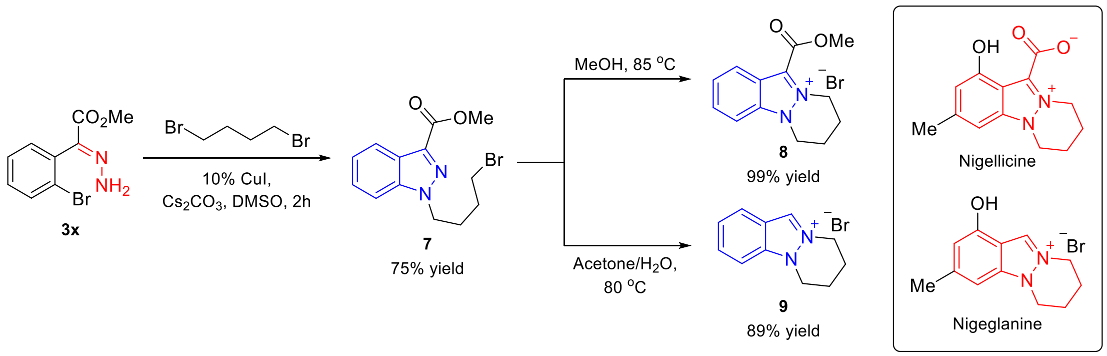

The construction of tricyclic pyridazino[1,2-*a*]indazolium ring frameworks 8 & 9.

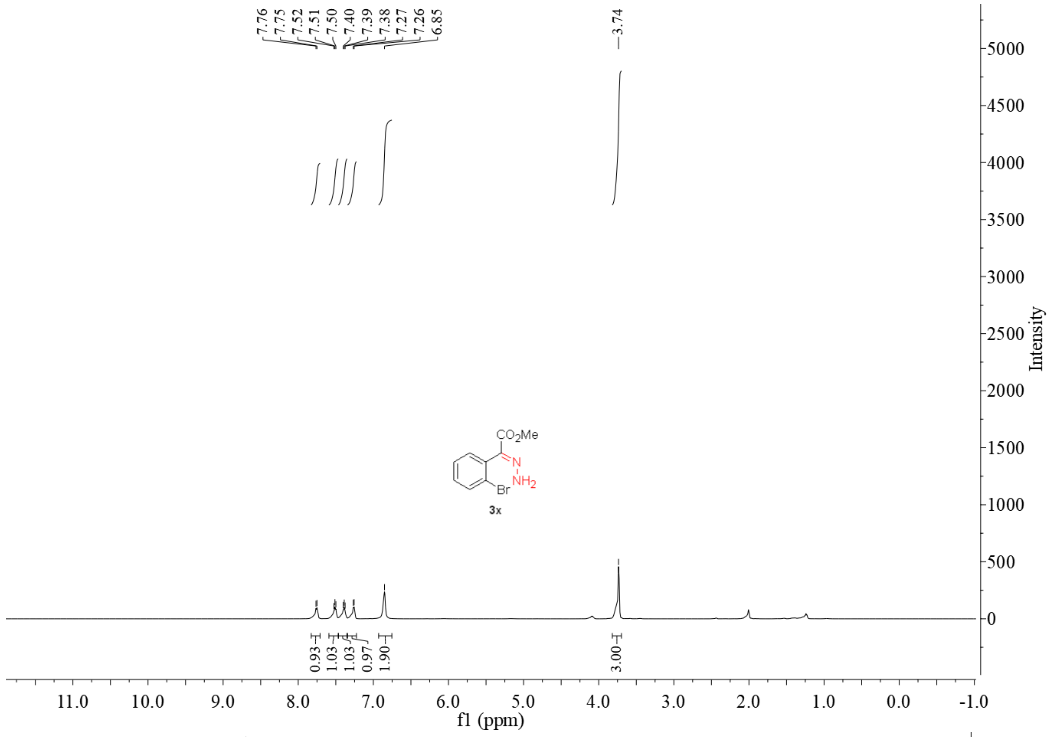

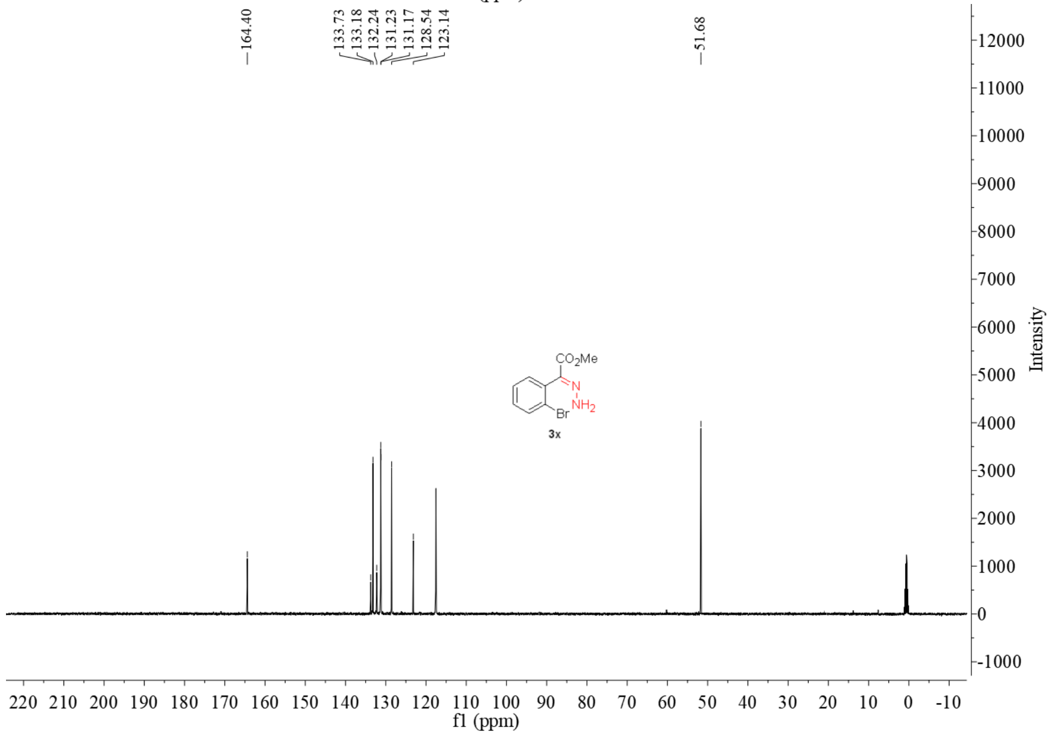

^{1}H, ^{13}C-NMR spectra of product 3x. **^{1}H NMR** (500 MHz, Acetonitrile-d_3) δ [ppm] 7.76 (d, J = 6.6 Hz, 1H), 7.59 – 7.47 (m, 1H), 7.46 – 7.35 (m, 1H), 7.34 – 7.23 (m, 1H), 6.85 (s, 2H), 3.74 (s, 3H); **^{13}C NMR** (125 MHz, Acetonitrile-d_3) δ [ppm] 164.4, 133.7, 133.2, 132.4, 131.2, 131.1, 128.5, 123.1, 51.7; **HRMS** (ESI) 278.9738, theoretical value for $C_9H_9BrN_2O_2$ $[M+Na]^+$ 278.9745.

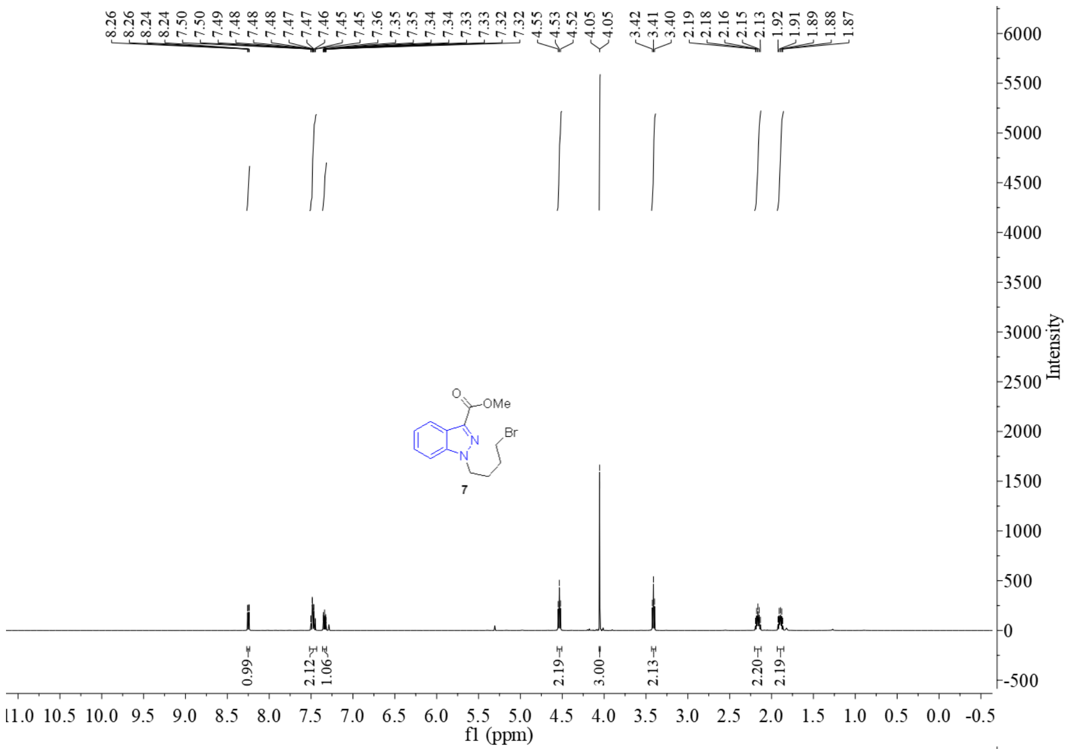

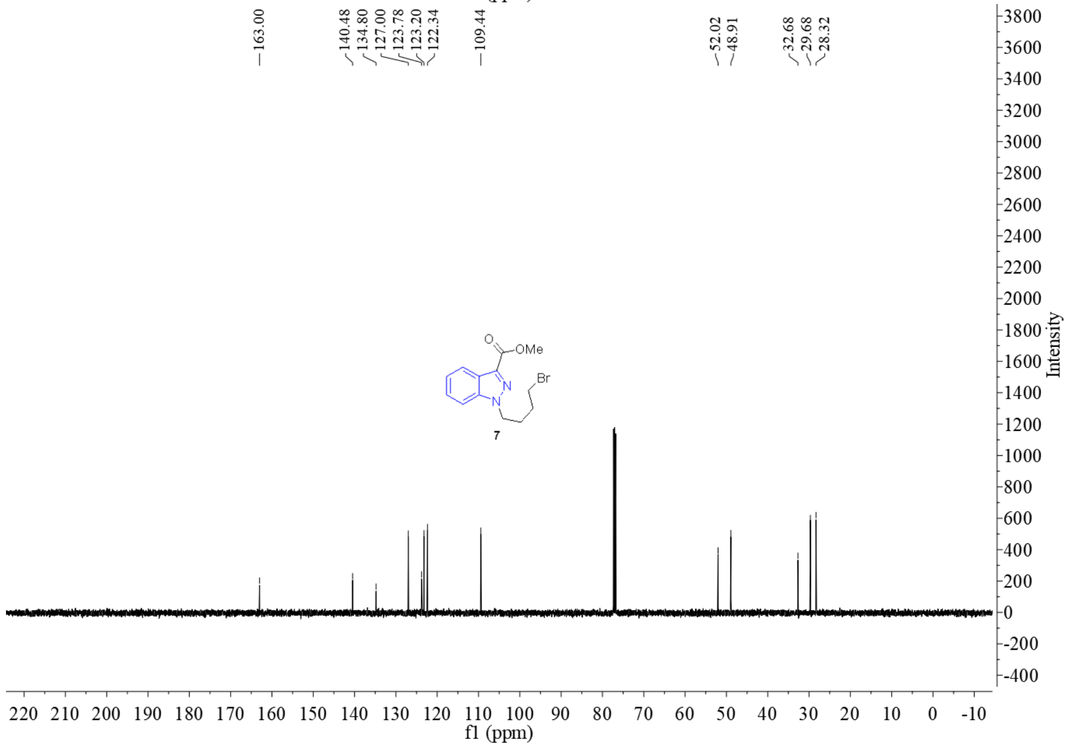

^{1}H, ^{13}C-NMR spectra of product 7. **^{1}H NMR** (500 MHz, Chloroform-*d*) δ [ppm] 8.25 (dd, J = 8.2, 0.9 Hz, 1H), 7.52 – 7.43 (m, 2H), 7.36 – 7.32 (m, 1H), 4.53 (t, J = 7.1 Hz, 2H), 4.05 (d, J = 0.8 Hz, 3H), 3.41 (t, J = 6.5 Hz, 2H), 2.16 (p, J = 7.2 Hz, 2H), 1.89 (dt, J = 13.5, 6.7 Hz, 2H); **^{13}C NMR** (125 MHz, Chloroform-*d*) δ [ppm] 163.0, 140.5, 134.8, 127.0, 123.8, 123.2, 122.3, 109.4, 52.0, 48.9, 32.7, 29.7, 28.3; **HRMS** (ESI) 333.0204, theoretical value for $C_{13}H_{15}BrN_2O_2$ $[M+Na]^+$ 333.0215.

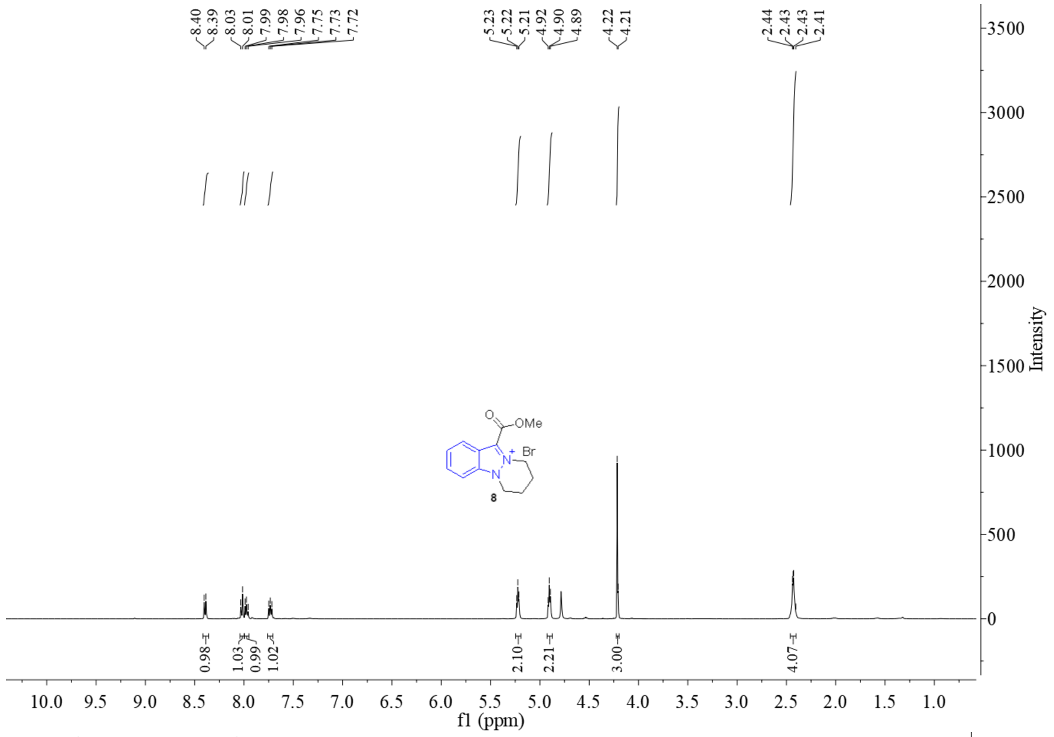

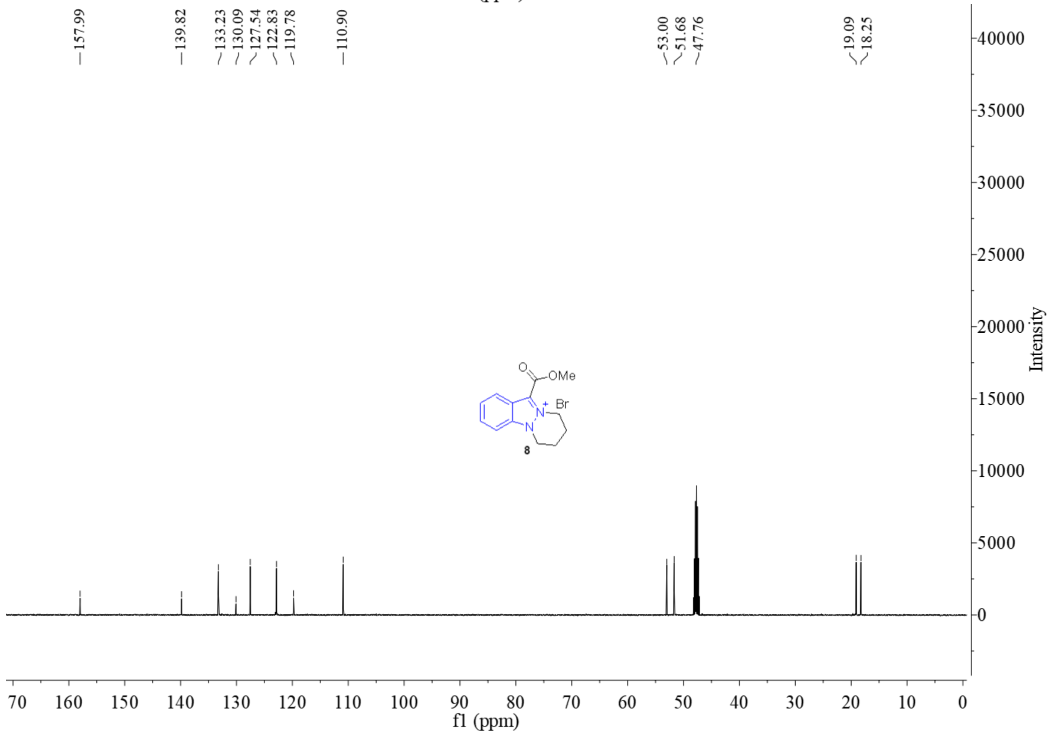

^{1}H, ^{13}C-NMR spectra of product 8. **^{1}H NMR** (500 MHz, Methanol-d_4) δ [ppm] 8.40 (d, J = 8.6 Hz, 1H), 8.02 (d, J = 8.8 Hz, 1H), 8.00 – 7.95 (m, 1H), 7.76 – 7.71 (m, 1H), 5.22 (t, J = 5.6 Hz, 2H), 4.90 (t, J = 5.6 Hz, 2H), 4.21 (d, J = 4.4 Hz, 3H), 2.46 – 2.40 (m, 4H); **^{13}C NMR** (125 MHz, Methanol-d_4) δ [ppm] 158.0, 139.8, 133.2, 130.1, 127.5, 122.8, 119.8, 110.9, 53.0, 51.7, 47.8, 19.1, 18.3; **HRMS** (APCI) 231.1130, theoretical value for $C_{13}H_{15}BrN_2O_2$ $[M-Br]^+$ 231.1134.

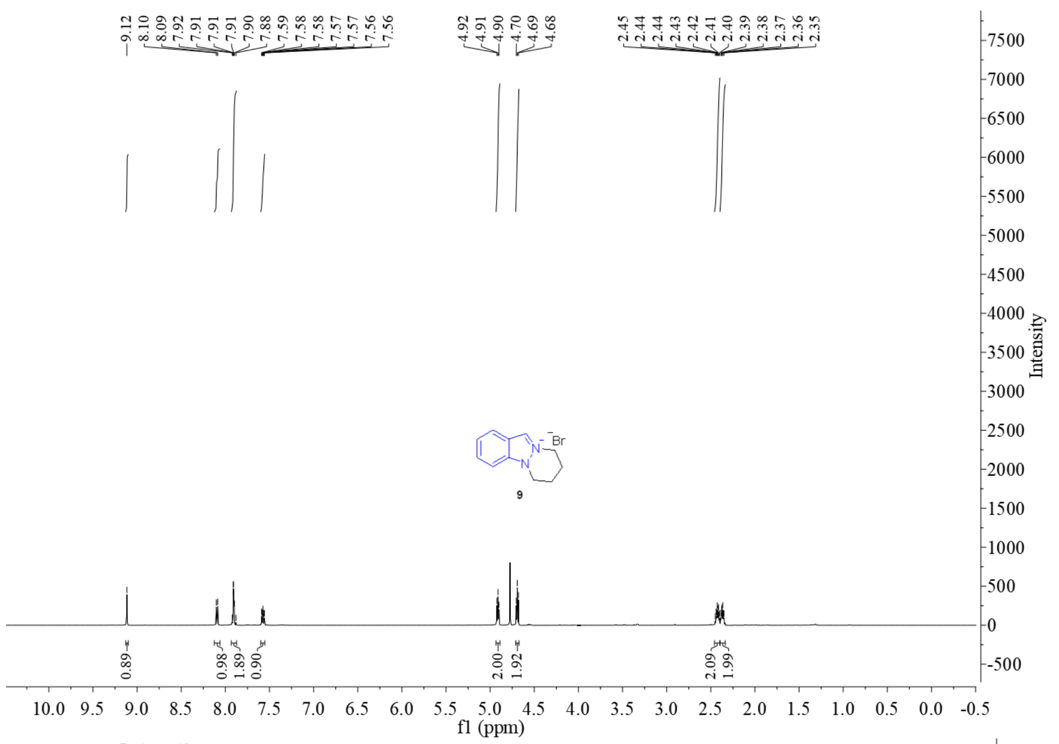

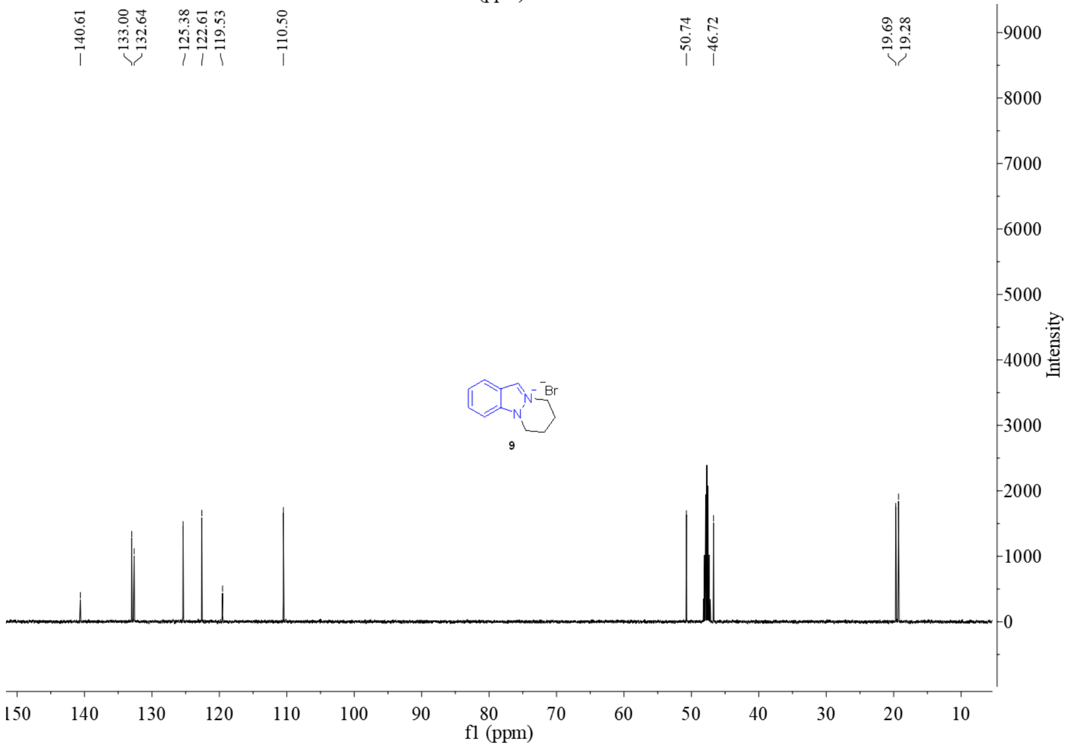

^{1}H, ^{13}C-NMR spectra of product 9. **^{1}H NMR** (500 MHz, Methanol-*d*4) δ [ppm] 9.12 (s, 1H), 8.09 (d, J = 8.4 Hz, 1H), 7.93 – 7.87 (m, 2H), 7.57 (ddd, J = 7.8, 5.4, 2.4 Hz, 1H), 4.91 (t, J = 5.9 Hz, 2H), 4.69 (t, J = 6.1 Hz, 2H), 2.43 (ddd, J = 12.3, 7.2, 5.2 Hz, 2H), 2.37 (dt, J = 10.8, 6.1 Hz, 2H); **^{13}C NMR** (125 MHz, Methanol-*d*4) δ [ppm] 140.6, 133.0, 132.6, 125.4, 122.6, 119.5, 110.5, 50.7, 46.7, 19.7, 19.3; **HRMS** (ESI) 173.1089, theoretical value for $C_{13}H_{15}BrN_2O_2$ $[M\text{-}Br]^+$ 173.1079.

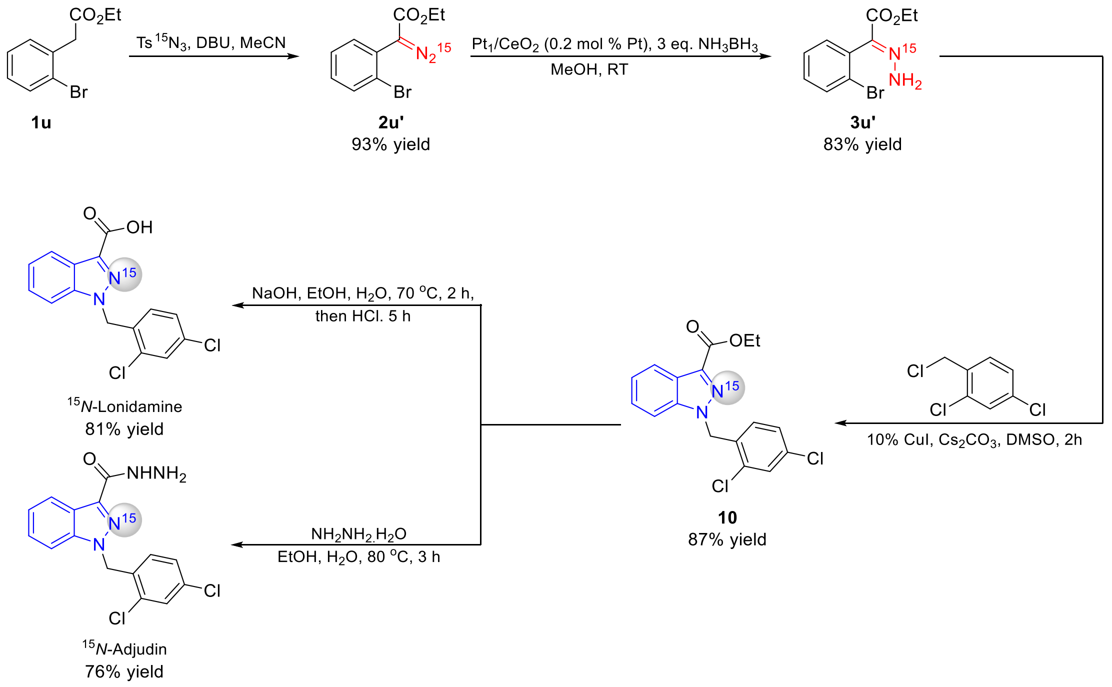

The synthesis of ^{15}N-labelled *Lonidamine* and *Adjudin*.

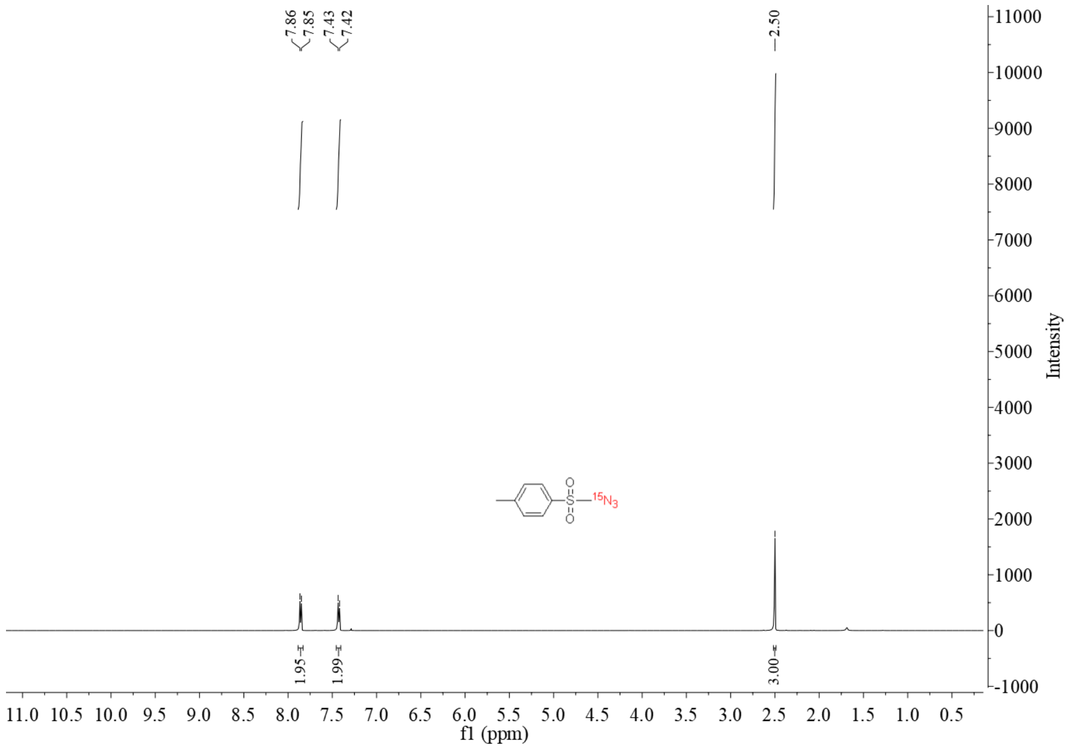

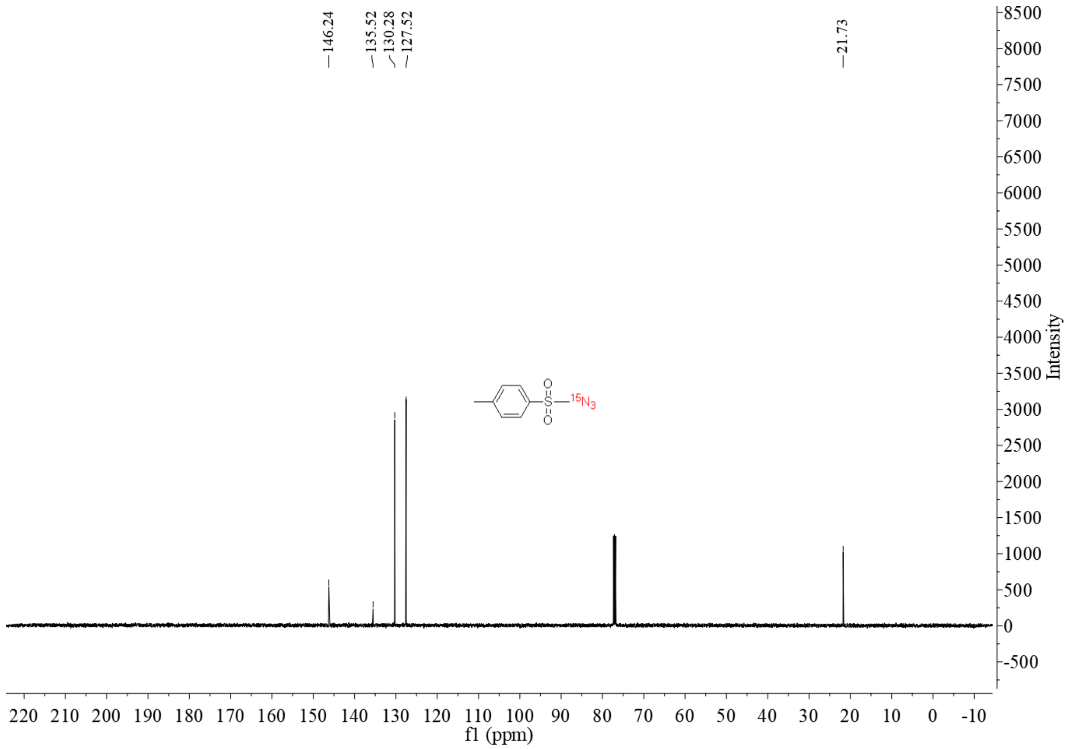

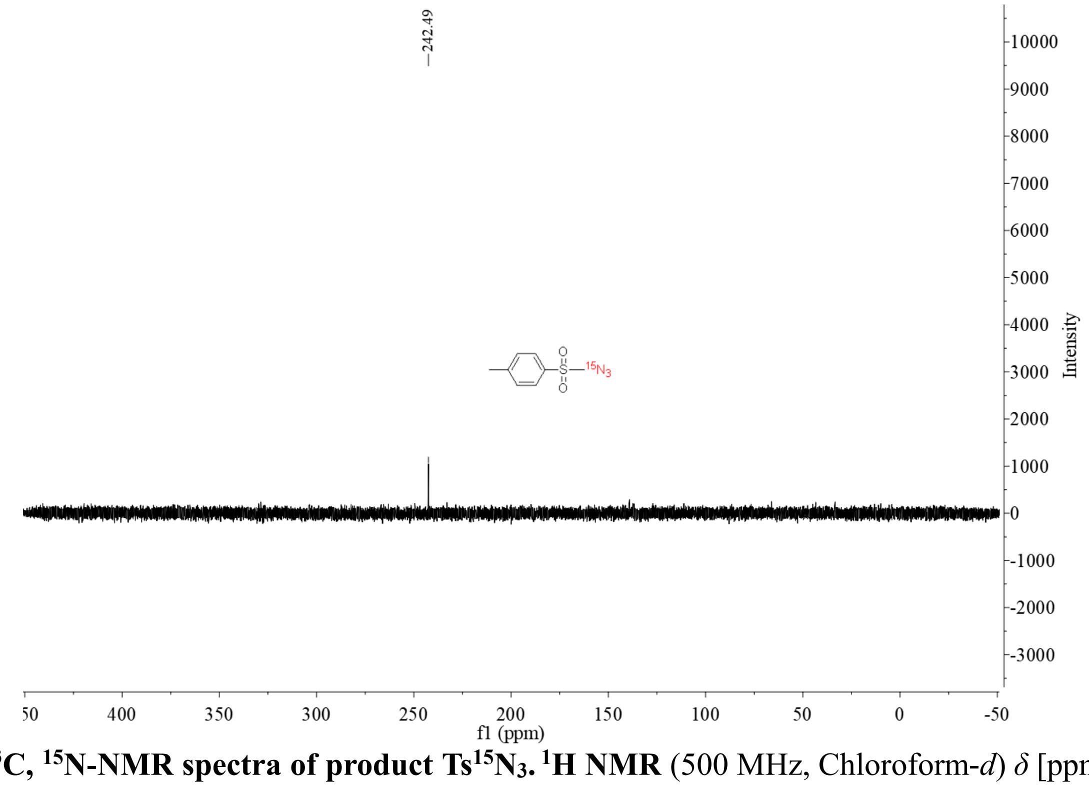

^{1}H, ^{13}C, ^{15}N-NMR spectra of product $Ts^{15}N_3$. **^{1}H NMR** (500 MHz, Chloroform-*d*) δ [ppm] 7.86 (d, J = 8.3 Hz, 2H), 7.43 (d, J = 8.0 Hz, 2H), 2.50 (s, 3H); **^{13}C NMR** (125 MHz, Chloroform-*d*) δ [ppm] 146.2, 135.5, 130.3, 127.5, 21.7; **^{15}N NMR** (51 MHz, Chloroform-*d*) δ [ppm] 242.5; **GC-MS** (APCI) 198.0, theoretical value for $C_7H_7N_2{}^{15}NO_2S$ 198.0.

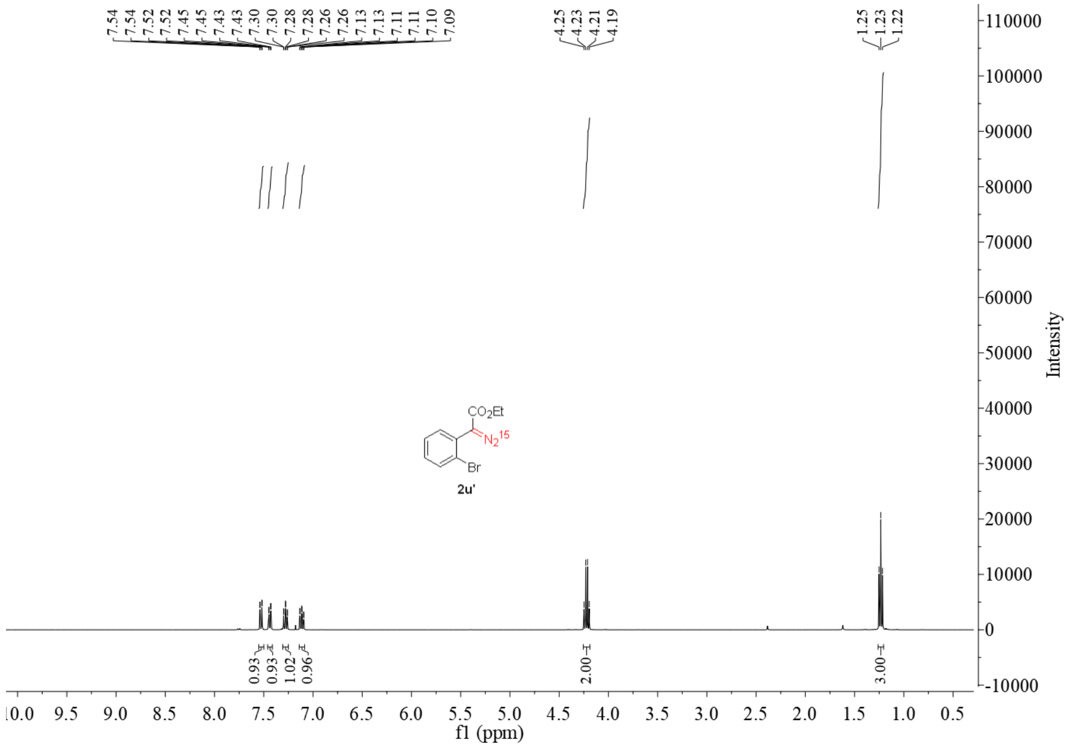

7.54
7.54
7.52
7.52
7.45
7.45
7.43
7.43
7.30
7.30
7.28
7.28
7.26
7.26
7.13
7.13
7.11
7.11
7.10
7.09
4.25
4.23
4.21
4.19
1.25
1.23
1.22
CO2Et
N2 15
Br
2u'
0.93
0.93
1.02
0.96
2.00
3.00
f1 (ppm)
Intensity

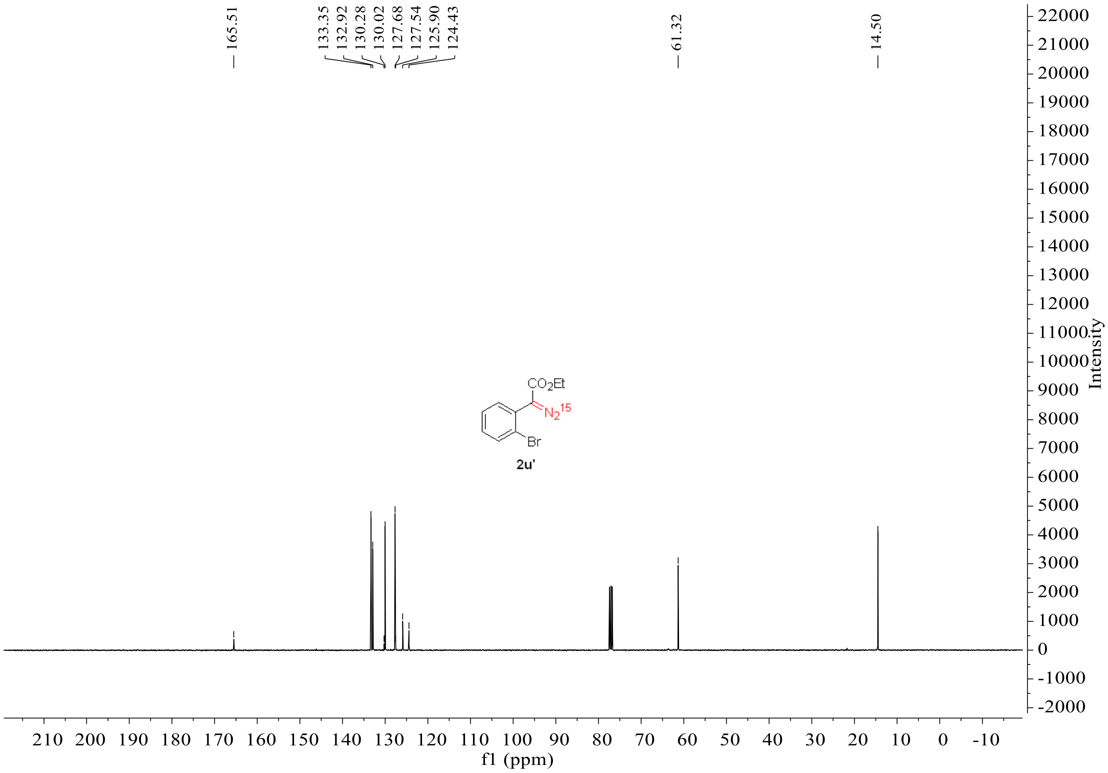

165.51
133.35
132.92
130.28
130.02
127.68
127.54
125.90
124.43
61.32
14.50
CO2Et
N2 15
Br
2u'
f1 (ppm)
Intensity

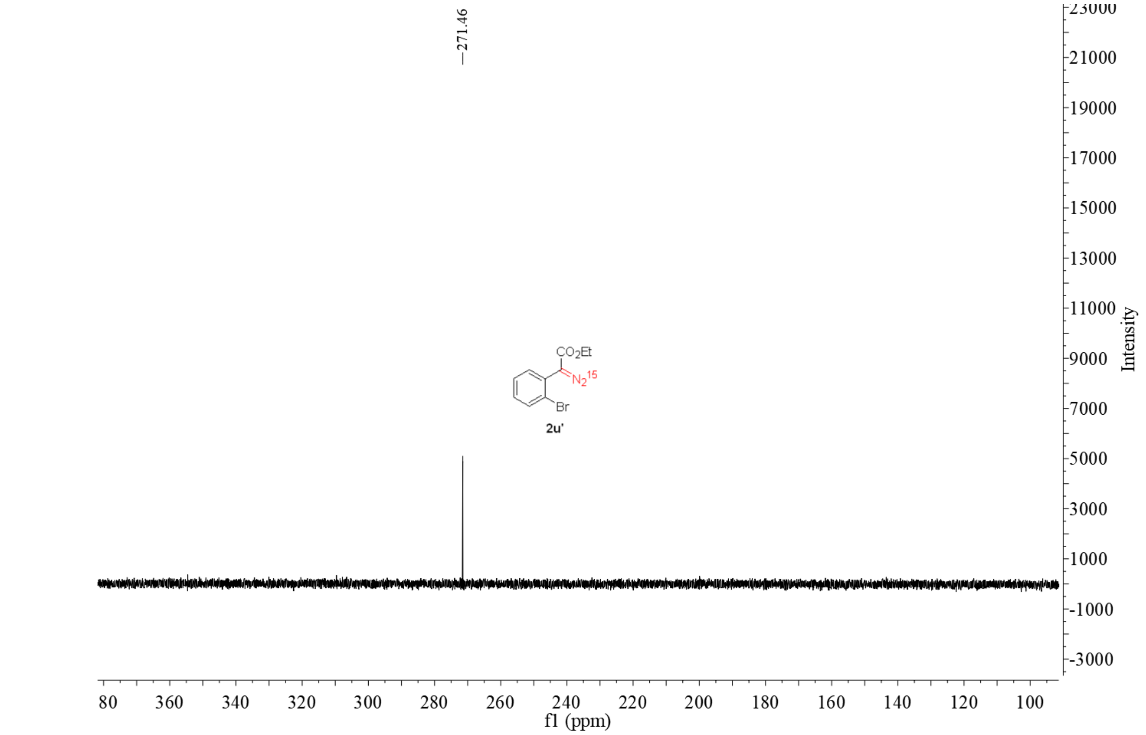

^{1}H, ^{13}C, ^{15}N-NMR spectra of product 2u'. **^{1}H NMR** (400 MHz, Chloroform-*d*) δ [ppm] 7.53 (dd, J = 8.1, 1.3 Hz, 1H), 7.44 (dd, J = 7.8, 1.6 Hz, 1H), 7.28 (td, J = 7.6, 1.3 Hz, 1H), 7.11 (td, J = 7.9, 1.7 Hz, 1H), 4.22 (q, J = 7.1 Hz, 2H), 1.23 (t, J = 7.1 Hz, 3H); **^{13}C NMR** (100 MHz, Chloroform-*d*) δ [ppm] 165.5, 133.4, 132.9, 130.0, 128.9 (d, J = 274 Hz), 127.6, 125.9, 124.4, 61.3, 14.5; **^{15}N NMR** (51 MHz, Chloroform-*d*) δ [ppm] 271.5; **HRMS** (APCI) 269.9889, theoretical value for $C_{10}H_9BrN^{15}NO_2$ $[M+H]^+$ 269.9896.

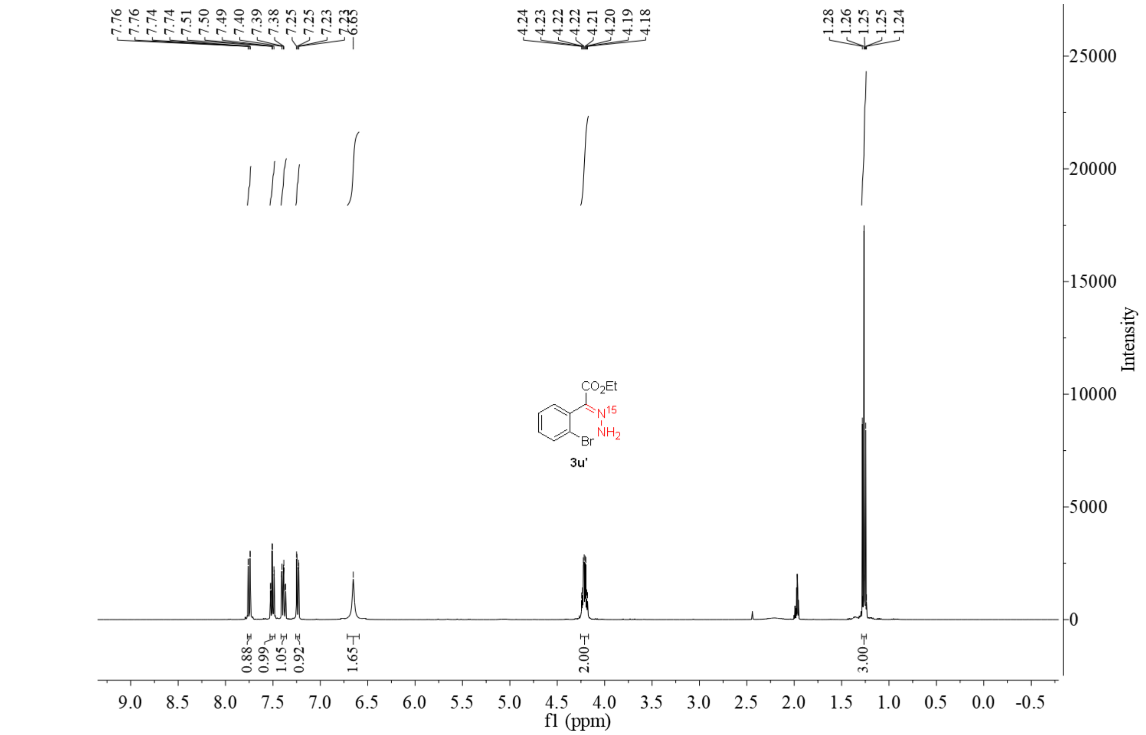

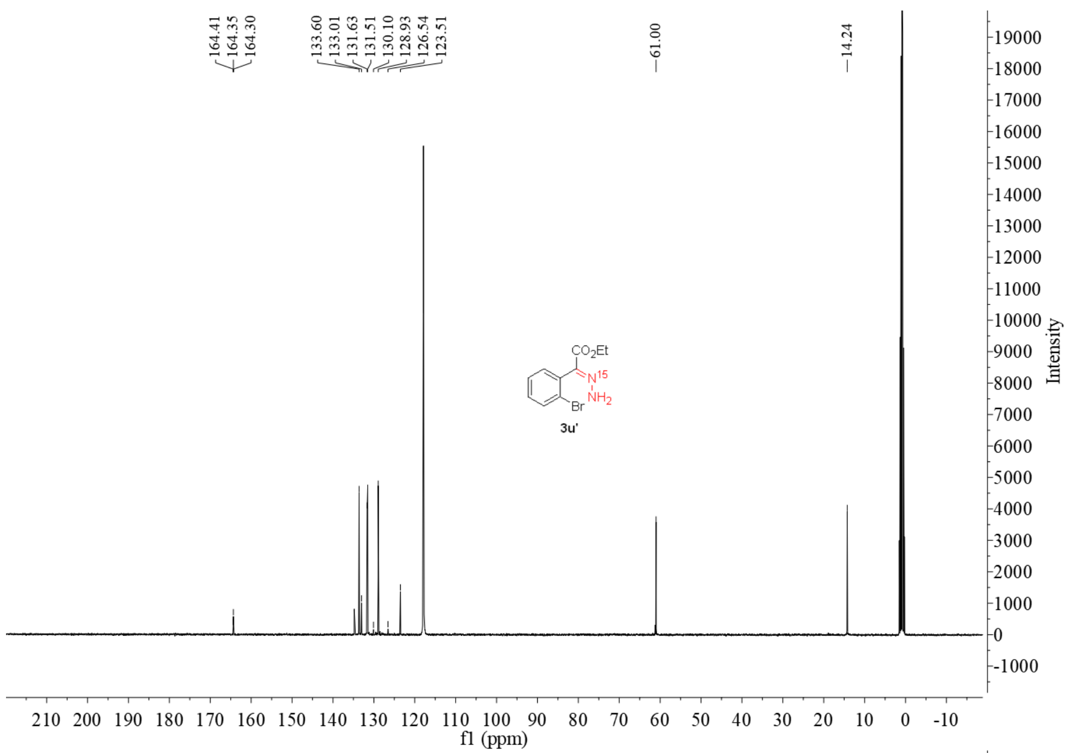

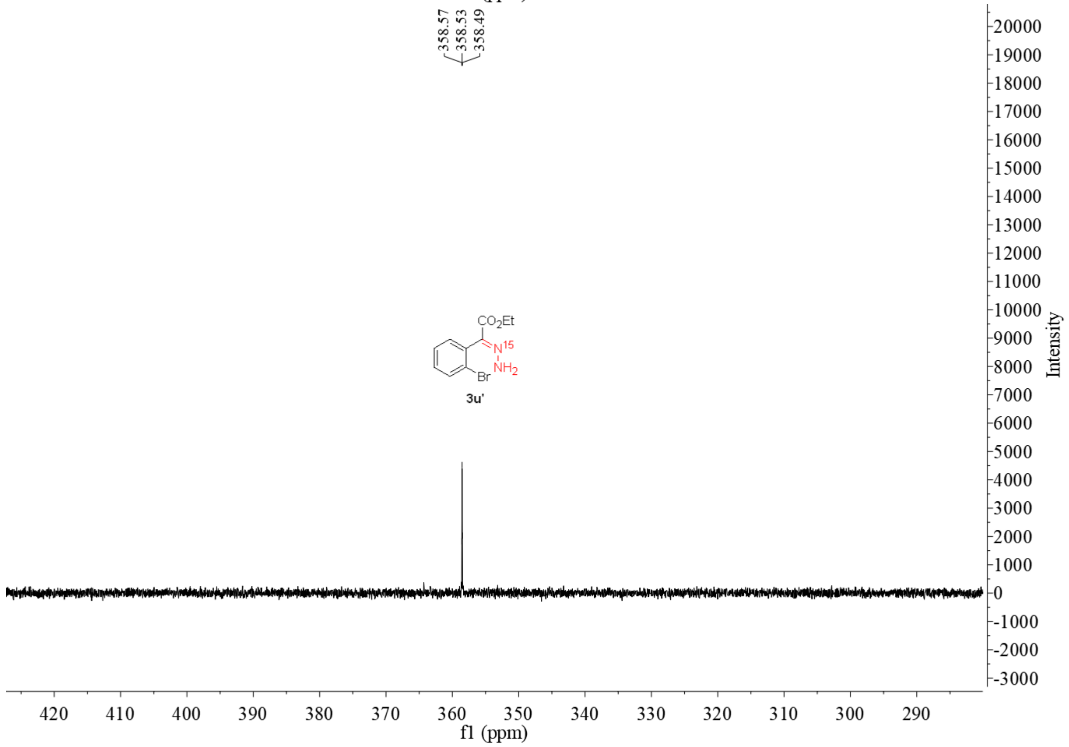

^{1}H, ^{13}C, ^{15}N-NMR spectra of product 3u’. **^{1}H NMR** (400 MHz, Acetonitrile-*d*3) δ [ppm] 7.75 (dd, J = 8.1, 1.1 Hz, 1H), 7.51 (td, J = 7.5, 1.2 Hz, 1H), 7.39 (td, J = 7.8, 1.7 Hz, 1H), 7.24 (dd, J = 7.6, 1.7 Hz, 1H), 6.65 (s, 2H), 4.21 (qd, J = 7.1, 3.4 Hz, 2H), 1.26 (t, J = 7.1 Hz, 3H); **^{13}C NMR** (100 MHz, Acetonitrile-*d*3) δ [ppm] 164.4 (t, J = 50 Hz,), 133.6, 133.0, 131.6, 131.5, 128.9, 128.3 (d, J = 365 Hz), 123.5, 61.0, 14.2; **^{15}N NMR** (51 MHz, Acetonitrile-*d*3) δ [ppm] 358.3 (t, J = 2.3 Hz); **HRMS** (ESI) 293.9864, theoretical value for $C_{10}H_{11}Br^{15}NNO_2$ $[M+Na]^+$ 293.9872.

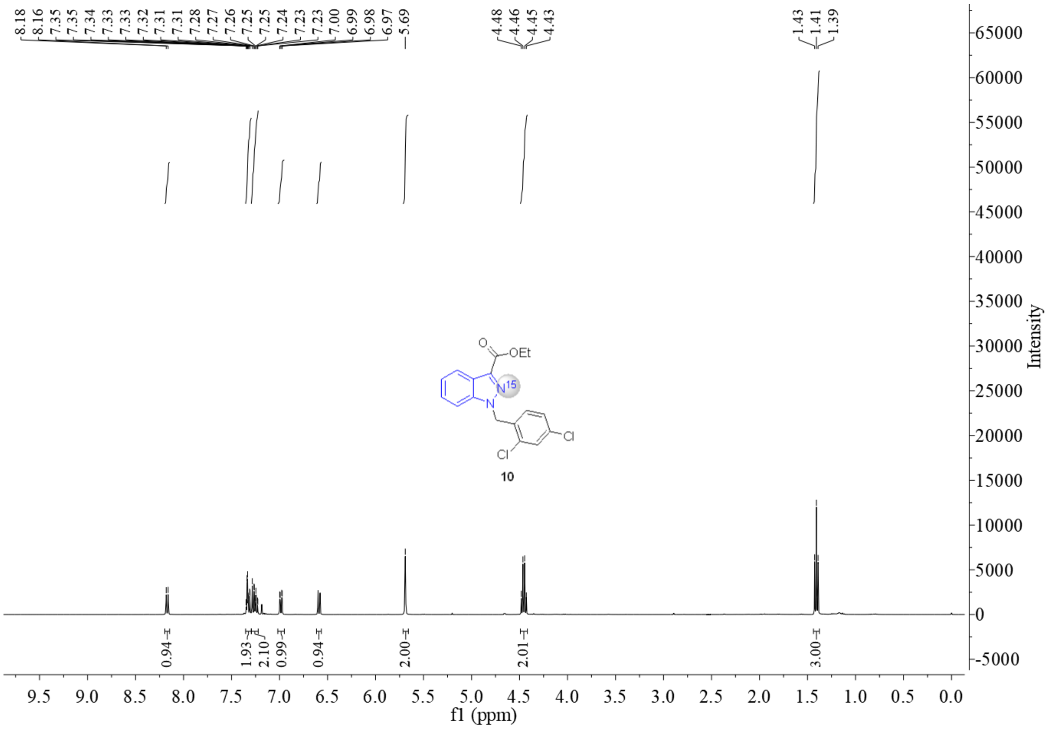

8.18
8.16
7.35
7.35
7.34
7.33
7.33
7.32
7.31
7.31
7.28
7.27
7.26
7.25
7.25
7.24
7.23
7.23
7.00
6.99
6.98
6.97
5.69
4.48
4.46
4.45
4.43
1.43
1.41
1.39
0.94
1.93
2.10
0.99
0.94
2.00
2.01
3.00
O
OEt
N15
N
Cl
Cl
10
f1 (ppm)
Intensity

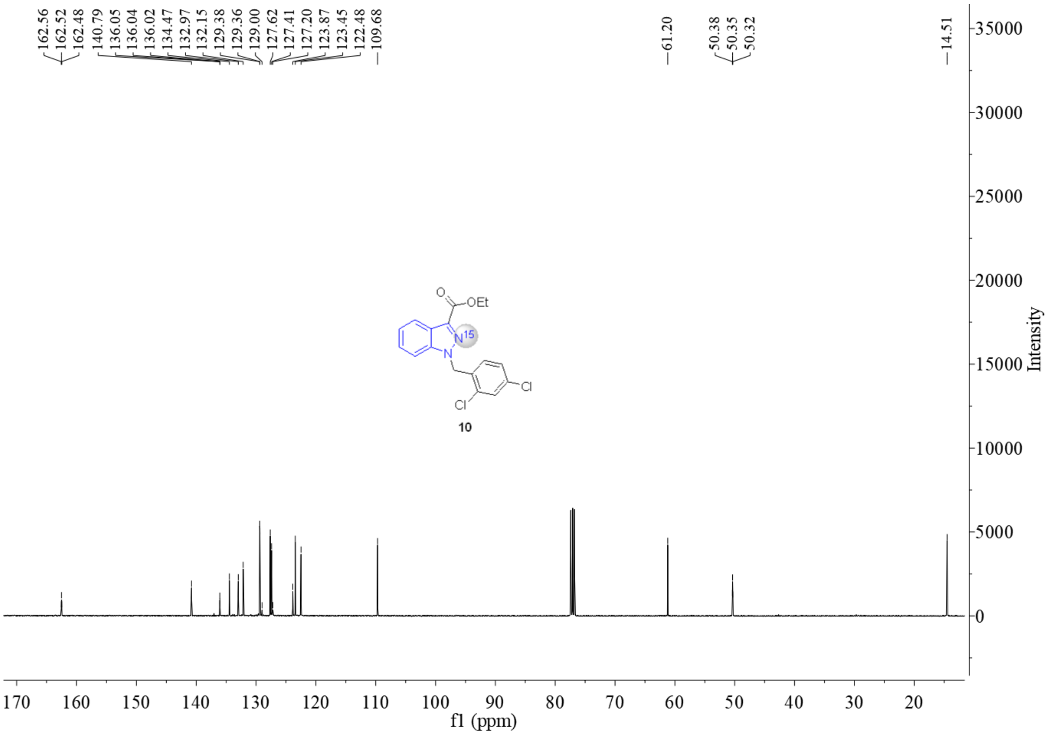

162.56
162.52
162.48
140.79
136.05
136.04
136.02
134.47
132.97
132.15
129.38
129.36
129.00
127.62
127.41
127.20
123.87
123.45
122.48
109.68
61.20
50.38
50.35
50.32
14.51
O
OEt
N15
N
Cl
Cl
10
f1 (ppm)
Intensity

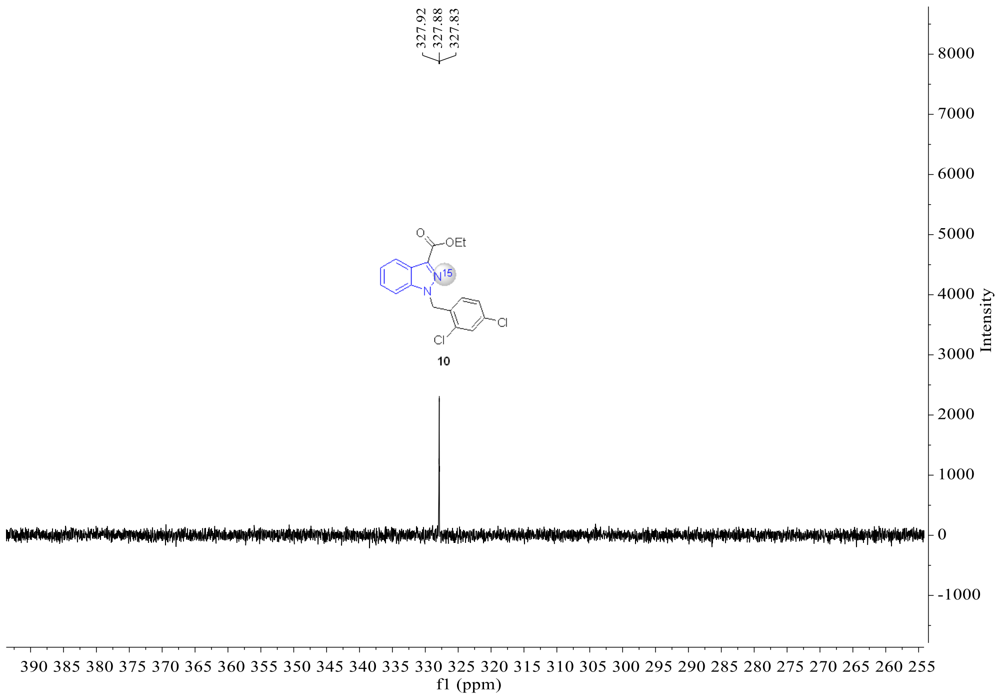

^{1}H, ^{13}C, ^{15}N-NMR spectra of product 10. **^{1}H NMR** (400 MHz, Chloroform-*d*) δ [ppm] 8.17 (d, *J* = 8.1 Hz, 1H), 7.35 – 7.29 (m, 2H), 7.29 – 7.22 (m, 2H), 6.99 (dd, *J* = 8.4, 2.1 Hz, 1H), 6.59 (d, *J* = 8.4 Hz, 1H), 5.69 (s, 2H), 4.49 – 4.42 (m, 2H), 1.41 (t, *J* = 7.1 Hz, 3H); **^{13}C NMR** (100 MHz, Chloroform-*d*) δ [ppm] 162.5 (t, *J* = 5 Hz), 140.8, 136.0 (t, *J* = 1 Hz), 134.5, 133.0, 132.2, 129.4, 129.3, 127.6, 127.4, 123.9, 123.4, 122.5, 109.7, 61.2, 50.3 (t, *J* = 3 Hz), 14.5; **^{15}N NMR** (51 MHz, Chloroform-*d*) δ [ppm] 327.8 (t, *J* = 2.3 Hz); **HRMS** (ESI) 350.0490, theoretical value for $C_{17}H_{14}Cl_2{}^{15}NNO_2$ $[M+Na]^+$ 350.0481.

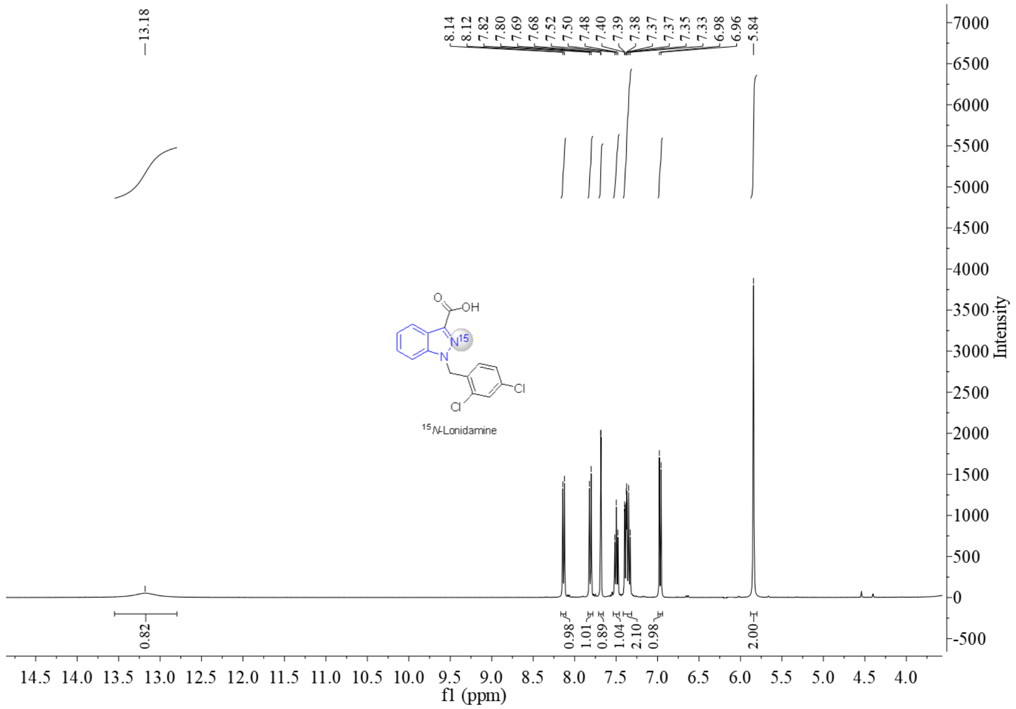

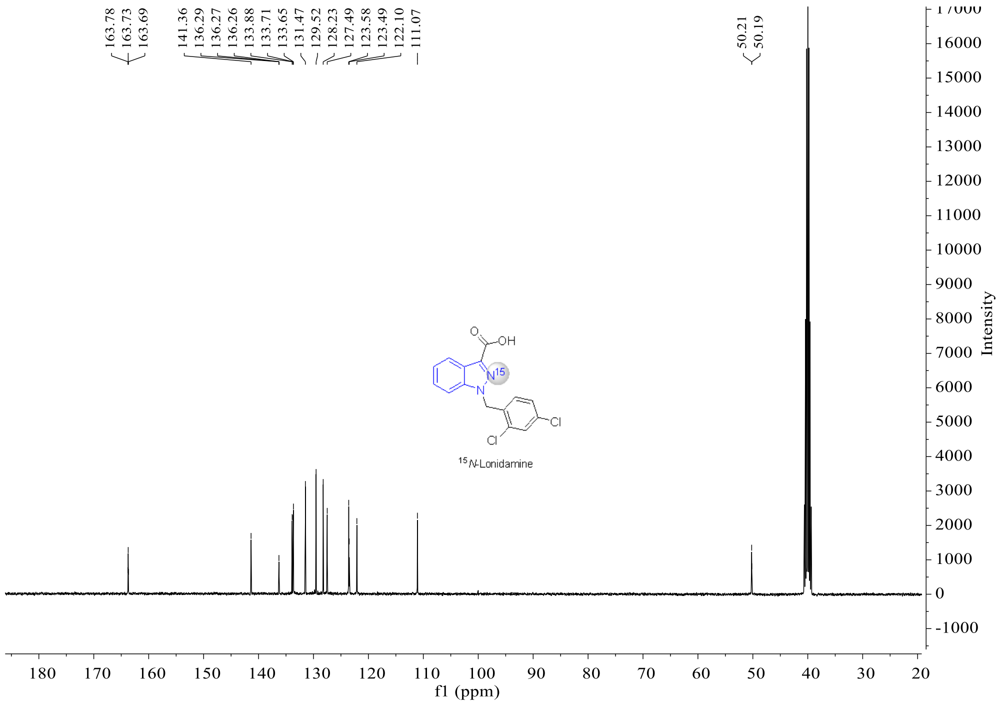

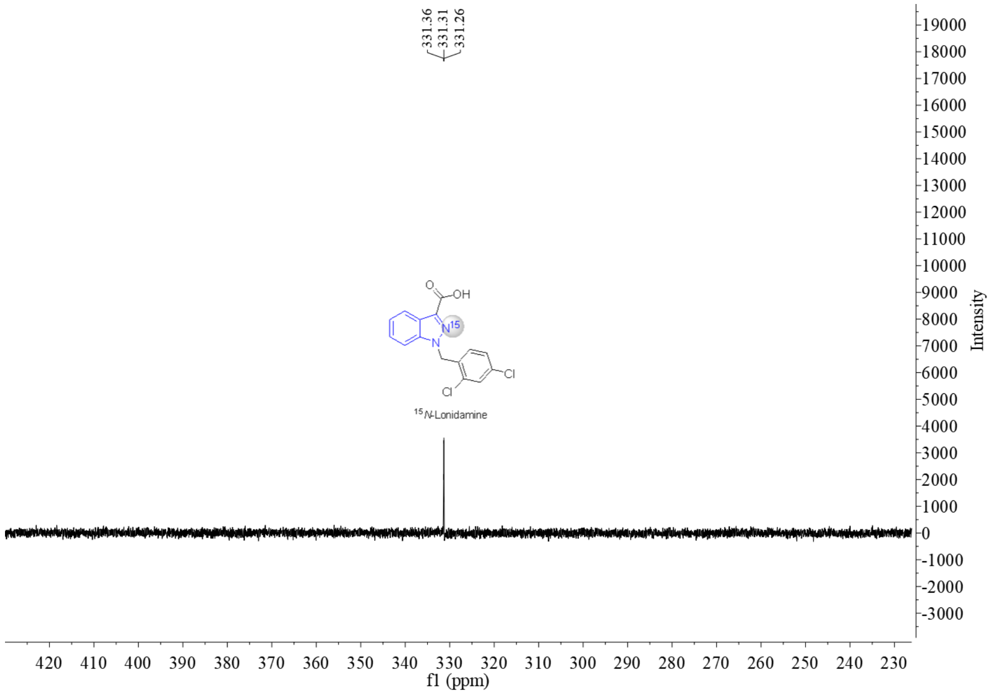

^{1}H, ^{13}C, ^{15}N-NMR spectra of product ^{15}N-labelled *Lonidamine*. **^{1}H NMR** (400 MHz, DMSO-d_6) δ [ppm] 13.18 (s, 1H), 8.13 (d, J = 8.2 Hz, 1H), 7.81 (d, J = 8.5 Hz, 1H), 7.68 (d, J = 2.1 Hz, 1H), 7.50 (t, J = 7.4 Hz, 1H), 7.41 – 7.31 (m, 2H), 6.97 (d, J = 8.4 Hz, 1H), 5.84 (s, 2H); **^{13}C NMR** (100 MHz, DMSO-d_6) δ [ppm] 163.7 (t, J = 5 Hz), 141.4, 136.2 (t, J = 1 Hz), 133.9, 133.7, 133.6, 131.5, 129.5, 128.2, 127.5, 123.6, 123.5, 122.1, 111.1, 50.2 (t, J = 2 Hz); **^{15}N NMR** (51 MHz, DMSO-d_6) δ [ppm] 331.3 (t, J = 2.6 Hz); **HRMS** (ESI) 343.9986, theoretical value for $C_{17}H_{14}Cl_2{}^{15}NNO_2$ $[M+Na]^+$ 343.9988.

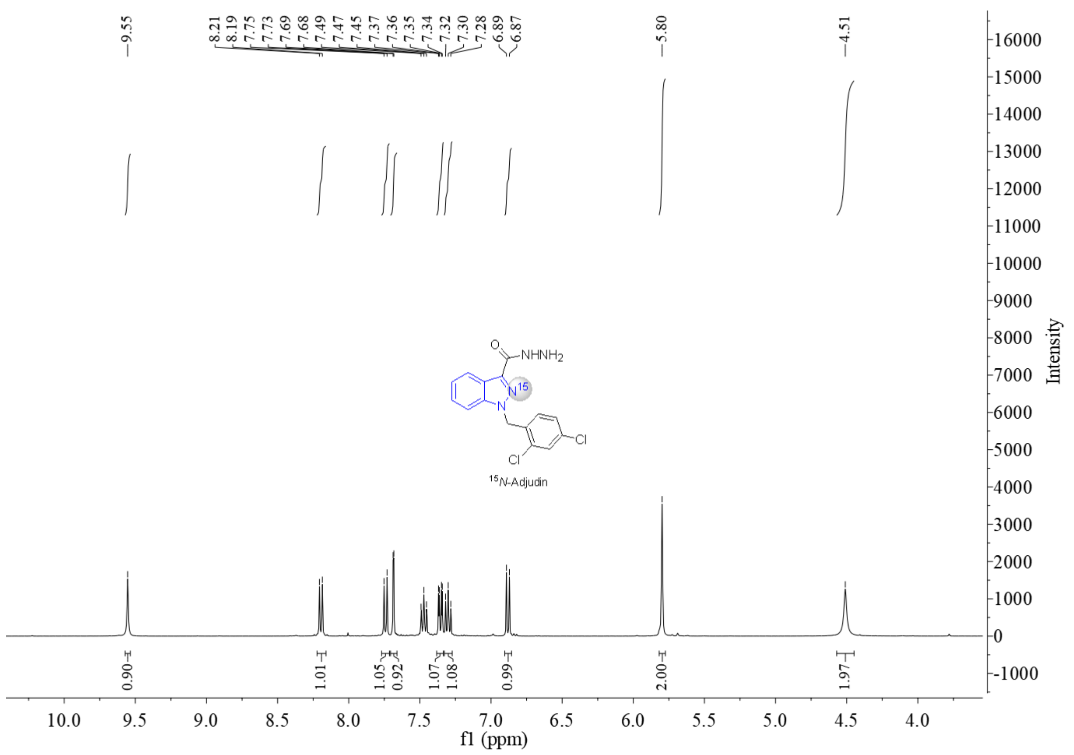
9.55
8.21
8.19
7.75
7.73
7.69
7.68
7.49
7.47
7.45
7.37
7.36
7.35
7.34
7.32
7.30
7.28
6.89
6.87
5.80
4.51
O
NHNH2
N15
N
Cl
Cl
15N-Adjudin
0.90
1.01
1.05
0.92
1.07
1.08
0.99
2.00
1.97
f1 (ppm)
Intensity

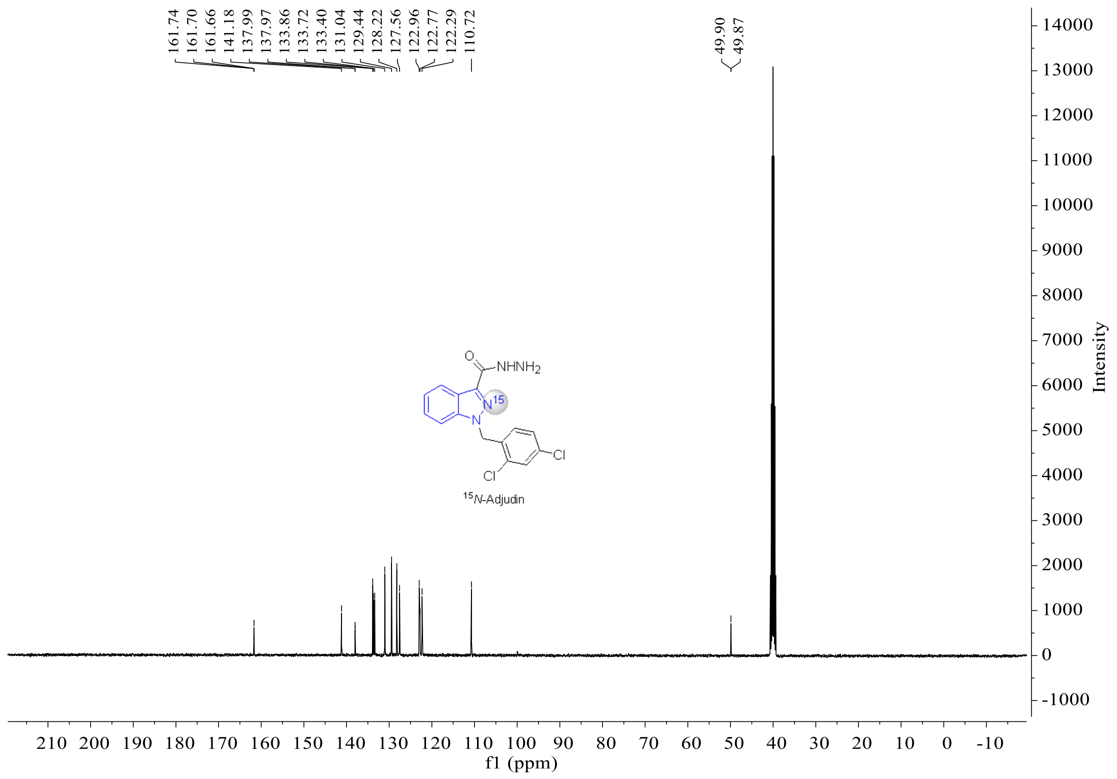
161.74
161.70
161.66
141.18
137.99
137.97
133.86
133.72
133.40
131.04
129.44
128.22
127.56
122.96
122.77
122.29
110.72
49.90
49.87
O
NHNH2
N15
N
Cl
Cl
15N-Adjudin
f1 (ppm)
Intensity

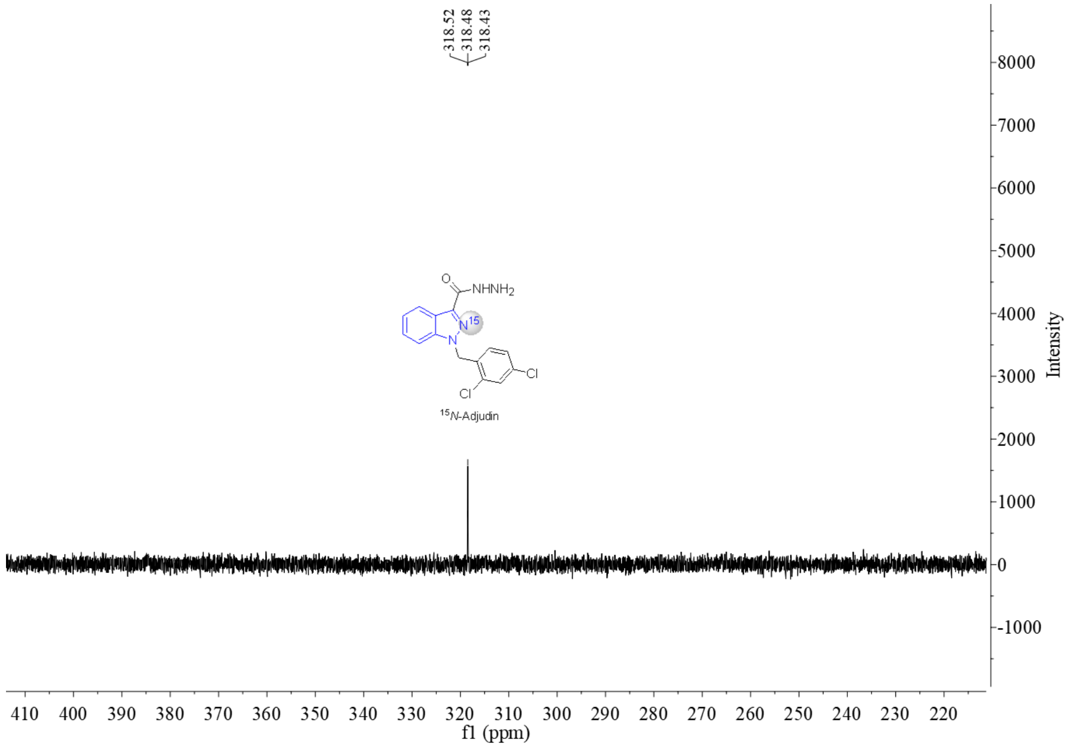

^{1}H, ^{13}C, ^{15}N-NMR spectra of product ^{15}N-labelled *Adjudin*. **^{1}H NMR** (400 MHz, DMSO-d_6) δ [ppm] 9.55 (s, 1H), 8.20 (d, J = 8.2 Hz, 1H), 7.74 (d, J = 8.6 Hz, 1H), 7.69 (d, J = 2.1 Hz, 1H), 7.36 (dd, J = 8.4, 2.1 Hz, 1H), 7.33 – 7.27 (m, 1H), 6.88 (d, J = 8.4 Hz, 1H), 5.80 (s, 2H), 4.51 (s, 2H); **^{13}C NMR** (100 MHz, DMSO-d_6) δ [ppm] 161.7 (t, J = 4 Hz), 141.2, 137.9 (t, J = 2 Hz), 133.9, 133.7, 133.4, 131.0, 129.4, 128.2, 127.6, 123.0, 122.8, 122.3, 110.7, 49.9 (t, J = 3 Hz); **^{15}N NMR** (51 MHz, DMSO-d_6) δ [ppm] 318.4 (t, J = 2.3 Hz); **HRMS** (ESI) 336.0439, theoretical value for $C_{15}H_{12}Cl_2N_3{}^{15}NO$ $[M+H]^+$ 336.0437.

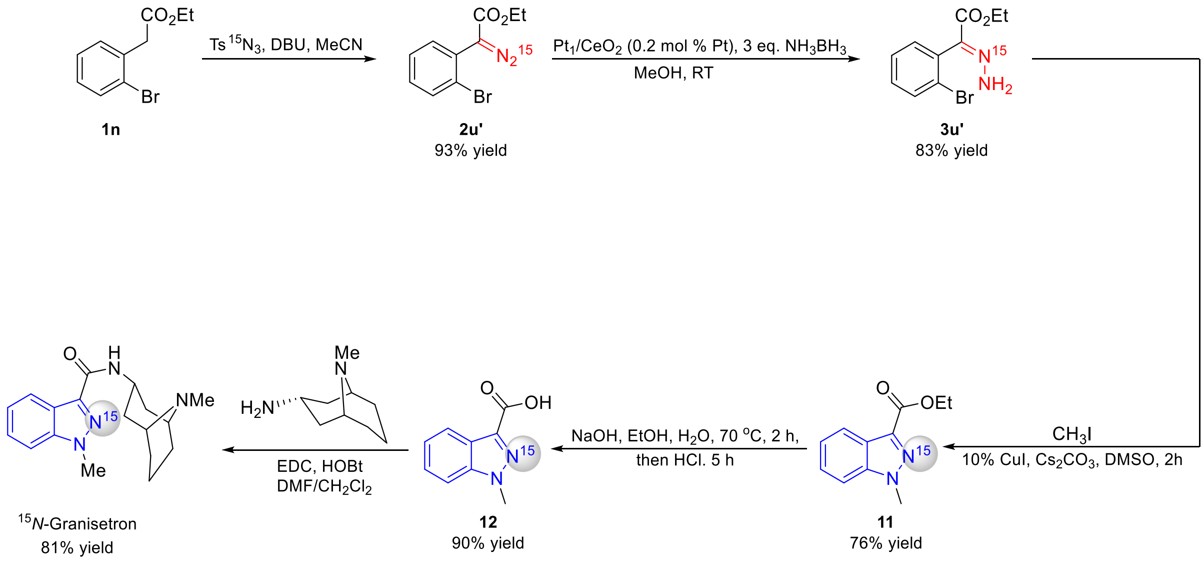

The synthesis of ^{15}N-labelled *Granisetron*.

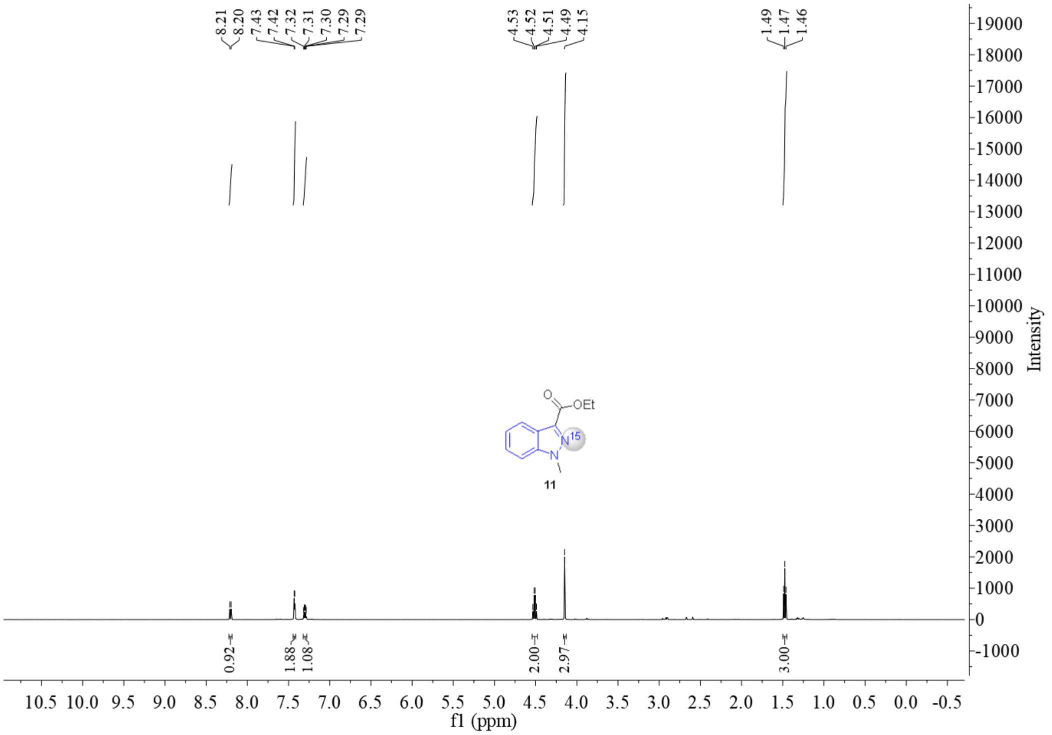
8.21
8.20
7.43
7.42
7.32
7.31
7.30
7.29
7.29
4.53
4.52
4.51
4.49
4.15
1.49
1.47
1.46
OEt
O
N15
N
11
0.92
1.88
1.08
2.00
2.97
3.00
f1 (ppm)
Intensity

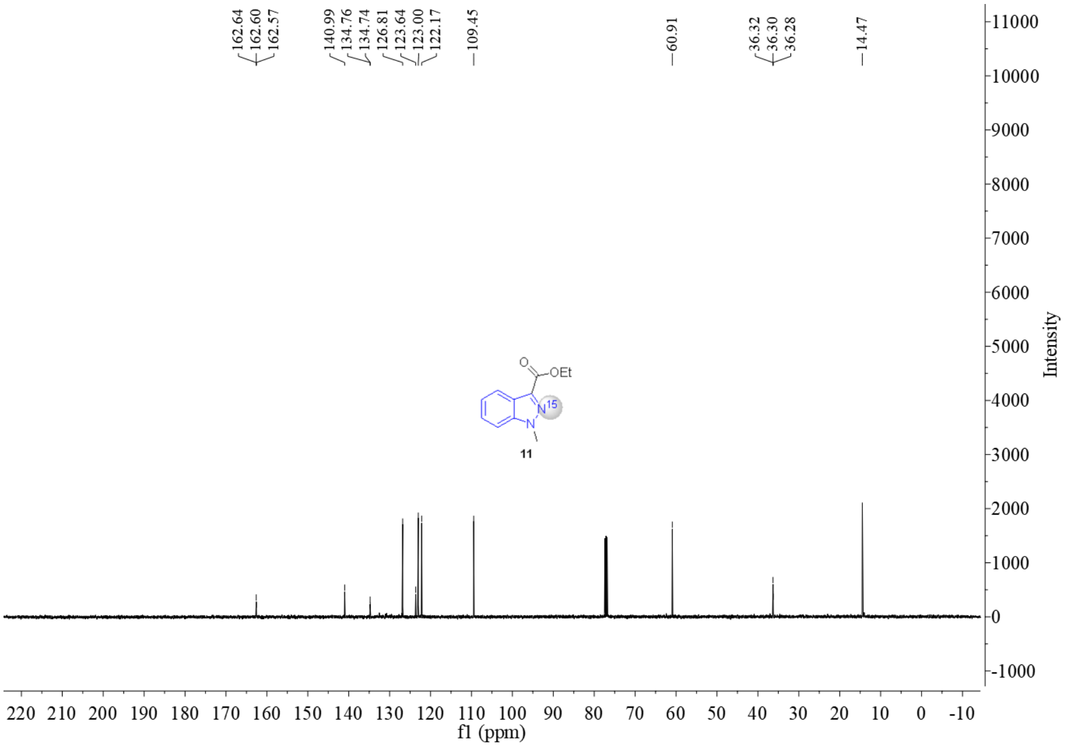
162.64
162.60
162.57
140.99
134.76
134.74
126.81
123.64
123.00
122.17
109.45
60.91
36.32
36.30
36.28
14.47
OEt
O
N15
N
11
f1 (ppm)
Intensity

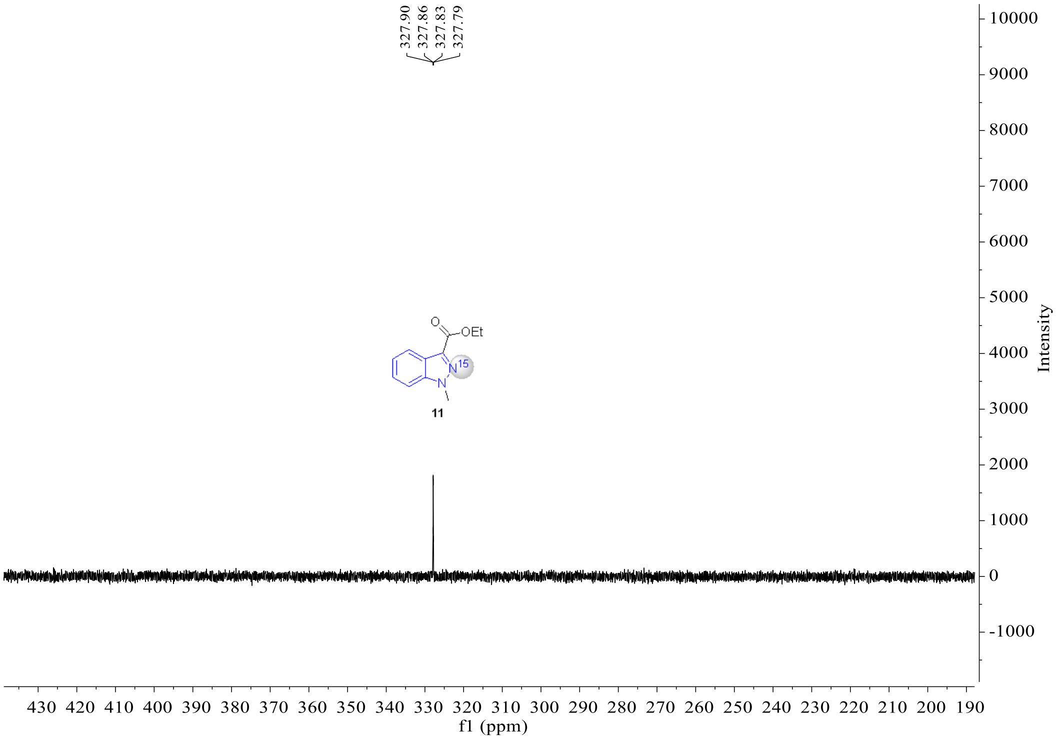

^{1}H, ^{13}C, ^{15}N-NMR spectra of product 11. **^{1}H NMR** (500 MHz, Chloroform-*d*) δ [ppm] 8.21 (d, J = 8.2 Hz, 1H), 7.43 (d, J = 3.6 Hz, 2H), 7.30 (dt, J = 7.6, 3.7 Hz, 1H), 4.51 (q, J = 7.1 Hz, 2H), 4.15 (s, 3H), 1.47 (t, J = 7.1 Hz, 3H); **^{13}C NMR** (125 MHz, Chloroform-*d*) δ [ppm] 162.6 (t, J = 4 Hz), 141.0, 134.7 (t, J = 2 Hz), 126.8, 123.6, 123.0, 122.2, 109.4, 60.9, 36.3 (t, J = 3 Hz), 14.5; **^{15}N NMR** (51 MHz, Chloroform-*d*) δ [ppm] 333.2 (t, J = 1.8 Hz); **HRMS** (ESI) 228.0769, theoretical value for $C_{11}H_{12}N^{15}NO_2$ $[M+Na]^+$ 228.0767.

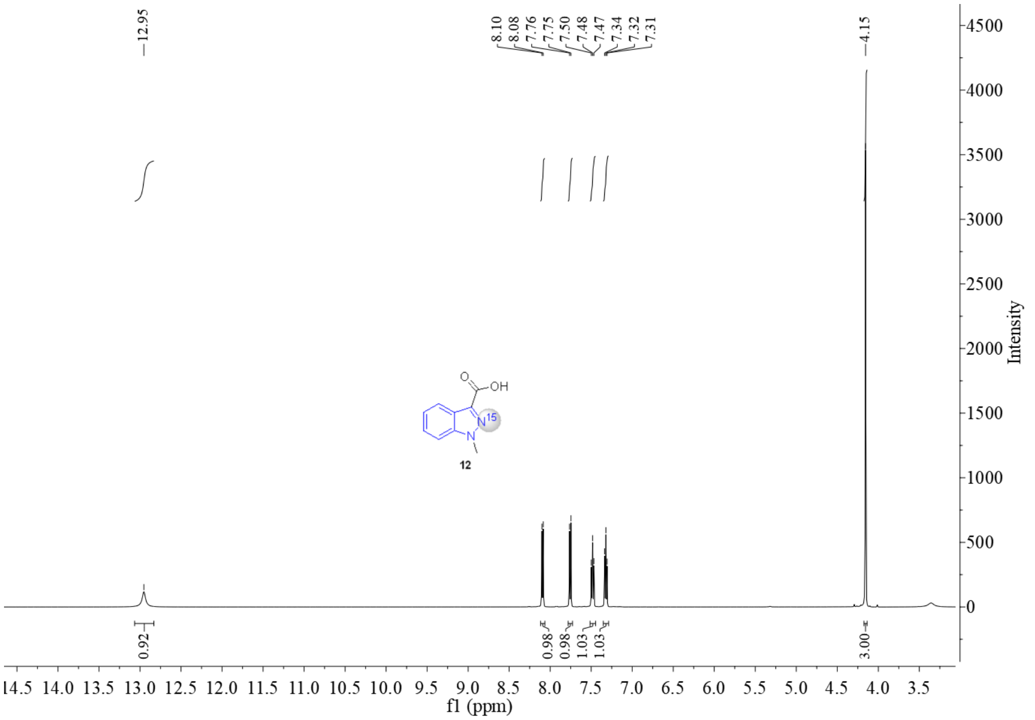

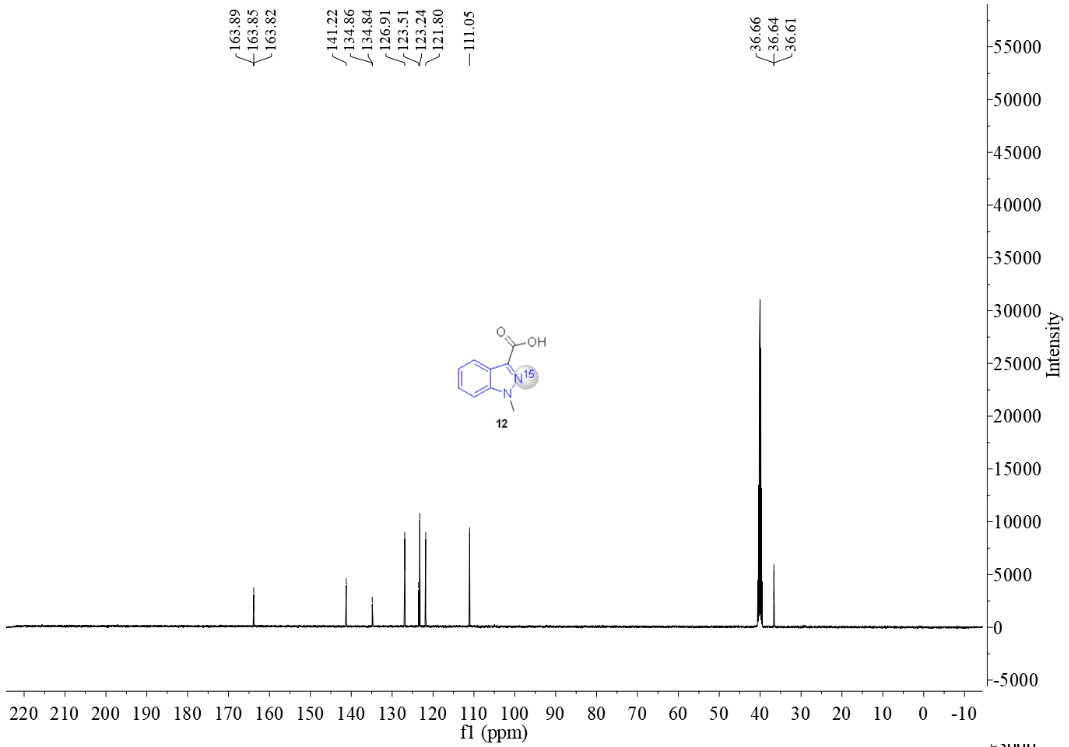

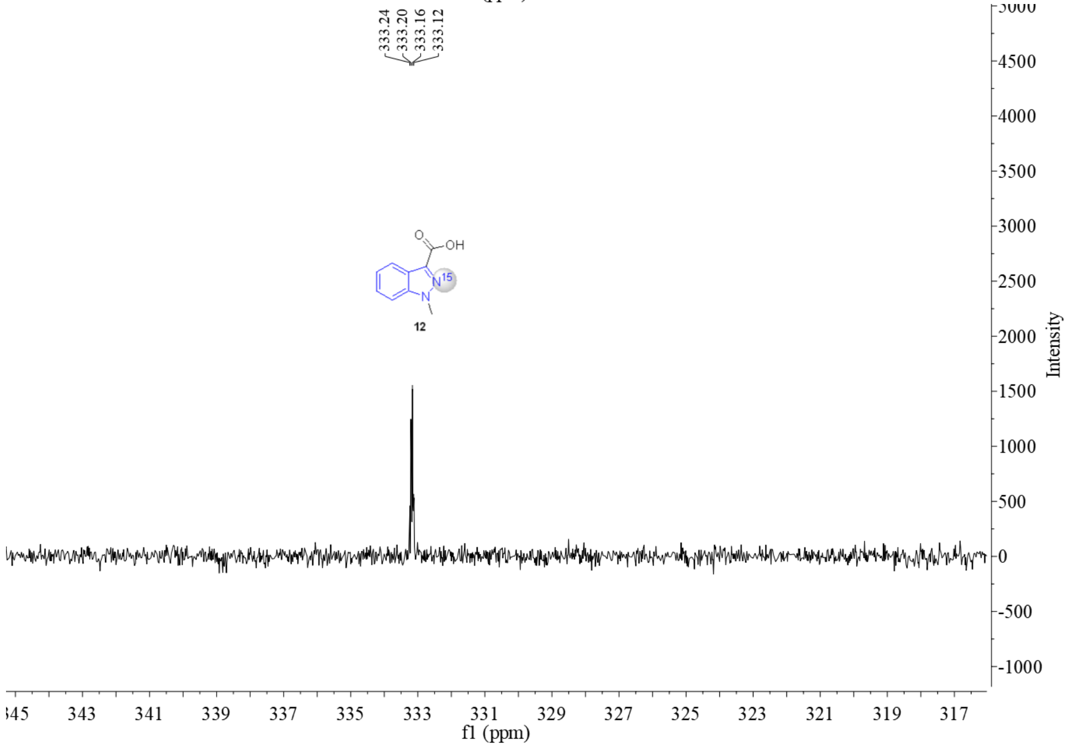

^{1}H, ^{13}C, ^{15}N-NMR spectra of product 12. **^{1}H NMR** (500 MHz, DMSO-d_6) δ [ppm] 12.95 (s, 1H), 8.09 (d, J = 8.2 Hz, 1H), 7.76 (d, J = 8.5 Hz, 1H), 7.48 (t, J = 7.7 Hz, 1H), 7.32 (t, J = 7.5 Hz, 1H), 4.15 (s, 3H); **^{13}C NMR** (125 MHz, DMSO-d_6) δ [ppm] 163.8 (t, J = 3 Hz), 141.2, 134.8 (t, J = 2 Hz), 126.9, 123.5, 123.2, 121.8, 111.0, 36.6 (t, J = 2.5 Hz); **^{15}N NMR** (51 MHz, DMSO-d_6) δ [ppm] 327.8 (q, J = 2.0 Hz); **HRMS** (ESI) 178.0633, theoretical value for $C_9H_8N^{15}NO_2$ $[M+H]^+$ 178.0634.

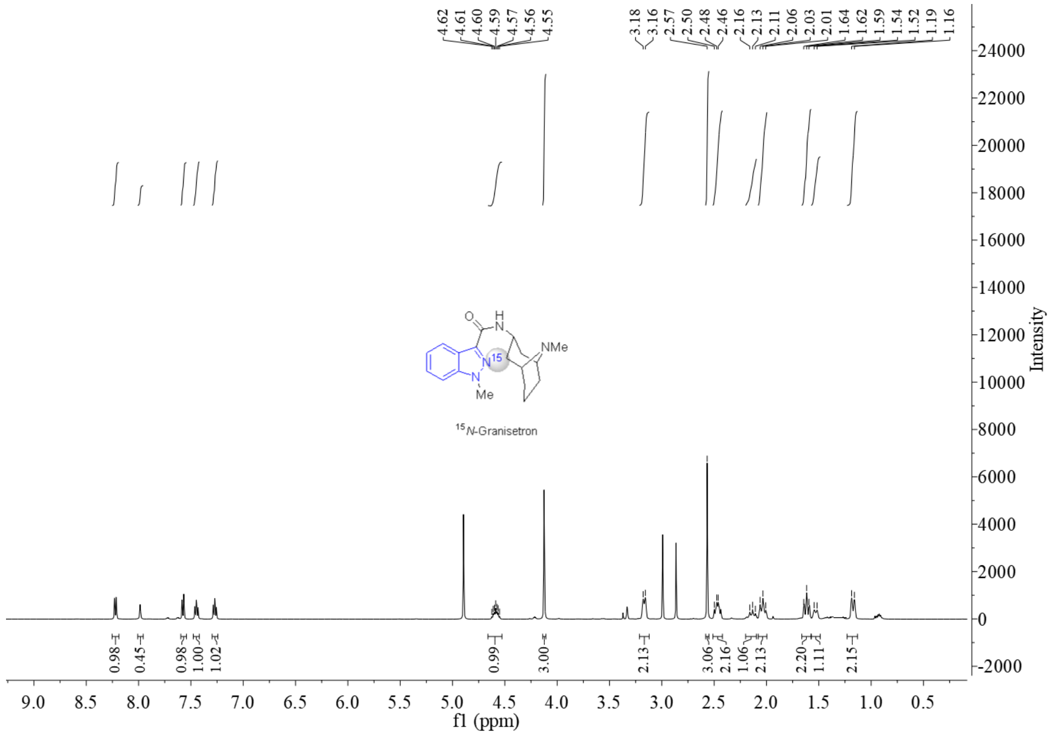
4.62 4.61 4.60 4.59 4.57 4.56 4.55
3.18 3.16 2.57 2.50 2.48 2.46 2.16 2.13 2.11 2.06 2.03 2.01 1.64 1.62 1.59 1.54 1.52 1.19 1.16
0.98 0.45 0.98 1.00 1.02 0.99 3.00 2.13 3.06 2.16 1.06 2.13 2.20 1.11 2.15
O
H
N
NMe
N
15
N
Me
15N-Granisetron
f1 (ppm)
Intensity

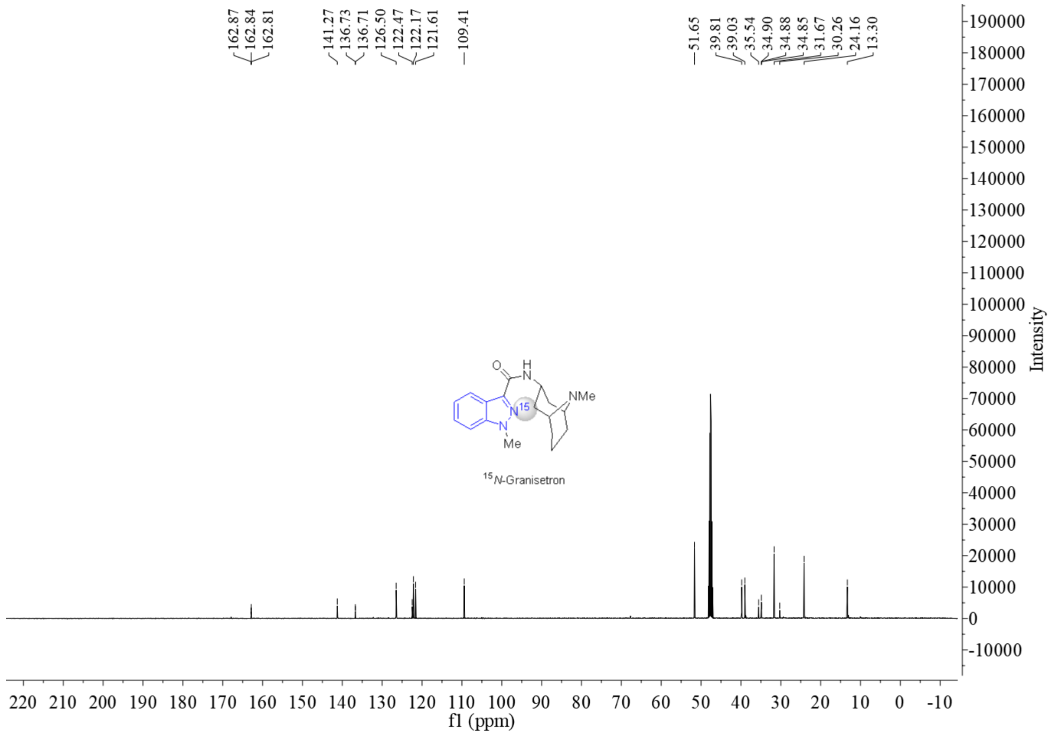
162.87 162.84 162.81
141.27 136.73 136.71 126.50 122.47 122.17 121.61
109.41
51.65
39.81 39.03 35.54 34.90 34.88 34.85 31.67 30.26 24.16 13.30
O
H
N
NMe
N
15
N
Me
15N-Granisetron
f1 (ppm)
Intensity

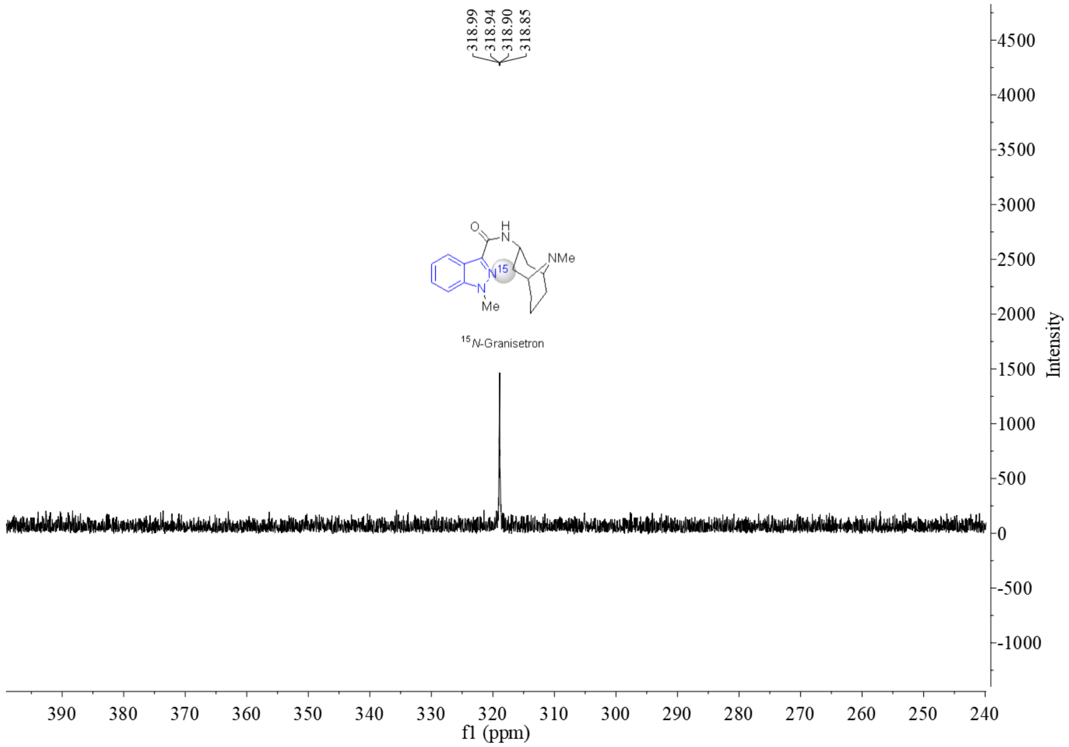

^{1}H, ^{13}C, ^{15}N-NMR spectra of product ^{15}N-labelled *Granisetron*. **^{1}H NMR** (500 MHz, Methanol-d_4) δ [ppm] 8.22 (dd, J = 8.1, 1.5 Hz, 1H), 7.98 (s, 1H), 7.58 (d, J = 8.5 Hz, 1H), 7.45 (ddd, J = 8.5, 6.9, 1.2 Hz, 1H), 7.30 – 7.24 (m, 1H), 4.59 (tt, J = 11.6, 6.8 Hz, 1H), 4.12 (d, J = 1.5 Hz, 3H), 3.17 (d, J = 10.4 Hz, 2H), 2.57 (s, 3H), 2.51 – 2.42 (m, 2H), 2.13 (t, J = 13.3 Hz, 1H), 2.03 (t, J = 13.4 Hz, 2H), 1.62 (t, J = 12.5 Hz, 2H), 1.53 (d, J = 13.5 Hz, 1H), 1.17 (d, J = 13.3 Hz, 2H); **^{13}C NMR** (125 MHz, Methanol-d_4) δ [ppm] 162.8 (t, J = 3 Hz), 141.3, 136.7 (t, J = 2 Hz), 126.5, 122.5, 122.2, 121.6, 109.4, 51.6, 39.8, 39.0, 35.5, 34.9 (t, J = 2.5 Hz), 31.7, 30.3, 24.2, 13.3; **^{15}N NMR** (51 MHz, Methanol-d_4) δ [ppm] 318.9 (q, J = 2.3 Hz); **HRMS** (ESI) 314.2005, theoretical value for $C_{18}H_{24}N_3{}^{15}NO$ $[M+H]^+$ 314.1998.

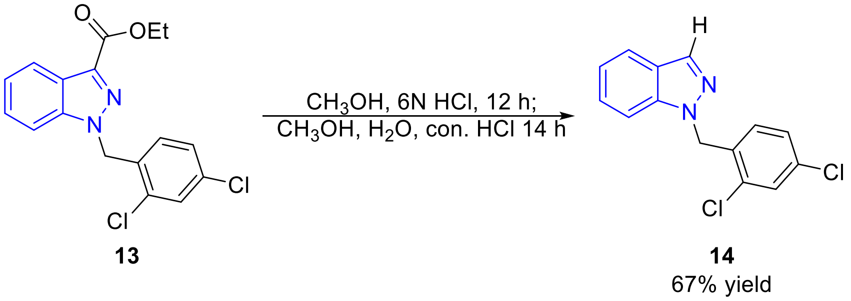

Product transformation by decarboxylation of ester to hydrogenation.

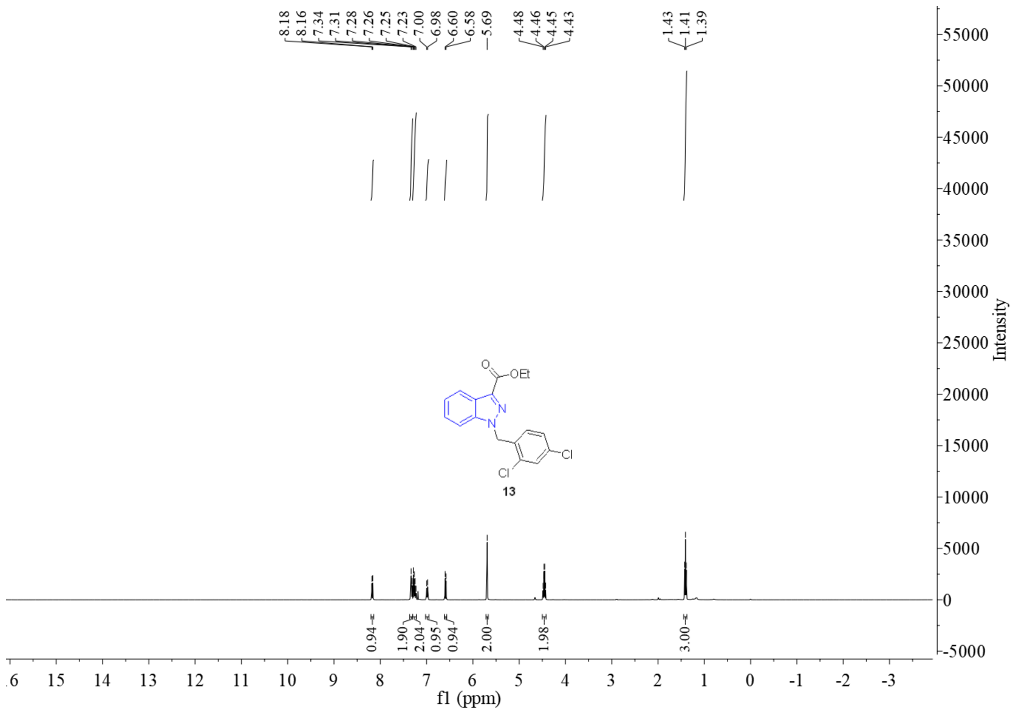

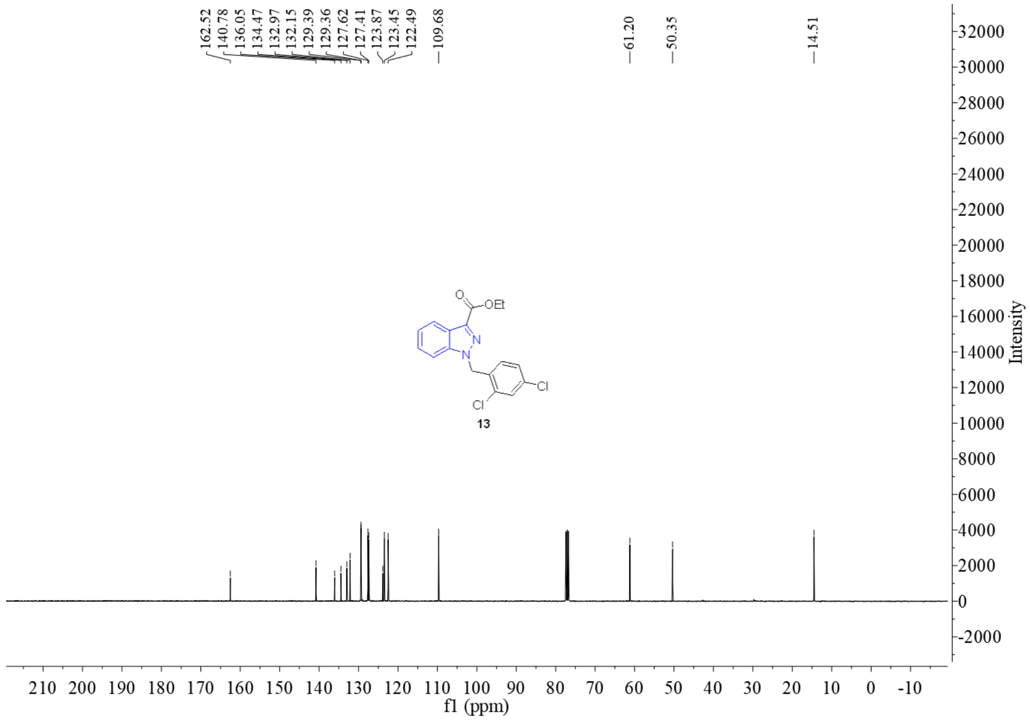

^{1}H, ^{13}C-NMR spectra of product 13. **^{1}H NMR** (400 MHz, Chloroform-*d*) δ [ppm] 8.17 (d, J = 8.1 Hz, 1H), 7.32 (d, J = 10.4 Hz, 2H), 7.26 (dd, J = 14.8, 7.8 Hz, 2H), 6.99 (d, J = 8.4 Hz, 1H), 6.59 (d, J = 8.4 Hz, 1H), 5.69 (s, 2H), 4.46 (q, J = 7.3 Hz, 2H), 1.41 (t, J = 7.1 Hz, 3H); **^{13}C NMR** (100 MHz, Chloroform-*d*) δ [ppm] 162.5, 140.8, 136.1, 134.5, 133.0, 132.2, 129.4, 129.3, 127.6, 127.4, 123.9, 123.4, 122.5, 109.7, 61.2, 50.4, 14.5; **HRMS** (ESI) 349.0509, theoretical value for $C_{17}H_{14}Cl_2N_2O_2$ $[M+H]^+$ 349.0510.

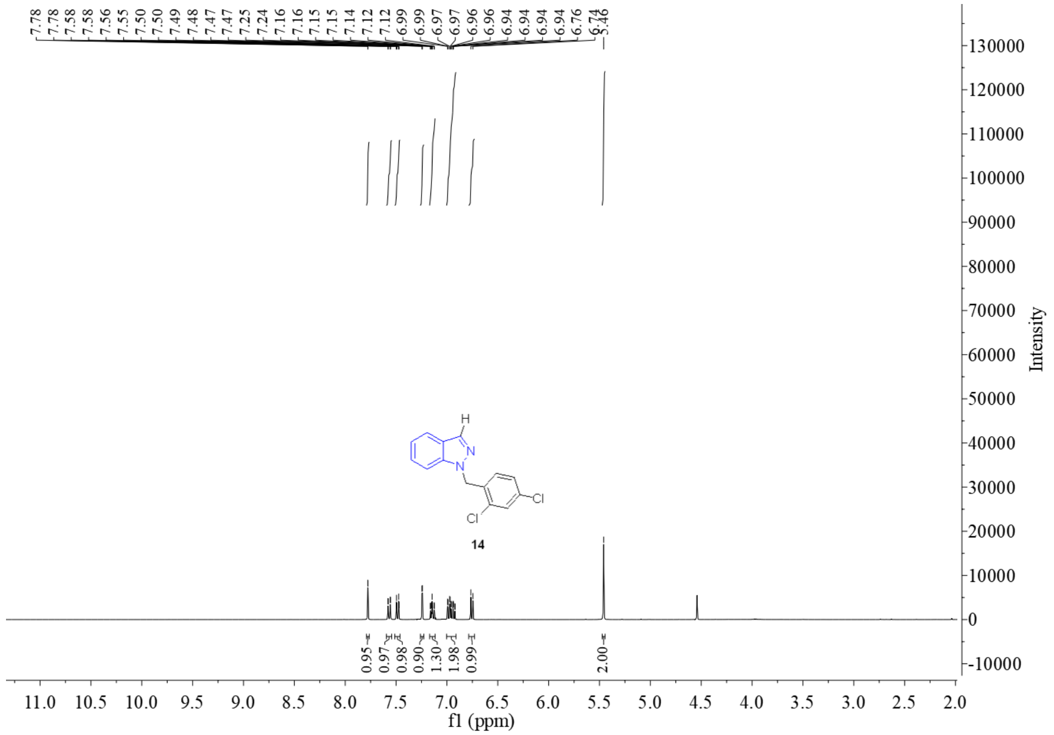

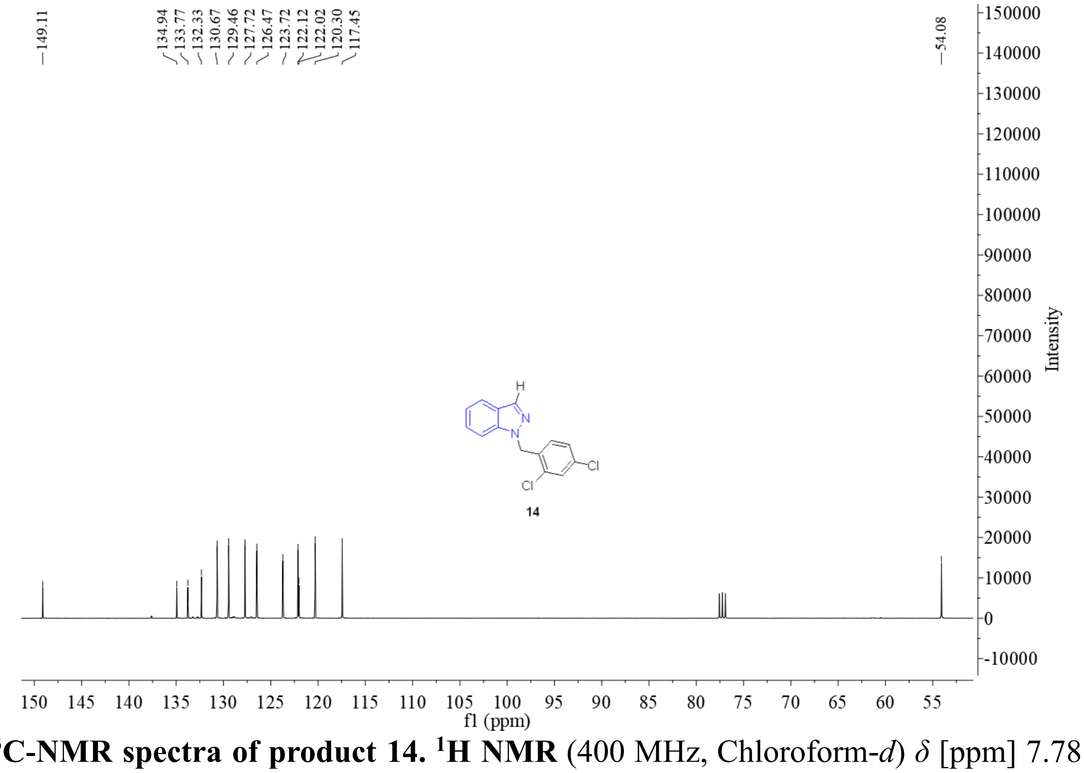

^{1}H, ^{13}C-NMR spectra of product 14. **^{1}H NMR** (400 MHz, Chloroform-*d*) δ [ppm] 7.78 (d, J = 0.7 Hz, 1H), 7.57 (dd, J = 8.8, 0.9 Hz, 1H), 7.48 (dt, J = 8.4, 1.0 Hz, 1H), 7.24 (d, J = 2.1 Hz, 1H), 7.17 – 7.12 (m, 1H), 7.00 – 6.91 (m, 2H), 6.75 (d, J = 8.3 Hz, 1H), 5.46 (s, 2H); **^{13}C NMR** (100 MHz, Chloroform-*d*) δ [ppm] 149.1, 134.9, 133.8, 132.3, 130.7, 129.5, 127.7, 126.5, 123.7, 122.1, 122.0, 120.3, 117.4, 54.1; **HRMS** (ESI) 277.0296, theoretical value for $C_{14}H_{10}Cl_2N_2$ $[M+H]^+$ 277.0299.

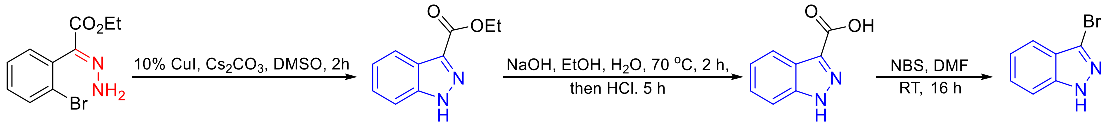

3u | **15** 81% yield | **16** 86% yield | **17** 78% yield

Product transformation by decarboxylation of ester to bromine.

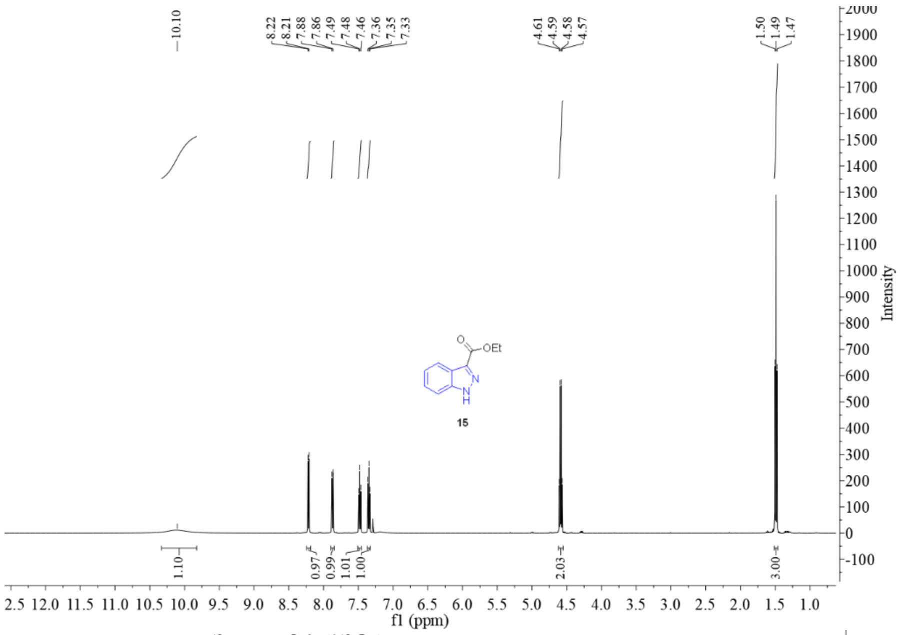

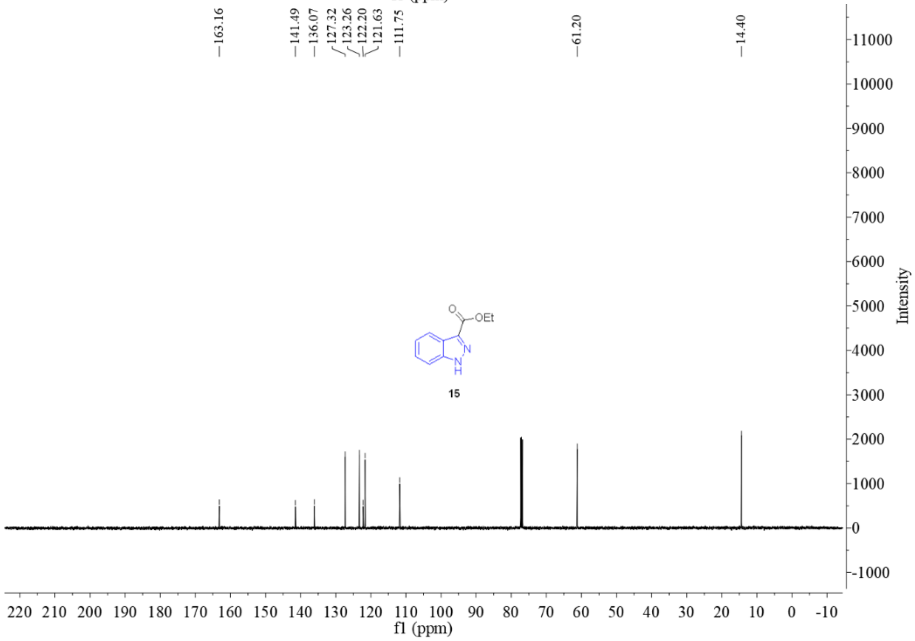

^{1}H, ^{13}C-NMR spectra of product 15. **^{1}H NMR** (500 MHz, Chloroform-*d*) δ [ppm] 10.10 (s, 1H), 8.22 (d, *J* = 8.2 Hz, 1H), 7.87 (d, *J* = 9.1 Hz, 1H), 7.48 (t, *J* = 7.7 Hz, 1H), 7.37 – 7.33 (m, 1H), 4.59 (q, *J* = 7.1 Hz, 2H), 1.49 (t, *J* = 7.1 Hz, 3H); **^{13}C NMR** (100 MHz, Chloroform-*d*) δ [ppm] 163.2, 141.5, 136.1, 127.3, 123.3, 122.2, 121.6, 111.8, 61.2, 14.4; **HRMS** (ESI) 189.0670, theoretical value for $C_{10}H_{10}N_2O_2$ [M-H]$^-$ 189.0664.

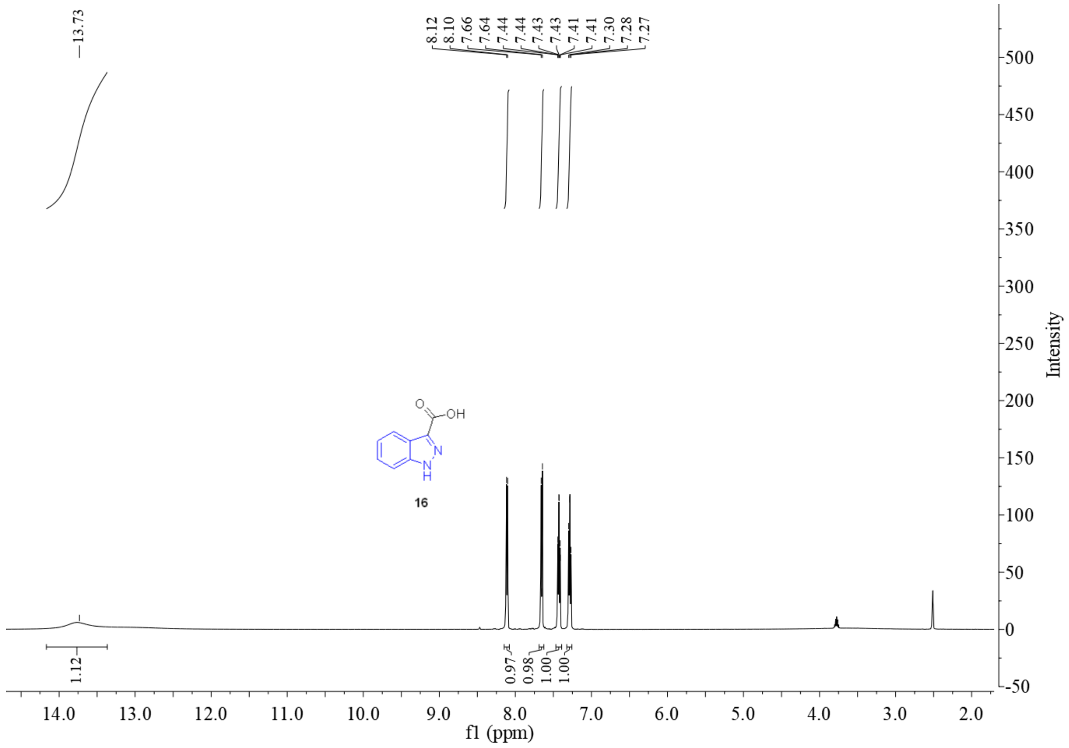

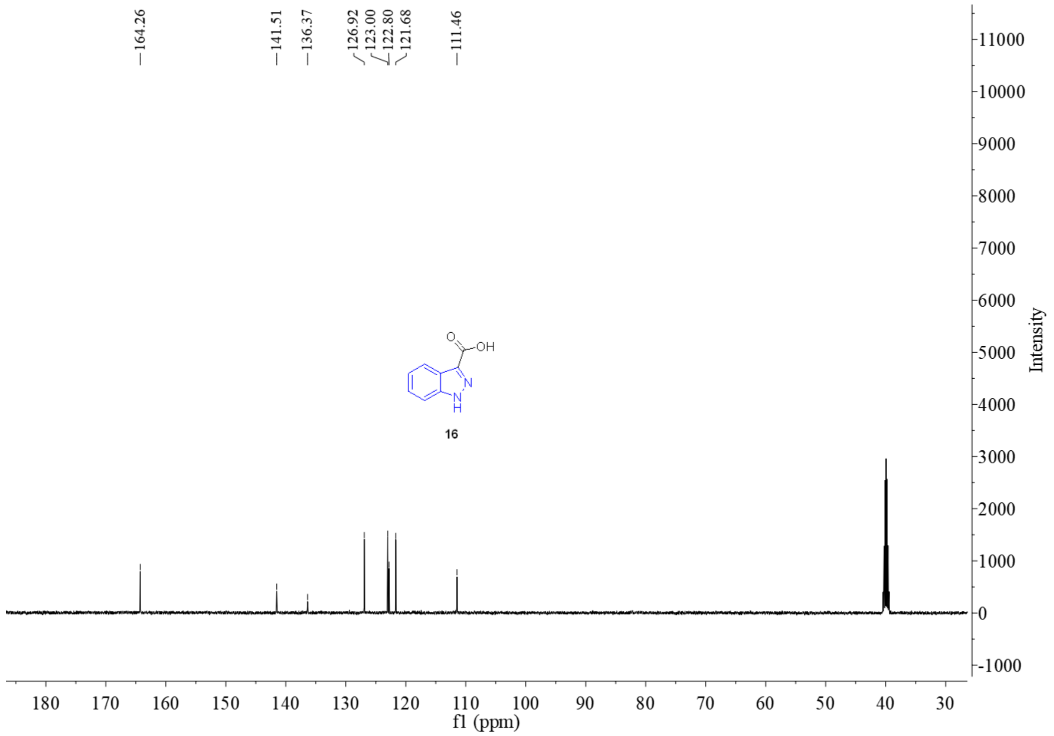

^{1}H, ^{13}C-NMR spectra of product 16. **^{1}H NMR** (500 MHz, DMSO-d_6) δ [ppm] 13.73 (s, 1H), 8.11 (d, J = 8.2 Hz, 1H), 7.65 (d, J = 8.4 Hz, 1H), 7.47 – 7.39 (m, 1H), 7.32 – 7.26 (m, 1H); **^{13}C NMR** (125 MHz, DMSO-d_6) δ [ppm] 164.3, 141.5, 136.4, 126.9, 123.0, 122.8, 121.7, 111.5; **HRMS** (ESI) 161.0353, theoretical value for $C_8H_6N_2O_2$ [M-H]$^-$161.0351.

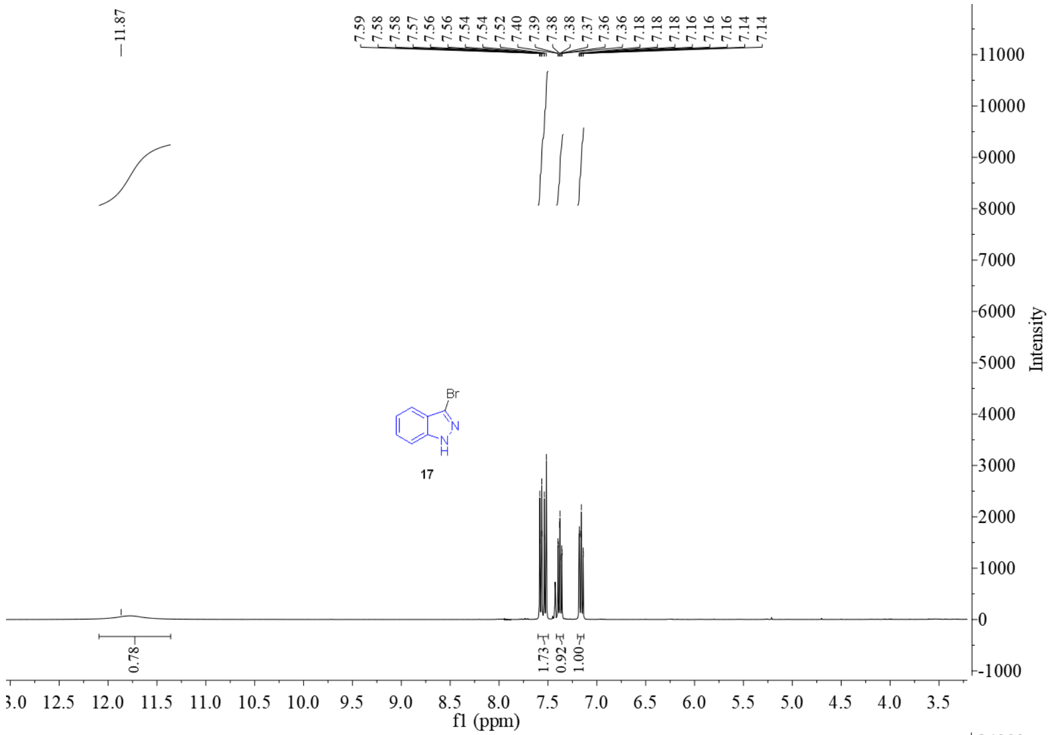

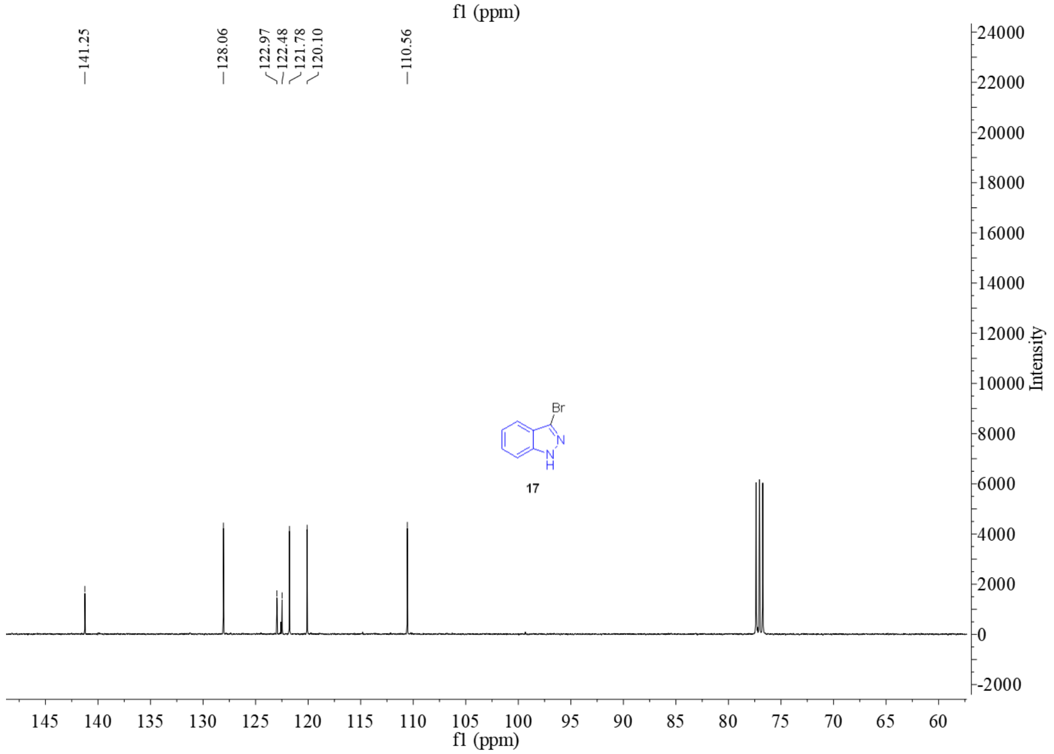

^{1}H, ^{13}C-NMR spectra of product 17. **^{1}H NMR** (400 MHz, Chloroform-*d*) δ [ppm] 11.87 (s, 1H), 7.60 – 7.50 (m, 2H), 7.38 (ddd, J = 8.4, 6.9, 1.0 Hz, 1H), 7.20 – 7.13 (m, 1H); **^{13}C NMR** (100 MHz, Chloroform-*d*) δ [ppm] 141.3, 128.1, 123.0, 122.5, 121.8, 120.1, 110.6; **HRMS** (ESI) 196.9711, theoretical value for $C_7H_5BrN_2$ $[M+H]^+$ 196.9714.

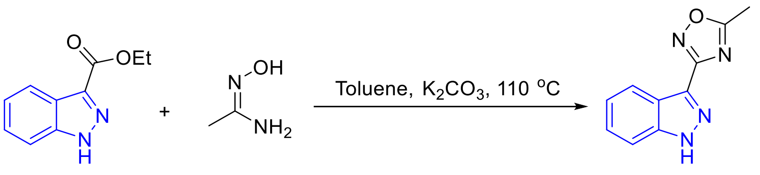

Product transformation to 1,2,4-oxadiazoles by cyclization of indazole carboxylic acid esters and amidoximes.

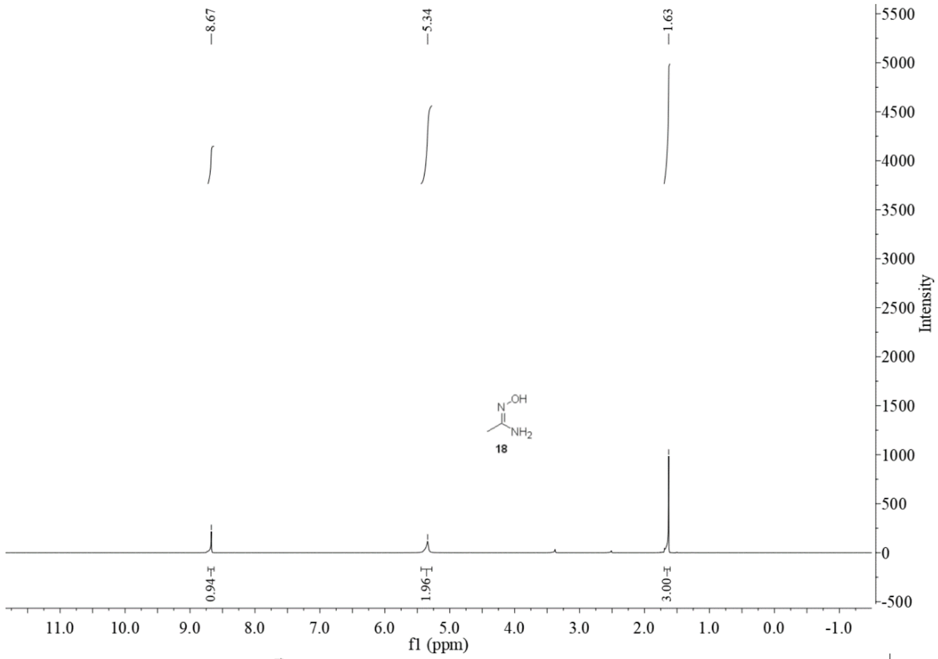

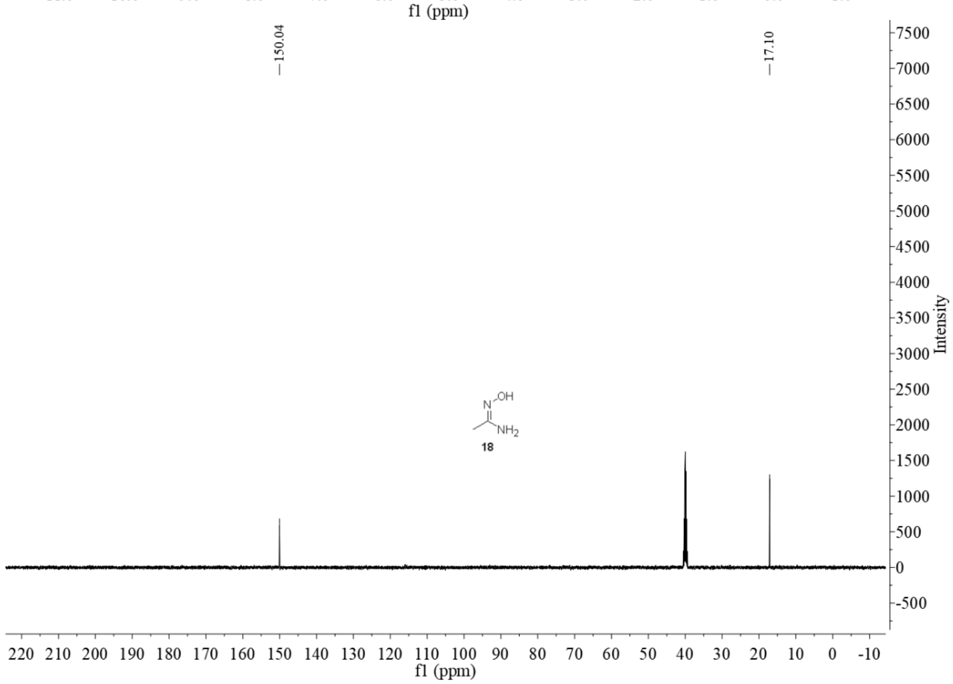

^{1}H, ^{13}C-NMR spectra of product 18. ^{1}H NMR (500 MHz, DMSO-*d*6) δ [ppm] 8.67 (s, 1H), 5.34 (s, 2H), 1.63 (s, 3H); **^{13}C NMR** (125 MHz, DMSO-*d*6) δ [ppm] 150.0, 17.1; **HRMS** (ESI) 75.0553, theoretical value for $C_2H_6N_2O$ $[M+H]^+$ 75.0558.

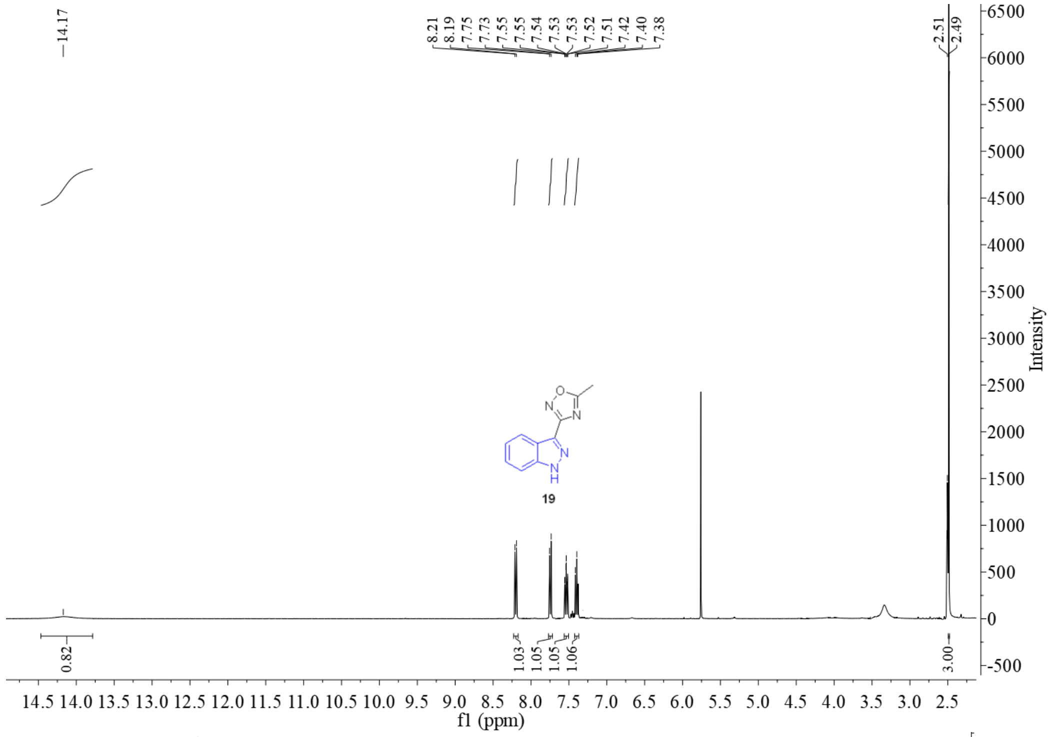

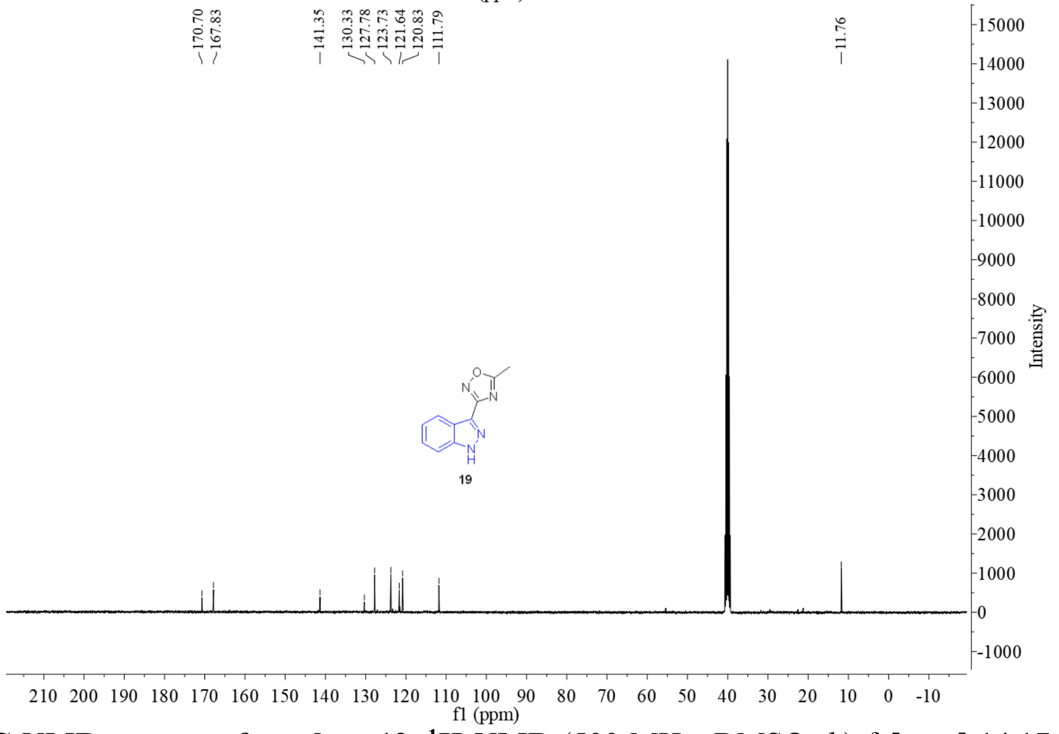

^{1}H, ^{13}C-NMR spectra of product 19. ^{1}H NMR (500 MHz, DMSO-d_6) δ [ppm] 14.17 (s, 1H), 8.20 (d, J = 8.2 Hz, 1H), 7.74 (d, J = 8.5 Hz, 1H), 7.53 (ddd, J = 8.3, 6.9, 1.0 Hz, 1H), 7.41 (d, J = 7.9 Hz, 1H), 2.49 (s, 3H); **^{13}C NMR** (125 MHz, DMSO-d_6) δ [ppm] 170.7, 167.8, 141.4, 130.3, 127.8, 123.7, 121.6, 120.8, 111.8, 11.8; **HRMS** (ESI) 201.0773, theoretical value for $C_{10}H_8N_4O$ $[M+H]^+$ 201.0776.

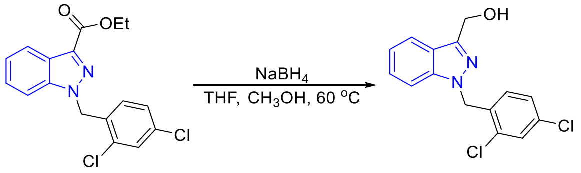

13 **20**

90% yield

Product transformation by reduction of carboxylic acid esters to alcohol.

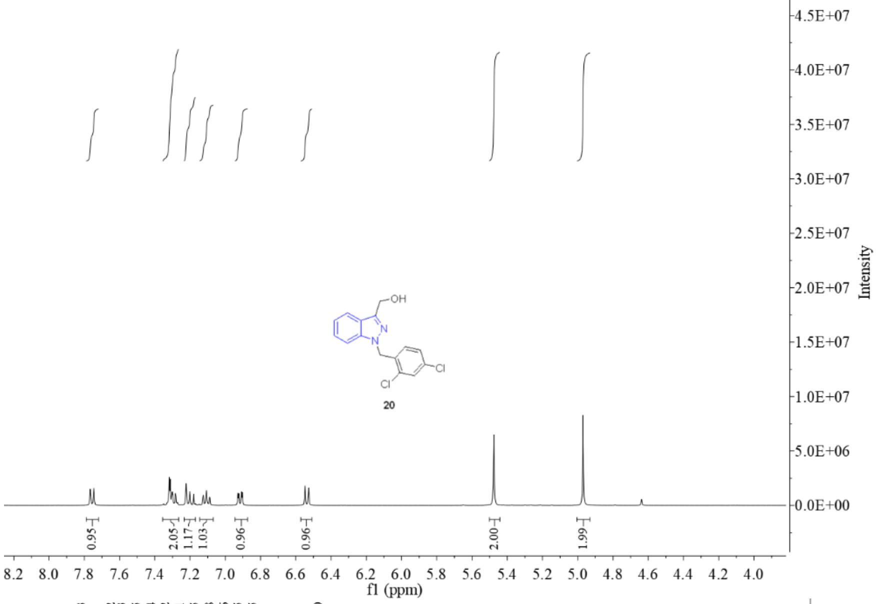

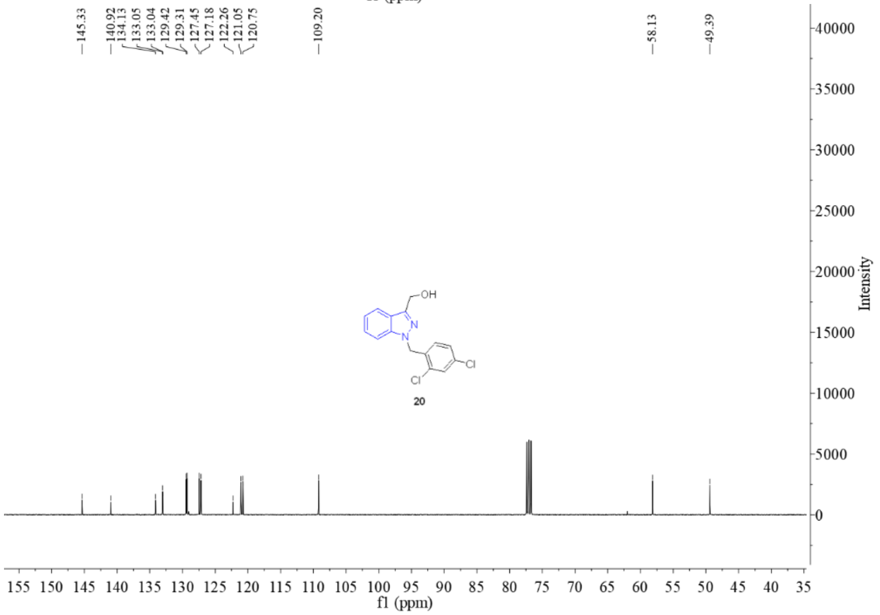

^{1}H, ^{13}C-NMR spectra of product 34. **^{1}H NMR** (400 MHz, Chloroform-*d*) δ [ppm] 7.76 (dt, J = 8.0, 1.1 Hz, 1H), 7.36 – 7.26 (m, 2H), 7.23 – 7.17 (m, 1H), 7.11 (ddd, J = 7.9, 6.8, 0.9 Hz, 1H), 6.92 (dd, J = 8.3, 2.1 Hz, 1H), 6.54 (d, J = 8.4 Hz, 1H), 5.48 (s, 2H), 4.97 (s, 2H); **^{13}C NMR** (100 MHz, Chloroform-*d*) δ [ppm] 145.3, 140.9, 134.1, 133.1, 133.0, 129.4, 129.3, 127.4, 127.2, 122.3, 121.1, 120.8, 109.2, 58.1, 49.4; **HRMS** (ESI) 329.0218, theoretical value for $C_{10}H_8N_4O$ [M+Na]$^+$ 329.0225.

Datablock I457 - ellipsoid plot

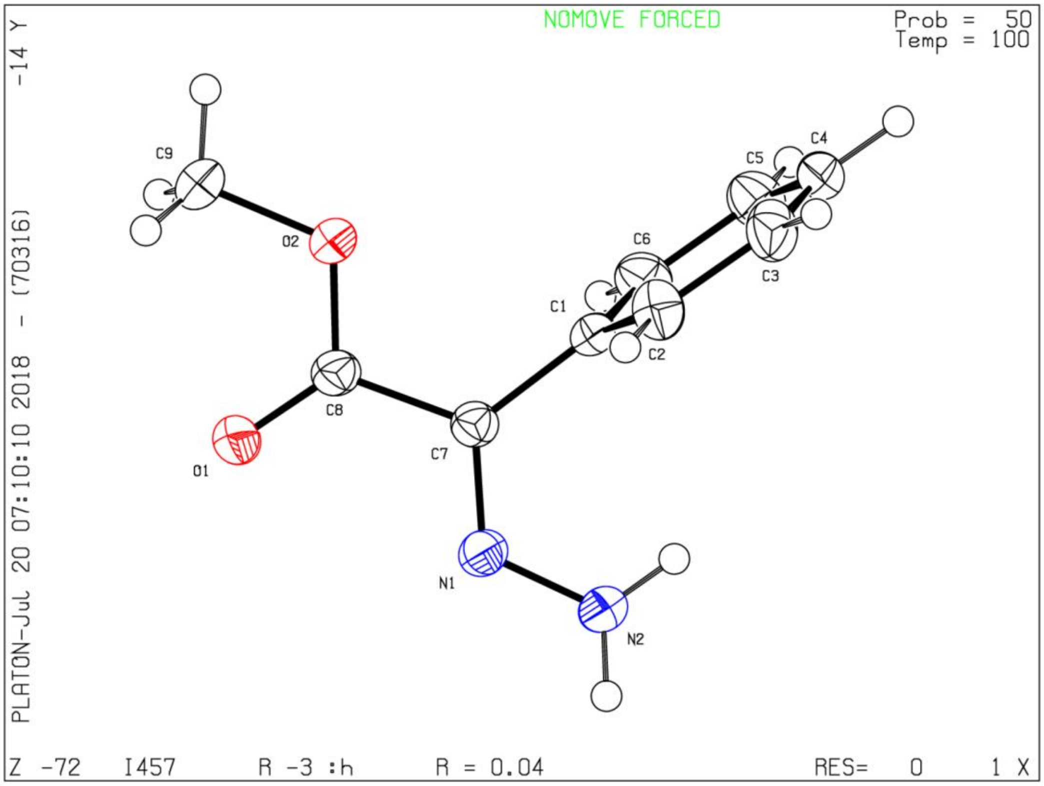

Crystallographic data of 3a.

The data have been deposited with the Cambridge Crystallographic Data Centre as supplementary publication number CCDC **1884857**. These data can be obtained free of charge from The Cambridge Crystallographic Data Centre via www.ccdc.cam.ac.uk/ data_request/cif.

checkCIF/PLATON report

You have not supplied any structure factors. As a result the full set of tests cannot be run.

THIS REPORT IS FOR GUIDANCE ONLY. IF USED AS PART OF A REVIEW PROCEDURE FOR PUBLICATION, IT SHOULD NOT REPLACE THE EXPERTISE OF AN EXPERIENCED CRYSTALLOGRAPHIC REFEREE.

No syntax errors found. CIF dictionary Interpreting this report

Datablock: I457

```
Bond precision:    C-C = 0.0019 A          Wavelength=1.54178

Cell:               a=13.811(1)      b=13.811(1)      c=25.3594(18)
                    alpha=90         beta=90          gamma=120
Temperature:        100 K

                 Calculated                  Reported
Volume           4189.1(8)                   4189.1(7)
Space group      R -3                        R -3 :h
Hall group       -R 3                        -R 3
Moiety formula   C9 H10 N2 O2                ?
Sum formula      C9 H10 N2 O2                C9 H10 N2 O2
Mr               178.19                      178.19
Dx,g cm-3        1.271                       1.271
Z                18                          18
Mu (mm-1)        0.760                       0.760
F000             1692.0                      1692.0
F000'            1697.47
h,k,lmax         16,16,30                    16,16,30
Nref             1774                        1772
Tmin,Tmax        0.729,0.779                 0.613,0.753
Tmin'            0.661

Correction method= # Reported T Limits: Tmin=0.613 Tmax=0.753
AbsCorr = MULTI-SCAN

Data completeness= 0.999           Theta(max)= 70.012

R(reflections)= 0.0397( 1700)      wR2(reflections)= 0.1116( 1772)

S = 1.042                   Npar= 127
```

The following ALERTS were generated. Each ALERT has the format
test-name_ALERT_alert-type_alert-level.
Click on the hyperlinks for more details of the test.

Alert level C

```
ABSTY02_ALERT_1_C  An _exptl_absorpt_correction_type has been given without
            a literature citation. This should be contained in the
            _exptl_absorpt_process_details field.
            Absorption correction given as Multi-scan
PLAT420_ALERT_2_C D-H Without Acceptor       N2       --H2A            Please Check
```

```
   0 ALERT level A = Most likely a serious problem - resolve or explain
   0 ALERT level B = A potentially serious problem, consider carefully
   2 ALERT level C = Check. Ensure it is not caused by an omission or oversight
   0 ALERT level G = General information/check it is not something unexpected

   1 ALERT type 1 CIF construction/syntax error, inconsistent or missing data
   1 ALERT type 2 Indicator that the structure model may be wrong or deficient
   0 ALERT type 3 Indicator that the structure quality may be low
   0 ALERT type 4 Improvement, methodology, query or suggestion
   0 ALERT type 5 Informative message, check
```

It is advisable to attempt to resolve as many as possible of the alerts in all categories. Often the minor alerts point to easily fixed oversights, errors and omissions in your CIF or refinement strategy, so attention to these fine details can be worthwhile. In order to resolve some of the more serious problems it may be necessary to carry out additional measurements or structure refinements. However, the purpose of your study may justify the reported deviations and the more serious of these should normally be commented upon in the discussion or experimental section of a paper or in the "special_details" fields of the CIF. checkCIF was carefully designed to identify outliers and unusual parameters, but every test has its limitations and alerts that are not important in a particular case may appear. Conversely, the absence of alerts does not guarantee there are no aspects of the results needing attention. It is up to the individual to critically assess their own results and, if necessary, seek expert advice.

Publication of your CIF in IUCr journals

A basic structural check has been run on your CIF. These basic checks will be run on all CIFs submitted for publication in IUCr journals (*Acta Crystallographica*, *Journal of Applied Crystallography*, *Journal of Synchrotron Radiation*); however, if you intend to submit to *Acta Crystallographica Section C* or *E* or *IUCrData*, you should make sure that full publication checks are run on the final version of your CIF prior to submission.

Publication of your CIF in other journals

Please refer to the *Notes for Authors* of the relevant journal for any special instructions relating to CIF submission.

PLATON version of 14/07/2018; check.def file version of 05/06/2018

The CheckCIF report of 3a.

Datablock I645 - ellipsoid plot

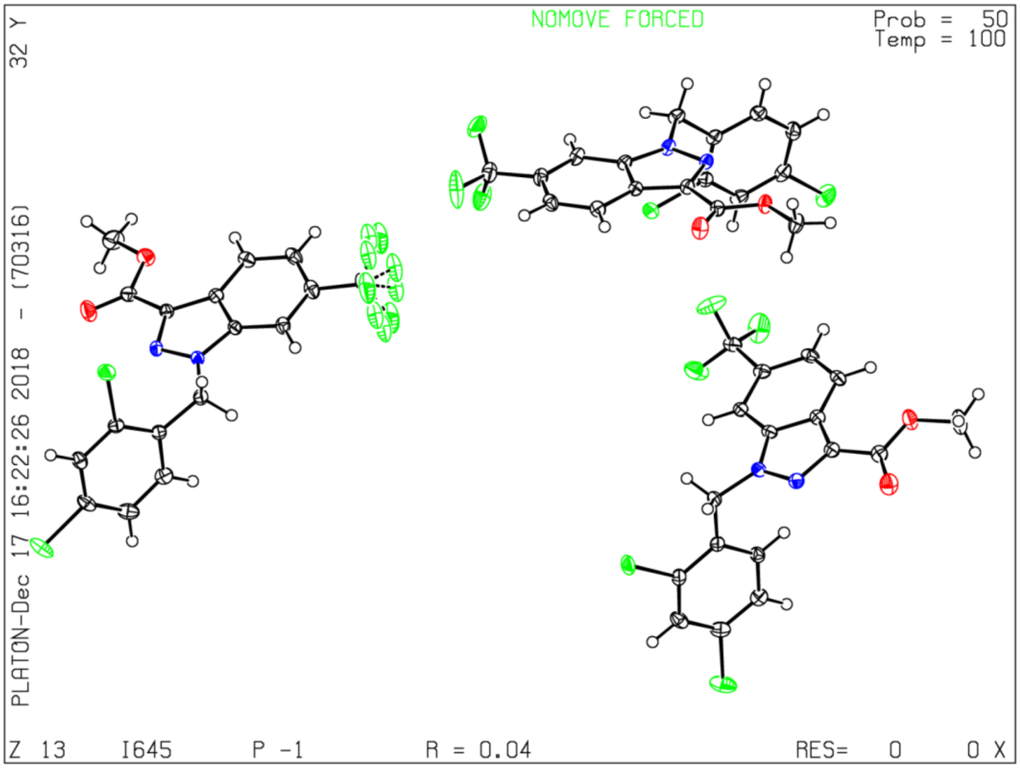

Crystallographic data of 6.

Crystallographic data for **6** have been deposited with the Cambridge Crystallographic Data Centre as supplementary publication number CCDC **1884861**. These data can be obtained free of charge from The Cambridge Crystallographic Data Centre via www.ccdc.cam.ac.uk/ data_request/cif.

checkCIF/PLATON report

Structure factors have been supplied for datablock(s) I645

THIS REPORT IS FOR GUIDANCE ONLY. IF USED AS PART OF A REVIEW PROCEDURE FOR PUBLICATION, IT SHOULD NOT REPLACE THE EXPERTISE OF AN EXPERIENCED CRYSTALLOGRAPHIC REFEREE.

No syntax errors found.   CIF dictionary   Interpreting this report

Datablock: I645

```
Bond precision:  C-C = 0.0030 A          Wavelength=1.54178

Cell:          a=12.2967(5)      b=13.8394(6)      c=16.7358(7)
               alpha=96.299(1)   beta=91.727(1)    gamma=115.695(1)
Temperature:   100 K

                Calculated                  Reported
Volume          2541.34(19)                 2541.33(19)
Space group     P -1                        P -1
Hall group      -P 1                        -P 1
Moiety formula  C17 H11 Cl2 F3 N2 O2        ?
Sum formula     C17 H11 Cl2 F3 N2 O2        C17 H11 Cl2 F3 N2 O2
Mr              403.18                      403.18
Dx,g cm-3       1.581                       1.581
Z               6                           6
Mu (mm-1)       3.887                       3.887
F000            1224.0                      1224.0
F000'           1232.37
h,k,lmax        14,16,19                    14,16,19
Nref            9002                        8920
Tmin,Tmax       0.313,0.456                 0.507,0.753
Tmin'           0.085

Correction method= # Reported T Limits: Tmin=0.507 Tmax=0.753
AbsCorr = MULTI-SCAN

Data completeness= 0.991           Theta(max)= 66.668

R(reflections)= 0.0365( 8684)      wR2(reflections)= 0.0919( 8920)

S = 1.029                    Npar= 728
```

The following ALERTS were generated. Each ALERT has the format
test-name_ALERT_alert-type_alert-level.
Click on the hyperlinks for more details of the test.

Alert level C

```
PLAT911_ALERT_3_C Missing FCF Refl Between Thmin & STh/L=    0.596         81 Report
```

Alert level G

```
PLAT002_ALERT_2_G Number of Distance or Angle Restraints on AtSite         10 Note
PLAT003_ALERT_2_G Number of Uiso or Uij Restrained non-H Atoms ...          6 Report
PLAT154_ALERT_1_G The s.u.'s on the Cell Angles are Equal ..(Note)      0.001 Degree
PLAT171_ALERT_4_G The CIF-Embedded .res File Contains EADP Records          3 Report
PLAT175_ALERT_4_G The CIF-Embedded .res File Contains SAME Records          2 Report
PLAT176_ALERT_4_G The CIF-Embedded .res File Contains SADI Records          2 Report
PLAT177_ALERT_4_G The CIF-Embedded .res File Contains DELU Records          1 Report
PLAT178_ALERT_4_G The CIF-Embedded .res File Contains SIMU Records          1 Report
PLAT230_ALERT_2_G Hirshfeld Test Diff for    F7X      --C35      .       10.3 s.u.
PLAT230_ALERT_2_G Hirshfeld Test Diff for    F8       --C35      .        6.9 s.u.
PLAT230_ALERT_2_G Hirshfeld Test Diff for    F8A      --C35      .       11.5 s.u.
PLAT230_ALERT_2_G Hirshfeld Test Diff for    F8X      --C35      .       13.0 s.u.
PLAT230_ALERT_2_G Hirshfeld Test Diff for    F9       --C35      .       13.9 s.u.
PLAT230_ALERT_2_G Hirshfeld Test Diff for    F9A      --C35      .        8.2 s.u.
PLAT230_ALERT_2_G Hirshfeld Test Diff for    F9X      --C35      .        7.0 s.u.
PLAT242_ALERT_2_G Low    'MainMol' Ueq as Compared to Neighbors of        C35 Check
PLAT242_ALERT_2_G Low    'MainMol' Ueq as Compared to Neighbors of        C18 Check
PLAT301_ALERT_3_G Main Residue  Disorder ..............(Resd  1  )        12% Note
PLAT431_ALERT_2_G Short Inter HL..A Contact  Cl3      ..O5       .       3.17 Ang.
                                                -x,1-y,1-z  =      2_566 Check
PLAT434_ALERT_2_G Short Inter HL..HL Contact Cl3      ..F1               3.12 Ang.
                                                     x,y,z  =      1_555 Check
PLAT860_ALERT_3_G Number of Least-Squares Restraints ............          52 Note
PLAT909_ALERT_3_G Percentage of I>2sig(I) Data at Theta(Max) Still        94% Note
PLAT910_ALERT_3_G Missing # of FCF Reflection(s) Below Theta(Min).          1 Note
PLAT978_ALERT_2_G Number C-C Bonds with Positive Residual Density.         10 Info
```

```
   0 ALERT level A = Most likely a serious problem - resolve or explain
   0 ALERT level B = A potentially serious problem, consider carefully
   1 ALERT level C = Check. Ensure it is not caused by an omission or oversight
  24 ALERT level G = General information/check it is not something unexpected

   1 ALERT type 1 CIF construction/syntax error, inconsistent or missing data
  14 ALERT type 2 Indicator that the structure model may be wrong or deficient
   5 ALERT type 3 Indicator that the structure quality may be low
   5 ALERT type 4 Improvement, methodology, query or suggestion
   0 ALERT type 5 Informative message, check
```

It is advisable to attempt to resolve as many as possible of the alerts in all categories. Often the minor alerts point to easily fixed oversights, errors and omissions in your CIF or refinement strategy, so attention to these fine details can be worthwhile. In order to resolve some of the more serious problems it may be necessary to carry out additional measurements or structure refinements. However, the purpose of your study may justify the reported deviations and the more serious of these should normally be commented upon in the discussion or experimental section of a paper or in the "special_details" fields of the CIF. checkCIF was carefully designed to identify outliers and unusual parameters, but every test has its limitations and alerts that are not important in a particular case may appear. Conversely, the absence of alerts does not guarantee there are no aspects of the results needing attention. It is up to the individual to critically assess their own results and, if necessary, seek expert advice.

Publication of your CIF in IUCr journals

A basic structural check has been run on your CIF. These basic checks will be run on all CIFs submitted for publication in IUCr journals (*Acta Crystallographica*, *Journal of Applied Crystallography*, *Journal of Synchrotron Radiation*); however, if you intend to submit to *Acta Crystallographica Section C* or *E* or *IUCrData*, you should make sure that full publication checks are run on the final version of your CIF prior to submission.

Publication of your CIF in other journals

Please refer to the *Notes for Authors* of the relevant journal for any special instructions relating to CIF submission.

PLATON version of 13/12/2018; check.def file version of 11/12/2018

The CheckCIF report of 6.

Datablock I626 - ellipsoid plot

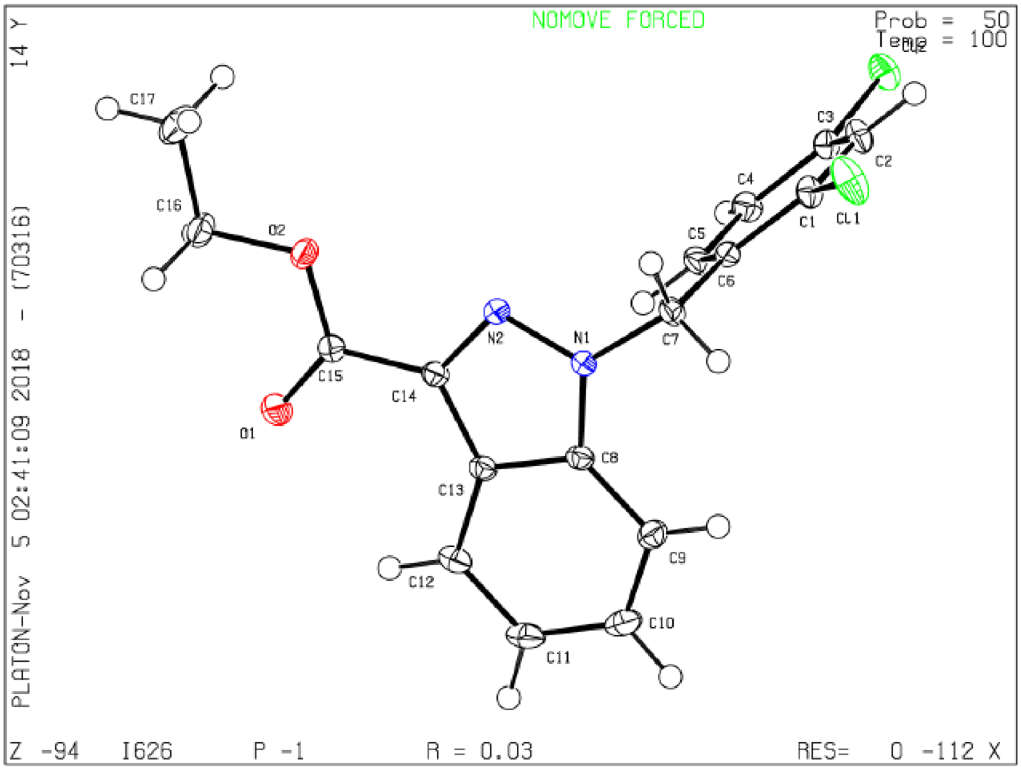

Crystallographic data of 13.

Crystallographic data for **13** have been deposited with the Cambridge Crystallographic Data Centre as supplementary publication number CCDC **1884860**. These data can be obtained free of charge from The Cambridge Crystallographic Data Centre via www.ccdc.cam.ac.uk/data_request/cif.

checkCIF/PLATON report

You have not supplied any structure factors. As a result the full set of tests cannot be run.

THIS REPORT IS FOR GUIDANCE ONLY. IF USED AS PART OF A REVIEW PROCEDURE FOR PUBLICATION, IT SHOULD NOT REPLACE THE EXPERTISE OF AN EXPERIENCED CRYSTALLOGRAPHIC REFEREE.

No syntax errors found.   CIF dictionary   Interpreting this report

Datablock: I626

```
Bond precision:   C-C = 0.0016 A          Wavelength=0.71073

Cell:          a=8.1430(5)       b=9.7173(6)       c=11.4040(7)
               alpha=90.722(2)   beta=107.647(2)   gamma=110.373(2)
Temperature:   100 K

                Calculated                  Reported
Volume          799.04(9)                   799.04(9)
Space group     P -1                        P -1
Hall group      : -P 1                      -P 1
Moiety formula  C17 H14 Cl2 N2 O2           ?
Sum formula     C17 H14 Cl2 N2 O2           C17 H14 Cl2 N2 O2
Mr              349.20                      349.20
Dx,g cm-3       1.451                       1.451
Z               2                           2
Mu (mm-1)       0.417                       0.417
F000            360.0                       360.0
F000'           360.72
h,k,lmax        11,13,15                    11,13,15
Nref            4507                        4500
Tmin,Tmax       0.805,0.848                 0.672,0.746
Tmin'           0.805

Correction method= # Reported T Limits: Tmin=0.672 Tmax=0.746
AbsCorr = MULTI-SCAN

Data completeness= 0.998          Theta(max)= 29.631

R(reflections)= 0.0302( 4207)     wR2(reflections)= 0.0808( 4500)

S = 1.052                   Npar= 209
```

The following ALERTS were generated. Each ALERT has the format
test-name_ALERT_alert-type_alert-level.
Click on the hyperlinks for more details of the test.

Alert level G

```
PLAT154_ALERT_1_G The s.u.'s on the Cell Angles are Equal ..(Note)      0.002 Degree
PLAT431_ALERT_2_G Short Inter HL..A Contact  Cl2      ..O1       .       3.09 Ang.
                                                  1+x,y,1+z  =      1_656 Check
```

```
   0 ALERT level A = Most likely a serious problem - resolve or explain
   0 ALERT level B = A potentially serious problem, consider carefully
   0 ALERT level C = Check. Ensure it is not caused by an omission or oversight
   2 ALERT level G = General information/check it is not something unexpected

   1 ALERT type 1 CIF construction/syntax error, inconsistent or missing data
   1 ALERT type 2 Indicator that the structure model may be wrong or deficient
   0 ALERT type 3 Indicator that the structure quality may be low
   0 ALERT type 4 Improvement, methodology, query or suggestion
   0 ALERT type 5 Informative message, check
```

It is advisable to attempt to resolve as many as possible of the alerts in all categories. Often the minor alerts point to easily fixed oversights, errors and omissions in your CIF or refinement strategy, so attention to these fine details can be worthwhile. In order to resolve some of the more serious problems it may be necessary to carry out additional measurements or structure refinements. However, the purpose of your study may justify the reported deviations and the more serious of these should normally be commented upon in the discussion or experimental section of a paper or in the "special_details" fields of the CIF. checkCIF was carefully designed to identify outliers and unusual parameters, but every test has its limitations and alerts that are not important in a particular case may appear. Conversely, the absence of alerts does not guarantee there are no aspects of the results needing attention. It is up to the individual to critically assess their own results and, if necessary, seek expert advice.

Publication of your CIF in IUCr journals

A basic structural check has been run on your CIF. These basic checks will be run on all CIFs submitted for publication in IUCr journals (*Acta Crystallographica*, *Journal of Applied Crystallography*, *Journal of Synchrotron Radiation*); however, if you intend to submit to *Acta Crystallographica Section C* or *E* or *IUCrData*, you should make sure that full publication checks are run on the final version of your CIF prior to submission.

Publication of your CIF in other journals

Please refer to the *Notes for Authors* of the relevant journal for any special instructions relating to CIF submission.

PLATON version of 19/10/2018; check.def file version of 15/10/2018

The CheckCIF report of 13.
